# Structure-Based Design of Covalent SARS-CoV‑2 Main Protease Inhibitors Targeting the Nirmatrelvir-Resistant E166 Mutants

**DOI:** 10.1021/jacsau.5c01178

**Published:** 2026-01-12

**Authors:** Zhengjun Cai, Navita Kohaal, Kyriakos Georgiou, Xueying Liang, Xiang Chi, Haozhou Tan, Bin Tan, Kan Li, Guangjin Fan, George Lambrinidis, Antonios Kolocouris, Xufang Deng, Yu Chen, Jun Wang

**Affiliations:** † Department of Medicinal Chemistry, Ernest Mario School of Pharmacy, 242612 Rutgers, the State University of New Jersey, Piscataway, New Jersey 08854, United States; ‡ Department of Molecular Medicine, Morsani College of Medicine, 7831 University of South Florida, Tampa, Florida 33612, United States; § Laboratory of Medicinal Chemistry, Section of Pharmaceutical Chemistry, Department of Pharmacy, School of Health Sciences, 69232 National and Kapodistrian University of Athens, Panepistimiopolis-Zografou, Athens 15771, Greece; ∥ Department of Physiological Sciences, College of Veterinary Medicine, 7618 Oklahoma State University, Stillwater, Oklahoma 74078, United States; ⊥ Oklahoma Center for Respiratory and Infectious Diseases, Oklahoma State University, Stillwater, Oklahoma 74078, United States

**Keywords:** SARS-CoV-2, main protease, E166V, antiviral, drug resistance

## Abstract

The COVID-19 pandemic spurred the rapid development of nirmatrelvir, a main protease (M^pro^) inhibitor now widely prescribed as part of Paxlovid (nirmatrelvir plus ritonavir). However, increasing use has raised concerns about drug resistance. Resistance selection studies have identified multiple M^pro^ mutations, with E166V emerging as a particularly resistant variant. Sequencing data from COVID-19 patients confirms E166V as a clinically relevant mutation, and importantly, this substitution also confers cross-resistance to several next-generation M^pro^ inhibitors under development. In response, this study reports the rational design of inhibitors active against nirmatrelvir-resistant E166V/A mutants. The lead candidate, **Jun13698**, shows potent inhibition of both wild-type M^pro^ and the E166V/A mutants. Structural studies and molecular dynamics simulations reveal that **Jun13698** forms stable complexes with wild-type and mutant proteases, consistent with its potent enzymatic and antiviral activity. Together, these findings position **Jun13698** as a promising next-generation M^pro^ inhibitor capable of overcoming clinically relevant nirmatrelvir resistance.

## Graphical abstract

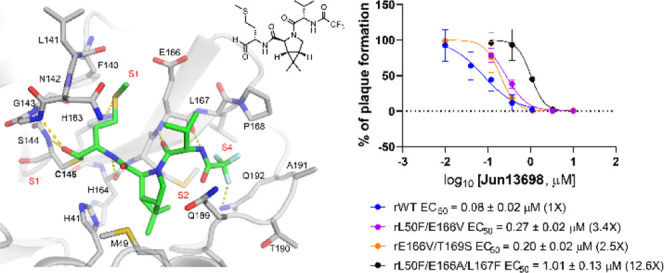

## Introduction

The outbreak of coronavirus disease 2019 (COVID-19), caused by the novel coronavirus SARS-CoV-2, has posed an unprecedented global health and economic crisis. The World Health Organization (WHO) declared a global pandemic in March 2020. The virus spreads mainly through respiratory droplets and aerosols, and because many infected individuals remain asymptomatic, effective contact tracing becomes challenging.^1^ While early strains such as the original Wuhan variant and subsequent variants like Delta were associated with high rates of severe disease and hospitalization, current circulating variants in the Omicron lineage have demonstrated increased transmissibility but generally reduced pathogenicity.^2,3^

To combat the spread and severity of COVID-19, the scientific community rapidly mobilized efforts to develop vaccines, diagnostic tools, and therapeutic interventions, including small molecules and antibodies.^4^ One of the first antivirals to gain FDA approval was the repurposed drug remdesivir, a nucleotide analog that inhibits the RNA-dependent RNA polymerase (RdRp) of SARS-CoV-2, thereby blocking viral replication.^5^ Later, molnupiravir, a nucleoside analog acting as a mutagen,^6^ and nirmatrelvir, a main protease (M^pro^) inhibitor administered in combination with ritonavir (*Paxlovid*), have expanded the arsenal of available COVID-19 treatments.^7,8^ These drugs have demonstrated effectiveness in reducing hospitalization and mortality, particularly when given during the early stages of infection. However, as SARS-CoV-2 continues to evolve—both with and without drug selection pressure—and persists in human and animal populations,^9–11^ there is a clear and ongoing need for additional antivirals to address emerging variants and potential drug-resistant mutants.

Among the list of SARS-CoV-2 antiviral drug targets, M^pro^ is the most extensively explored.^12^ M^pro^ is a viral cysteine protease that cleaves the viral polyproteins pp1a and pp1ab at more than 11 sites, generating individual nonstructural proteins essential for viral replication.^13^ M^pro^ is highly conserved among viruses in the coronavirus family, and M^pro^ inhibitors have shown broad-spectrum antiviral activity against SARS-CoV-2, SARS-CoV, MERS-CoV, HCoV-OC43, HCoV-229E, and HCoV-NL63.^7,14,15^

In addition to nirmatrelvir, a series of covalent and noncovalent M^pro^ inhibitors are at the preclinical and clinical development stages or approved outside the United States.^16^ Representative examples include ensitrelvir,^17,18^ TKB245,^19^ ML2006a4,^20^ Ibuzatrelvir,^21^ EDP-235,^22^ and simnotrelvir (approved in China).^23^ While these newer generations of M^pro^ inhibitors generally demonstrate improved antiviral efficacy or pharmacokinetic (PK) properties, they bind to the same site and share structural similarities with nirmatrelvir. As such, there is a concern about cross-resistance.

In parallel to the antiviral drug development, there is a significant interest in predicting and validating drug-resistant mutants. Our previous structure-based prediction identified several nirmatrelvir-resistant hot spots in M^pro^, including S144, M165, E166, and H172.^11^ Our results, together with those of others, have shown that mutants at these hotspot residues, including S144A/M, M165T, E166A/G/V, and H172Q/Y, exhibit a high degree of drug resistance without significantly compromising the enzymatic activity of M^pro^. The physiological relevance of these drug-resistant mutants was further validated in independent studies by other groups using infectious SARS-CoV-2 virus, reporter virus, or surrogate systems in mammalian cells, bacteria, or yeast.^24–28^ For example, Iketani et al. performed an in vitro serial viral passage experiment under the drug selection pressure of nirmatrelvir in Vero E6 cells and Huh7-ACE2 cells. They identified S144A, E166V/A, and H172Y/Q as high-frequency mutations.^25^ Zhu et al. conducted similar experiments and uncovered S144A as a drug-resistant mutation.^29^ Jochmans et al. conducted a passage experiment with a preclinical M^pro^ inhibitor, ALG-097161, and identified E166A as the drug-resistant mutant.^30^ They further showed that E166A had cross-resistance to nirmatrelvir. Zhou et al. similarly identified E166V through a serial viral passage experiment in Vero E6 cells and validated the drug resistance using recombinant viruses.^28^

More concerning are the findings of drug-resistant M^pro^ mutations from human patient samples. In individuals treated with nirmatrelvir, especially those who are immunocompromised, the E166V mutation is the most frequently identified, followed by E166A.^29,31–34^ E166V displayed cross-resistance to pyrrolidone-containing M^pro^ inhibitors, including TKB-245^19^, ML2006a4,^20^ Ibuzatrelvir,^21^ EDP-235,^22^ and simnotrelvir^23^ (Figure 1).

**Figure 1:**
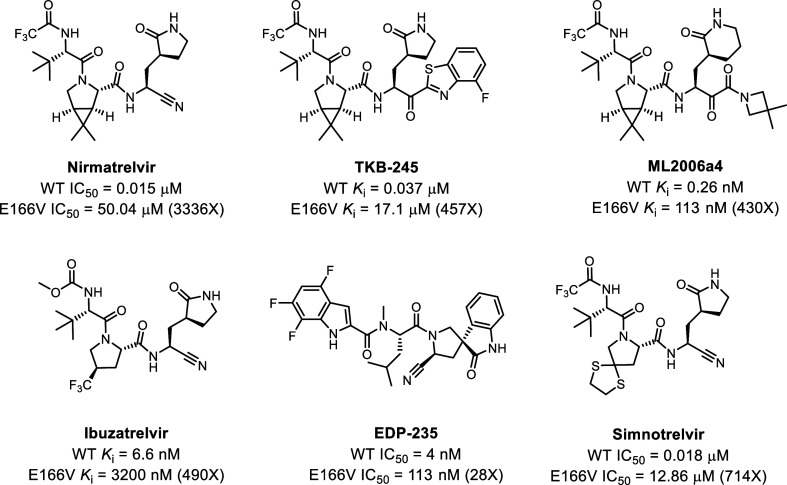
Structures and activities of representative pyrrolidone-containing SARS-CoV-2 M^pro^ inhibitors with cross-resistance to nirmatrelvir. It is worth noting that the enzymatic assay was performed under different conditions. Therefore, comparing the IC_50_ or K_i_ values from different studies is not meaningful.

Given the clinical relevance of the E166V mutant, this study aims to design M^pro^ inhibitors targeting both the E166V mutant and the wild-type (WT) protein. The lead compound **Jun13698**, which contains the methionine side chain in the P1 substituent instead of the pyrrolidinone moiety, demonstrated potent inhibition against the M^pro^ E166V in the enzymatic assay. The antiviral activity of **Jun13698** was further validated in the antiviral assay using recombinant SARS-CoV-2 viruses containing single, double, or triple E166V/A M^pro^ mutants.

## Results and Discussion

### Structure-Based Design of SARS-CoV-2 M^pro^ Inhibitors Targeting the E166 Mutants

The X-ray crystal structure of SARS-CoV-2 M^pro^ in complex with nirmatrelvir (Figure 2A, PDB: 7RFS) reveals that residue E166 plays a critical role in inhibitor binding.^7^ It forms three hydrogen bonds with nirmatrelvir: one between the E166 side-chain carboxylate oxygen and the amide NH of the P1 pyrrolidone, and two between the E166 main-chain carbonyl and NH with the main-chain NH and carbonyl of the P3 *tert*-butyl glycine moiety, respectively (Figure 2A). This triad of hydrogen bonds is conserved among several other M^pro^ inhibitors currently in development, including TKB-245,^19^ ML2006a4,^20^ Ibuzatrelvir,^21^ EDP-235,^22^ and simnotrelvir^23^ (Figure 2B–F). The critical role of E166 in inhibitor binding is further supported by the observed cross-resistance of the E166V mutant to M^pro^ inhibitors bearing either 5-membered pyrrolidone or 6-membered piperidone P1 substituents (Figure 1). The substitution of the polar E166 residue with hydrophobic amino acids, such as valine, alanine, or glycine, as emerged in patients treated with nirmatrelvir, eliminates the side-chain hydrogen bond, resulting in a reduced binding affinity and drug resistance.^11^

**Figure 2:**
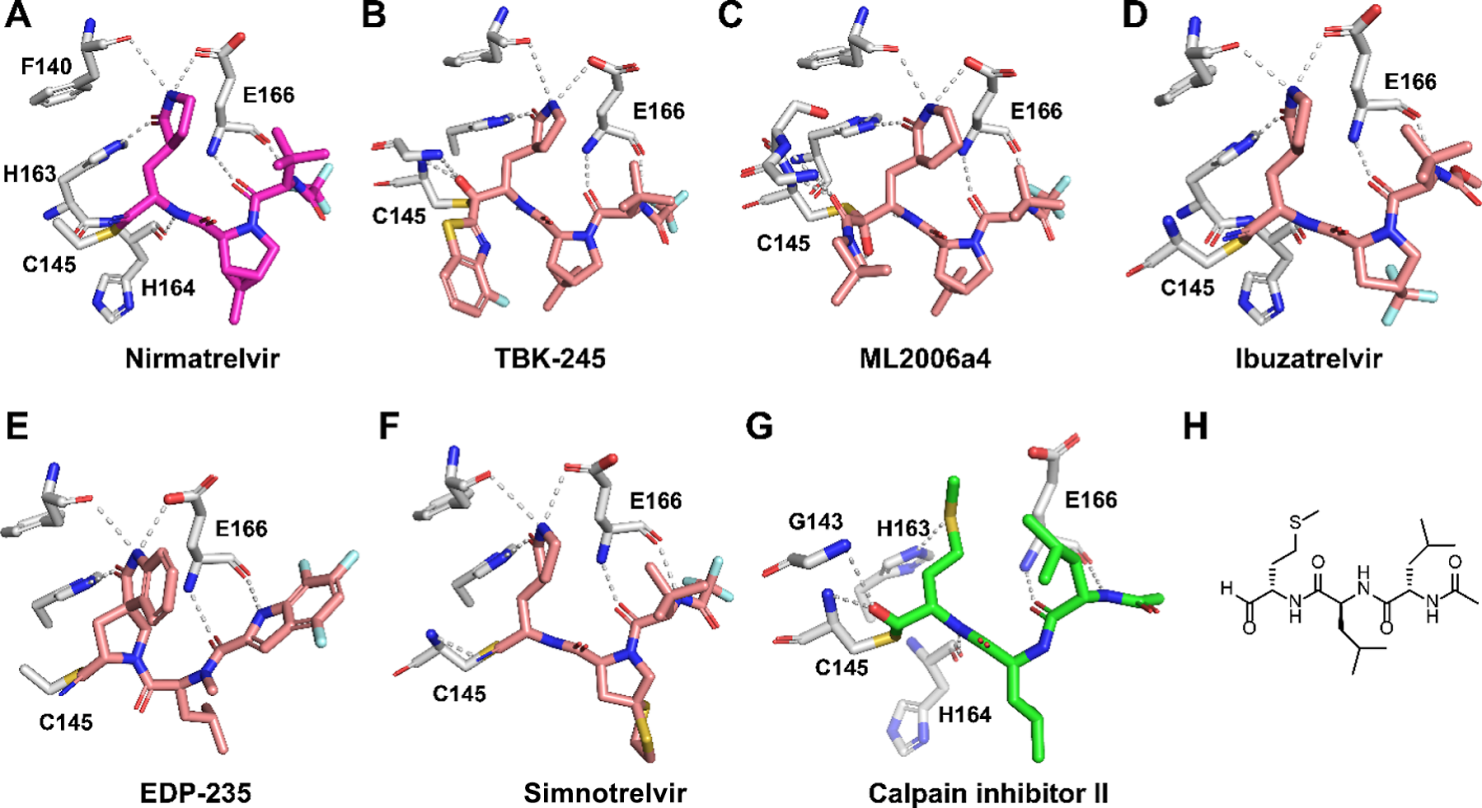
X-ray crystal structure of SARS-CoV-2 M^pro^ with inhibitors. X-ray crystal structures of SARS-CoV-2 M^pro^ with nirmatrelvir (PDB: 7RFS) (A), TKB-245 (PDB: 9ARQ) (B), ML2006a4 (PDB: 8F02) (C), ibuzatrelvir (PDB: 8V6U) (D), EDP-235 (PDB: 8VDJ) (E), simontrelvir (PDB: 8IGX) (F), and calpain inhibitor II (PDB: 6XA4) (G). (H) Chemical structure of calpain inhibitor II.

To design inhibitors effective against E166 mutants (E166V, E166A and E166G), we hypothesize that effective inhibitors must satisfy two key criteria: first, the P1 substituent should avoid forming a hydrogen bond with E166, thereby removing dependence on the side-chain interaction; second, the P1 substituent should be hydrophobic to retain favorable hydrophobic interactions with the mutated V166, A166, or G166 residues. In our search for M^pro^ inhibitors that fulfill these two criteria, we focused on calpain inhibitor II—a peptidyl aldehyde identified initially by us as an M^pro^ inhibitor through drug repurposing screening (Figure 2G,H).^35^ We subsequently solved the X-ray crystal structure of M^pro^ in complex with calpain inhibitor II (Figure 2G,H), which revealed that the P1 methionine side chain occupies the S1 subsite without engaging in a hydrogen bond with the E166 side chain.^15^ Instead, the P1 methionine side chain extends into the S1 subsite, where its sulfur atom forms a weak hydrogen bond with His163. Notably, the methionine side chain is hydrophobic, suggesting it may maintain favorable interactions with the mutated V166, A166, or G166 residues. Based on this structural insight, we hypothesized that calpain inhibitor II would retain comparable inhibitory potency against the E166V/A/G mutants relative to wild-type M^pro^. To evaluate this, we determined the inhibition constants (*K*_*i*_) of calpain inhibitor II against WT M^pro^ as well as the E166V, E166A, and E166G variants in the FRET-based enzymatic assay. Nirmatrelvir and ibuzatrelvir were included as controls. It was found that calpain inhibitor II showed consistent inhibition against E166G/A/V with K_i_ values increasing by 1.3-, 6.1-, and 15.9-fold compared to WT, respectively (Figure 3A). In contrast, the K_i_ values of nirmatrelvir and ibuzatrelvir against E166A and E166V mutants increased by more than 100-fold and 1000-fold, respectively (Figure 3B,C). E166A and E166V mutants likewise exhibited strong resistance to the noncovalent M^pro^ inhibitor ensitrelvir, displaying over a 100-fold increase in K_i_ values (Figure 3D). These results suggest calpain inhibitor II is a promising lead for the design of M^pro^ inhibitors targeting the drug-resistant E166 mutants.

**Figure 3:**
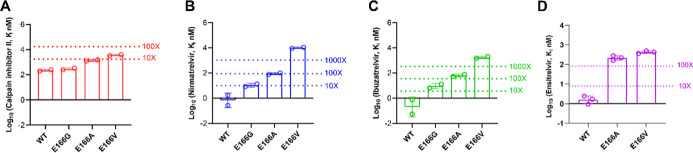
Enzymatic inhibitory activity (*K*_i_) of SARS-CoV-2 M^pro^ mutants E166G, E166A, and E166V against calpain inhibitor II (A), nirmatrelvir (B), ibuzatrelvir (C), and ensitrelvir (D). The values are the mean of two replicates.

Although calpain inhibitor II demonstrated consistent inhibition of both WT M^pro^ and the E166 mutants (E166V, E166A, and E166G), its potency requires further optimization. To address this, we designed hybrid M^pro^ inhibitors by grafting the P1 methionine side chain of calpain inhibitor II onto the nirmatrelvir scaffold. Specifically, the P1 pyrrolidone ring in nirmatrelvir was replaced with a methionine side chain (Figure 4). For the reactive warhead, we explored several electrophilic moieties, including aldehyde, bisulfite (as a prodrug of the aldehyde), ketoamide, and benzothiazolyl ketone. At the P2 position, we evaluated both cyclopentylproline and trifluoromethylproline, while for the P3 substituent, we examined valine and cyclopropylglycine. In addition to the original trifluoromethylamide N-terminal capping group, we also tested a carbamate. These substituents were selected based on their presence in advanced M^pro^ inhibitors and their potential to enhance potency and resistance profiles (Figure 1).

**Figure 4:**
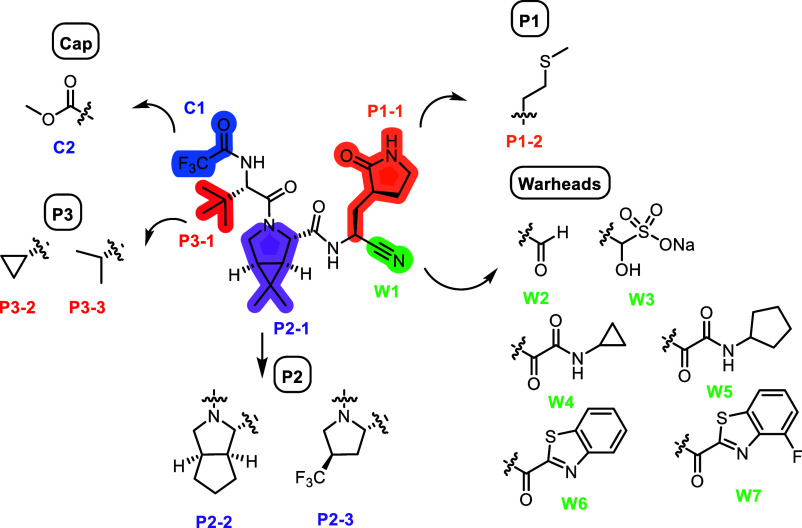
Inhibitor design targeting the SARS-CoV-2 M^pro^ E166 mutants.

The designed inhibitors were synthesized as described in Schemes S1–S2. All compounds were initially evaluated against WT M^pro^ using a FRET-based enzymatic assay. Compounds exhibiting *K*_*i*_ values ≤200 nM were subsequently tested against the E166V, E166A, and E166G mutants. To assess the breadth of activity, additional naturally occurring nirmatrelvir-resistant M^pro^ variants—S144A, S144M, N165T, H172Q, and H172Y—were also included. These mutants were selected based on their preserved enzymatic activity relative to the WT.^11^ The results are shown in Table 1.

**Table 1:**
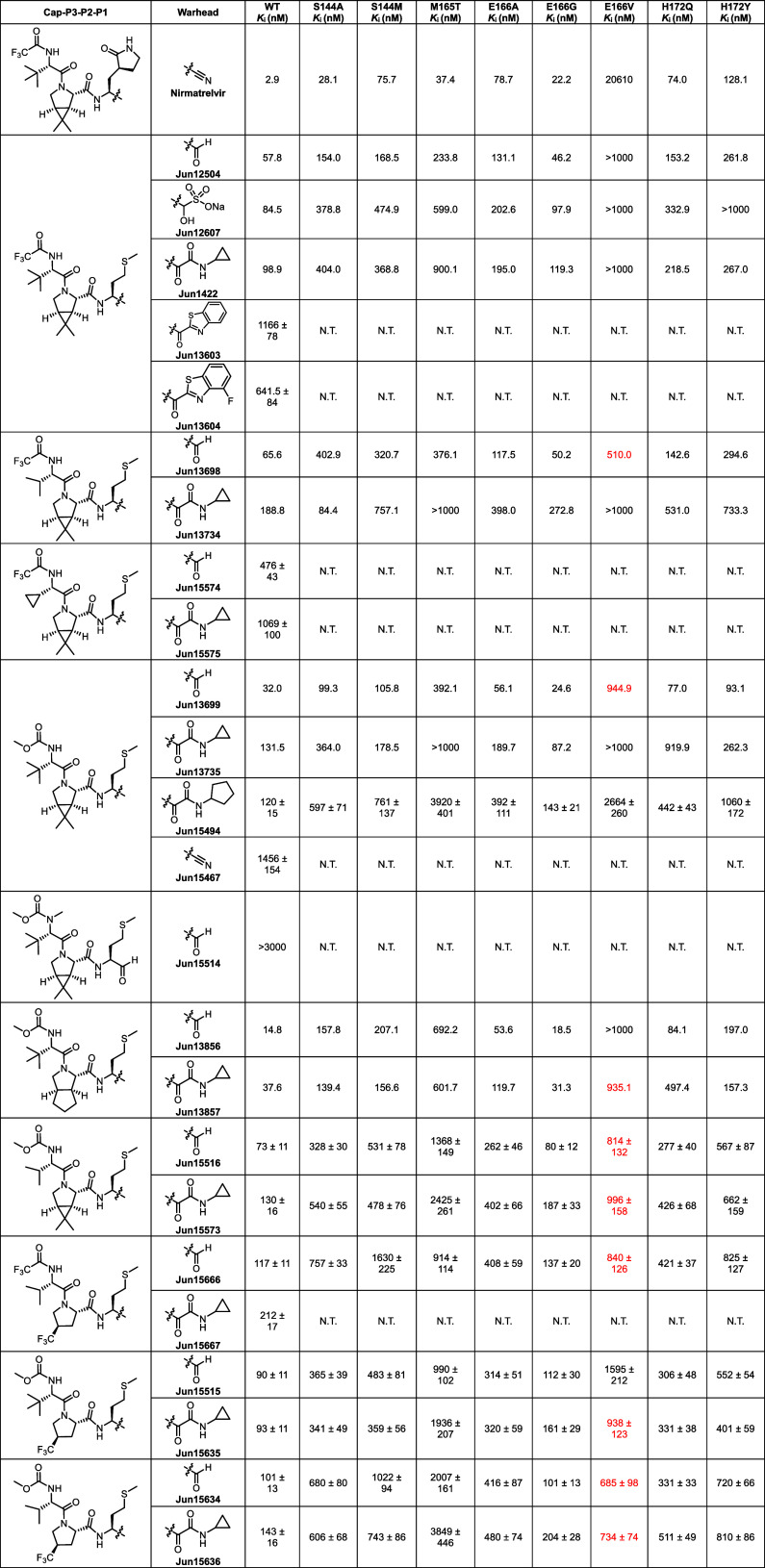
Enzymatic Inhibitory Activity of M^pro^ Inhibitors Against Drug-Resistant Mutants

As expected, the E166V mutation conferred high-level resistance to nirmatrelvir (*K*_*i*_ = 20.61 μM), representing a 7106-fold reduction in potency compared to the WT (*K*_*i*_ = 2.9 nM). Notably, several of our designed inhibitors retained substantial activity against E166V, including **Jun13698** (*K*_*i*_ = 510.0 nM), **Jun13699** (944.9 nM), **Jun13857** (935.1 nM), **Jun15516** (814.0 nM), **Jun15573** (996.0 nM), **Jun15666** (840.0 nM), **Jun15635** (938.0 nM), **Jun15634** (685.0 nM), and **Jun15636** (734.0 nM) (Table 1, highlighted in red).

Structure–activity relationship (SAR) analysis suggests that for effective inhibition of E166 mutants, the P2 position favors dimethylcyclopropylproline or trifluoromethylproline over cyclopentylproline (e.g., **Jun13699** and **Jun15515** vs **Jun13856**), while the P3 position favors valine over *tert*-butyl glycine (e.g., **Jun13698** vs **Jun12504**; **Jun15634** vs **Jun15515**). Among these, **Jun13698** emerged as a lead compound, showing potent inhibition of WT M^pro^ (*K*_*i*_ = 65.6 nM) and broad-spectrum activity against E166A (117.5 nM), E166G (50.2 nM), S144A (402.9 nM), S144M (320.7 nM), M165T (376.1 nM), H172Q (142.6 nM), and H172Y (294.6 nM) (Figure 5A,B).

**Figure 5:**
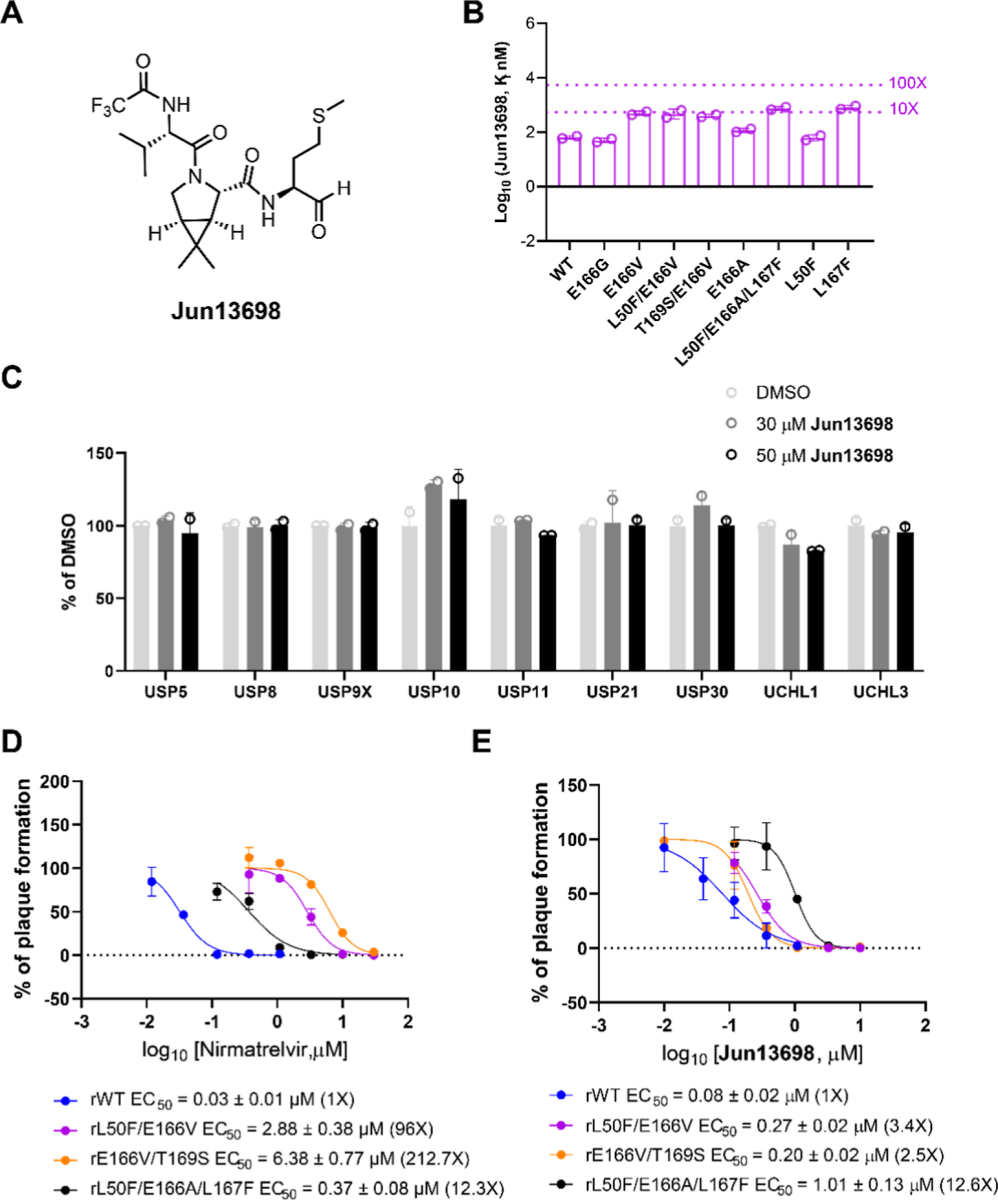
Structure and inhibition of **Jun13698** against SARS-CoV-2 M^pro^ drug-resistant mutants. (A) Chemical structure of **Jun13698**. (B) Enzymatic inhibitory activity (*K*_i_) of **Jun13698** against SARS-CoV-2 M^pro^ mutants. The values are the mean of two replicates. (C) Counter screening of **Jun13698** against host deubiquitinases. (D) Plaque assay EC_50_ plots of nirmatrelvir. (E) Plaque assay EC_50_ plots of **Jun13698**. EC_50_ values are the mean ± SD of two repeats.

In addition, **Jun13699** (*K*_*i*_ = 56.1 nM) and **Jun13856** (53.6 nM) showed greater potency than nirmatrelvir (78.7 nM) against E166A, while **Jun13856** (18.5 nM) also outperformed nirmatrelvir (22.2 nM) against E166G (Table 1). Representative kinetic curves and K_i_ fittings of **Jun13698** in inhibiting WT, E166V, and E166A are shown in Figure S1. Collectively, **Jun13698** stands out as the most promising lead, demonstrating potent inhibition of E166V/A/G variants and consistent activity across a panel of clinically relevant resistance-associated mutants, including S144A/M, M165T, and H172Q/Y.

To profile the off-target effects of **Jun13698**, we tested it against a panel of host deubiquitinases, which are also cysteine proteases, including USP5, USP8, USP9X, USP10, USP11, USP21, USP30, UCHL1, and UCHL3. **Jun13698** exhibited less than 20% inhibition at concentrations up to 50 μM across tested enzymes, indicating promising selectivity (Figure 5C).

### Jun13698 Displayed Potent Antiviral Activity Against Nirmatrelvir-Resistant SARS-CoV-2 Viruses

As predicted by the enzymatic assay results, **Jun13698** was expected to retain potent antiviral activity against SARS-CoV-2 variants harboring the E166V and E166A mutations. To evaluate this, we assessed the antiviral efficacy of **Jun13698** using plaque reduction assays against recombinant SARS-CoV-2 viruses encoding L50F/E166V, E166V/T169S, and L50F/E166A/L167F mutations.^11,36^ Notably, the E166V single mutant exhibited severely impaired replication fitness. The inclusion of L50F and T169S mutations was necessary to restore viral replication capacity. Notably, the L50F/E166V, E166V/T169S, and L50F/E166A/L167F mutations were also coselected during serial viral passage in cell culture or have also been observed in nirmatrelvir-treated patients, indicating their role in the evolution of resistance.^21,28,30,37^

Nirmatrelvir was tested in parallel as a control. The rL50F/E166V and rE166V/T169S viruses showed strong resistance to nirmatrelvir, with EC_50_ shifts of 96- and 212.7-fold relative to the WT virus, respectively (Figure 5D). The rL50F/E166A/L167F mutant conferred moderate resistance, exhibiting a 12.3-fold EC_50_ shift (0.37 μM vs 0.03 μM for wild type). In contrast, **Jun13698** maintained potent antiviral activity against both rL50F/E166V (EC_50_ = 0.27 μM) and rE166V/T169S (EC_50_ = 0.20 μM) viruses, with modest EC_50_ shifts of 3.4- and 2.5-fold compared to WT (EC_50_ = 0.08 μM) (Figure 5E). Against the rL50F/E166A/L167F mutant, **Jun13698** displayed a 12.6-fold EC_50_ shift (1.01 μM vs 0.08 μM), indicating moderate resistance similar to that observed with nirmatrelvir. **Jun13698** was not cytotoxic in Vero E6 cells with a CC_50_ value greater than 250 μM.

### X-Ray Crystal Structures

We solved the crystal structures of SARS-CoV-2 M^pro^ WT in complex with three covalent inhibitors—**Jun13698**, **Jun13699**, and **Jun13735**—at resolutions of 2.87 Å, 2.47 Å, and 2.69 Å, respectively, with two in the C2 space group, and one in I2, with one protomer per asymmetric unit (Figure 6, Table S1). All three inhibitors form covalent adducts with the catalytic C145 via thiohemiacetal (aldehyde warheads, **Jun13698** and **Jun13699**) or hemithioketal (α-ketoamide warhead, **Jun13735**) linkages. These covalent linkages are stabilized by the oxyanion hole formed by the backbone amides of G143, S144, and C145. Despite differences in warhead chemistry, all inhibitors share a conserved binding mode in the S1 and S2 subsites: the P1 methionine side chain replaces the canonical glutamine, fitting into the S1 subsite and forming a weak hydrogen bond with His163, as well as nonpolar interactions with the E166 side chain and the L141 main chain atoms. At the same time, a dimethylcyclopropylproline moiety at P2 engages the hydrophobic S2 pocket, while similar hydrogen bonds are formed between all three compounds and the backbone polar groups of S144, H164, and E166 in the S1–S3 subsites. Structural divergence is observed beyond these common features. **Jun13698** contains a trifluoroacetamide group that extends into the S3/S4 subsites and forms a hydrogen bond with the side chain of Q192, contributing to complex stabilization. This hydrogen bond resembles the interaction seen in the WT M^pro^ with the trifluoromethyl group in nirmatrelvir (PDB: 8DZ2) (Figure 6D). Meanwhile, both **Jun13699** and **Jun13735** contain a P3 *tert*-butyl glycine that contacts the E166 side chain, similar to nirmatrelvir. **Jun13735**, bearing an α-ketoamide warhead, forms a hemithioketal adduct with C145, with its hydroxyl group forming a hydrogen bond with H41. A cyclopropyl group at the warhead end projects toward the S1’ subsite and makes new nonpolar interactions with the protein; however, this addition may reduce potency due to possible suboptimal positioning, as suggested by a decrease in inhibitory activity.

**Figure 6:**
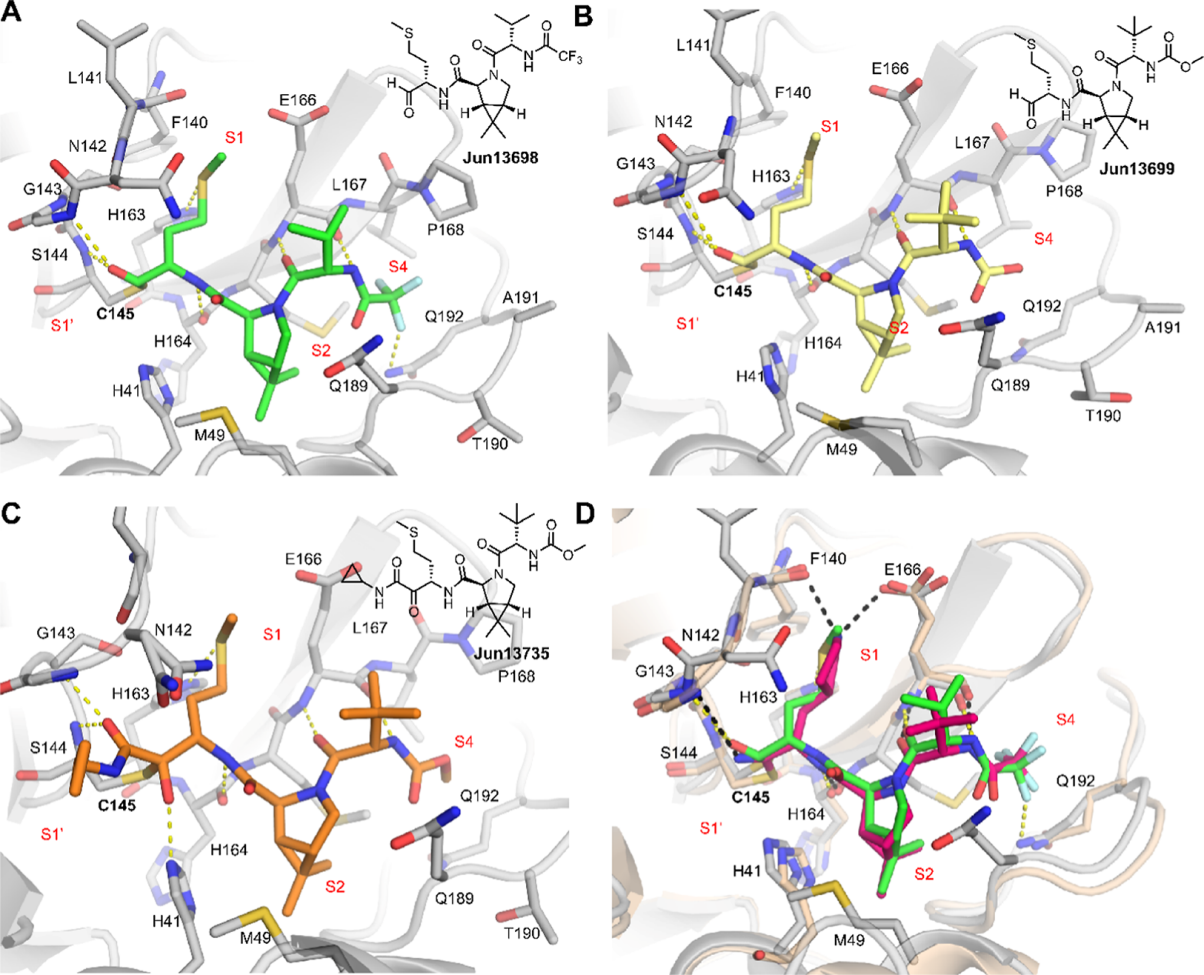
Complex structures of SARS-CoV-2 M^pro^ with inhibitors. Hydrogen bonds are shown as yellow dashed lines, and M^pro^ is shown in gray. SARS-CoV-2 M^pro^ in complex with (A) **Jun13698** (green), (B) **Jun13699** (yellow), (C) **Jun13735** (orange), and (D) the binding pose of nirmatrelvir with M^pro^ (wheat and hot pink, PBD: 8DZ2) superimposed onto the M^pro^ in complex with **Jun13698** (gray and green), with hydrogen bonds between M^pro^ and nirmatrelvir shown as black dashed lines. Substrate binding subsites are shown in red text.

To understand the structural basis of E166V-mediated resistance, we examined the crystal structures of WT and E166V mutant SARS-CoV-2 M^pro^ in complex with nirmatrelvir (PDB: 8DZ2 and 8H82, respectively) (Figure 7A). In the WT structure, the γ-lactam ring of nirmatrelvir forms key hydrogen bonds with E166 within the S1 subsite (Figure 7A, black dashed lines). These interactions are abolished in the E166 V mutant, where the substitution of glutamate with valine eliminates the hydrogen bonding potential, contributing to the observed resistance phenotype (Figure 7A, red dashed lines).

**Figure 7:**
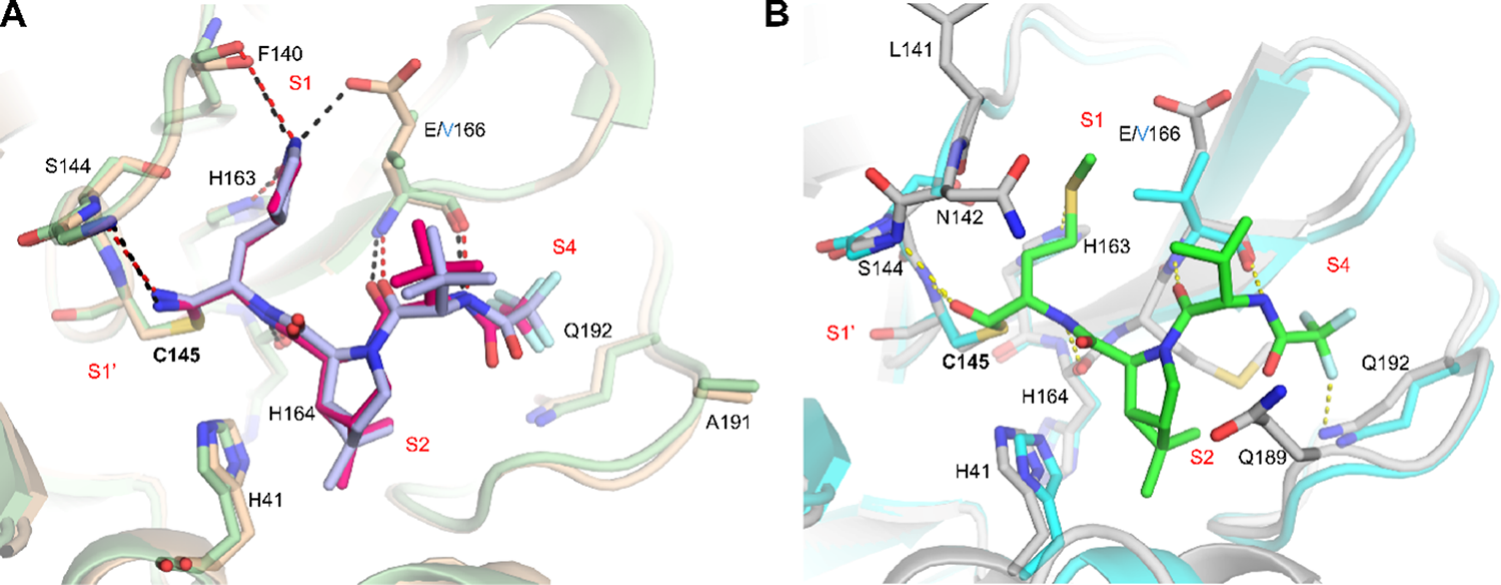
Complex structures of SARS-CoV-2 M^pro^ (A) WT with nirmatrelvir (PDB: 8DZ2, wheat and hot pink), with hydrogen bonds shown as black dashed lines, superimposed with E166V mutant in complex nirmatrelvir (PDB: 8H82, sage and purple), with hydrogen bonds shown as red dashed lines. (B) The binding pose of **Jun13698** with M^pro^ (gray and green) superimposed onto our M^pro^ E166V mutant structure (cyan), with hydrogen bonds shown as yellow dashed lines. The WT mutation is indicated with blue text, and substrate binding subsites are shown in red text.

Our attempts to obtain the complex structure of M^pro^ mutants with the new inhibitors failed, possibly, in part, due to the compounds’ solubility. However, our efforts led to several unbound mutant crystal structures, including E166V, E166V/L50F, and T169S (Figure 8). To investigate whether methionine-substituted inhibitors could overcome E166V resistance, we superimposed the crystal structure of **Jun13698**-bound WT M^pro^ onto the E166V mutant structure, with the hydrogen bond of the **Jun13698** complex shown in yellow dashed lines. This analysis revealed that the P1 methionine side chain of **Jun13698** projects into the S1 pocket, where it is poised to engage in favorable nonpolar interactions with Val166 in the mutant protease (Figure 7B). The T169S and L50F/E166V structures are also similar overall to the WT and E166V respectively, suggesting these mutants would interact with the new inhibitors in the same fashion. These findings support our design strategy to replace the canonical glutamine surrogate with a hydrophobic methionine at P1, thereby maintaining S1 subsite engagement despite the E166V mutation. Together, these structural comparisons highlight a potential path to circumvent resistance by exploiting alternative interaction networks in the mutated active site.

**Figure 8:**
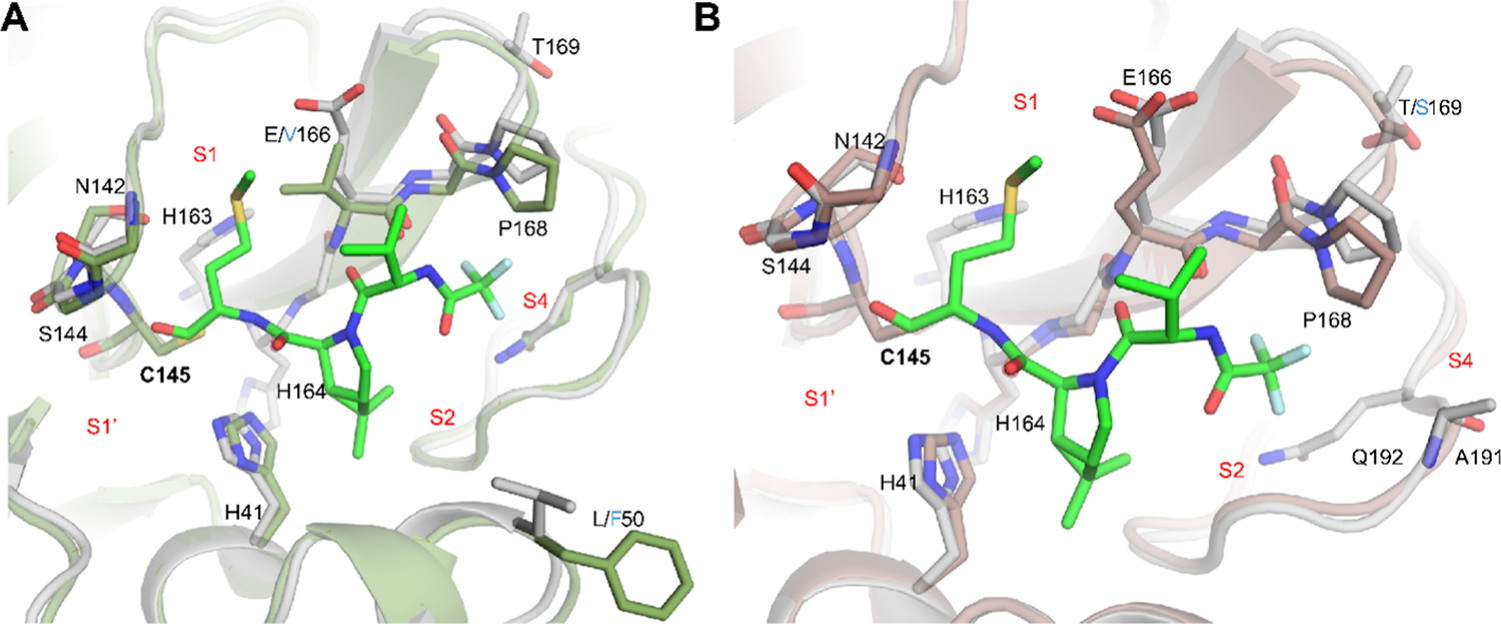
X-ray crystal structures of M^pro^ mutants. (A) E166V/L50F mutant (olive) aligned with M^pro^ in complex with **Jun13698** (gray and green). (B) T169S mutant (mauve) aligned with M^pro^ in complex with **Jun13698** (gray and green). The WT mutation is indicated with blue text, and substrate binding subsites are shown in red text.

### Molecular Dynamics (MD) Simulations of Nirmatrelvir and Jun13698 in Complex with M^pro^ WT, E166A, and E166V

To further understand the drug resistance and inhibition, we performed MD simulations of M^pro^ WT, E166V, and E166A mutants in complex with nirmatrelvir and **Jun13698**. The MD simulations showed that the covalent forms of both drugs with WT and mutant M^pro^ proteins are stable, forming similar strong hydrogen bonding interactions (data not shown).^38,39^ Therefore, we compared the docking of these two compounds before covalent bond formation to interpret the observed resistance of nirmatrelvir compared to **Jun13698** against the M^pro^ E166A and E166V mutants using MD simulations with starting structures based on the corresponding X-ray structures (PDB: 9EL4, 8H82). The simulations of nirmatrelvir revealed that the drug remains stable in the substrate-binding pocket (Figure 9A) of the M^pro^ WT, with an RMSD of the ligand at ∼2.5 Å and RMSD­(Cα) (without loops) stabilizing at ∼≤ 2 Å (Figure 9C). The protein–ligand frequency interactions plot (Figure 9B) shows that in the nirmatrelvir’s complex strong hydrogen bonding interactions are formed: (a) between the pyrrolidinone-carbonyl and trifluoroacetamide moiety and mainly the peptide bond of E166; (b) between the pyrrolidinone group and side chains of H163, and peptide bond of F140; (c) between the amide bond formed by the proline-like moiety and side chain of H164 (water bridges are also formed between this amide bond of the drug and H41); (d) between the cyano group of the drug and side chain of C145 as well as G143. Hydrophobic interactions are formed between the trifluoromethyl group and the side chains of M165, L167, P168, A191, and Q192, as well as between the dimethyl group and the side chains of H41 and D187. However, the binding of nirmatrelvir to the M^pro^ E166A and E166V mutants was unstable (Figure 9D–I). The 1 μs-MD simulations revealed that mutation E166A displaced the drug significantly from the starting structure, with an RMSD_ligand_ ∼6 Å (Figure 9D,F). Specifically, the mutation E166V produced a strong repulsion between V166 and the lactam group of the drug, causing a large displacement of nirmatrelvir away from the binding area (Figure 9G,I), as became evident after ∼450 ns MD simulations. This drug displacement causes the loss of important hydrogen bonding interactions with residues H163 and H164 (Figure 9E,H), while the lactam moiety of the drug, nirmatrelvir, is oriented toward the water phase (Figure 9D,G), losing contact with the enzyme’s binding site. In contrast, the MD simulations of **Jun13698** with M^pro^ WT, E166A and E166V mutants (Figure 9J–R) revealed that **Jun13698** remains stable in the substrate-binding pocket (Figure 9J,L,M,O,P,R) during the 500 ns–MD simulations, forming strong hydrogen bonding interactions (a) through the NH group of the valine moiety with the side chain of E166; (b) between the carbonyl groups of the proline-like peptide bond or the valine moiety and the side chains of H41, (c) water-mediated hydrogen bonds between the proline-like carbonyl group and side chain of H163 and between the carbonyl group of the trifluoroacetamide moiety and the NH group of the amide side chain of Q189. Hydrophobic interactions are formed between the trifluoromethyl group and the side chains of M165, L167, P168, A191, and Q192, between the dimethyl group and the side chains of H41 and D187, as well as between the CH_2_CH_2_SMe and the side chain of H41 (Figure 9K,N,Q).

**Figure 9:**
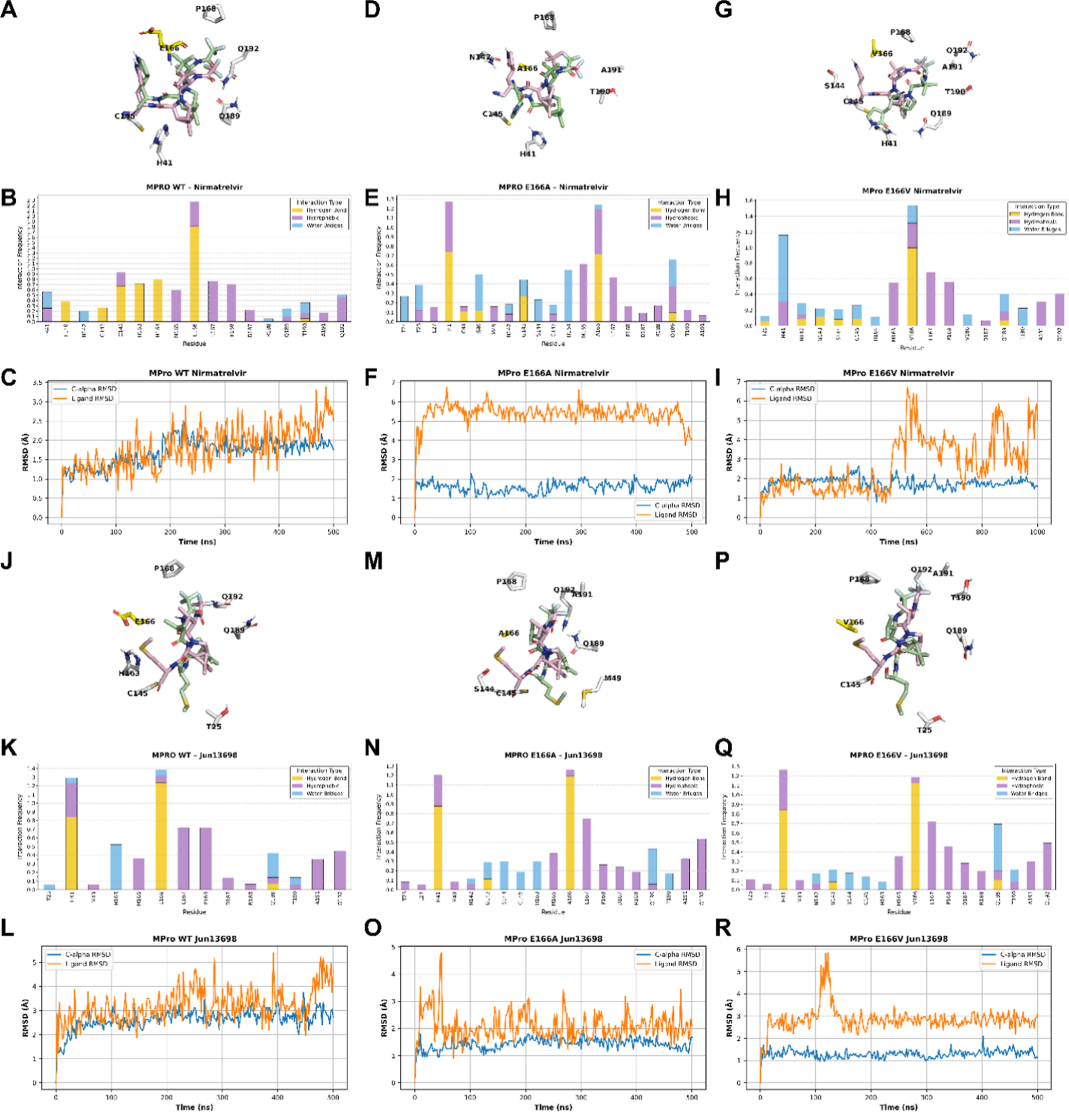
MD simulation results with Amber19sb force field (ff19sb)^40^ and the Gromacs 2023 program for the docking complex between nirmatrelvir and M^pro^ proteins, WT (panels A–C), E166A (panels D–F), and E166V (panels G–I) or **Jun13698** and M^pro^ proteins, WT (panels J–L), E166A (panels M–O), and E166V (panels P–R). For the representation of the MD simulation frames (panels A, D, G, J, M, P), pink sticks were used for ligand carbons (starting structure) or light green sticks (final snapshot); protein is illustrated using a light gray cartoon representation; amino acids within a 4 Å radius of the ligand are shown as sticks. Bars in the histograms represent protein–ligand interaction frequencies (panels B, E, H, K, N, Q with the most important lasting for more than 20% of the simulation period. Hydrophobic interactions are depicted in light purple, hydrogen bonds in yellow, and water bridges in light blue. RMSD plot of the ligand’s heavy atoms is depicted with an orange line, and the RMSD plot of the protein Cα carbons without the loops is depicted with a blue line (panels C, F, I, L, O, R). For the MD simulations, we used the X-ray structure of nirmatrelvir (PDB 8DZ2) and **Jun13698** covalently bound to M^pro^ WT (PDB 9XYM).

Collectively, the MD simulations showed that while binding of nirmatrelvir against M^pro^ WT is stable (Figure 9A–C), the binding of nirmatrelvir to the M^pro^ E166A/V mutants is unstable (Figure 9D–I). In contrast, **Jun13698** remains stable in M^pro^ WT, E166A, and E166V mutants (Figure 9J–R).

## Conclusions

Among the resistance-associated mutations identified in the SARS-CoV-2 M^pro^, the E166V-substitution stands out as one of the most prevalent and impactful escape variants. Residue E166 lies within the S1 subsite of the M^pro^ active site, where it plays a critical role in substrate recognition by forming hydrogen bonds with the conserved P1 glutamine of the viral polyprotein. Most potent M^pro^ inhibitors, including nirmatrelvir, mimic this glutamine using a pyrrolidone or piperidone ring that depends on hydrogen bonding with E166 for tight binding. Substitution of glutamate with valine at this position abolishes the polar interaction, markedly reducing the binding affinity of glutamine-mimicking inhibitors and compromising their antiviral activity (Figures 1 and 2).

To overcome this resistance mechanism, we rationally designed covalent M^pro^ inhibitors that replace the canonical glutamine surrogate—typically a five-membered pyrrolidone or six-membered piperidone—with a methionine side chain at the P1 position. From this series, the lead compound **Jun13698** emerged, maintaining potent inhibitory activity against WT M^pro^ as well as the E166A and E166V mutants. Notably, **Jun13698** demonstrated strong antiviral efficacy against recombinant SARS-CoV-2 strains carrying E166A or E166V substitutions, underscoring its potential as a candidate for further optimization. Structural analyses and molecular dynamics simulations provide mechanistic insight into the resistance conferred by E166V/A and the broad-spectrum activity of **Jun13698**. Although **Jun13698** is an aldehyde-containing compound, it was well-tolerated and did not show toxicity at up to 250 μM. Additional aldehyde-containing drugs in clinical use include streptomycin, voxelotor, and bofutrelvir.^41^ Nevertheless, given the high reactivity and off-target effects of aldehydes, future efforts will focus on designing M^pro^ inhibitors with more pharmacologically compliant reactive warheads, optimizing their pharmacokinetic properties, and advancing evaluation in animal models.

## Supplementary Material



## Notes

Experimental procedures for the M^pro^ protein expression, enzymatic assay, and antiviral assay; MD simulations; Table S1; Synthesis procedures and compound characterization; NMR spectra. (PDF)
