# Supplementary material for: Structure-Based Design of Covalent SARS-CoV‑2 Main Protease Inhibitors Targeting the Nirmatrelvir-Resistant E166 Mutants
Source: JACS Au. 2026 Jan 12;6(1):233–44. doi: 10.1021/jacsau.5c01178 (PMC12848695; doi:10.1021/jacsau.5c01178)
Supplement: Supplementary file 1 [file au5c01178_si_001.pdf]

Supporting information

Structure-based design of covalent SARS-CoV-2 main protease inhibitors targeting the nirmatrelvir-resistant E166 mutants

Zhengjun Cai,[1,#] Navita Kohaal,[2,#] Kyriakos Georgiou,[3] Xueying Liang,[4] Xiang Chi,[4] Haozhou Tan,[1] Bin Tan,[1] Kan Li,[1] Guangjin Fan,[1] George Lambrinidis,[3] Antonios Kolocouris,[3] Xufang Deng,[4,5] Yu Chen,[2,*] and Jun Wang[1,*]

[1]Department of Medicinal Chemistry, Ernest Mario School of Pharmacy, Rutgers, the State University of New Jersey, Piscataway, NJ, 08854, USA

[2]Department of Molecular Medicine, Morsani College of Medicine, University of South Florida, Tampa, FL, 33612, USA

[3]Laboratory of Medicinal Chemistry, Section of Pharmaceutical Chemistry, Department of Pharmacy, School of Health Sciences, National and Kapodistrian University of Athens, Panepistimiopolis-Zografou, Athens, 15771, Greece

[4]Department of Physiological Sciences, College of Veterinary Medicine, Oklahoma State University, Stillwater, OK, 74078, USA

[5]Oklahoma Center for Respiratory and Infectious Diseases, Oklahoma State University, Stillwater, OK, 74078, USA

[#]These authors contributed equally to this work

*Corresponding authors. Email: junwang@pharmacy.rutgers.edu (J.W.), ychen1@usf.edu (Y.C.)

Table of Contents

Figure S1 S3

1) **Experimental procedures**

SARS-CoV-2 enzymatic assay S4

Expression and purification of M^{pro} mutants S4

Counter screening against host USPs S5

Crystallization and data collection S6

Cells and viruses S7

Antiviral plaque assay S8

MD simulations S8

Synthesis of inhibitors S14

HNMR FNMR and CNMR spectra S44

2) **Supplementary Tables and Schemes**

Table S1 X-ray Data Collection and Refinement Statistics

S 1 3

Scheme S1 Synthesis of M^{pro} inhibitors S15

Scheme S2 Synthesis of M^{pro} inhibitors S17

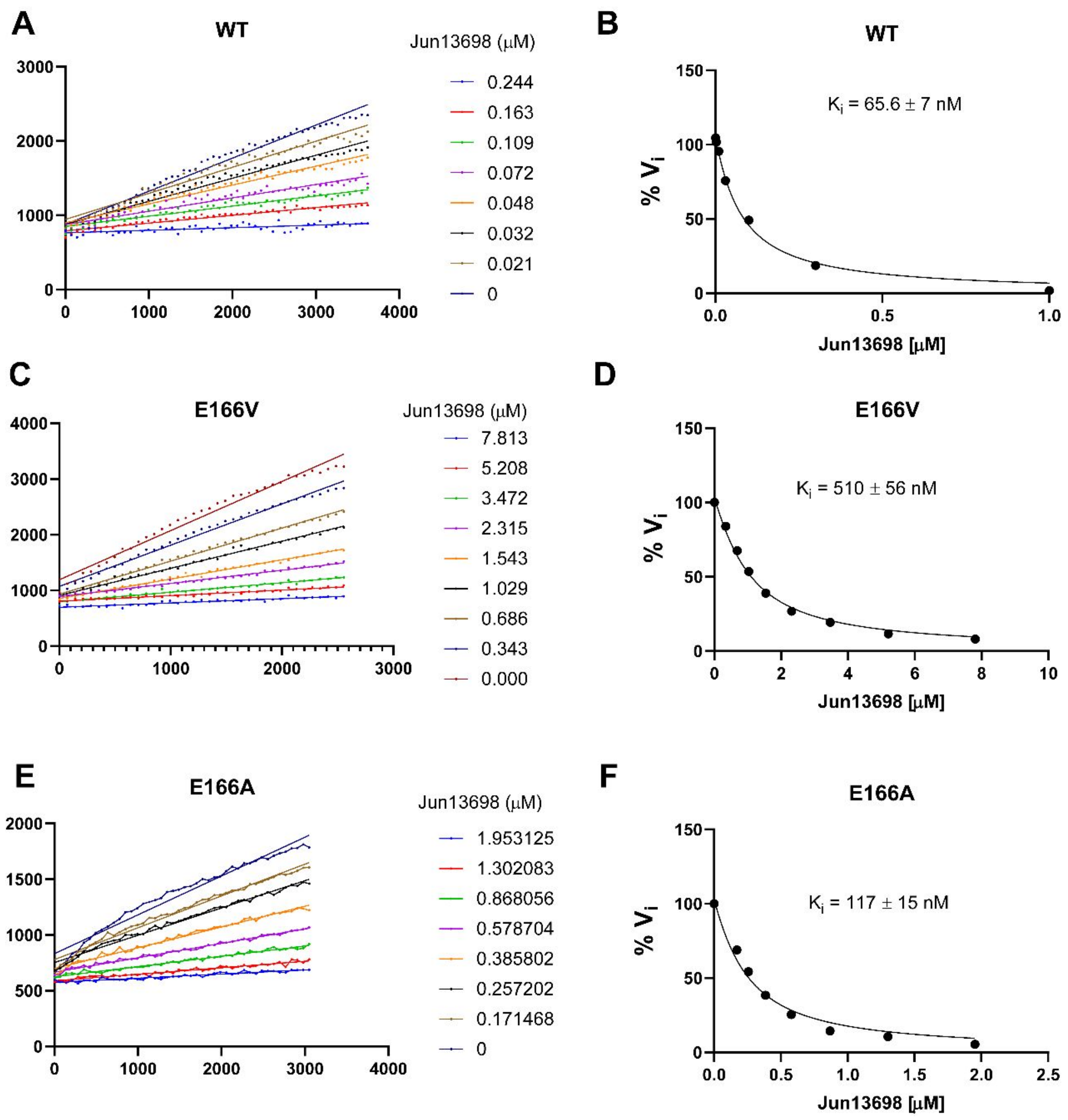

Figure S1. Representative kinetic curves and K_i fitting. Kinetic curves of Jun13698 in inhibiting WT (A), E166V (C), and E166A (E), and the corresponding K_i curve fittings for WT (B), E166V (D), and E166A (F).

1) Experimental Procedures

SARS-CoV-2 M^{pro} enzymatic assay

The enzymatic assay of SARS-CoV-2 M^{pro} was performed by mixing the various concentrations of the testing compounds with the FRET substrate (final concentration 20 μM) in the reaction buffer (HEPES 20 mM, NaCl 120 mM, EDTA 0.4 mM, DTT 4 mM, and glycerol 20%, pH 6.5), followed by reaction initiation through addition of wildtype or mutant M^{pro} at optimized concentration. No preincubation of M^{pro} with the testing compound before the reaction initiation. The fluorescent signal under excitation of 360 nm and emission of 460 nm was monitored every 71 seconds, and the first 1 hour of initial velocity was analyzed to determine the K_i. The K_i was calculated in GraphPad Prism 8.0 by plotting initial velocity against inhibitor concentrations using the Morrison equation for tight binding ($Y = V_0*(1 - ((((E_t + X + (K_i*(1 + (S/K_m)))) - (((E_t + X +(K_i*(1 + (S/K_m))))^2) - 4*E_t*X)^{0.5}))/(2*E_t)))$, X = inhibitor concentration; Y = enzyme velocity; E_t = enzyme concentration; V_0 = enzyme velocity in the absence of inhibitor). The reported values were the average of two independent replicates with 95% confidence interval calculated by SE = (upper limit − lower limit)/3.92.

Expression and purification of M^{pro} mutants

M^{pro} mutant proteins were expressed and purified as previously described.[1] Briefly, M^{pro} mutant constructs were expressed in *Escherichia coli* Rosetta (DE3) pLysS cells. Cultures were grown in 1 L LB medium containing kanamycin (50 μg/mL) and chloramphenicol (35 μg/mL) at 37 °C until an OD_{600} of 0.6–0.8 was reached. Protein expression was induced with 0.5 mM IPTG at 250 rpm, and cells were incubated at 20 °C overnight. Cells were harvested at 4000 g for 15 min at 4 °C, and the pellet was resuspended in 40 mL lysis buffer (20 mM Tris-HCl pH 8.0, 300 mM NaCl, 40 mM imidazole, 10% glycerol). Lysis was performed by ultrasonication in a 10-second on/15-second off cycle for a total of 15 min at an amplitude of 6. Lysates were clarified by centrifugation at 45,000

g for 60 min at 4 °C and loaded onto a HisTrap HP column. The column was washed with lysis buffer, and the target protein was eluted with a linear gradient of 20–500 mM imidazole. Pooled fractions were buffer-exchanged into cleavage buffer (20 mM Tris-HCl pH 8.0, 100 mM NaCl, 10% glycerol) and incubated with ULP1 protease overnight for digestion. The digest was passed through a HisTrap HP column, and the flow-through was concentrated and further purified by size-exclusion chromatography on a HiPrep 16/60 Sephacryl S-300 HR column using storage buffer (20 mM Tris-HCl pH 8.0, 200 mM NaCl, 1 mM DTT).

Counter screening against host USPs

USP5 (full-length, UniProt P45974-2), USP8 (residues 734–1110, UniProt P40818), USP9X (catalytic domain 1551–1970, PDB 5WCH), USP10 (residues 376–798, UniProt Q14694), USP11 (D1D2), USP21 (residues 209–563, UniProt Q9UK80), USP30 (Addgene 110744), UCHL1 (residues 1–223, PDB 8PW1), and UCHL3 (residues 1–230, PDB 7YV4) were codon-optimized for bacterial expression and cloned into pET28a(+) vectors containing an N-terminal hexa-histidine tag followed by a TEV protease cleavage site. Proteins were expressed in *E. coli* BL21-CodonPlus (DE3)-RIL cells (Agilent) induced with 0.5 mM isopropyl β-D-1-thiogalactopyranoside (IPTG) at 17 °C for 16 h. Cell pellets were resuspended in lysis buffer (25 mM Tris-HCl, 500 mM NaCl, 5% glycerol, 1 mM PMSF, pH 7.5) and lysed by sonication on ice (30% amplitude, 1 s on/1 s off). Cell debris was removed by centrifugation, and the clarified lysate was loaded onto a HisTrap HP column (Cytiva). The column was washed with buffer A (25 mM Tris-HCl, 500 mM NaCl, 5% glycerol, 30 mM imidazole, pH 7.5) until baseline stabilization. Proteins were eluted using buffer B (25 mM Tris-HCl, 500 mM NaCl, 5% glycerol, 300 mM imidazole, pH 7.5) with a linear gradient over 4 column volumes. Eluted fractions were analyzed by SDS–PAGE, and fractions with >95% purity were pooled and dialyzed against buffer

(25 mM Tris-HCl, 500 mM NaCl, 5% glycerol, pH 7.5) to remove imidazole.

Counter-screening was performed in 384-well plates using a reaction buffer consisting of 50 mM HEPES (pH 7.2), 0.5 mM EDTA, 1 mM DTT, and 0.1 mg/mL BSA. Test compounds were preincubated with purified DUBs for 30 min at room temperature, followed by the addition of Ub-AMC substrate (UBPBIO, M3010) to a final concentration of 0.2 μM. Fluorescence was monitored at an excitation wavelength of 340 nm and an emission wavelength of 460 nm using a Cytation 5 plate reader (BioTek), with measurements recorded every 71 seconds for 1 hour. Initial velocities were calculated by plotting the first 50 min of fluorescence signal versus time and fitting the data with linear regression in GraphPad Prism 8.0. Reported values represent the mean of two independent replicates, with error bars indicating standard deviation.

Crystallization and Data Collection

SARS-CoV-2 main protease (M^{pro}) was diluted to 2 μM in a total volume of 5 mL using buffer containing 20 mM Tris-HCl (pH 8.0) and 200 mM NaCl. Inhibitors were prepared in the same buffer and equilibrated overnight at room temperature. Equal volumes of protein and inhibitor solutions were combined the following day to yield a 10 mL mixture at 1 μM final concentration for both components, which was incubated overnight at 4 °C. The complex solution was concentrated to approximately 100 μL using Amicon centrifugal filters (10 kDa cutoff). Final protein concentrations, determined by a Nanodrop 2000c spectrophotometer, ranged between 1.2 and 3.03 mg/mL.

Crystals were grown by the hanging-drop vapor diffusion method by mixing 1.5 μL of protein–inhibitor complex with 1.5 μL of reservoir solution and equilibrating against 1 mL of reservoir at 20 °C. Crystals typically appeared within 3–5 days. The crystallization conditions were as follows: (1) for M^{pro} in complex with **Jun13698** and M^{pro} in complex with **Jun13735**, 0.07 M Bis-Tris (pH 8.0), 25% (w/v) PEG 3350; (2) for M^{pro} in complex with **Jun13699**, 0.1 M

Bis-Tris (pH 6.5), 0.1 M Li_2SO_4, 0.1 M NaCl, 20% (w/v) PEG 3350, and 10% (v/v) 1,6-hexanediol; (3) for other apo structures, (a) 25% PEG 3350, 0.1 M K/Na tartrate, and 0.005 M $MgCl_2$; (b) 0.2 M NaCl, 10% 1,6-hexanediol, and 20% PEG MME 2K; (c) 0.1 M $MgCl_2$, 20% PEG 3350.

Crystals were cryoprotected by transferring to mother liquor supplemented with 20% (v/v) glycerol before flash-cooling in liquid nitrogen. Data were collected at 100 K. Diffraction datasets were obtained as follows: (1) M^{pro} in complex with **Jun13699** at the Brookhaven National Laboratory National Synchrotron Light Source II 19-ID (NYX) beamline; (2) **Jun13698**, **Jun13735** at the Lawrence Berkeley National Laboratory Adavanced Light Source 5.0.2 beamline; (3) T169S at the Lawrence Berkeley National Laboratory Adavanced Light Source 5.0.1 beamline; (3) E166V, at the SLAC National Accelerator Laboratory 12-1 beamline; and (4) L50F/E166V at the Argonne National Laboratory Advanced Photon Source Structural Biology Center (SBC) 19-ID beamline. Data were indexed, integrated, and scaled with XDS,[2] HKL-2000,[3] or HKL-3000.[4] Molecular replacement was carried out using PHASER with SARS-CoV-2 M^{pro} (PDB: 6WTT) as the search model. Model building and refinement were performed using Coot,[5] Phenix,[6] and CCP4,[7] and structures were validated with the PDB-redo,[8] and the PDB-validation server. Figures were generated using PyMOL (Schrödinger, LLC). Complete crystallographic statistics are provided in Table S1.

Cells and viruses

Vero cells expressing human angiotensin-converting enzyme 2 (hACE2) and transmembrane protease, serine 2 (hTMPRSS2) (Vero-AT) was obtained through BEI Resources, NIAID, NIH (BEI, NR-54970). It was maintained in Dulbecco's modified Eagle medium (DMEM) (Corning, 10013CM) containing 10% heat-inactivated fetal bovine serum (FBS), 1% Pen/Strep, 1× nonessential amino acid, and 10 μg/mL puromycin (InVivogen, ant-pr-1) to maintain the

expression of hTMPRSS2 and hACE2. The following recombinant viruses were generated previously (PMID: 38547259) or in this study using the SARS-CoV-2 infectious molecular clone plasmid kit (BEI, NR-53762): recombinant wild-type SARS-CoV-2 WA1 strain (rWT), and recombinant M^{pro} mutant viruses (rL50F/E166V, rL50F/E166V, and rL50F/E166A/L167F). These viruses were titrated with Vero-AT cells and full-genome sequenced using the ARTIC method (PMID: 34578324).

Antiviral plaque assay

The antiviral plaque assay was performed similarly as we described previously (PMID: 40379662). Briefly, Vero-AT cells (3.0×10^5 cells/well) were seeded in 12-well plates a day before viral infection. The M^{pro} inhibitors dissolved in DMSO were serially diluted in DMEM with 3-fold dilutions between test concentrations. Cells in 12-well plates were infected with respective viruses for 1 h incubation at 37 °C. The inoculum was removed and 1 mL 1X DMEM-1.2% Avicel (FMC polymers) mixture containing serially-diluted compound and 2 µM CP-100356 was added to each well. After 48 h of incubation at 37 °C, the DMEM-Avicel mixture was removed and the cells were stained using 0.1% crystal violet solution. Plates were photographed and measured for the area of cells affected by infection using ImageJ.

MD simulations

Protein and Ligand Preparation

The crystal structures of the M^{pro} protein in complex with nirmatrelvir and **Jun13698** were imported within the Maestro interface (Schrödinger Release 2025-2: Maestro, Schrödinger, LLC, New York, NY, 2025) and processed using the Protein Preparation Wizard. The N- and C-termini of the protein were capped by acetyl and methylamino groups, respectively. Crystallographic water molecules beyond 5 Å from the ligand were deleted. The protonation states

were assigned via the PROPKA[9] at a pH of 7 ± 0.5. Lastly, a restraint minimization was performed using the OPLS2005 force field (Schrödinger Release 2025-2: Maestro, Schrödinger, LLC, New York, NY, 2025) to remove any potential steric clashes. Mutations such as E166A and E166V were created via manual change of an amino acid within the Maestro interface (Schrödinger Release 2025-2: Maestro, Schrödinger, LLC, New York, NY, 2025).

Using the covalently attached ligands from the crystallographic complexes as templates, we created non-covalent precursors of nirmatrelvir and **Jun13698** that match their chemical forms with optimal interactions with the protein, as in their covalently bound forms. Thus, the aldehyde warhead of **Jun13698** and the nitrile warhead of nirmatrelvir were modeled, representing the reactive moieties before covalent bond formation with C45. These ligand forms were employed in all MD simulations. Each ligand's geometry was optimized using Hartree-Fock theory with the 6-31G* basis set in the Gaussian 09 program. The electrostatic potential (ESP) charges were calculated at the same scale. RESP fitting was used to calculate atomic partial charges. The Antechamber tool from AmberTools[10] was then used to assign GAFF2[11] atom types and produce force field parameters needed for subsequent simulations. Finally, the ligand topology was incorporated into the protein system via tleap.[10]

System Setup for the MD Simulations

The complexes of M^{pro} with nirmatrelvir and **Jun13698** were used as the basis for MD simulations. Solvation was performed using Packmol-Memgen,[12] embedding each complex in a cubic TIP3P water.[13] Box with dimensions of 14 × 14 × 14 $Å^3$ and a 0.15 M NaCl concentration to mimic physiological ionic strength. System neutralization was achieved by adding either Na^+ or $Cl^−$ counterions as needed. The tleap module from AmberTools[10] was subsequently used to assign ff19sb[14] Parameters for the protein, generate

system topologies, and prepare the coordinate files for simulations using the TIP3P water model.[13]

MD Simulations Protocol

To guarantee gradual relaxation of the solvent environment surrounding the protein-ligand complex, all systems were subjected to a multi-step equilibration procedure before the production of molecular dynamics simulations. The process began with an energy minimization phase consisting of 2,500 steps of the steepest descent method, followed by 2,500 steps of conjugate gradient, applying harmonic positional restraints of 100 $kcal{\cdot}mol^{-1}{\cdot}Å^{-2}$ on all protein and ligand heavy atoms. This was followed by an NVT ensemble run of 1 ns, during which the system temperature gradually increased from 100 K to 310 K under the same positional restraints. A subsequent 1 ns NPT equilibration was conducted at 310 K and 1 bar using the same restraints. In the next step, an additional 1 ns NPT equilibration was performed with the positional restraint force constant reduced to 10 $kcal{\cdot}mol^{-1}{\cdot}Å^{-2}$. To further relax internal protein degrees of freedom, a restrained minimization was applied, consisting of 1,000 steps (500 steepest descent and 500 conjugate gradient), this time restraining only the backbone heavy atoms and ligand heavy atoms. Following this, three 1 ns NPT equilibration phases were conducted, each with positional restraint strength that decreased gradually: 10, 1, and 0.1 $kcal{\cdot}mol^{-1}{\cdot}Å^{-2}$. Lastly, a 10 ns unrestrained NPT simulation was conducted at 310 K in a $NPT\gamma$ (with $\gamma = 10$ dyn cm^{-1}) to guarantee that the system was fully relaxed before production began.

All equilibration steps used a nonbonded cutoff of 12 Å; NVT MD simulation steps were performed with a Langevin thermostat (dynamics)[15] for temperature control as implemented in Amber22 software, employing a

Langevin collision frequency of 2.0 ps and a friction coefficient constant at 1 ps^{-1}; in the NPT*γ* MD simulation steps for the pressure control, the Monte Carlo barostat[16, 17] with isotropic pressure scaling to maintain pressure at 1 bar and a 1 ps pressure relaxation time for the pressure control was used and the Langevin thermostat with a collision frequency of 2 ps^{-1} was applied. Bonds involving hydrogen atoms were constrained by the SHAKE algorithm,[18] and was used a time step of 1 fs was used for the integration of the equations of motion for the first 2 NVT and the first NPT equilibration steps, and for the rest of the NPT steps, the time step was set at 2 fs with the leapfrog Verlet integrator.[18, 19] Long-range electrostatics were calculated using the Particle Mesh Ewald (PME) method,[20] with a 1 Å grid, and short-range non-bonding interactions were truncated at 12 Å with a continuum model long-range correction applied for energy and pressure.

Particle Mesh Ewald Molecular Dynamics (pmemd) is the primary engine for running MD simulations with Amber22 software, and the energy minimization step was performed using the Central Processing Unit (CPU) of the workstations by the implementation of pmemd.[10, 20] The rest of the equilibration steps, including the unrestrained production, were run with Amber22 software on RTX 4090 GPUs in lab workstations using pmemd.CUDA algorithm.[10, 20] The pmemd.CUDA executable provides the ability to use NVIDIA GPUs to run the MD simulations.

The final frame from the equilibration phase was extracted using *cpptraj* and employed as the starting structure for the production MD simulations with the Gromacs 2023 program.[21] To enable compatibility with Gromacs, the Amber topology and coordinate files were converted to the Gromacs format using the ParmED package.[22] Each M^{pro} system was then subjected to a 500 ns to 1 μs production MD simulation under periodic boundary conditions. The Nosé–

Hoover thermostat[23] was used to maintain the temperature at 310 K, and the Parrinello–Rahman barostat[24] was applied for pressure control at 1 bar with isotropic scaling. The SHAKE algorithm[18] was employed to constrain all covalent bonds that involved hydrogen atoms to facilitate a 2-fs integration timestep. The PME method[25] was employed to address long-range electrostatics, with a real-space cutoff of 12 Å. The Lennard-Jones interactions were subjected to the same 12 Å threshold, and a force-switching function was employed to ensure that the interactions decayed smoothly to zero. To evaluate dynamic interactions and structural changes throughout the simulation, trajectory samples were recorded every 100 ps for analysis, ensuring a sufficient temporal resolution.

Analysis of the simulations

Custom Python scripts were developed with MDAnalysis,[26] NumPy,[27] Pandas, and RDKit, to conduct MD trajectory analyses. These encompassed protein–ligand contact evaluations and RMSD. Matplotlib was employed to generate all graphs, thereby guaranteeing that all systems were consistently visualized. VMD[28] was employed to visualize the MD simulation trajectories. For hydrogen bond interactions distance = 2.5 Å between donor and acceptor heavy atoms, and an angle ≥120° between donor-hydrogen-acceptor atoms and ≥ 90° between hydrogen-acceptor-bonded atoms were considered. Non-specific hydrophobic contacts were measured if the residue fell within 4.0 Å from a ligand's aromatic or aliphatic carbon. In contrast, π-π interactions were measured if two aromatic groups are stacked face-to-face or face-to-edge. Water-mediated interactions were measured if the distance between donor and acceptor atoms is 2.7 Å, the angle between donor-hydrogen-acceptor atoms is ≥ 110° and the angle between hydrogen-acceptor-bonded atoms is ≥ 80°.

Table S1. X-ray Data Collection and Refinement Statistics

**Structure (PDB ID)**	9Q7S	9XYM	9XZ6	9XYX	9XYZ	9PBC
Mutation	WT	WT	WT	T169S	E166V	L50F/E166V
Ligand	**Jun13735**	**Jun13698**	**Jun13699**	Apo	Apo	Apo
Space Group	C 1 2 1	I 1 2 1	C 1 2 1	C 1 2 1	C 1 2 1	C 1 2 1
**Cell Dimensions**						
a, *b*, *c* (Å)	114.101	45.388	113.732	114.49	112.084	113.575
	53.189	53.489	52.847	53.258	54.039	54.028
	45.609	113.716	45.427	44.704	44.694	45.059
α, β, γ (°)	90	90	90	90	90	90
	102.02	101.22	102.56	101.15	100.5	101.24
	90	90	90	90	90	90
Resolution (Å)	50-2.69	48.23-2.87	50-2.46	43.86-2.67	48.52-2.42	50-2.09
No. Reflections	7315	6138	9510	7555	10033	15645
R_{merge} (%)	6.9	15.0	16.5	8.1	20.5	9.0
$I / \sigma I$	15.68 (2.38)	6.5 (1.1)	14.59 (2.03)	12.9 (2.3)	8.7 (2.0)	23.16 (2.4)
CC1/2	0.988 (0.854)	0.993 (0.423)	0.976 (0.883)	0.995 (0.802)	0.992 (0.515)	0.992 (0.855)
Completeness (%)	98.2	98.5	99	99.1	98.9	98.42
Redundancy	4.0 (3.6)	4.1 (4.0)	6.9 (6.4)	3.3 (3.3)	6.6 (6.8)	6.3 (5.4)
**Refinement**						
Resolution (Å)	48.06-2.70	48.28-2.87	47.76-2.47	43.90-2.67	48.57-2.42	44.23-2.10
R_{work}/R_{free} (%)	20.19/24.54	19.98/25.33	20.54/26.47	21.20/26.01	20.50/27.66	20.45/25.94
**No. Heavy Atoms**						
Protein	2393	2365	2382	2409	2386	2365
Ligand/Ion	36	31	30	0	0	0
Water	18	12	32	10	22	69
***B*-Factors (Å²)**						
Protein	50.05	72.18	46.9	55.78	42.58	49.57
Ligand/Ion	54.42	87.46	62.79	0	0	0
Water	39.22	44.54	46.42	33.48	32.15	44.03
**Ramanchandran Plot**						
Favored Region (%)	95.39	92.74	97.03	94.06	96.36	96.37
Allowed Region (%)	3.95	6.6	2.64	5.28	3.31	3.3
Outlier Region (%)	0.66	0.66	0.33	0.66	0.33	0.33

* Values in parentheses represent highest resolution shells

Synthesis of inhibitors

The intermediate **5a**-**5d**, as shown in **Scheme 1a**, were prepared by an amide coupling of **1a-1b** and **2a-2c**, followed by hydrolysis of the ester and one more step of amide coupling with L-Methionine methyl ester hydrochloride. Intermediate **5e** was obtained by a N-methylation of **3a** followed by ester hydrolysis and an amide coupling reaction. Intermediate **8** was prepared in the following manner: an amide coupling between compound **6** and L-methionine methyl ester hydrochloride yielded compound **7**, which subsequently underwent Boc deprotection and amide coupling with compound **2b** to afford intermediate **8** (**Scheme 1b**).

5a-**5c**, **8** went through an ester reduction reaction to afford the corresponding alcohol **9a**-**9d**, which underwent Boc deprotection and ester aminolysis reaction to obtain **10a**-**10d**. Aldehydes **Jun12504**, **Jun13698**, **Jun15574**, and **Jun15666** were obtained via oxidation of their corresponding alcohols by using Dess–Martin periodinane (DMP) (**Scheme 1c**). A Passerini reaction between the isocyanide and various aldehydes yielded α-acyloxy amide **11a**–**11d**, and subsequent hydrolysis of the acetate groups followed by oxidation of the resulting alcohols using DMP afforded the ketoamides **Jun1422**, **Jun13734**, **Jun15575** and **Jun15667** (**Scheme 1c**).

Compounds **Jun13699**, **Jun15516**, **Jun13856**, and **Jun15514** were synthesized as follows: intermediates **5a**, **5b**, **5d**, and **5e** underwent Boc deprotection followed by substitution with methyl chloroformate to afford compounds **12a**–**12d**; Subsequent reduction of **12a**–**12d** with sodium borohydride ($NaBH_4$) yielded the corresponding alcohols **13a**–**13d**, which were then oxidized using DMP to give **Jun13699**, **Jun15516**, **Jun13856**, and **Jun15514** (**Scheme 1d**). Ketoamides **Jun13735**, **Jun15573**, **Jun13857** and **Jun15494** were prepared following the same procedure used for the synthesis of **Jun1422** (**Scheme 1d**).

Scheme S1. Synthesis of M^{pro} inhibitors.

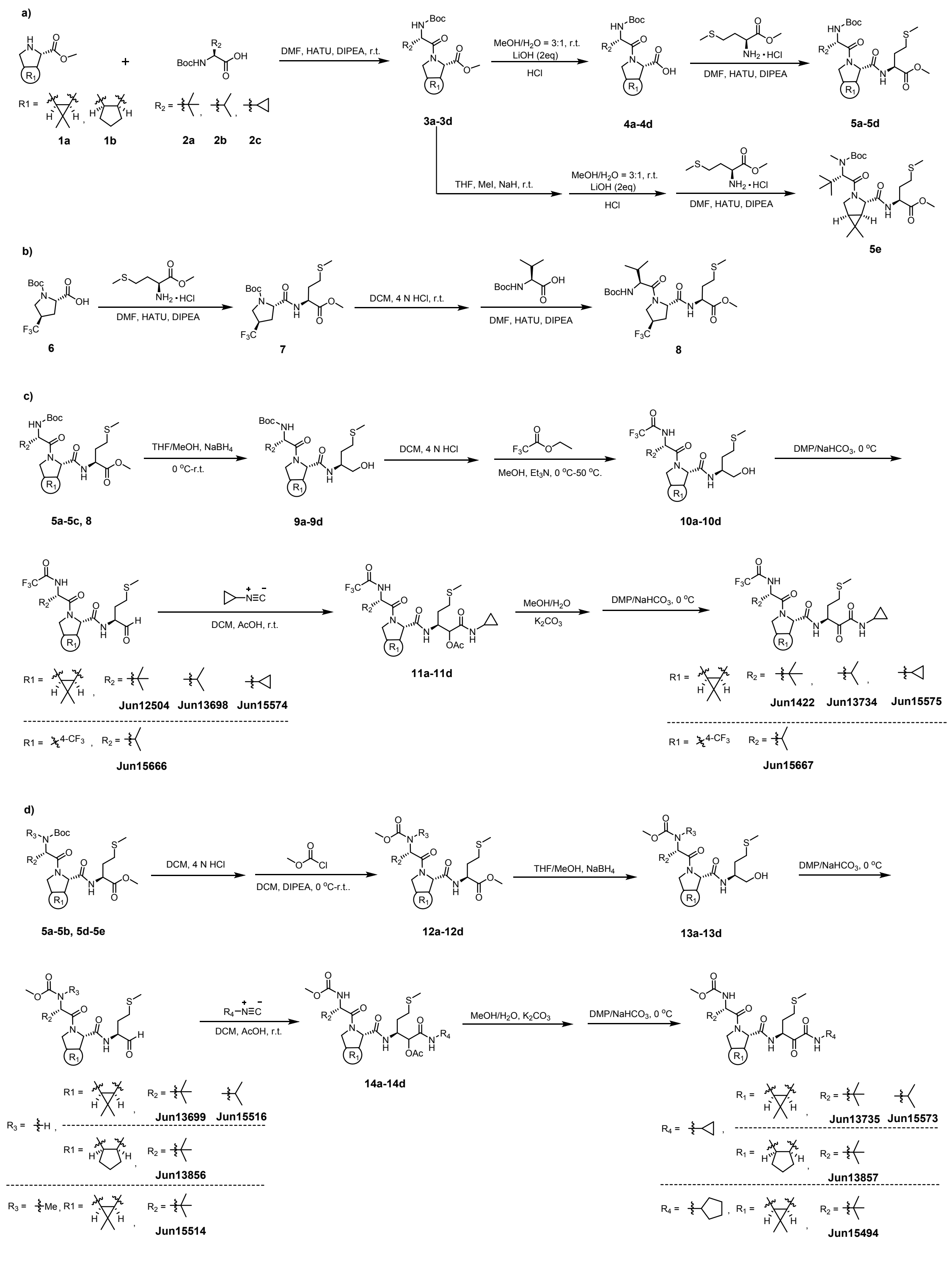

Jun13603 and **Jun13604** were synthesized as follows: Boc deprotection of compound **4a** yielded the free amine, which underwent ester aminolysis with ethyl trifluoroacetate to afford intermediate **15**; Amide coupling of **15** with L-methionine methyl ester hydrochloride gave compound **16**, which was then

hydrolyzed to get acid **17**; Subsequent amide coupling of **17** with N,O-dimethylhydroxylamine hydrochloride afforded the Weinreb amide **18**; Finally, **18** underwent a Weinreb ketone synthesis to yield **Jun13603** and **Jun13604** (**Scheme 2a**). **Jun15515**, **Jun15634**, **Jun15635**, and **Jun15636** were synthesized through the following steps: A continuously esterification reaction and Boc deprotection of compounds **2a**-**2b** yielded intermediates **19a**-**19b**; A subsequent acylation reaction followed by hydrolysis afforded intermediates **20a**-**20b**; Then **20a**-**20b** underwent amide coupling with the deprotected intermediate **21** (derived from compound **7**) to give **22a**-**22b**; Hydrolysis of the ester groups followed by oxidation of the resulting alcohols afforded the aldehydes **Jun15515** and **Jun15634**. Compounds **Jun15635** and **Jun15636** were synthesized following the same procedure as for **Jun15667** (**Scheme 2b**).

Jun15467 was afforded through an ester aminolysis reaction of **12a** and the following up dehydration reaction of **24** (**Scheme 2c**). The bisulfite compound **Jun12607** was obtained through a nucleophilic addition of **Jun12504** and $NaHSO_3$ (**Scheme 2d**).

Scheme S2. Synthesis of M^{pro} inhibitors

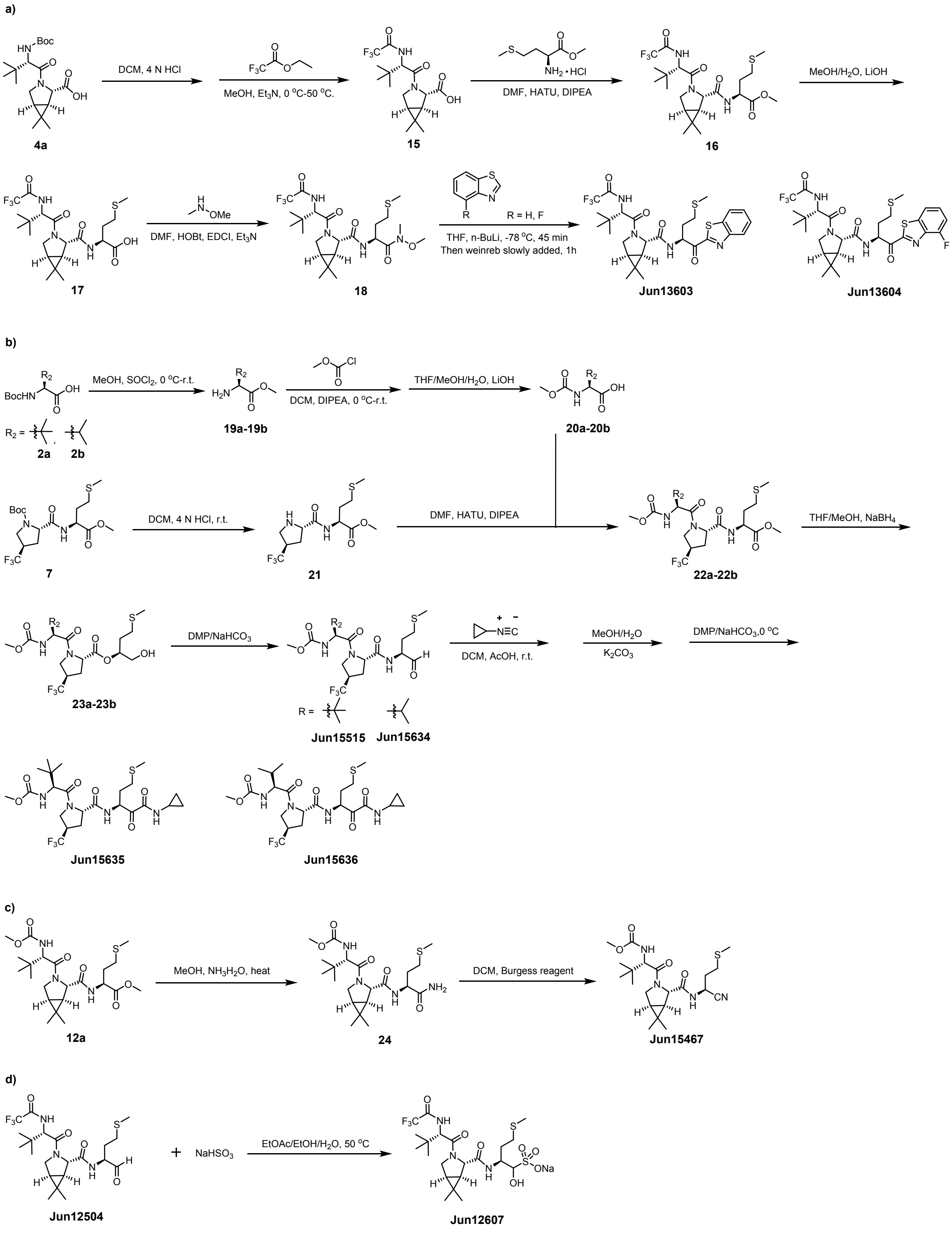

1a (1 eq) was dissolved in anhydrous DMF at 0 °C, then acid **2a** (1.05 eq) and HATU (1.1 eq) were sequentially added, the reaction mixture was stirred at 0 °C for 5 min. DIPEA (2 eq) was added drop-wise and the reaction was stirred at rt for 2 h. After finished, the mixture was added H_2O and EtOAc, organic layer was separated and water layer was extracted with EtOAc for 2 times. Combined organic phase was washed with brine (50 mL×2), dried over Na_2SO_4 and concentrated. The crude **3a** was primary purified by column chromatography

and then directly dissolved in MeOH/H_2O (3:1), LiOH (2 eq) was added and the reaction mixture was stirred at rt for 2 h. After finished, solvent was removed under vacuum, the residue was dissolved into water, 1 N HCl was added and the forming participate was filtered, washed with water and dried to get the acid **4a**. Lately **4a** (1 eq) and L-Methionine methyl ester hydrochloride (1.5 eq) went through the similar condensation reaction as mentioned in the synthesis of **3a** to get intermediate **5a**.

To a dried 100 ml round bottom flask were added NaH (2.5 eq) and anhydrous THF, the flask was degassed and added a N_2 balllon. then **3a** (dissolved in anhydrous THF, 1 eq) was slowly added to the reaction mixture and stirred at 0 ^{o}C for 30 min. Then MeI (2 eq) was slowly added and the reaction mixture stirred at rt for 24 h. When finished, the reaction was quenched with H_2O and extracted with EtOAc for 3 times, combined organic layer was washed with NaCl aqueous solution, dried with Na_2SO_4, filtered and concentrated to get the intermediate. Lately, the intermediate went through hydrolyzed and amide coupling with L-Methionine methyl ester hydrochloride to get the intermediate **5e**.

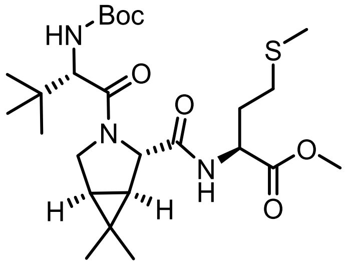

Methyl ((1R,2S,5S)-3-((S)-2-((tert-butoxycarbonyl)amino)-3,3-dimethylbut-anoyl)-6,6-dimethyl-3-azabicyclo[3.1.0]hexane-2-carbonyl)-L-methioninate (5a). Off white solid (66%). ^{1}H NMR (400 MHz, $CDCl_3$): δ 7.10 (d, J = 7.8 Hz, 1H), 5.17 (d, J = 10.2 Hz, 1H), 4.70 (td, J = 7.7, 5.3 Hz, 1H), 4.39 (s, 1H), 4.23 (d, J = 10.3 Hz, 1H), 4.01 (d, J = 10.4 Hz, 1H), 3.83 (dd, J = 10.3, 5.3 Hz, 1H), 3.76 (s, 3H), 2.52 (t, J = 7.5 Hz, 2H), 2.08 (s, 3H), 1.99–1.94 (m, 1H), 1.64 (d, J = 7.7 Hz, 1H), 1.54–1.49 (m, 1H), 1.40 (s, 9H), 1.04 (s, 3H), 0.99 (s, 9H), 0.88 (s, 3H). ^{13}C NMR (101 MHz, $CDCl_3$): δ 172.16, 171.92, 170.59, 155.94, 79.68, 60.47, 58.82, 52.46, 51.54, 48.38, 34.91, 31.89, 29.84, 29.51, 28.20, 27.60, 26.33, 26.28, 18.95, 15.35, 12.52. $C_{25}H_{43}N_3O_6S$, MS calcd for m/z $[M+H]^+$: 514.3 (calculated), 514.3 (found).

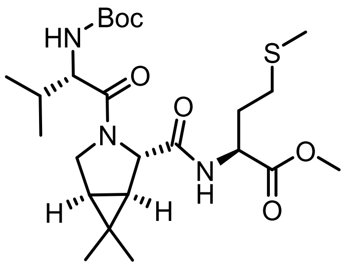

Methyl ((1R,2S,5S)-3-((tert-butoxycarbonyl)-L-valyl)-6,6-dimethyl-3-azabicyclo[3.1.0]hexane-2-carbonyl)-L-methioninate (5b). 5b was prepared by an analogous procedure to **5a**. Liquid (58%). ^{1}H NMR (400 MHz, $CDCl_3$): δ 7.18 (d, J = 8.0 Hz, 1H), 5.27 (d, J = 9.7 Hz, 1H), 4.70 (td, J = 7.8, 5.0 Hz, 1H), 4.38 (s, 1H), 4.18–4.06 (m, 1H), 3.95 (d, J = 10.4 Hz, 1H), 3.84 (dd, J = 10.3, 5.2 Hz, 1H), 3.75 (s, 3H), 2.85 (s, 1H), 2.50 (t, J = 7.5 Hz, 2H), 2.06 (s, 3H), 1.97–1.91 (m, 1H), 1.60 (d, J = 7.6 Hz, 1H), 1.52 (dd, J = 7.7, 5.2 Hz, 1H), 1.40 (s, 9H), 1.04 (s, 3H), 0.94–0.88 (m, 9H). ^{13}C NMR (101 MHz, $CDCl_3$): δ 172.26, 172.02, 170.62, 155.80, 79.56, 60.60, 57.59, 52.43, 51.52, 47.70, 31.76, 31.12, 29.80, 28.25, 27.64, 26.25, 19.25, 19.08, 18.01, 15.32, 12.67. $C_{24}H_{41}N_3O_6S$, MS calcd for m/z $[M+H]^+$: 500.3 (calculated), 500.2 (found).

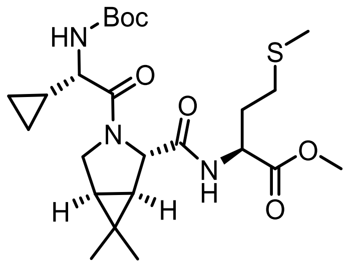

Methyl ((1R,2S,5S)-3-((S)-2-((tert-butoxycarbonyl)amino)-2-cyclopropylacetyl)-6,6-dimethyl-3-azabicyclo[3.1.0]hexane-2-carbonyl)-L-methioninate (5c). 5c was prepared by an analogous procedure to **5a**. Liquid (62%). ^{1}H NMR (400 MHz, $CDCl_3$): δ 7.41 (s, 1H), 5.38 (s, 1H), 4.76–4.47 (m, 2H), 3.85 (s, 2H), 3.72 (s, 3H), 2.53 (t, J = 7.5 Hz, 2H), 2.25–2.13 (m, 1H), 2.08 (s, 3H), 2.06–2.00 (m, 1H), 1.84–1.53 (m, 2H), 1.42 (s, 9H), 1.03 (s, 3H), 0.94 (dd, J = 8.9, 5.7 Hz, 1H), 0.86 (s, 3H). ^{13}C NMR (101 MHz, $CDCl_3$): δ 171.91, 171.23, 169.97, 155.85, 80.45, 61.80, 52.28, 51.75, 48.09, 36.40, 31.16, 30.20, 29.29, 28.30, 27.56, 26.16, 18.79, 15.39, 12.60. $C_{24}H_{39}N_3O_6S$. MS calcd for m/z $[M+H]^+$: 498.3 (calculated), 498.2 (found).

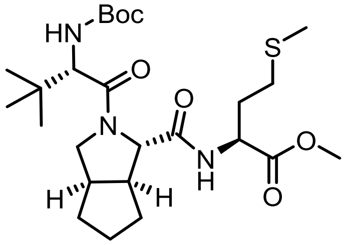

Methyl ((1S,3aR,6aS)-2-((S)-2-((tert-butoxycarbonyl)amino)-3,3-dimethyl-butanoyl)octahydrocyclopenta[c]pyrrole-1-carbonyl)-L-methioninate (5d). **5d** was prepared by an analogous procedure to **5a**. Off white solid (three steps, yield 48%). ^{1}H NMR (400 MHz, $CDCl_3$): δ 7.49 (d, J = 8.1 Hz, 1H), 5.49 (d, J = 10.0 Hz, 1H), 4.62 (td, J = 7.8, 5.2 Hz, 1H), 4.34 (d, J = 2.6 Hz, 1H), 4.28 (d, J = 10.0 Hz, 1H), 3.78 (dd, J = 10.6, 7.0 Hz, 1H), 3.68 (s, 3H), 2.75 (p, J = 3.7 Hz, 2H), 2.52–2.41 (m, 2H), 2.09–2.02 (m, 1H), 1.99 (s, 3H), 1.93–1.75 (m, 3H), 1.66–1.57 (m, 1H), 1.56–1.47 (m, 1H), 1.44–1.39 (m, 2H), 1.35 (s, 9H), 0.92 (s, 9H). ^{13}C NMR (101 MHz, $CDCl_3$): δ 172.35, 171.56, 171.50, 155.75, 79.28, 66.35, 58.14, 54.33, 52.27, 51.36, 46.15, 43.20, 35.51, 32.11, 31.81, 31.58, 29.90, 28.26, 26.34, 25.27, 15.25. $C_{25}H_{43}N_3O_6S$, MS calcd for m/z $[M+H]^+$: 514.3 (calculated), 514.2 (found).

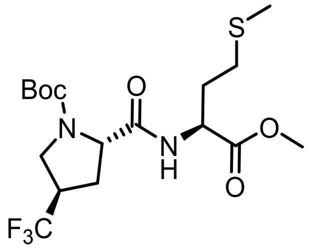

tert-butyl (2S,4R)-2-(((S)-1-methoxy-4-(methylthio)-1-oxobutan-2-yl)carba-moyl)-4-(trifluoromethyl)pyrrolidine-1-carboxylate (7). **7** was synthesized from **6** according to the procedure to **5a**. ^{1}H NMR (400 MHz, $CDCl_3$): δ 7.38 (d, J = 7.8 Hz, 1H), 4.52 (q, J = 7.1 Hz, 1H), 4.34 (t, J = 16.0 Hz, 1H), 3.62 (s, 3H), 3.50 (d, J = 9.4 Hz, 1H), 3.37 (d, J = 9.5 Hz, 1H), 3.10 (d, J = 8.5 Hz, 1H), 2.37 (q, J = 7.9, 7.1 Hz, 3H), 2.08–1.98 (m, 2H), 1.95 (s, 3H), 1.87 (dd, J = 15.7, 8.7 Hz, 1H), 1.35 (s, 9H). ^{13}C NMR (101 MHz, $CDCl_3$): δ 171.81, 170.85, 154.73, 130.49, 127.74, 124.99, 122.23, 81.01, 58.89, 52.21, 51.41, 45.82, 42.03, 41.76, 41.47, 41.17, 31.31, 29.62, 28.06, 22.43, 15.11. $C_{17}H_{27}F_3N_2O_5S$, MS calcd for m/z $[M+H]^+$: 429.1 (calculated), 429.2 (found).

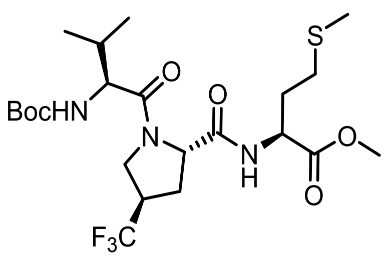

Methyl ((2S,4R)-1-((tert-butoxycarbonyl)-L-valyl)-4-(trifluoromethyl)pyrroledine-2-carbonyl)-L-methioninate (8). **7** went through the NHBoc deprotection under the 4 N HCl condition as mentioned before to get the free amine, then the amine coupled with **2b** using the similar procedure to **3a** to get the intermediate **8**. Yellow white solid (62%). ^{1}H NMR (400 MHz, $CDCl_3$): δ 7.82 (d, J = 8.2 Hz, 1H), 5.81 (d, J = 9.5 Hz, 1H), 4.80–4.66 (m, 2H), 4.24 (t, J = 8.8 Hz, 1H), 4.07–3.97 (m, 1H), 3.93 (t, J = 9.4 Hz, 1H), 3.76 (s, 3H), 3.42 (q, J = 8.2 Hz, 1H), 2.52 (q, J = 7.1, 6.7 Hz, 2H), 2.44–2.35 (m, 1H), 2.26–2.17 (m, 1H), 2.06 (s, 3H), 1.96 (p, J = 7.3 Hz, 2H), 1.44 (s, 9H), 1.28 (d, J = 10.2 Hz, 1H), 0.94 (t, J = 7.4 Hz, 6H). ^{13}C NMR (101 MHz, $CDCl_3$): δ 172.25, 172.03, 170.68, 155.63, 127.56, 124.80, 79.46, 59.11, 56.88, 52.24, 51.25, 46.35, 42.30, 42.01, 31.47, 31.21, 29.64, 28.12, 22.45, 19.08, 17.60, 14.99. $C_{22}H_{36}F_3N_3O_6S$, MS calcd for m/z $[M+H]^+$: 528.2 (calculated), 528.2 (found).

Ether **5a** (1 eq) was dissolved in anhydrous THF/MeOH (15:1, v/v), $NaBH_4$ (6 eq) was added slowly at 0 °C and the reaction mixture was stirred at rt for 2 h. After finished, saturated NH_4Cl solution was added to quench the reaction. Subsequently, the mixture was extracted with EtOAc for 3 times, combined organic phase was washed with brine (20 ml × 2), dried over Na_2SO_4 and concentrated. The reaction was transformed quantitative and the product alcohol **9a** was used directly for next step without further purification.

HCl (8 eq, 4 N in dioxane) was added to a solution of **9a** in anhydrous DCM, the reaction was stirred at rt for 2 h. After finished, remove the all solvent in vacuo. The residue was washed with DCM for 3 times, and was dried again by rotary evaporator to get the intermediate amine, which was used directly for the next step without purification. Intermediate amine (1 eq) and triethylamine (4 eq) was dissolved in anhydrous MeOH, then the reaction mixture was cooled at 0 ^{o}C, ethyl trifluoroacetate (1.8 eq) was slowly added and the reaction was heated at 50 ^{o}C for overnight. After finished, the reaction mixture was concentrated under vacuum, H_2O and EtOAc was added, organic layer was separated and water layer was extracted with EtOAc for two times. Combined organic phase was washed with brine, dried over Na_2SO_4 and concentrated. The crude product was purified by column chromatography (DCM:MeOH, 20: 1 v/v) to afford intermediate **10a**.

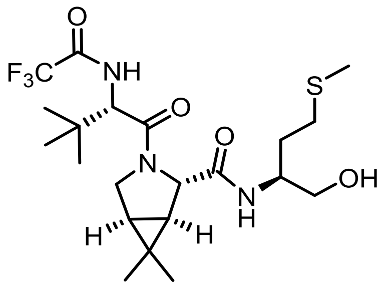

(1R,2S,5S)-3-((S)-3,3-dimethyl-2-(2,2,2-trifluoroacetamido)butanoyl)-N-((S)-1-hydroxy-4-(methylthio)butan-2-yl)-6,6-dimethyl-3-azabicyclo [3.1.0]hexane-2-carboxamide (10a). Off white solid (45%). ^{1}H NMR (400 MHz, $CDCl_3$): δ 7.28–7.21 (m, 1H), 7.05 (d, J = 8.5 Hz, 1H), 4.52 (d, J = 9.4 Hz, 1H), 4.29 (s, 1H), 3.99 (dt, J = 8.6, 4.4 Hz, 1H), 3.90 (dd, J = 10.4, 5.3 Hz, 1H), 3.73 (d, J = 5.3 Hz, 1H), 3.62–3.52 (m, 2H), 2.46 (dt, J = 15.9, 7.6 Hz, 2H), 2.00 (s, 3H), 1.74 (td, J = 7.6, 2.9 Hz, 1H), 1.51 (dd, J = 7.8, 5.2 Hz, 1H), 1.45 (d, J = 7.6 Hz, 1H), 1.20 (q, J = 3.8, 2.4 Hz, 1H), 0.98 (s, 3H), 0.96 (s, 9H), 0.78 (s, 3H). ^{13}C NMR (101 MHz, $CDCl_3$): δ 171.00, 169.10, 157.49, 157.22, 156.85, 156.47, 120.05, 117.20, 114.34, 111.48, 64.20, 61.20, 58.02, 50.87, 48.70, 35.83, 30.50, 30.38, 27.67, 26.31, 26.07, 19.06, 15.26, 12.54. $C_{21}H_{34}F_3N_3O_4S$, MS calcd for m/z $[M+H]^+$: 482.2 (calculated), 482.2 (found).

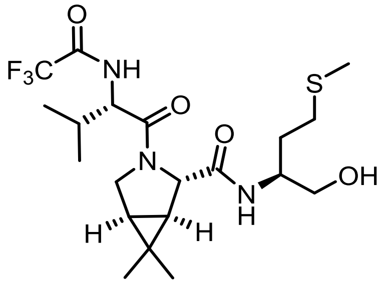

(1R,2S,5S)-N-((S)-1-hydroxy-4-(methylthio)butan-2-yl)-6,6-dimethyl-3-((2,2,2-trifluoroacetyl)-L-valyl)-3-azabicyclo[3.1.0]hexane-2-carboxamide (10b). 10b was prepared by an analogous procedure to **10a**. Off white solid (73%). ^{1}H NMR (400 MHz, $CDCl_3$): δ 7.53 (d, J = 8.6 Hz, 1H), 7.04 (d, J = 8.4 Hz, 1H), 4.38 (t, J = 8.1 Hz, 1H), 4.25 (s, 1H), 3.98 (ddt, J = 11.3, 7.6, 3.9 Hz, 1H), 3.87 (dd, J = 10.3, 5.1 Hz, 1H), 3.80 (d, J = 10.3 Hz, 1H), 3.63–3.59 (m, 2H), 3.05 (s, 1H), 2.56–2.39 (m, 2H), 2.01 (s, 3H), 1.76 (q, J = 7.2 Hz, 2H), 1.52 (dd, J = 7.7, 4.9 Hz, 1H), 1.46 (d, J = 7.6 Hz, 1H), 0.98 (s, 3H), 0.93 (d, J = 6.7 Hz, 3H), 0.85 (d, J = 6.7 Hz, 3H), 0.81 (s, 3H). ^{13}C NMR (101 MHz, $CDCl_3$): δ 170.84, 169.74, 158.04, 157.40, 157.03, 156.66, 117.26, 114.40, 64.36, 61.36, 56.80, 51.17, 47.97, 31.56, 31.05, 30.43, 27.69, 26.14, 19.24, 19.20, 17.68, 12.64. $C_{20}H_{32}F_3N_3O_4S$, MS calcd for m/z $[M+H]^+$: 468.2 (calculated), 468.2 (found).

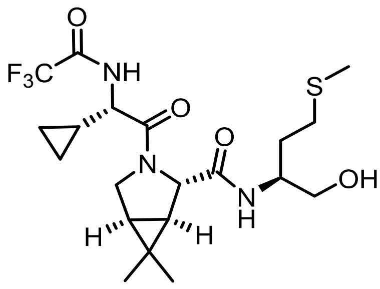

(1R,2S,5S)-3-((S)-2-cyclopropyl-2-(2,2,2-trifluoroacetamido)acetyl)-N-((S)-1-hydroxy-4-(methylthio)butan-2-yl)-6,6-dimethyl-3-azabicyclo[3.1.0]hexane-2-carboxamide (10c). 10c was prepared by an analogous procedure to **10a**. Off white solid (76%). ^{1}H NMR (400 MHz, $CDCl_3$): δ 9.54 (s, 1H), 6.85 (d, J = 8.9 Hz, 1H), 4.45 (s, 1H), 4.10 (ddt, J = 10.5, 7.0, 3.5 Hz, 1H), 3.77 (d, J = 11.0 Hz, 1H), 3.66 (ddd, J = 20.2, 15.1, 10.7 Hz, 4H), 2.51 (q, J = 6.8 Hz, 2H), 2.10 (s, 3H), 1.91 (dq, J = 9.7, 4.9, 3.7 Hz, 1H), 1.83 (q, J = 7.3 Hz, 2H), 1.60 (d, J = 7.5 Hz, 2H), 1.48 (dd, J = 7.5, 5.2 Hz, 1H), 1.06 (s, 3H), 0.94–0.86 (m, 1H), 0.85 (s, 3H). ^{13}C NMR (101 MHz, $CDCl_3$): δ 171.57, 168.85, 159.66, 159.29, 119.93, 117.08, 114.22, 111.37, 64.51, 62.87, 51.59, 48.34, 37.10, 30.66, 30.04, 29.77, 27.42, 25.89, 19.09, 15.39, 14.68, 13.51, 12.35. $C_{20}H_{30}F_3N_3O_4S$. MS calcd for m/z $[M+H]^+$: 466.2 (calculated), 466.2 (found).

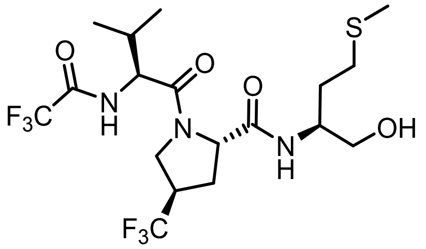

(2S,4R)-N-((S)-1-hydroxy-4-(methylthio)butan-2-yl)-1-((2,2,2-trifluoroacetyl)-L-valyl)-4-(trifluoromethyl)pyrrolidine-2-carboxamide (10d). 10d was synthesized from **8** using the similar procedure to **10a**. Off white solid (48%). ^{1}H NMR (400 MHz, $CDCl_3$): δ 7.92 (d, J = 8.5 Hz, 1H), 7.46 (d, J = 8.6 Hz, 1H), 4.61 (dd, J = 8.4, 3.0 Hz, 1H), 4.50 (t, J = 8.1 Hz, 1H), 4.09–3.91 (m, 3H), 3.66 (s, 3H), 3.48 (q, J = 8.1 Hz, 1H), 2.57–2.45 (m, 2H), 2.37 (ddd, J = 13.3, 7.4, 2.9 Hz, 1H), 2.22–2.16 (m, 1H), 2.14–2.09 (m, 1H), 2.07 (s, 3H), 1.84–1.74 (m, 2H), 0.99 (dd, J = 20.8, 6.8 Hz, 6H). ^{13}C NMR (101 MHz, $CDCl_3$): δ 170.44, 170.39, 157.44, 157.07, 127.44, 124.69, 117.22, 114.36, 64.02, 59.82, 56.40, 50.81, 46.71, 42.45, 42.16, 31.19, 30.48, 30.42, 27.69, 18.92, 17.74, 15.16. $C_{18}H_{27}F_6N_3O_4S$, MS calcd for m/z $[M+H]^+$: 496.2 (calculated), 496.2 (found).

Alcohol **10a** (1 eq) was dissolved in DCM, Dess−Martin periodinane (1.1 eq) and $NaHCO_3$ (1.1 eq) were slowly added at 0 °C and the resulting mixture was

stirred at 0 °C for 0.5 h. Then the mixture was quenched with solution of $NaHCO_3$ and $Na_2S_2O_3$, DCM was added to extract the mixture, and the organic phase was washed with NaCl aqueous solution for 2 times, dried over Na_2SO_4, filtered and concentrated. The residue was purified by Prep-HPLC to give the final product **Jun12504**.

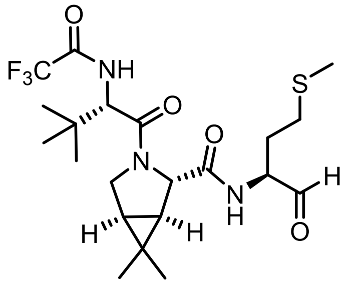

(1R,2S,5S)-3-((S)-3,3-dimethyl-2-(2,2,2-trifluoroacetamido)butanoyl)-6,6-dimethyl-N-((S)-4-(methylthio)-1-oxobutan-2-yl)-3-azabicyclo[3.1.0]hexane-2-carboxamide (Jun12504). Off white solid (60%). ^{1}H NMR (400 MHz, $CDCl_3$): δ 9.58 (s, 1H), 7.24–7.18 (m, 1H), 7.07 (d, J = 9.5 Hz, 1H), 4.58 (td, J = 7.4, 5.0 Hz, 1H), 4.51 (d, J = 9.4 Hz, 1H), 3.90 (dd, J = 10.3, 5.1 Hz, 1H), 3.80–3.73 (m, 1H), 3.43–3.30 (m, 1H), 2.73 (s, 3H), 2.50 (h, J = 6.6 Hz, 2H), 1.87 (dt, J = 14.3, 7.1 Hz, 1H), 1.55–1.42 (m, 2H), 1.25–1.16 (m, 1H), 0.99 (s, 3H), 0.96 (s, 9H), 0.80 (s, 3H). ^{13}C NMR (101 MHz, $CDCl_3$): δ 198.44, 171.01, 169.06, 157.17, 156.80, 117.21, 114.36, 60.89, 57.98, 57.91, 48.60, 38.58, 35.92, 30.31, 29.78, 28.47, 27.73, 26.33, 26.10, 19.21, 15.24, 12.59. ^{19}F NMR (376 MHz, $CDCl_3$): δ -75.52. $C_{21}H_{32}F_3N_3O_4S$, HRMS calcd for m/z $[M+H]^+$: 480.2144 (calculated), 480.2152 (found).

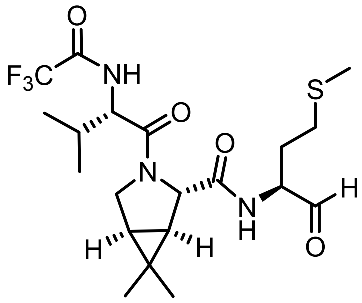

(1R,2S,5S)-6,6-dimethyl-N-((S)-4-(methylthio)-1-oxobutan-2-yl)-3-((2,2,2-trifluoroacetyl)-L-valyl)-3-azabicyclo[3.1.0]hexane-2-carboxamide (Jun13698). Aldehyde **Jun13698** was prepared by an analogous procedure to **Jun12504**. Off white solid (64%). ^{1}H NMR (400 MHz, DMSO-d_6): δ 9.75 (d, J = 8.0 Hz, 1H), 9.46 (s, 1H), 8.52 (d, J = 7.6 Hz, 1H), 4.33–4.21 (m, 2H), 4.13 (t, J = 9.5 Hz, 1H), 3.83 (d, J = 6.4 Hz, 3H), 2.56 (dd, J = 9.6, 5.0 Hz, 1H), 2.14–2.04 (m, 1H), 2.03 (s, 3H), 1.75 (ddd, J = 14.3, 10.1, 6.1 Hz, 1H), 1.52 (dt, J =

13.6, 7.2 Hz, 1H), 1.40 (dd, J = 7.6, 2.6 Hz, 1H), 1.09–1.00 (s, 3H), 0.95–0.83 (m, 9H). ^{13}C NMR (101 MHz, DMSO-d_6): δ 201.38, 171.80, 168.69, 157.12, 156.76, 117.74, 114.87, 60.46, 57.83, 57.64, 47.57, 31.37, 29.75, 28.34, 27.56, 26.40, 19.25, 18.95, 15.00, 12.87. ^{19}F NMR (376 MHz, $CDCl_3$): δ -75.51. $C_{20}H_{30}F_3N_3O_4S$, HRMS calcd for m/z $[M+H]^+$: 466.1987 (calculated), 466.1992 (found).

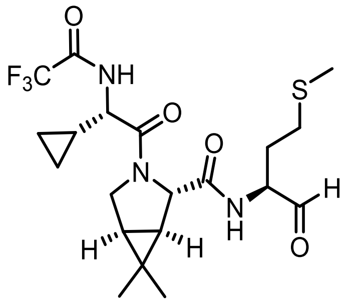

(1R,2S,5S)-3-((S)-2-cyclopropyl-2-(2,2,2-trifluoroacetamido)acetyl)-6,6-dimethyl-N-((S)-4-(methylthio)-1-oxobutan-2-yl)-3-azabicyclo[3.1.0]hexane-2-carboxamide (Jun15574). Aldehyde **Jun15574** was prepared by an analogous procedure to **Jun12504**. Off white solid (66%). ^1H NMR (400 MHz, $CDCl_3$): δ 9.58 (s, 1H), 8.11 (s, 1H), 7.36 (d, J = 7.3 Hz, 1H), 4.63 (s, 1H), 4.47–4.38 (m, 1H), 3.81 (d, J = 10.6 Hz, 1H), 3.71 (dd, J = 10.6, 5.3 Hz, 1H), 2.60 (dt, J = 13.7, 6.8 Hz, 1H), 2.51 (dt, J = 13.8, 7.2 Hz, 1H), 2.20 (dq, J = 9.9, 3.8, 2.6 Hz, 1H), 2.08 (s, 3H), 2.05–1.98 (m, 1H), 1.93 (ddd, J = 10.5, 7.7, 5.2 Hz, 1H), 1.67 (d, J = 7.5 Hz, 1H), 1.48 (dd, J = 7.6, 5.2 Hz, 1H), 1.39 (ddd, J = 10.4, 7.8, 5.6 Hz, 1H), 1.20–1.12 (m, 1H), 1.07 (s, 3H), 1.00 (ddd, J = 10.6, 7.7, 5.3 Hz, 1H), 0.86 (s, 3H). ^{13}C NMR (101 MHz, $CDCl_3$): δ 199.57, 171.90, 168.28, 158.89, 158.52, 116.95, 114.09, 61.97, 58.07, 48.38, 36.74, 29.91, 29.49, 27.77, 27.37, 25.98, 19.09, 15.16, 14.19, 12.41. ^{19}F NMR (376 MHz, DMSO-d_6): δ -73.99. $C_{20}H_{28}F_3N_3O_4S$. HRMS calcd for m/z $[M+H]^+$: 464.1834 (calculated), 464.1857 (found).

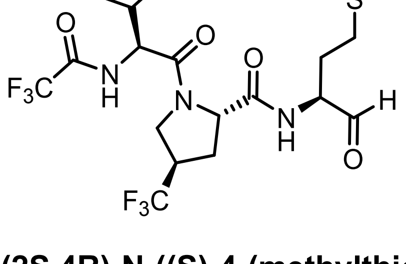

(2S,4R)-N-((S)-4-(methylthio)-1-oxobutan-2-yl)-1-((2,2,2-trifluoroacetyl)-L-valyl)-4-(trifluoromethyl)pyrrolidine-2-carboxamide (Jun15666). Jun15666 was prepared by an analogous procedure to **Jun12504**. Off white solid (59%).

^1H NMR (400 MHz, DMSO-d_6): δ 9.84 (d, J = 7.9 Hz, 1H), 9.46 (s, 1H), 8.55 (d, J = 7.4 Hz, 1H), 4.55 (dd, J = 8.5, 5.2 Hz, 1H), 4.29 (dt, J = 14.2, 7.3 Hz, 2H), 3.99 (t, J = 6.5 Hz, 3H), 3.41 (q, J = 8.0 Hz, 1H), 2.57 (ddd, J = 14.0, 8.6, 5.4 Hz, 1H), 2.33 (dt, J = 14.4, 7.7 Hz, 1H), 2.23–2.08 (m, 3H), 2.04 (s, 3H), 1.76 (ddt, J = 13.4, 8.7, 4.4 Hz, 1H), 0.94 (d, J = 6.4 Hz, 3H), 0.91 (d, J = 6.8 Hz, 3H). ^{13}C NMR (101 MHz, DMSO-d_6): δ 201.24, 171.79, 169.09, 157.15, 156.78, 117.76, 114.90, 59.09, 57.73, 57.43, 46.82, 41.69, 41.41, 29.93, 29.74, 29.12, 28.24, 19.07, 18.79, 14.96. ^{19}F NMR (376 MHz, DMSO-d_6): δ -70.25, -73.59. $C_{18}H_{25}F_6N_3O_4S$, HRMS calcd for m/z $[M+H]^+$: 494.1548 (calculated), 494.1556 (found).

Aldehyde **Jun12504** (1 eq) was dissolved in anhydrous DCM (15 mL), acetic acid (1.2 eq) and isocyanide (1.1 eq) were added to the mixture, the reaction was stirred at rt for 24 h under N_2 atmosphere. Then, the solvent was removed in vacuo and crude product was purified by chromatography on silica gel (DCM:MeOH, 20: 1 v/v) to give compound **11a**. **11a** (1 eq) was dissolved in MeOH (10 mL) and H_2O (2 mL), K_2CO_3 (2 eq) was added and the mixture was stirred at rt for 1 h. After finished, the mixture was added 1 N HCl to adjusted to neutral. EtOAc was added, organic layer was separated and water layer was extracted with EtOAc for two times. Combined organic phase was washed with NaCl, dried over Na_2SO_4 and concentrated to get the corresponding alcohol. Alcohol (1 eq) was dissolved in anhydrous DCM, Dess-Martin periodinane (1.1 eq) and $NaHCO_3$ (1.1eq) were slowly added at 0 °C and the reaction was stirred at 0 °C for 0.5 h. Then the mixture was quenched with solution of $NaHCO_3$ and $Na_2S_2O_3$, DCM was added to extract the mixture, and the organic phase was washed with NaCl aqueous solution for 2 times, dried over Na_2SO_4, filtered and concentrated. The residue was purified by Prep-HPLC to give the final product **Jun1422**.

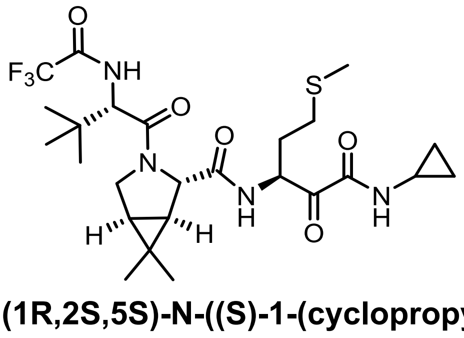

(1R,2S,5S)-N-((S)-1-(cyclopropylamino)-5-(methylthio)-1,2-dioxopentan-3-yl)-3-((S)-3,3-dimethyl-2-(2,2,2-trifluoroacetamido)butanoyl)-6,6-dimethyl-3-azabicyclo[3.1.0]hexane-2-carboxamide (Jun1422). Off white solid

(67%). ^{1}H NMR (400 MHz, $CDCl_3$): δ 7.09 (t, J = 9.1 Hz, 2H), 7.00 (d, J = 3.9 Hz, 1H), 5.45–5.34 (m, 1H), 4.57 (d, J = 9.3 Hz, 1H), 4.37 (s, 1H), 3.95 (dd, J = 10.4, 4.9 Hz, 1H), 3.86 (d, J = 10.5 Hz, 1H), 2.79 (tq, J = 7.7, 3.9 Hz, 1H), 2.55 (t, J = 6.8 Hz, 2H), 2.43–2.29 (m, 1H), 2.14 (dd, J = 14.3, 7.1 Hz, 1H), 2.05 (s, 3H), 1.63–1.49 (m, 2H), 1.06 (s, 3H), 1.04 (s, 9H), 0.87 (s, 3H), 0.86 (s, 2H), 0.66–0.55 (m, 2H). ^{13}C NMR (101 MHz, $CDCl_3$): δ 194.88, 170.51, 169.45, 160.45, 157.35, 156.98, 117.16, 114.30, 61.12, 57.99, 54.49, 48.71, 35.85, 31.20, 29.99, 29.91, 27.74, 26.28, 26.11, 22.50, 19.27, 15.23, 12.46, 6.52, 6.46. ^{19}F NMR (376 MHz, DMSO-d_6): δ -73.01. $C_{25}H_{37}F_3N_4O_5S$. HRMS calcd for m/z $[M+H]^+$: 563.2515 (calculated), 563.2526 (found).

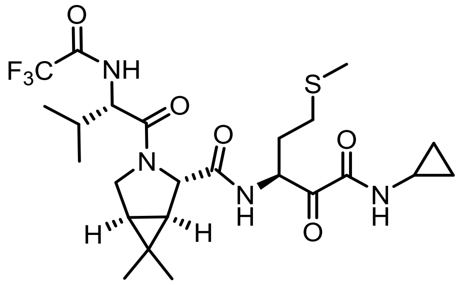

(1R,2S,5S)-N-((S)-1-(cyclopropylamino)-5-(methylthio)-1,2-dioxopentan-3-yl)-6,6-dimethyl-3-((2,2,2-trifluoroacetyl)-L-valyl)-3-azabicyclo[3.1.0]hexane-2-carboxamide (Jun13734). Jun13734 was prepared by an analogous procedure to **Jun1422**. Off white solid (65%). ^{1}H NMR (400 MHz, $CDCl_3$): δ 7.05 (d, J = 8.7 Hz, 1H), 7.00 (d, J = 7.4 Hz, 1H), 6.86 (dd, J = 14.8, 4.0 Hz, 1H), 5.31 (td, J = 7.2, 4.4 Hz, 1H), 4.46 (td, J = 8.9, 6.3 Hz, 1H), 4.30 (s, 1H), 3.86–3.78 (m, 1H), 3.72 (d, J = 10.2 Hz, 1H), 2.71 (tq, J = 7.5, 3.9 Hz, 1H), 2.08 (dq, J = 13.8, 6.7 Hz, 3H), 1.49 (d, J = 2.6 Hz, 2H), 1.34 (s, 1H), 1.19 (s, 2H), 0.99 (s, 3H), 0.94 (d, J = 6.6 Hz, 3H), 0.87–0.82 (m, 7H), 0.79 (d, J = 7.7 Hz, 3H), 0.54 (dt, J = 6.6, 3.1 Hz, 2H). ^{13}C NMR (101 MHz, $CDCl_3$): δ 195.30, 170.15, 169.33, 160.34, 157.26, 156.90, 117.25, 114.35, 60.93, 56.48, 54.41, 47.76, 31.23, 30.04, 29.68, 27.61, 26.18, 22.44, 19.37, 19.28, 17.36, 12.67, 6.50, 6.46. ^{19}F NMR (376 MHz, $CDCl_3$): δ -75.62. $C_{24}H_{35}F_3N_4O_5S$, HRMS calcd for m/z $[M+H]^+$: 549.2359 (calculated), 549.2382 (found).

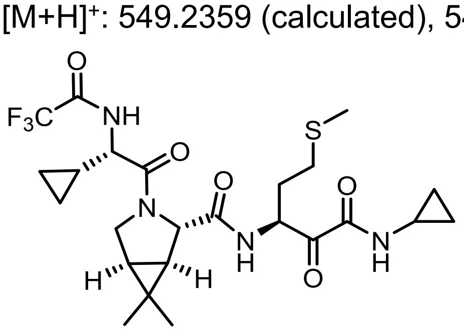

(1R,2S,5S)-3-((S)-2-cyclopropyl-2-(2,2,2-trifluoroacetamido)acetyl)-N-((S)-

1-(cyclopropylamino)-5-(methylthio)-1,2-dioxopentan-3-yl)-6,6-dimethyl-3-azabicyclo[3.1.0]hexane-2-carboxamide (Jun15575). Jun15575 was prepared by an analogous procedure to **Jun1422**. Off white solid (65%). ^{1}H NMR (400 MHz, $CDCl_3$): δ 8.15 (s, 1H), 7.71 (d, J = 7.4 Hz, 1H), 7.12 (d, J = 3.7 Hz, 1H), 5.21–5.13 (m, 1H), 4.55 (s, 1H), 3.92 (d, J = 10.8 Hz, 1H), 3.76 (dd, J = 10.9, 5.3 Hz, 1H), 2.74 (dq, J = 7.3, 3.6 Hz, 1H), 2.56 (p, J = 6.7 Hz, 2H), 2.34 (ddd, J = 14.6, 7.2, 5.0 Hz, 1H), 2.15 (dt, J = 14.3, 7.1 Hz, 1H), 2.04 (s, 3H), 1.88 (ddd, J = 10.6, 7.8, 5.2 Hz, 1H), 1.62 (d, J = 7.6 Hz, 1H), 1.55 (ddd, J = 10.5, 7.7, 5.8 Hz, 1H), 1.47 (dd, J = 7.5, 5.3 Hz, 1H), 1.21 (ddd, J = 10.6, 7.9, 5.7 Hz, 1H), 1.05 (s, 3H), 0.85 (s, 3H), 0.84 (s, 2H), 0.62 (dt, J = 6.8, 4.9 Hz, 2H). ^{13}C NMR (101 MHz, $CDCl_3$): δ 193.44, 171.12, 168.54, 161.25, 158.76, 158.00, 62.03, 55.00, 48.31, 36.49, 30.91, 29.99, 29.55, 27.43, 26.02, 22.39, 19.11, 15.36, 15.07, 14.56, 12.47, 6.46, 6.34. ^{19}F NMR (376 MHz, DMSO-d_6): δ -74.02. $C_{24}H_{33}F_3N_4O_5S$. HRMS calcd for m/z $[M+H]^+$: 547.2202 (calculated), 547.2206 (found).

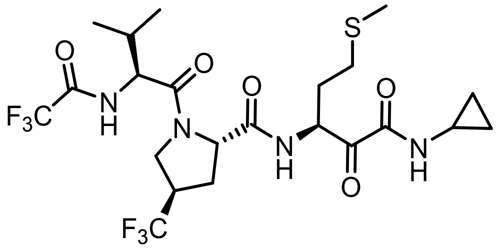

(2S,4R)-N-((S)-1-(cyclopropylamino)-5-(methylthio)-1,2-dioxopentan-3-yl)-1-((2,2,2-trifluoroacetyl)-L-valyl)-4-(trifluoromethyl)pyrrolidine-2-carboxamide (Jun15667). Jun15667 was prepared from its aldehyde by an analogous procedure to **Jun1422**. Off white solid (51%). ^{1}H NMR (400 MHz, $CDCl_3$): δ 7.40 (d, J = 7.2 Hz, 1H), 7.22 (d, J = 8.6 Hz, 1H), 6.98 (d, J = 3.9 Hz, 1H), 5.38–5.23 (m, 1H), 4.73 (d, J = 8.2 Hz, 1H), 4.64–4.52 (m, 1H), 3.87 (ddd, J = 13.2, 8.6, 3.5 Hz, 2H), 3.40 (h, J = 8.0 Hz, 1H), 2.78 (tq, J = 7.7, 4.0 Hz, 1H), 2.59–2.48 (m, 2H), 2.42 (ddd, J = 13.4, 7.3, 2.3 Hz, 1H), 2.32 (dtd, J = 14.0, 6.8, 4.6 Hz, 1H), 2.23–2.09 (m, 3H), 2.05 (s, 3H), 1.03 (d, J = 6.7 Hz, 3H), 0.96 (d, J = 6.6 Hz, 3H), 0.86 (d, J = 7.2 Hz, 2H), 0.61 (dt, J = 6.4, 3.2 Hz, 2H). ^{13}C NMR (101 MHz, $CDCl_3$): δ 194.92, 170.10, 169.79, 160.35, 157.33, 156.95, 127.28, 124.52, 117.19, 114.33, 59.50, 55.89, 54.36, 46.55, 42.47, 42.18, 31.54, 30.96, 29.91, 27.42, 22.50, 19.25, 17.43, 15.17, 6.51, 6.43. ^{19}F NMR (376 MHz, DMSO-d_6): δ -70.15, 73.58. $C_{22}H_{30}F_6N_4O_5S$, HRMS calcd for m/z $[M+H]^+$: 577.1919 (calculated), 577.1940 (found).

HCl (8 eq, 4 N in dioxane) was added to a solution of **5a** (1 eq) in anhydrous DCM, the reaction was stirred at rt for 2 h. After finished, remove the all solvent in vacuo. The residue was washed with DCM for 3 times, and was dried again by rotary evaporator to get the intermediate amine, which was used directly for the next step without purification. Amine (1 eq) and N,N-Diisopropylethylamine (4 eq) was dissolved in anhydrous DCM, then the reaction mixture was cooled at 0 °C, methyl chloroformate (3.6 eq) was slowly added, and the reaction was stirred at rt for 1 h. After finished, the reaction mixture was quenched by adding NH_4Cl aqueous solution, then DCM was added to extracted the mixture, combined organic layer was washed with NaCl aqueous solution, dried over Na_2SO_4, filtered and concentrated. The crude product was purified by column chromatography to afford intermediate **12a**.

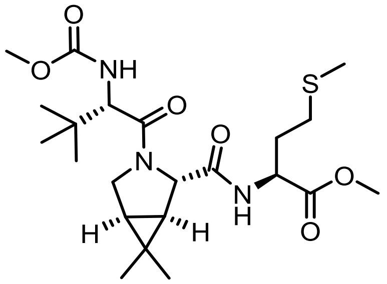

Methyl ((1R,2S,5S)-3-((S)-2-((methoxycarbonyl)amino)-3,3-dimethylbutanoyl)-6,6-dimethyl-3-azabicyclo[3.1.0]hexane-2-carbonyl)-L-methioninate (12a). Off white solid (68%). ^{1}H NMR (400 MHz, $CDCl_3$): δ 7.52 (d, J = 8.4 Hz, 1H), 6.09 (d, J = 10.0 Hz, 1H), 4.73 (td, J = 8.4, 4.5 Hz, 1H), 4.32 (s, 1H), 4.22 (d, J = 10.0 Hz, 1H), 3.88 (dd, J = 10.2, 4.8 Hz, 1H), 3.81 (d, J = 9.8 Hz, 1H), 3.70 (s, 3H), 3.56 (s, 3H), 2.53–2.40 (m, 2H), 1.96 (s, 3H), 1.91–1.80 (m, 1H), 1.52–1.44 (m, 2H), 1.21–1.14 (m, 1H), 0.97 (s, 3H), 0.90 (s, 9H), 0.82 (s, 3H). ^{13}C NMR (101 MHz, $CDCl_3$): δ 172.68, 170.97, 170.85, 157.04, 60.67, 59.29, 52.33, 51.12, 48.32, 35.47, 31.84, 30.33, 29.65, 27.85, 26.32, 26.15, 19.02, 15.04, 12.79. $C_{22}H_{37}N_3O_6S$, MS calcd for m/z $[M+H]^+$: 472.2 (calculated), 472.2 (found).

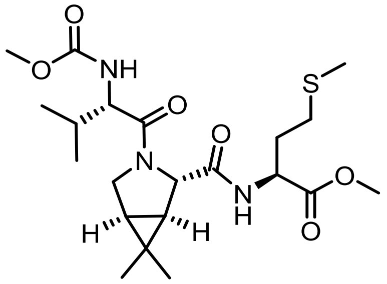

Methyl ((1R,2S,5S)-3-((methoxycarbonyl)-L-valyl)-6,6-dimethyl-3-azabicyclo [3.1.0]hexane-2-carbonyl)-L-methioninate (12b). 12b was prepared by

an analogous procedure to **12a**. Off white solid (75%). ^{1}H NMR (400 MHz, $CDCl_3$): δ 7.32–7.22 (m, 1H), 5.71 (d, J = 9.4 Hz, 1H), 4.71 (td, J = 7.9, 4.9 Hz, 1H), 4.38 (s, 1H), 4.18 (t, J = 8.2 Hz, 1H), 3.87 (d, J = 2.8 Hz, 2H), 3.75 (s, 3H), 3.63 (s, 3H), 2.50 (t, J = 7.5 Hz, 2H), 2.18–2.07 (m, 1H), 2.05 (s, 3H), 1.96 (ddd, J = 17.8, 9.1, 5.6 Hz, 2H), 1.60–1.51 (m, 2H), 1.04 (s, 3H), 0.95 (d, J = 6.8 Hz, 3H), 0.89 (d, J = 6.4 Hz, 6H). ^{13}C NMR (101 MHz, $CDCl_3$): δ 172.37, 171.43, 170.66, 157.05, 60.74, 58.01, 52.44, 52.22, 51.47, 47.67, 31.75, 31.07, 30.05, 29.79, 27.72, 26.22, 19.29, 19.13, 17.67, 15.26, 12.70. $C_{21}H_{35}N_3O_6S$. MS calcd for m/z $[M+H]^+$: 458.2 (calculated), 458.2 (found).

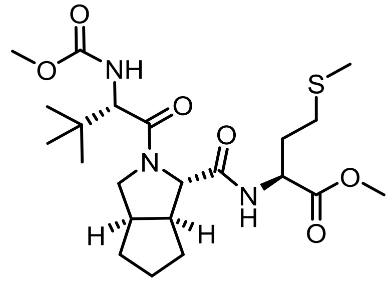

Methyl ((1S,3aR,6aS)-2-((S)-2-((methoxycarbonyl)amino)-3,3-dimethylbutanoyl)octahydrocyclopenta[c]pyrrole-1-carbonyl)-L-methioninate (12c). **12c** was prepared by an analogous procedure to **12a**. Yellow white solid (63%). ^{1}H NMR (400 MHz, $CDCl_3$): δ 7.57 (d, J = 8.3 Hz, 1H), 6.03 (d, J = 9.9 Hz, 1H), 4.72 (td, J = 8.1, 5.0 Hz, 1H), 4.40–4.32 (m, 2H), 3.87 (dd, J = 10.5, 7.6 Hz, 1H), 3.74 (s, 3H), 3.64 (s, 3H), 2.88–2.73 (m, 2H), 2.58–2.44 (m, 2H), 2.15–2.06 (m, 1H), 2.04 (s, 3H), 1.97–1.81 (m, 3H), 1.74–1.64 (m, 1H), 1.62–1.52 (m, 1H), 1.48–1.38 (m, 2H), 0.97 (s, 9H). ^{13}C NMR (101 MHz, $CDCl_3$): δ 172.61, 171.68, 171.27, 157.07, 66.60, 58.90, 54.49, 52.40, 52.16, 51.30, 46.37, 43.36, 35.66, 32.08, 31.99, 31.62, 29.94, 26.35, 25.45, 15.26. $C_{22}H_{37}N_3O_6S$, MS calcd for m/z $[M+H]^+$: 472.2 (calculated), 472.2 (found).

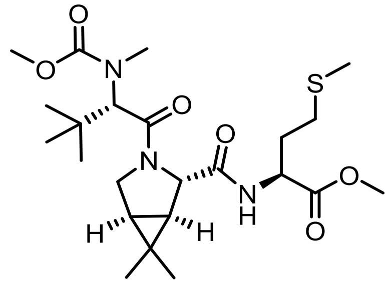

Methyl ((1R,2S,5S)-3-((S)-2-((methoxycarbonyl)(methyl)amino)-3,3-dimethylbutanoyl)-6,6-dimethyl-3-azabicyclo[3.1.0]hexane-2-carbonyl)-L-methioninate (12d). **12d** was prepared by an analogous procedure to **12a**. Yellow white solid (70%). ^{1}H NMR (400 MHz, $CDCl_3$): δ 7.14 (t, J = 8.4 Hz, 1H),

4.67 (s, 1H), 4.59–4.49 (m, 1H), 4.24 (s, 1H), 3.76 (dd, J = 10.6, 5.1 Hz, 1H), 3.68 (d, J = 10.5 Hz, 1H), 3.62 (d, J = 5.0 Hz, 6H), 2.84 (s, 3H), 2.42 (t, J = 7.6 Hz, 2H), 1.97 (s, 3H), 1.86 (dt, J = 14.2, 7.3 Hz, 1H), 1.44 (d, J = 7.6 Hz, 1H), 1.35 (dd, J = 7.6, 5.1 Hz, 1H), 1.14 (d, J = 3.8 Hz, 1H), 0.93 (s, 9H), 0.90 (s, 3H), 0.68 (s, 3H). ^{13}C NMR (101 MHz, $CDCl_3$): δ 172.09, 170.84, 169.04, 158.15, 61.48, 60.35, 52.99, 52.23, 51.58, 47.70, 36.27, 32.19, 31.71, 29.85, 29.81, 27.36, 26.03, 18.60, 15.24, 12.19. $C_{23}H_{39}N_3O_6S$, MS calcd for m/z $[M+H]^+$: 486.2 (calculated), 486.2 (found).

Ether **12a** (1 eq) was dissolved in anhydrous THF/MeOH (15:1, v/v), $NaBH_4$ (6 eq) was added slowly at 0 °C and the reaction mixture was stirred at rt for 2 h. After finished, saturated NH_4Cl solution was added to quench the reaction. Subsequently, the mixture was extracted with EtOAc for 3 times, combined organic phase was washed with NaCl aqueous solution for 2 times, dried over Na_2SO_4, filtered and concentrated. The reaction was transformed quantitative and the product alcohol **13a** was used directly for next step without further purification. Lately, alcohol **13a** went through the same procedure as described in the synthesis of **Jun12504** to get final product **Jun13699**.

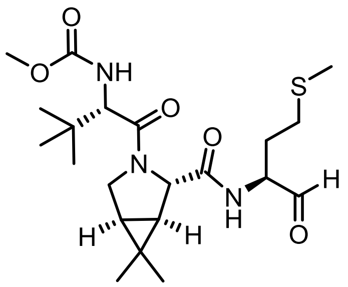

Methyl ((S)-1-((1R,2S,5S)-6,6-dimethyl-2-(((S)-4-(methylthio)-1-oxobutan-2-yl)carbamoyl)-3-azabicyclo[3.1.0]hexan-3-yl)-3,3-dimethyl-1-oxobutan-2-yl)carbamate (Jun13699). Off white solid (60%). ^{1}H NMR (400 MHz, $CDCl_3$): δ 9.56 (s, 1H), 7.16–7.06 (m, 1H), 5.47 (d, J = 10.0 Hz, 1H), 4.53 (td, J = 7.1, 4.9 Hz, 1H), 4.32 (s, 1H), 4.19 (d, J = 9.8 Hz, 1H), 3.84 (dp, J = 10.5, 4.9 Hz, 2H), 3.57 (s, 3H), 2.52–2.45 (m, 2H), 1.98 (s, 3H), 1.87 (dd, J = 14.4, 7.2 Hz, 1H), 1.56–1.44 (m, 2H), 0.98 (s, 3H), 0.91 (s, 9H), 0.82 (s, 3H). ^{13}C NMR (101 MHz, $CDCl_3$): δ 198.37, 171.57, 171.21, 157.06, 60.72, 59.48, 57.99, 52.38, 48.51, 35.33, 29.88, 29.72, 28.50, 27.83, 26.34, 19.14, 15.24, 12.63. $C_{21}H_{35}N_3O_5S$, HRMS calcd for m/z $[M+H]^+$: 442.2377 (calculated), 442.2392 (found).

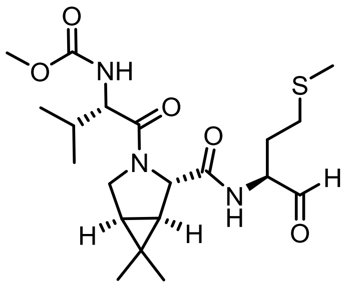

Methyl ((S)-1-((1R,2S,5S)-6,6-dimethyl-2-(((S)-4-(methylthio)-1-oxobutan-2-yl)carbamoyl)-3-azabicyclo[3.1.0]hexan-3-yl)-3-methyl-1-oxobutan-2-yl)carbamate (Jun15516). Jun15516 was prepared by an analogous procedure to **Jun12504**. Off white solid (66%). ^{1}H NMR (400 MHz, DMSO-d_6): δ 9.46 (s, 1H), 8.51 (d, J = 7.4 Hz, 1H), 7.36 (d, J = 8.2 Hz, 1H), 4.26 (d, J = 6.6 Hz, 2H), 3.90–3.77 (m, 3H), 3.51 (s, 3H), 2.56 (dt, J = 8.8, 5.1 Hz, 1H), 2.46 (dd, J = 13.5, 7.6 Hz, 1H), 2.03 (s, 3H), 1.89 (dt, J = 10.3, 5.3 Hz, 1H), 1.75 (ddt, J = 13.2, 8.7, 4.4 Hz, 1H), 1.52 (dd, J = 7.6, 4.6 Hz, 1H), 1.39 (d, J = 7.6 Hz, 1H), 1.04 (s, 3H), 0.92 (s, 3H), 0.88 (t, J = 7.1 Hz, 6H). ^{13}C NMR (101 MHz, DMSO-d_6): δ 201.43, 172.08, 170.56, 157.27, 60.33, 58.65, 57.66, 51.86, 47.35, 31.34, 30.00, 29.78, 28.35, 27.70, 26.47, 19.27, 15.01, 13.09. $C_{20}H_{33}N_3O_5S$, HRMS calcd for m/z $[M+H]^+$: 428.2219 (calculated), 428.2217 (found).

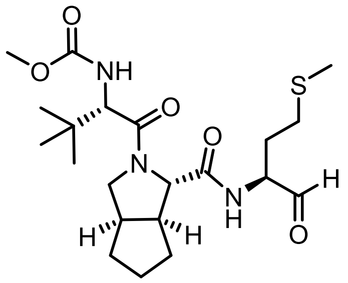

Methyl ((S)-3,3-dimethyl-1-((1S,3aR,6aS)-1-(((S)-4-(methylthio)-1-oxobutan-2-yl)carbamoyl)hexahydrocyclopenta[c]pyrrol-2(1H)-yl)-1-oxobutan-2-yl)carbamate (Jun13856). Jun13856 was prepared by an analogous procedure to **Jun12504**. Off white solid (70%). ^{1}H NMR (400 MHz, $CDCl_3$): δ 9.54 (s, 1H), 7.32 (d, J = 7.5 Hz, 1H), 5.61 (d, J = 9.8 Hz, 1H), 4.46 (dd, J = 7.5, 5.1 Hz, 1H), 4.32–4.24 (m, 2H), 3.77 (dd, J = 10.5, 7.2 Hz, 1H), 3.59 (s, 3H), 2.77 (tq, J = 7.4, 3.7 Hz, 2H), 2.53–2.41 (m, 2H), 2.20–2.10 (m, 1H), 1.98 (s, 3H), 1.91–1.78 (m, 3H), 1.73–1.61 (m, 1H), 1.59–1.48 (m, 1H), 1.48–1.36 (m, 2H), 0.91 (s, 9H). ^{13}C NMR (101 MHz, $CDCl_3$): δ 198.65, 171.92, 171.55, 156.99, 66.54, 58.93, 57.96, 54.43, 52.29, 46.02, 43.40, 35.57, 32.00, 31.57, 29.86, 28.51, 26.38, 25.43, 15.25. $C_{21}H_{35}N_3O_5S$, HRMS calcd for m/z $[M+H]^+$: 442.2376 (calculated), 442.2392 (found).

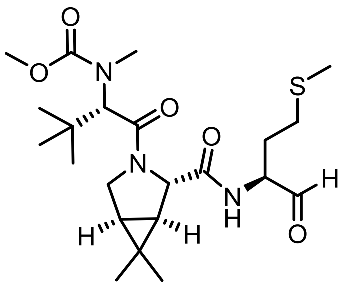

Methyl ((S)-1-((1R,2S,5S)-6,6-dimethyl-2-(((S)-4-(methylthio)-1-oxobutan-2-yl)carbamoyl)-3-azabicyclo[3.1.0]hexan-3-yl)-3,3-dimethyl-1-oxobutan-2-yl)(methyl)carbamate (Jun15514). Jun15514 was prepared by an analogous procedure to **Jun12504**. Off white solid (59%). ^{1}H NMR (400 MHz, $CDCl_3$): δ 9.63 (s, 1H), 7.27 (s, 1H), 4.78 (s, 1H), 4.60 (q, J = 6.3 Hz, 1H), 4.36 (d, J = 11.4 Hz, 1H), 3.90–3.79 (m, 2H), 3.74 (s, 3H), 2.95 (s, 3H), 2.57 (t, J = 7.0 Hz, 2H), 2.35–2.23 (m, 1H), 2.08 (s, 3H), 1.98 (dd, J = 14.4, 7.2 Hz, 1H), 1.59 (t, J = 9.9 Hz, 1H), 1.55–1.45 (m, 1H), 1.04 (d, J = 6.8 Hz, 12H), 0.83 (s, 3H). ^{13}C NMR (101 MHz, $CDCl_3$): δ 198.20, 171.50, 169.55, 158.40, 61.78, 60.56, 58.06, 53.26, 47.84, 36.48, 32.39, 29.77, 29.71, 28.51, 27.43, 26.14, 18.79, 15.34, 12.21. $C_{22}H_{37}N_3O_5S$, HRMS calcd for m/z $[M+H]^+$: 456.2532 (calculated), 456.2552 (found).

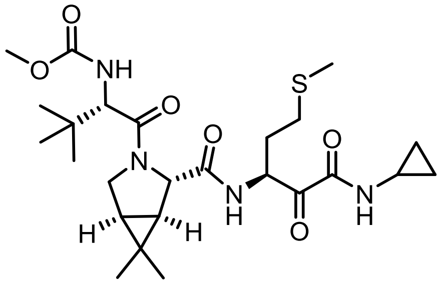

Methyl ((S)-1-((1R,2S,5S)-2-(((S)-1-(cyclopropylamino)-5-(methylthio)-1,2-dioxopentan-3-yl)carbamoyl)-6,6-dimethyl-3-azabicyclo[3.1.0]hexan-3-yl)-3,3-dimethyl-1-oxobutan-2-yl)carbamate (Jun13735). Jun13735 was prepared by an analogous procedure to **Jun1422**. Off white solid (48%). ^{1}H NMR (400 MHz, DMSO-*d6*): δ 8.66 (d, J = 5.2 Hz, 1H), 8.38 (d, J = 7.2 Hz, 1H), 6.99 (d, J = 8.8 Hz, 1H), 5.07–4.99 (m, 1H), 4.22 (s, 1H), 3.95 (d, J = 8.8 Hz, 1H), 3.71 (d, J = 3.5 Hz, 2H), 3.44 (s, 3H), 2.72–2.63 (m, 1H), 2.60–2.51 (m, 1H), 1.96 (s, 3H), 1.95 (s, 1H), 1.75–1.64 (m, 1H), 1.39 (dt, J = 6.6, 3.1 Hz, 1H), 1.25 (d, J = 7.6 Hz, 1H), 0.95 (s, 3H), 0.86 (s, 9H), 0.82 (s, 3H), 0.62–0.55 (m, 2H), 0.53–0.47 (m, 2H). ^{13}C NMR (101 MHz, DMSO-*d6*): δ 197.13, 171.61, 169.73, 162.50, 59.99, 59.80, 53.58, 51.92, 47.93, 34.78, 31.17, 30.07, 27.66, 26.85, 26.54, 22.94, 19.09, 14.93, 13.02, 5.87, 5.85. $C_{25}H_{40}N_4O_6S$, HRMS calcd for m/z $[M+H]^+$: 525.2749 (calculated), 525.2775 (found).

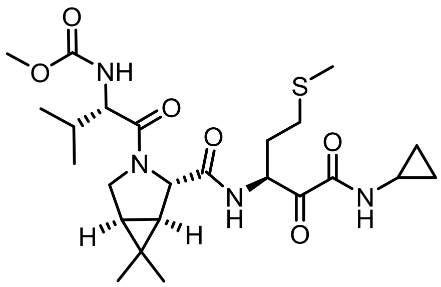

Methyl ((S)-1-((1R,2S,5S)-2-(((S)-1-(cyclopropylamino)-5-(methylthio)-1,2-dioxopentan-3-yl)carbamoyl)-6,6-dimethyl-3-azabicyclo[3.1.0]hexan-3-yl)-3-methyl-1-oxobutan-2-yl)carbamate (Jun15573). Jun15573 was prepared by an analogous procedure to **Jun1422**. Off white solid (54%). ^{1}H NMR (400 MHz, DMSO-*d*$_6$): δ 8.74 (d, J = 5.2 Hz, 1H), 8.47 (d, J = 6.9 Hz, 1H), 7.34 (d, J = 8.2 Hz, 1H), 5.06 (ddd, J = 10.4, 6.8, 3.7 Hz, 1H), 4.26 (s, 1H), 3.83 (s, 3H), 3.82 (s, 3H), 3.74 (dd, J = 10.1, 5.3 Hz, 2H), 2.78–2.70 (m, 1H), 2.61 (ddd, J = 13.3, 8.5, 4.8 Hz, 1H), 2.03 (s, 3H), 1.92–1.71 (m, 2H), 1.46 (dd, J = 7.6, 5.1 Hz, 1H), 1.32 (d, J = 7.6 Hz, 1H), 1.02 (s, 3H), 0.90 (s, 3H), 0.86 (t, J = 6.9 Hz, 6H), 0.65 (dq, J = 6.8, 4.1, 3.0 Hz, 2H), 0.57 (p, J = 4.4 Hz, 2H). ^{13}C NMR (101 MHz, DMSO-*d*$_6$): δ 197.15, 171.62, 170.53, 162.50, 157.27, 59.99, 58.70, 53.66, 51.86, 47.39, 31.24, 30.07, 29.93, 27.59, 26.60, 22.96, 19.39, 19.27, 19.20, 14.90, 13.09, 5.88, 5.86. $C_{24}H_{38}N_4O_6S$, HRMS calcd for m/z $[M+H]^+$: 511.2590 (calculated), 511.2606 (found).

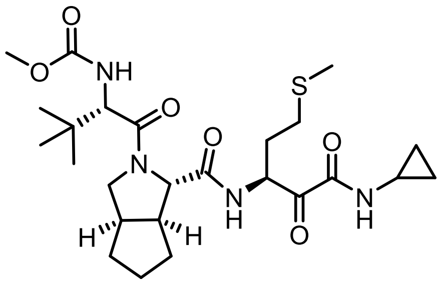

Methyl ((S)-1-((1S,3aR,6aS)-1-(((S)-1-(cyclopropylamino)-5-(methylthio)-1,2-dioxopentan-3-yl)carbamoyl)hexahydrocyclopenta[c]pyrrol-2(1H)-yl)-3,3-dimethyl-1-oxobutan-2-yl)carbamate (Jun13857). Jun13857 was prepared by an analogous procedure to **Jun1422**. Off white solid (52%). ^{1}H NMR (400 MHz, DMSO-*d*$_6$): δ 8.65 (d, J = 5.1 Hz, 1H), 8.24 (d, J = 7.2 Hz, 1H), 6.99 (d, J = 8.6 Hz, 1H), 5.05–4.97 (m, 1H), 4.14 (d, J = 4.1 Hz, 1H), 4.04 (d, J = 8.6 Hz, 1H), 3.67 (dd, J = 10.3, 7.5 Hz, 1H), 3.56 (dd, J = 10.6, 3.5 Hz, 1H), 3.46 (s, 3H), 2.71–2.63 (m, 1H), 2.60–2.51 (m, 2H), 1.96 (s, 3H), 1.81–1.66 (m, 3H), 1.62–1.45 (m, 3H), 1.34 (dt, J = 12.0, 6.1 Hz, 1H), 0.86 (s, 9H), 0.63–0.56 (m, 2H), 0.53–0.47 (m, 2H). ^{13}C NMR (101 MHz, DMSO-*d*$_6$): δ 196.99, 172.42,

170.03, 162.59, 157.33, 65.50, 59.45, 54.28, 53.49, 51.91, 47.72, 43.12, 34.90, 32.06, 31.74, 30.07, 30.00, 26.85, 25.14, 22.92, 14.91, 5.88, 5.86. $C_{25}H_{40}N_4O_6S$, HRMS calcd for m/z $[M+H]^+$: 525.2749 (calculated), 525.2775 (found).

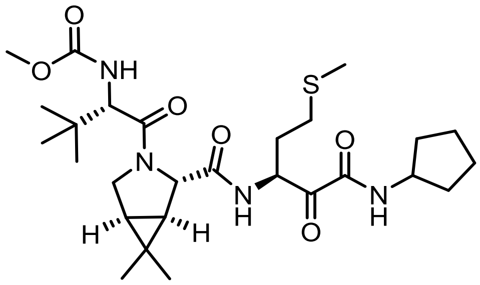

Methyl ((S)-1-((1R,2S,5S)-2-(((S)-1-(cyclopentylamino)-5-(methylthio)-1,2-dioxopentan-3-yl)carbamoyl)-6,6-dimethyl-3-azabicyclo[3.1.0]hexan-3-yl)-3,3-dimethyl-1-oxobutan-2-yl)carbamate (Jun15494). Jun15494 was prepared by an analogous procedure to **Jun1422**. Off white solid (49%). ^1H NMR (400 MHz, DMSO-d_6): δ 8.61 (d, J = 7.7 Hz, 1H), 8.43 (d, J = 7.4 Hz, 1H), 7.06 (d, J = 8.8 Hz, 1H), 5.09 (ddd, J = 10.6, 7.3, 3.5 Hz, 1H), 4.28 (s, 1H), 4.01 (dd, J = 8.1, 4.0 Hz, 2H), 3.77 (d, J = 3.6 Hz, 2H), 3.49 (s, 3H), 2.61 (ddd, J = 13.3, 8.5, 4.8 Hz, 1H), 2.52–2.43 (m, 2H), 2.01 (s, 3H), 1.77 (ddt, J = 14.5, 9.8, 5.4 Hz, 3H), 1.62 (d, J = 9.8 Hz, 2H), 1.54–1.42 (m, 5H), 1.31 (d, J = 7.7 Hz, 1H), 1.01 (s, 3H), 0.91 (s, 9H), 0.88 (s, 3H). ^{13}C NMR (101 MHz, DMSO-d_6): δ 197.58, 171.61, 169.73, 161.14, 60.02, 59.80, 53.73, 51.91, 50.83, 47.94, 34.78, 32.19, 31.20, 30.07, 27.68, 26.86, 26.54, 24.03, 19.10, 14.94, 13.04. $C_{27}H_{44}N_4O_6S$, HRMS calcd for m/z $[M+H]^+$: 553.3060 (calculated), 553.3083 (found).

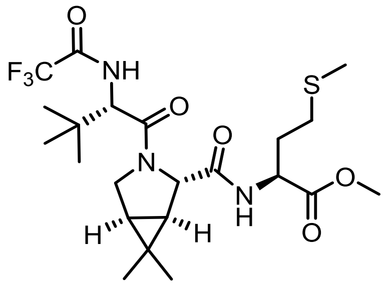

Methyl ((1R,2S,5S)-3-((S)-3,3-dimethyl-2-(2,2,2-trifluoroacetamido)butanoyl)-6,6-dimethyl-3-azabicyclo[3.1.0]hexane-2-carbonyl)-L-methioninate (16). 4a first went through the same procedure as described in the synthesis of **10a** to get intermediate **15**, then **15** went through the same procedure as described in the synthesis of **5a** to get intermediate **16**. Yellow white solid (three steps, yield 58%). ^1H NMR (400 MHz, $CDCl_3$): δ 7.46 (d, J = 8.0 Hz, 2H), 4.82 (td, J = 8.2, 5.1 Hz, 1H), 4.61 (d, J = 9.5 Hz, 1H), 4.40 (s, 1H), 4.00 (dd, J =

10.2, 5.3 Hz, 1H), 3.85 (d, J = 10.3 Hz, 1H), 3.77 (s, 3H), 2.52 (ddq, J = 20.8, 13.5, 7.5 Hz, 2H), 2.16–2.09 (m, 1H), 2.06 (s, 3H), 1.92 (dq, J = 14.0, 7.7 Hz, 1H), 1.59–1.54 (m, 1H), 1.49 (d, J = 7.6 Hz, 1H), 1.03 (s, 12H), 0.86 (s, 3H). ^{13}C NMR (101 MHz, $CDCl_3$): δ 172.71, 170.69, 168.96, 157.65, 157.28, 156.91, 156.53, 120.11, 117.25, 114.39, 111.54, 60.84, 57.98, 52.46, 51.14, 48.62, 35.84, 31.97, 30.46, 29.92, 27.66, 26.38, 26.05, 19.13, 15.24, 12.69. $C_{22}H_{34}F_3N_3O_5S$, MS calcd for m/z $[M+H]^+$: 510.2 (calculated), 510.2 (found).

Intermediate **16** dissolved in MeOH/H_2O (3:1), LiOH (2 eq) was added and the reaction mixture was stirred at rt for 2 h. After finished, solvent was removed under vacuum, the residue was dissolved into water, 1 N HCl was added and the forming participate was filtered, washed with water and dried to get the acid **17**. Acid **17** (1 eq) and N,O-Dimethylhydroxylamine hydrochloride (1.8 eq) were dissolved in anhydrous DMF at 0 °C, EDCI (1.3 eq) and HOBt (1.3 eq) were subsequently added, the reaction mixture stirred at 0 °C for 5 min. Et_3N (3 eq) was added to the reaction mixture and the reaction stirred at rt for 24 h. When finished, the reaction was quenched with water and EtOAc was added to extracted for 3 times, combined organic layer was washed with NaCl aqueous solution for 3 times, dried over Na_2SO_4, filtered and concentrated. The crude product was purified by column chromatography (Hexane : EtOAc = 1:1) to afford weinreb amide **18**.

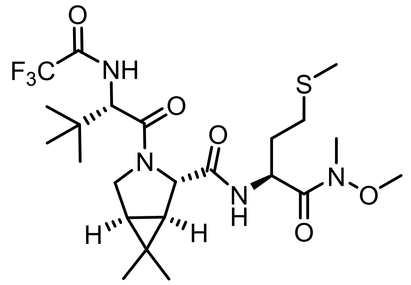

(1R,2S,5S)-3-((S)-3,3-dimethyl-2-(2,2,2-trifluoroacetamido)butanoyl)-N-((S)-1-(methoxy(methyl)amino)-4-(methylthio)-1-oxobutan-2-yl)-6,6-dimethyl-3-azabicyclo[3.1.0]hexane-2-carboxamide (18). Off white solid (62%). ^{1}H NMR (400 MHz, $CDCl_3$): δ 8.04 (d, J = 9.3 Hz, 1H), 7.86 (d, J = 9.1 Hz, 1H), 5.32 (s, 1H), 4.65 (d, J = 9.5 Hz, 1H), 4.45 (s, 1H), 4.03 (dd, J = 10.1, 5.3 Hz, 1H), 3.86 (d, J = 10.3 Hz, 1H), 3.80 (s, 3H), 3.22 (s, 3H), 2.60 (dq, J = 12.5, 6.9, 6.0 Hz, 1H), 2.48 (dt, J = 13.6, 7.8 Hz, 1H), 2.05 (s, 3H), 2.01 (d, J = 8.3 Hz, 1H), 1.85 (dd, J = 13.6, 7.6 Hz, 1H), 1.60–1.53 (m, 1H), 1.38 (d, J = 7.6 Hz, 1H), 1.04 (s, 9H), 1.01 (s, 3H), 0.85 (s, 3H). ^{13}C NMR (101 MHz, $CDCl_3$): δ 172.05, 170.92, 168.86, 157.85, 157.48, 157.10, 156.73, 120.18, 117.33,

114.47, 111.61, 61.73, 60.79, 58.12, 48.62, 47.78, 35.69, 31.83, 30.83, 30.12, 27.62, 26.52, 26.03, 19.03, 15.14, 12.77. $C_{23}H_{37}F_3N_4O_5S$, MS calcd for m/z $[M+H]^+$: 539.2 (calculated), 539.2 (found).

To a dried 100 ml round bottom flask were added benzothiazole (4 eq) and anhydrous THF, the flask was degassed and added a N_2 balllon. Then the solution of n-BuLi (1.6 M in Hexane, 3.5 eq) was slowly added to the mixture at -78 °C more than 10 min and kept stirred at -78 ^oC for 45 min. Weinreb amide **18** (1 eq) was dissolved in anhydrous THF and was dropwised to the reaction mixture, the reaction was stirred at -78 °C. After 1 h, the reaction was quenched with ice water, EtOAc was added to extract for 2 times, combined organic layer was washed with NaCl aqueous solution, dried over Na_2SO_4, filtered and concentrated. The crude product was purified by column chromatography (Hexane : EtOAc = 1 :1) to get the final compound **Jun13603**.

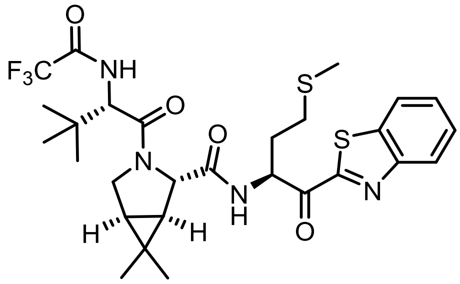

(1R,2S,5S)-N-((S)-1-(benzo[d]thiazol-2-yl)-4-(methylthio)-1-oxobutan-2-yl)-3-((S)-3,3-dimethyl-2-(2,2,2-trifluoroacetamido)butanoyl)-6,6-dimethyl-3-azabicyclo[3.1.0]hexane-2-carboxamide (Jun13603). Off white solid (70%). ^1H NMR (400 MHz, $CDCl_3$): δ 8.20 (d, J = 7.5 Hz, 1H), 8.06–7.93 (m, 1H), 7.66–7.53 (m, 2H), 7.33–7.24 (m, 1H), 7.10 (d, J = 9.4 Hz, 1H), 6.04 (td, J = 8.3, 4.2 Hz, 1H), 4.62 (d, J = 9.5 Hz, 1H), 4.46 (s, 1H), 3.98 (dd, J = 10.3, 4.7 Hz, 1H), 3.85 (d, J = 10.3 Hz, 1H), 2.66 (q, J = 7.3 Hz, 2H), 2.50 (dtd, J = 15.5, 7.7, 4.3 Hz, 1H), 2.14 (dt, J = 14.8, 7.5 Hz, 1H), 2.06 (s, 3H), 1.61–1.53 (m, 2H), 1.08 (s, 9H), 1.05 (s, 3H), 0.89 (s, 3H). ^{13}C NMR (101 MHz, $CDCl_3$): δ 192.78, 170.43, 168.99, 163.65, 157.53, 157.16, 156.79, 156.41, 153.45, 137.28, 128.16, 127.25, 125.81, 122.40, 120.13, 117.27, 114.41, 111.55, 61.06, 57.91, 55.02, 48.60, 36.06, 32.54, 30.16, 27.69, 26.38, 26.16, 19.20, 15.27, 12.60. ^{19}F NMR (376 MHz, $CDCl_3$): δ -75.56. $C_{28}H_{35}F_3N_4O_4S_2$, HRMS calcd for m/z $[M+H]^+$: 613.2135 (calculated), 613.2162 (found).

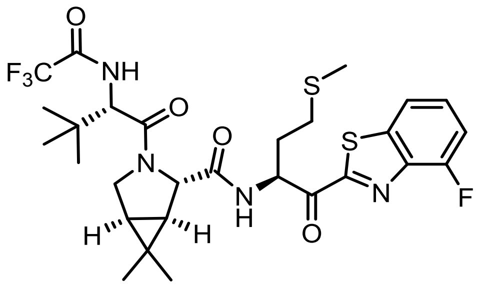

(1R,2S,5S)-3-((S)-3,3-dimethyl-2-(2,2,2-trifluoroacetamido)butanoyl)-N-((S)-1-(4-fluorobenzo[d]thiazol-2-yl)-4-(methylthio)-1-oxobutan-2-yl)-6,6-dimethyl-3-azabicyclo[3.1.0]hexane-2-carboxamide (Jun13604). Jun13604 was prepared by an analogous procedure to **Jun13603**. Off white solid (56%). ^1H NMR (400 MHz, $CDCl_3$): δ 7.73–7.64 (m, 1H), 7.45 (td, J = 8.1, 4.6 Hz, 1H), 7.27–7.15 (m, 2H), 7.05 (d, J = 9.5 Hz, 1H), 5.96 (td, J = 8.3, 4.1 Hz, 1H), 4.54 (d, J = 9.4 Hz, 1H), 4.39 (s, 1H), 3.90 (dd, J = 10.3, 4.9 Hz, 1H), 3.77 (d, J = 10.4 Hz, 1H), 2.59 (t, J = 7.3 Hz, 2H), 2.51–2.40 (m, 1H), 2.11–2.03 (m, 1H), 2.00 (s, 3H), 1.53–1.45 (m, 2H), 0.99 (s, 9H), 0.97 (s, 3H), 0.81 (s, 3H). ^{13}C NMR (101 MHz, $CDCl_3$): δ 192.65, 170.45, 169.03, 164.22, 158.59, 157.54, 157.17, 156.79, 156.42, 156.00, 142.82, 142.68, 139.63, 139.61, 129.19, 129.12, 118.09, 118.04, 117.26, 114.40, 112.68, 112.51, 60.98, 57.93, 55.04, 48.61, 36.04, 32.21, 30.15, 27.67, 26.37, 26.14, 19.18, 15.10, 12.60. ^{19}F NMR (376 MHz, $CDCl_3$): δ -75.55, -118.43. $C_{28}H_{34}F_4N_4O_4S_2$, HRMS calcd for m/z $[M+H]^+$: 631.2036 (calculated), 631.2067 (found).

$SOCl_2$ (8 eq) was slowly added to **2a** in anhydrous MeOH at 0 °C, then the reaction mixture was stirred at rt for overnight. After the reaction was done, the solvent was directly removed in vacuo to get the crude **19a**. **19a** then substituted with the methyl chloroformate and hydrolyzed to get the intermediate **20a**, **20b** was synthesized as mentioned of **20a**.

HCl (8 eq, 4 N in dioxane) was added to a solution of **7** in anhydrous DCM, the reaction was stirred at rt for overnight. After finished, remove the all solvent in vacuo to get the intermediate amine **21**, **21** (1 eq) and acid **20a** or **20b** (1.5 eq) went through the similar procedure of intermediate **3a** to get **22a** or **22b**.

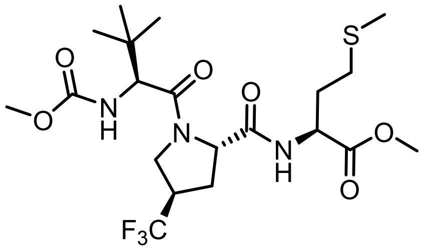

Methyl ((2S,4R)-1-((S)-2-((methoxycarbonyl)amino)-3,3-dimethylbutanoyl) -4-(trifluoromethyl)pyrrolidine-2-carbonyl)-L-methioninate (22a). ^{1}H NMR (400 MHz, $CDCl_3$): δ 7.59 (d, J = 8.3 Hz, 1H), 6.04 (d, J = 9.7 Hz, 1H), 4.66 (td, J = 8.3, 5.0 Hz, 2H), 4.27 (d, J = 9.7 Hz, 1H), 3.88 (p, J = 11.1, 10.6 Hz, 2H), 3.69 (s, 3H), 3.60 (s, 3H), 3.36 (dt, J = 16.5, 8.5 Hz, 1H), 2.48 (d, J = 19.6 Hz, 2H), 2.30 (ddd, J = 13.2, 7.3, 2.8 Hz, 1H), 2.12–2.04 (m, 2H), 2.03–1.92 (m, 3H), 1.86 (dq, J = 14.4, 7.5 Hz, 1H), 0.93 (s, 9H). ^{13}C NMR (101 MHz, $CDCl_3$): δ 172.36, 171.61, 170.64, 157.02, 127.47, 124.71, 59.25, 58.94, 52.46, 52.28, 51.27, 47.15, 42.78, 42.49, 42.19, 41.90, 35.52, 31.72, 29.74, 27.59, 26.18, 15.12. $C_{20}H_{32}F_3N_3O_6S$, MS calcd for m/z $[M+H]^+$: 500.2 (calculated), 500.2 (found).

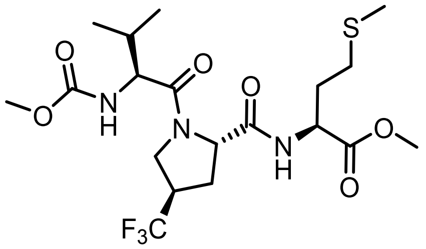

Methyl ((2S,4R)-1-((methoxycarbonyl)-L-valyl)-4-(trifluoromethyl)pyrroledine-2-carbonyl)-L-methioninate (22b). ^{1}H NMR (400 MHz, $CDCl_3$): δ 7.79 (d, J = 8.4 Hz, 1H), 6.15 (d, J = 9.1 Hz, 1H), 4.71 (qd, J = 8.2, 3.7 Hz, 2H), 4.24 (t, J = 8.4 Hz, 1H), 4.02–3.88 (m, 2H), 3.76 (s, 3H), 3.67 (s, 3H), 3.47 (dt, J = 16.7, 8.4 Hz, 1H), 2.51 (dq, J = 13.2, 6.0 Hz, 2H), 2.43–2.33 (m, 1H), 2.17–2.08 (m, 2H), 2.06 (s, 3H), 1.96 (dq, J = 16.3, 6.7, 6.0 Hz, 2H), 0.96 (t, J = 6.8 Hz, 6H). ^{13}C NMR (101 MHz, $CDCl_3$): δ 172.40, 172.11, 170.58, 157.00, 127.49, 124.73, 59.25, 57.57, 52.39, 52.20, 51.28, 46.40, 42.71, 42.42, 42.13, 41.84, 31.62, 31.16, 29.77, 27.61, 19.08, 17.76, 15.09. $C_{19}H_{30}F_3N_3O_6S$, MS calcd for m/z $[M+H]^+$: 486.2 (calculated), 486.2 (found).

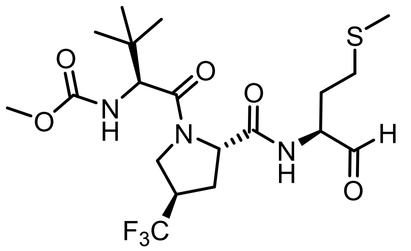

Methyl ((S)-3,3-dimethyl-1-((2S,4R)-2-(((S)-4-(methylthio)-1-oxobutan-2-yl)carbamoyl)-4-(trifluoromethyl)pyrrolidin-1-yl)-1-oxobutan-2-yl)carbamate (Jun15515). Jun15515 was prepared from **22a** by an analogous procedure to **Jun13699**. Off white solid (57%). ^{1}H NMR (400 MHz, DMSO-d_6): δ 9.33 (s, 1H), 8.38 (d, J = 7.5 Hz, 1H), 7.07 (d, J = 8.7 Hz, 1H), 4.42 (dd, J = 8.4, 5.6 Hz,

1H), 4.16 (td, J = 8.5, 4.2 Hz, 1H), 4.02 (d, J = 8.5 Hz, 1H), 3.82 (d, J = 6.8 Hz, 2H), 3.56–3.49 (m, 1H), 3.41 (s, 3H), 3.26 (q, J = 10.0, 8.8 Hz, 1H), 2.45 (td, J = 8.3, 4.4 Hz, 1H), 2.33 (dd, J = 13.7, 7.5 Hz, 1H), 2.17 (dq, J = 14.6, 7.7, 6.7 Hz, 1H), 2.03 (dt, J = 13.3, 6.7 Hz, 1H), 1.90 (s, 3H), 1.61 (dtd, J = 14.2, 8.7, 5.3 Hz, 1H), 0.82 (s, 9H). ^{13}C NMR (101 MHz, DMSO-*d6*): δ 201.24, 172.04, 170.21, 157.42, 59.47, 58.99, 57.70, 51.99, 47.18, 34.94, 29.75, 28.99, 28.34, 26.67, 26.53, 14.97. ^{19}F NMR (376 MHz, DMSO-*d6*): δ -70.10. $C_{19}H_{30}F_3N_3O_5S$, HRMS calcd for m/z $[M+H]^+$: 470.1937 (calculated), 470.1930 (found).

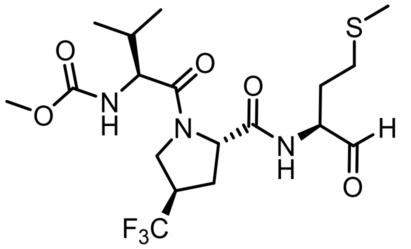

Methyl ((S)-3-methyl-1-((2S,4R)-2-(((S)-4-(methylthio)-1-oxobutan-2-yl)carbamoyl)-4-(trifluoromethyl)pyrrolidin-1-yl)-1-oxobutan-2-yl)carbamate (Jun15634). Jun15634 was prepared from **22b** by an analogous procedure to **Jun13699**. Off white solid (52%). ^{1}H NMR (400 MHz, DMSO-*d6*): δ 9.46 (s, 1H), 8.51 (d, J = 7.3 Hz, 1H), 7.44 (d, J = 8.2 Hz, 1H), 4.56 (dd, J = 8.5, 4.9 Hz, 1H), 4.26 (td, J = 8.4, 4.3 Hz, 1H), 4.02–3.93 (m, 4H), 3.53 (s, 3H), 3.40 (h, J = 8.1 Hz, 1H), 2.57 (ddd, J = 13.9, 8.7, 5.4 Hz, 1H), 2.47 (dd, J = 13.8, 7.5 Hz, 1H), 2.30 (dq, J = 15.2, 8.2, 6.7 Hz, 1H), 2.22–2.13 (m, 1H), 2.04 (s, 3H), 1.92 (p, J = 7.3 Hz, 1H), 1.76 (ddt, J = 13.5, 8.9, 4.5 Hz, 1H), 0.89 (d, J = 6.7 Hz, 6H). ^{13}C NMR (101 MHz, DMSO-*d6*): δ 201.26, 172.00, 171.00, 157.28, 58.98, 58.32, 57.73, 51.93, 46.55, 30.18, 29.75, 29.05, 28.24, 19.18, 19.04, 14.96. ^{19}F NMR (376 MHz, DMSO-*d6*): δ -70.05. $C_{18}H_{28}F_3N_3O_5S$, HRMS calcd for m/z $[M+H]^+$: 456.1780 (calculated), 456.1775 (found).

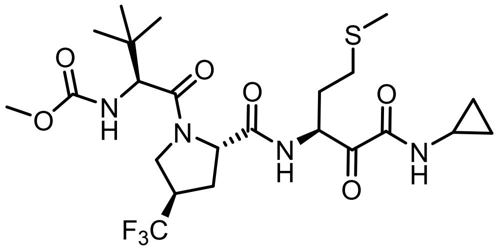

Methyl ((S)-1-((2S,4R)-2-(((S)-1-(cyclopropylamino)-5-(methylthio)-1,2-dioxopentan-3-yl)carbamoyl)-4-(trifluoromethyl)pyrrolidin-1-yl)-3,3-dimethyl-1-oxobutan-2-yl)carbamate (Jun15635). Jun15635 was prepared

from its aldehyde by an analogous procedure to **Jun1422**. Off white solid (41%). ^{1}H NMR (400 MHz, $CDCl_3$): δ 7.42 (d, J = 7.2 Hz, 1H), 6.98 (d, J = 3.9 Hz, 1H), 5.57 (d, J = 9.6 Hz, 1H), 5.35 (td, J = 7.3, 4.5 Hz, 1H), 4.74 (d, J = 8.6 Hz, 1H), 4.30 (d, J = 9.6 Hz, 1H), 3.92 (d, J = 8.5 Hz, 2H), 3.68 (s, 3H), 3.36 (q, J = 8.4 Hz, 1H), 2.78 (tq, J = 7.7, 4.0 Hz, 1H), 2.59–2.50 (m, 2H), 2.45 (ddd, J = 13.1, 7.2, 2.2 Hz, 1H), 2.32 (ddd, J = 14.9, 7.3, 4.9 Hz, 1H), 2.09 (d, J = 8.4 Hz, 1H), 2.04 (s, 3H), 1.02 (s, 9H), 0.86 (d, J = 7.5 Hz, 2H), 0.60 (tt, J = 8.4, 6.8, 5.1 Hz, 2H). ^{13}C NMR (101 MHz, DMSO-d_6): δ 196.80, 171.54, 170.11, 162.46, 157.41, 59.41, 58.70, 53.73, 51.98, 47.06, 34.91, 30.00, 29.92, 28.87, 26.68, 26.52, 22.94, 14.86, 5.85, 5.81. ^{19}F NMR (376 MHz, DMSO-d_6): δ -70.04. $C_{23}H_{35}F_3N_4O_6S$, HRMS calcd for m/z $[M+H]^+$: 553.2308 (calculated), 553.2320 (found).

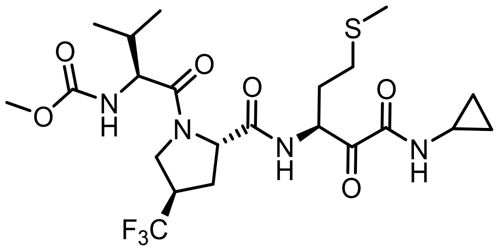

Methyl ((S)-1-((2S,4R)-2-(((S)-1-(cyclopropylamino)-5-(methylthio)-1,2-dioxopentan-3-yl)carbamoyl)-4-(trifluoromethyl)pyrrolidin-1-yl)-3-methyl-1-oxobutan-2-yl)carbamate (15636). Jun15636 was prepared from its aldehyde by an analogous procedure to **Jun1422**. Off white solid (44%). ^{1}H NMR (400 MHz, $CDCl_3$): δ 7.41 (d, J = 7.0 Hz, 1H), 6.96 (d, J = 3.9 Hz, 1H), 5.47 (d, J = 9.0 Hz, 1H), 5.31 (dt, J = 7.6, 4.1 Hz, 1H), 4.74 (d, J = 8.3 Hz, 1H), 4.25 (t, J = 8.1 Hz, 1H), 3.88 (dt, J = 25.2, 9.9 Hz, 2H), 3.68 (s, 3H), 3.36 (d, J = 8.4 Hz, 1H), 2.78 (tt, J = 6.4, 3.3 Hz, 1H), 2.50 (dtt, J = 21.5, 13.8, 6.5 Hz, 3H), 2.35–2.24 (m, 1H), 2.09 (d, J = 6.4 Hz, 1H), 2.04 (s, 3H), 1.99 (q, J = 6.8 Hz, 1H), 0.97 (dd, J = 15.0, 6.7 Hz, 6H), 0.87–0.82 (m, 2H), 0.65–0.56 (m, 2H). ^{13}C NMR (101 MHz, $CDCl_3$): δ 194.89, 169.93, 160.40, 157.09, 59.32, 57.50, 54.17, 52.51, 46.48, 31.23, 30.88, 29.94, 27.01, 22.48, 19.30, 17.67, 15.17, 6.50, 6.45. ^{19}F NMR (376 MHz, DMSO-d_6): δ -69.97. $C_{22}H_{33}F_3N_4O_6S$, HRMS calcd for m/z $[M+H]^+$: 539.2151 (calculated), 539.2163 (found).

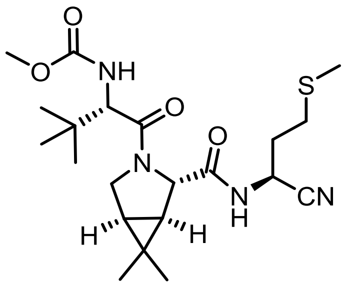

Compound **Jun15467** was synthesized according to the literature.[29] **12a** was added to the mixture of NH_3H_2O (20 eq) in MeOH at rt, then the reaction mixture was stirred at 46 ^oC for overnight and monitored by TLC. After the reaction done, the solvent was concentrated and extracted with EtOAc, then the conbined organic layer was collected, dried with Na_2SO_4, filtered and dried under vacuum to get the crude intermediate carbamate **24**. **24** was dissolved in anhydrous DCM, then the Burgess reagent (2.5 eq) was slowly added to the mixture at 0 ^oC and the reaction was stirred at 45 ^oC for 30 min. When finished (checked by TLC), H_2O was added to quench the reaction and the organic layer was separated, washed with NaCl aqueous solution, dried with Na_2SO_4, filtered and concentrated. The crude product was purified by Prep-HPLC to give the final product. ^1H NMR (400 MHz, $CDCl_3$): δ 8.53 (s, 1H), 7.57 (d, J = 8.6 Hz, 1H), 5.92 (d, J = 9.8 Hz, 1H), 5.15 (q, J = 7.5 Hz, 1H), 4.33 (s, 1H), 4.29 (d, J = 9.8 Hz, 1H), 3.95 (d, J = 3.0 Hz, 2H), 3.66 (s, 3H), 2.62 (t, J = 7.2 Hz, 2H), 2.09 (s, 3H), 2.07 (d, J = 7.6 Hz, 1H), 1.60 (dt, J = 14.7, 6.1 Hz, 2H), 1.08 (s, 3H), 0.99 (s, 9H), 0.91 (s, 3H). ^{13}C NMR (101 MHz, $CDCl_3$): δ 171.86, 170.56, 157.22, 117.65, 60.74, 59.57, 52.47, 48.63, 39.53, 35.34, 32.38, 29.37, 28.01, 26.36, 26.13, 19.27, 15.21, 12.62. $C_{21}H_{34}N_4O_4S$, HRMS calcd for m/z $[M+H]^+$: 439.2379 (calculated), 439.2397 (found).

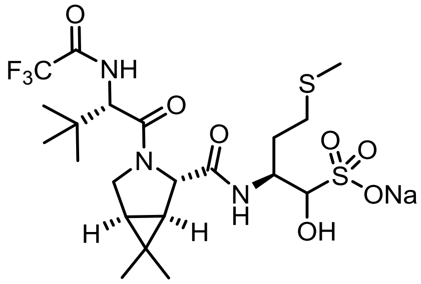

The bisulfite compound **Jun12607** was synthesized according to literatures.[30, 31] Aldehyde (0.2 mmol) and sodium bisulfite (0.2 ml, 1M) were dissolved in 3.5 mL EtOAc/EtOH/H_2O (4:2:1) and the mixture was stirred at 50 ^oC for 4 h. After finished (checked by LCMS), the reaction mixture was cooled to room temperature and filtered through a plug of anhydrous Na_2SO_4. The remaining

solid in the filter was thoroughly washed with absolute ethanol, and the filtrate was dried over anhydrous sodium sulfate, filtered, and concentrated to yield an off-white solid which was washed with anhydrous diethyl ether (3 x 10 mL), vacuum filtered and dried in vacuo to obtain desired compounds as white-powder. ^{1}H NMR (400 MHz, DMSO-*d₆*): δ 9.40 (s, 1H), 7.63 (d, J = 9.0 Hz, 1H), 5.65 (dd, J = 40.5, 6.3 Hz, 1H), 4.46 (s, 1H), 4.23 (t, J = 17.5 Hz, 2H), 4.08 (dq, J = 12.9, 6.5, 5.8 Hz, 1H), 4.00–3.88 (m, 2H), 3.72 (ddd, J = 17.1, 11.4, 4.7 Hz, 1H), 2.88 (d, J = 64.5 Hz, 1H), 2.05 (s, 3H), 1.41 (s, 1H), 1.24 (t, J = 7.1 Hz, 1H), 1.14 (d, J = 3.7 Hz, 2H), 1.05 (s, 12H), 0.89 (s, 3H). ^{13}C NMR (101 MHz, DMSO-*d_6*): δ 170.45, 167.54, 83.77, 61.08, 60.21, 58.72, 48.13, 35.21, 30.66, 30.57, 27.51, 26.85, 26.46, 21.22, 18.86, 15.19, 14.54, 12.85. ^{19}F NMR (376 MHz, DMSO-*d_6*): δ -73.00. $C_{21}H_{33}F_3N_3NaO_7S_2$, HRMS calcd for m/z $[M\text{-}Na+H]^+$: 560.1712 (calculated), 560.1780 (found).

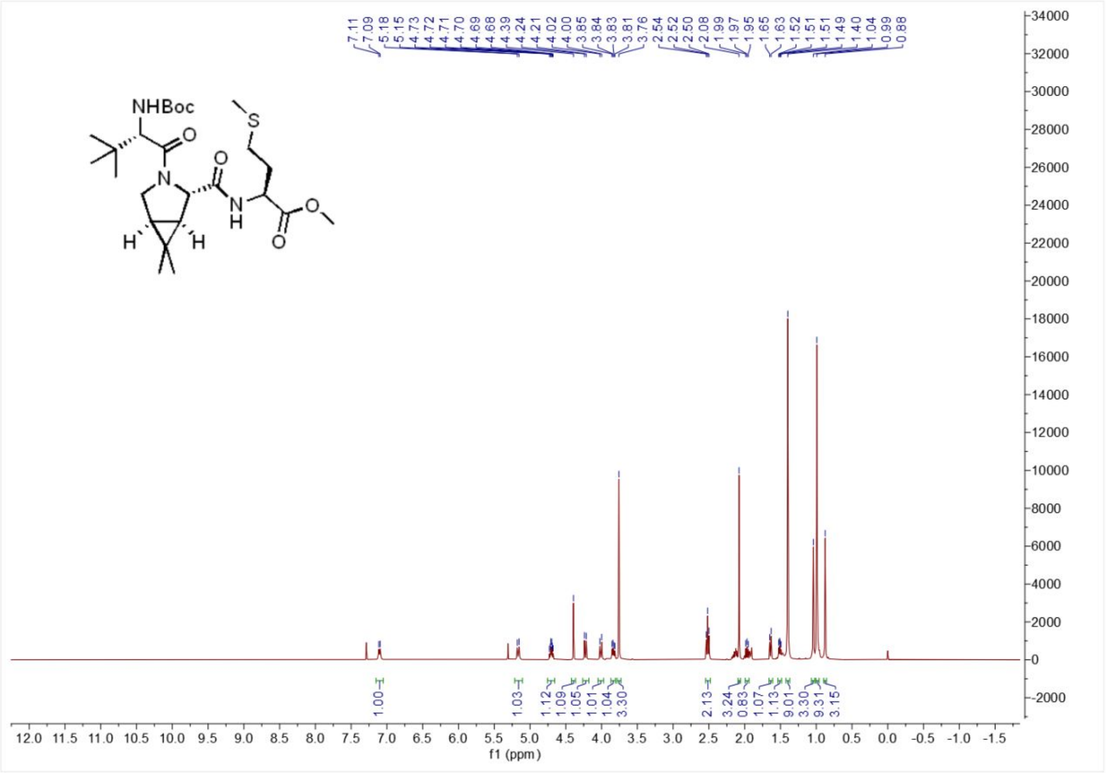

^{1}H NMR spectra of **5a**

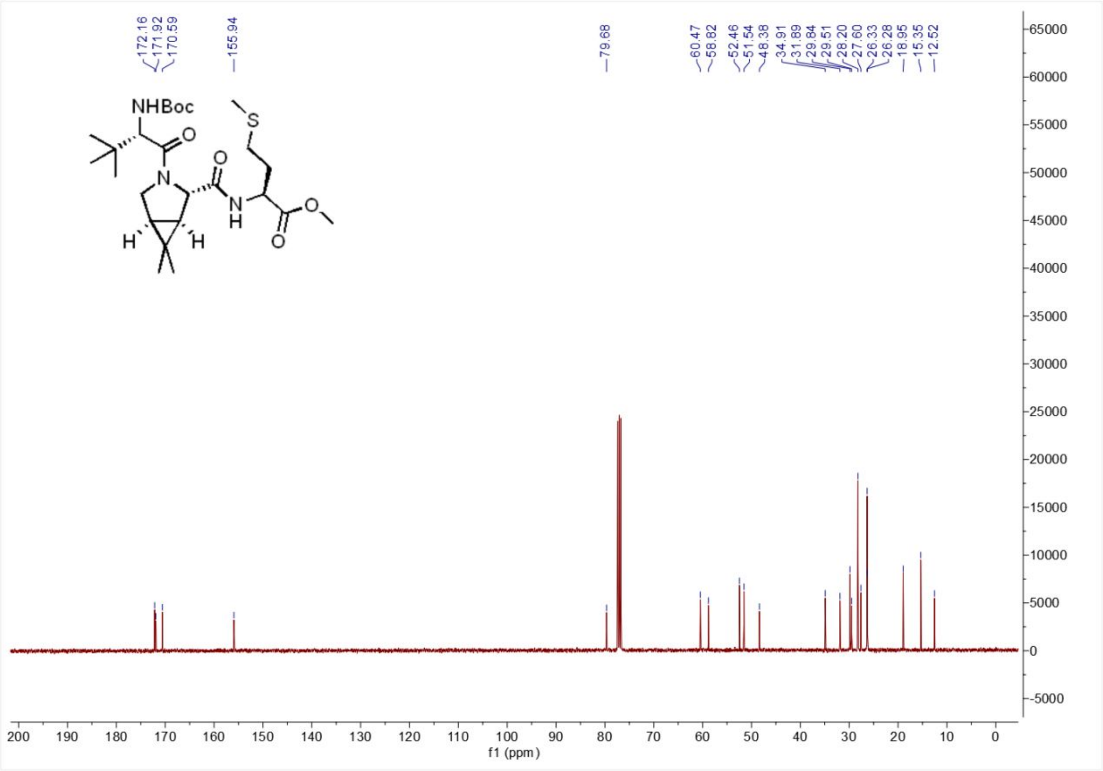

^{13}C NMR spectra of **5a**

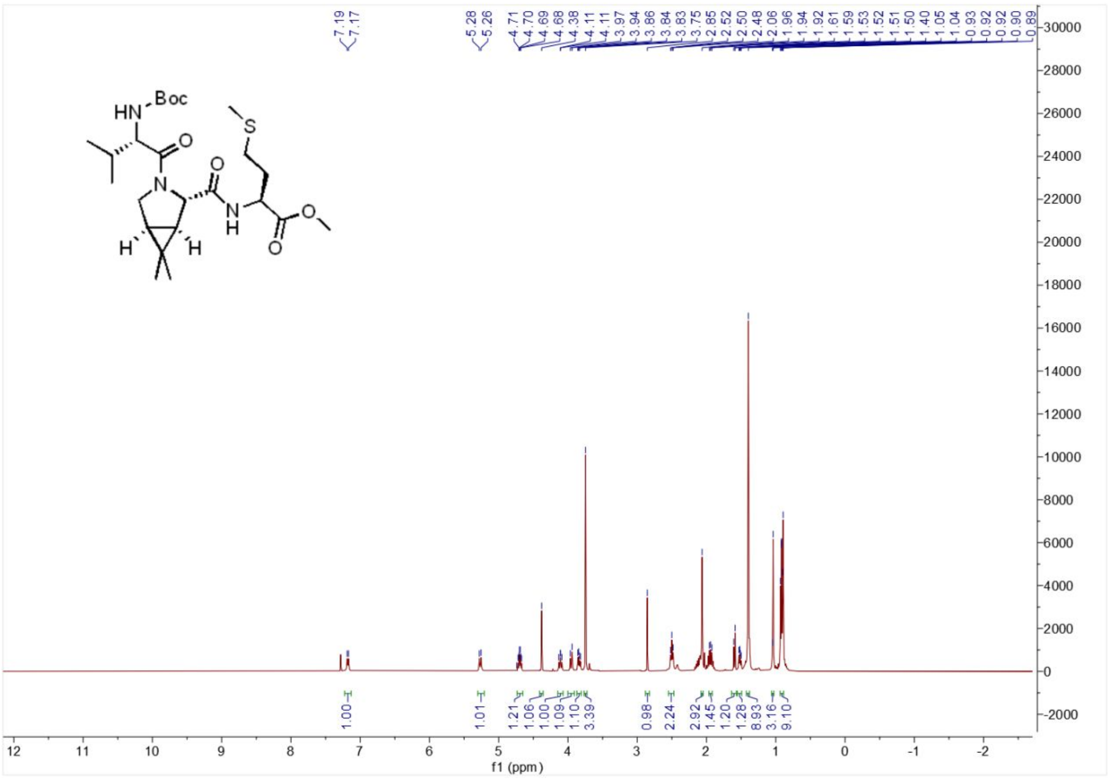

^{1}H NMR spectra of **5b**

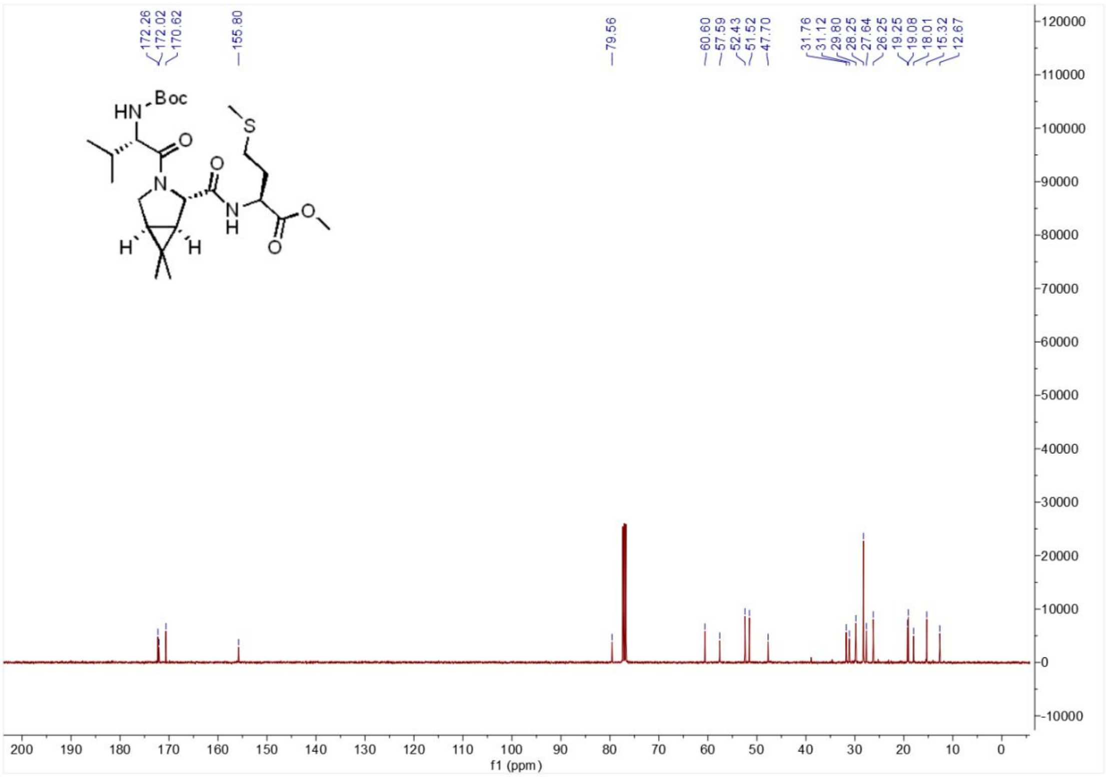

^{13}C NMR spectra of **5b**

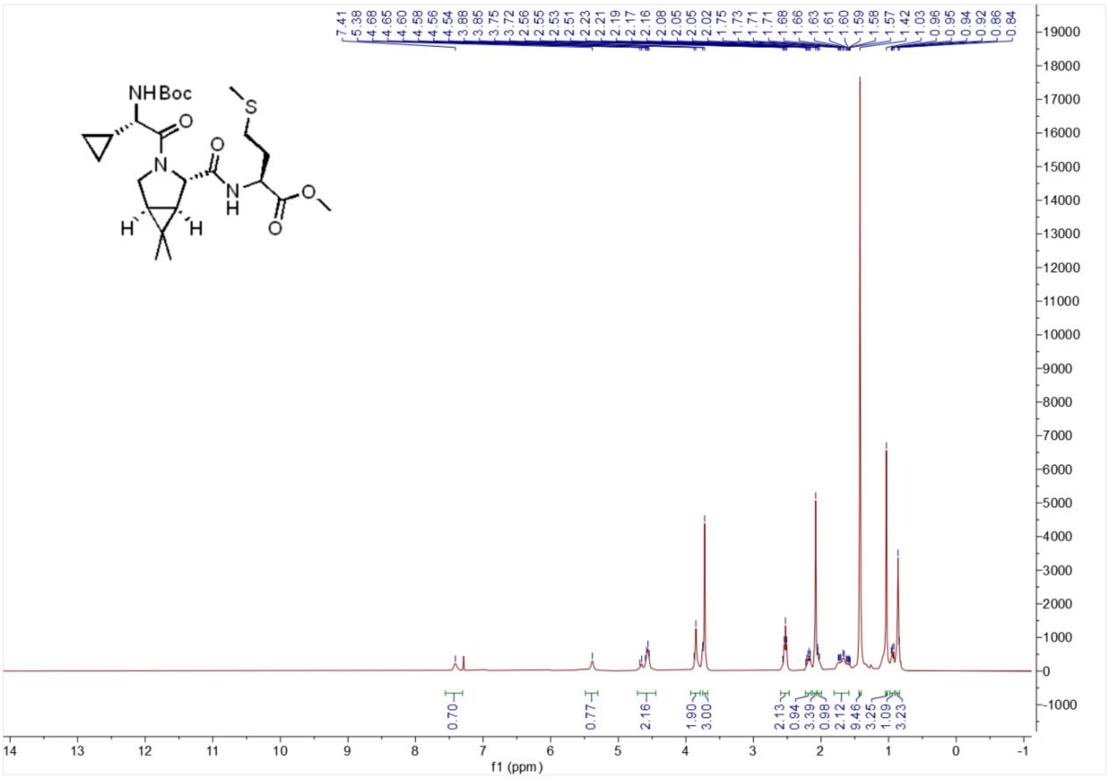

^{1}H NMR spectra of **5c**

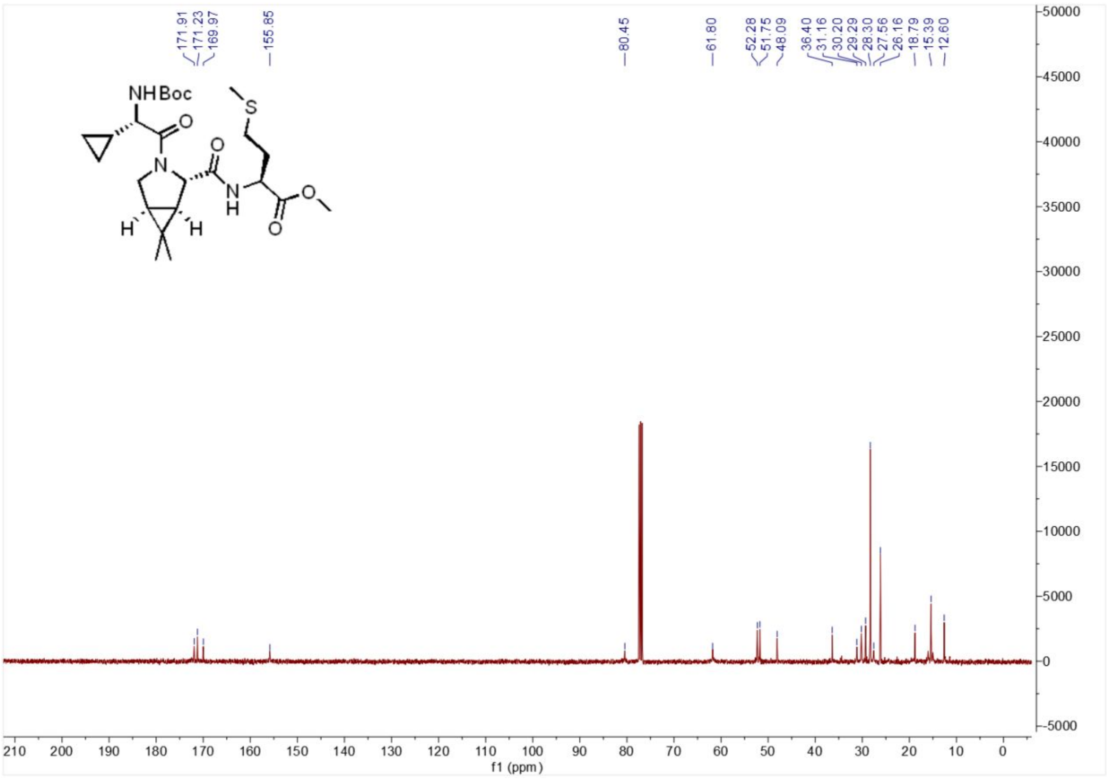

^{13}C NMR spectra of **5c**

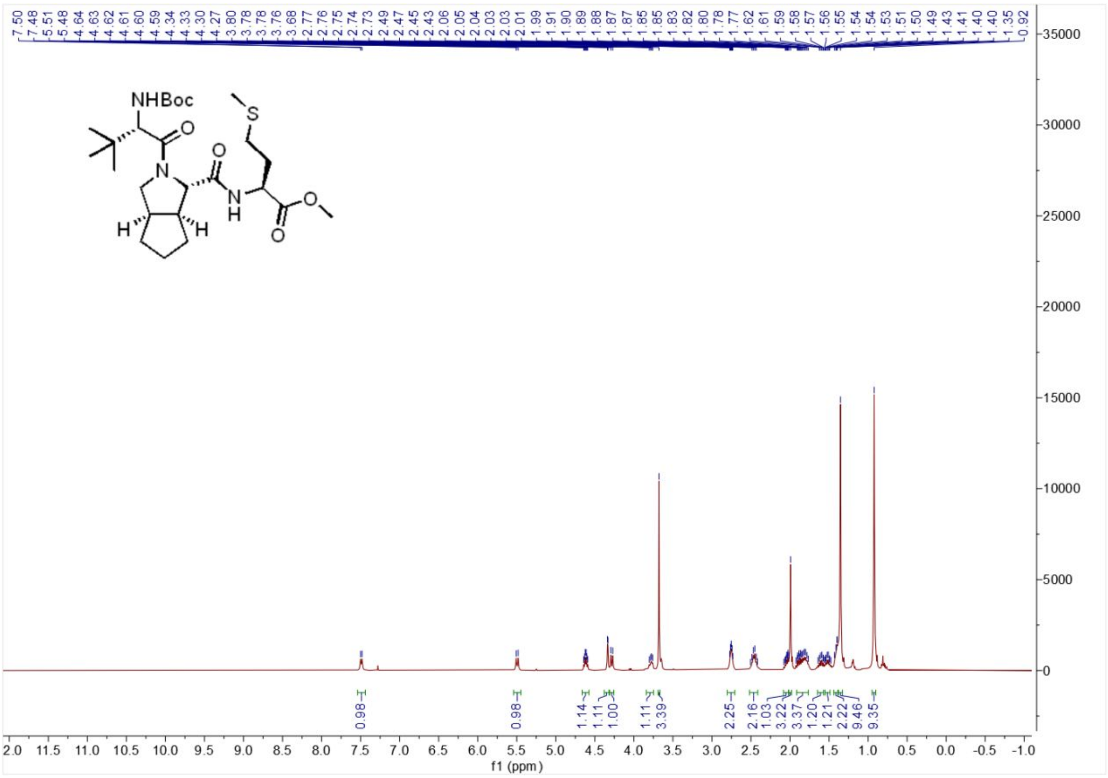

^{1}H NMR spectra of **5d**

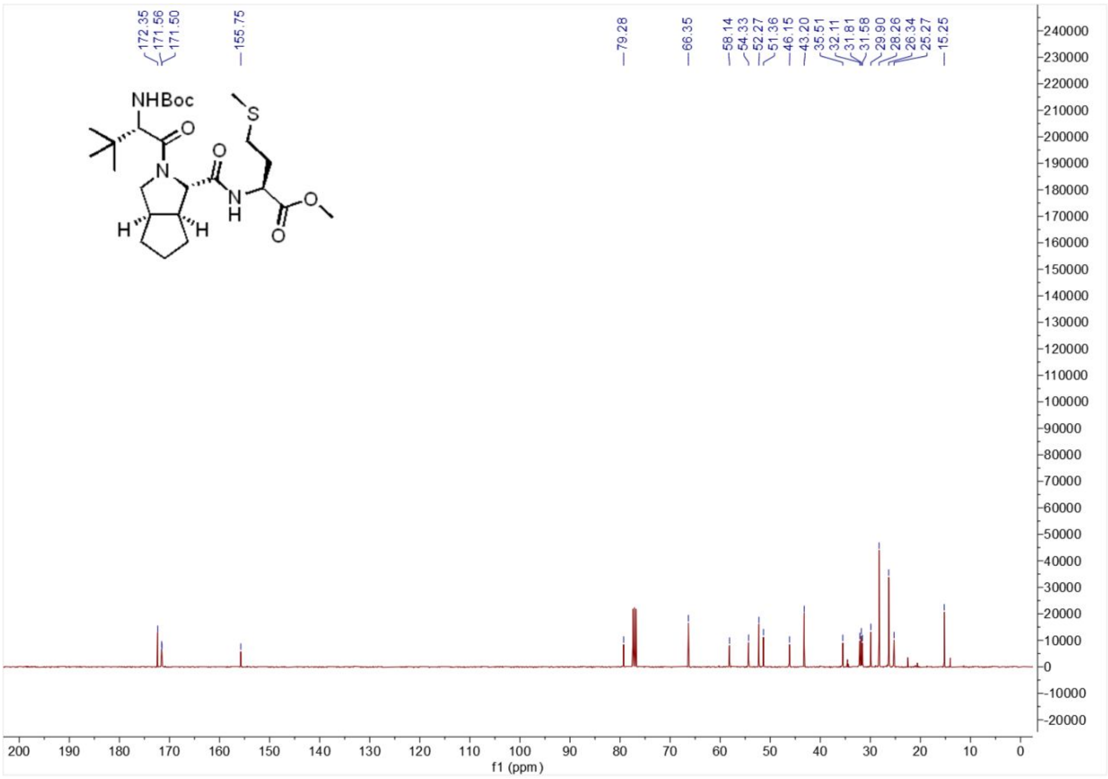

^{13}C NMR spectra of **5d**

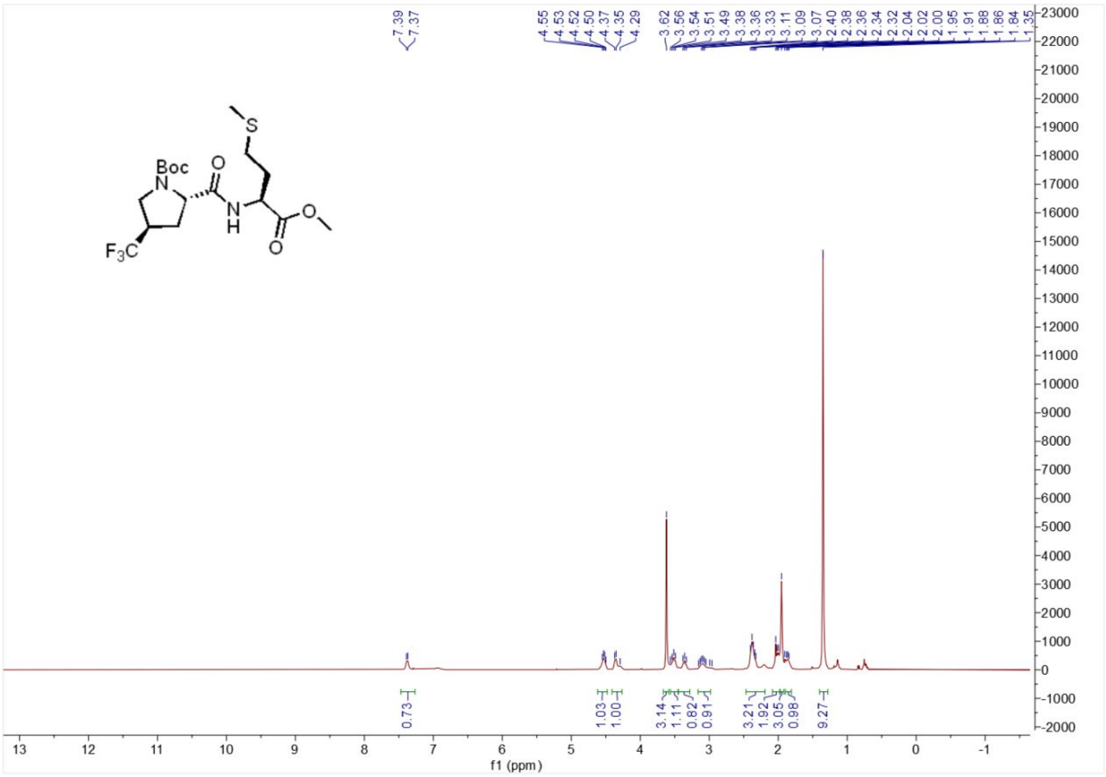

^{1}H NMR spectra of **7**

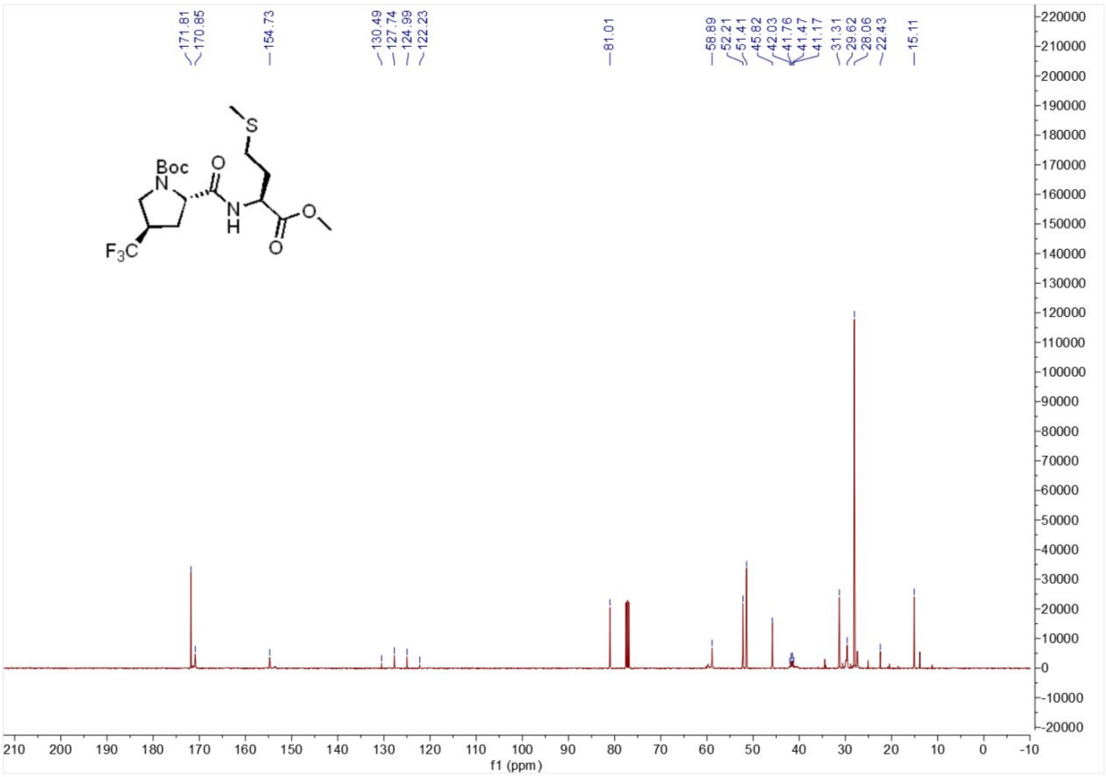

^{13}C NMR spectra of **7**

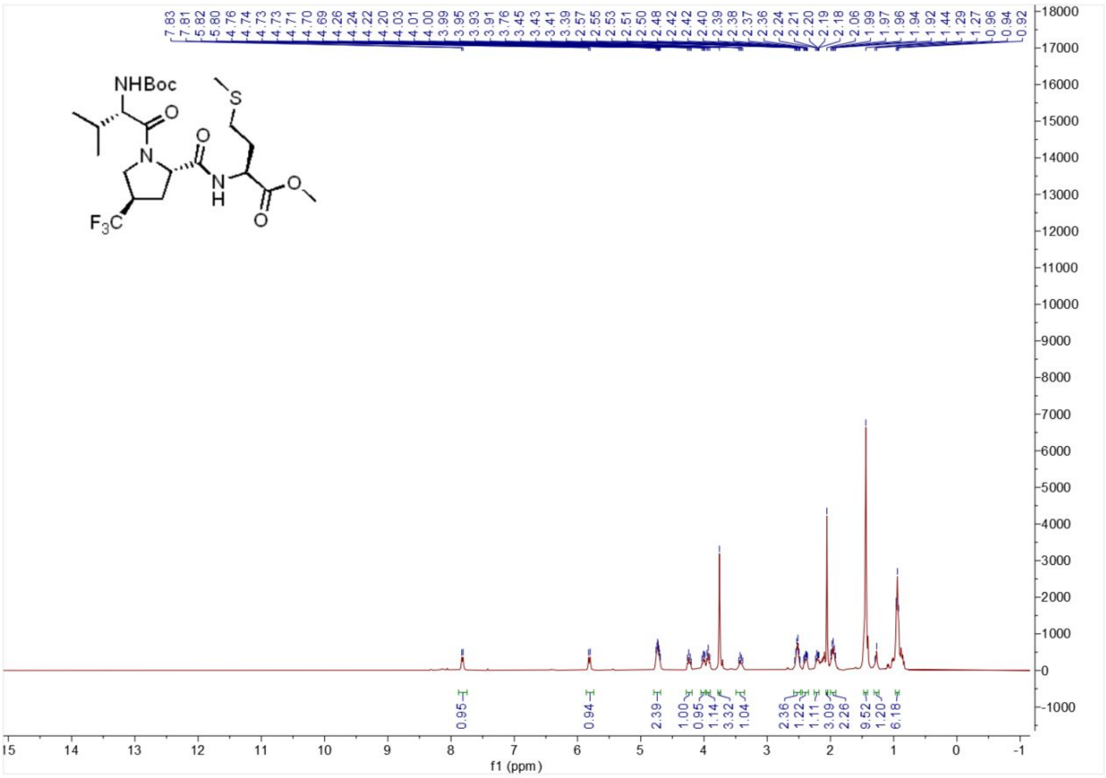

^{1}H NMR spectra of **8**

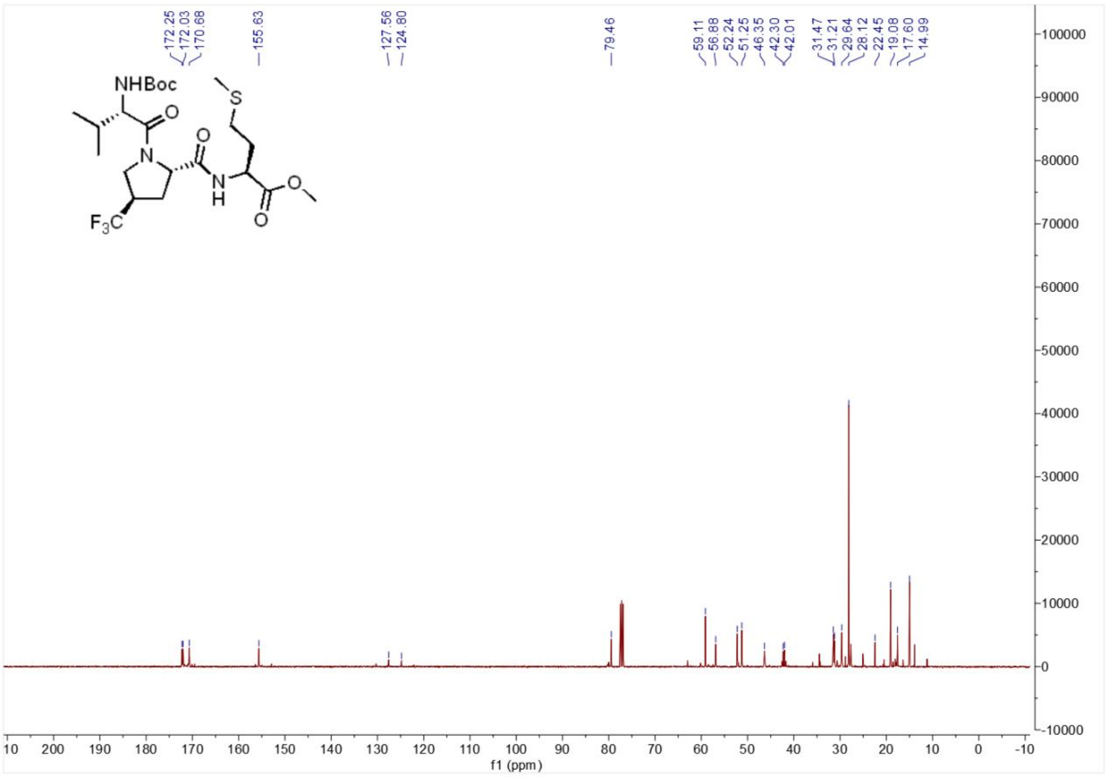

^{13}C NMR spectra of **8**

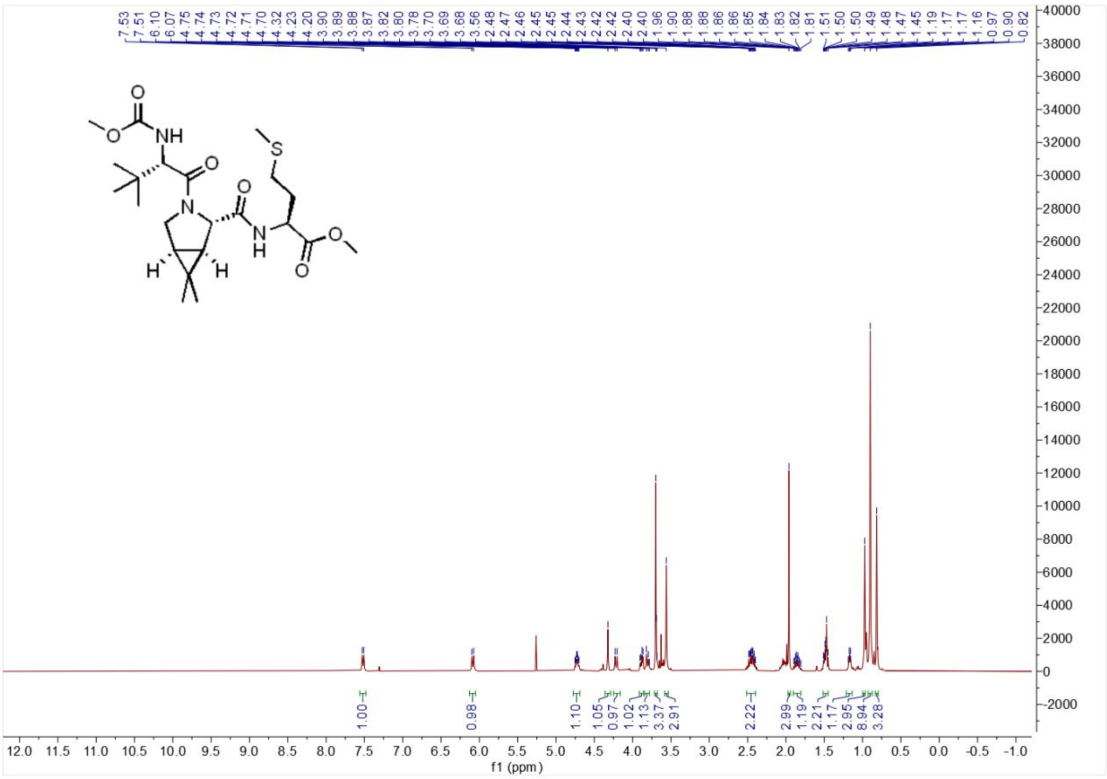

^{1}H NMR spectra of **12a**

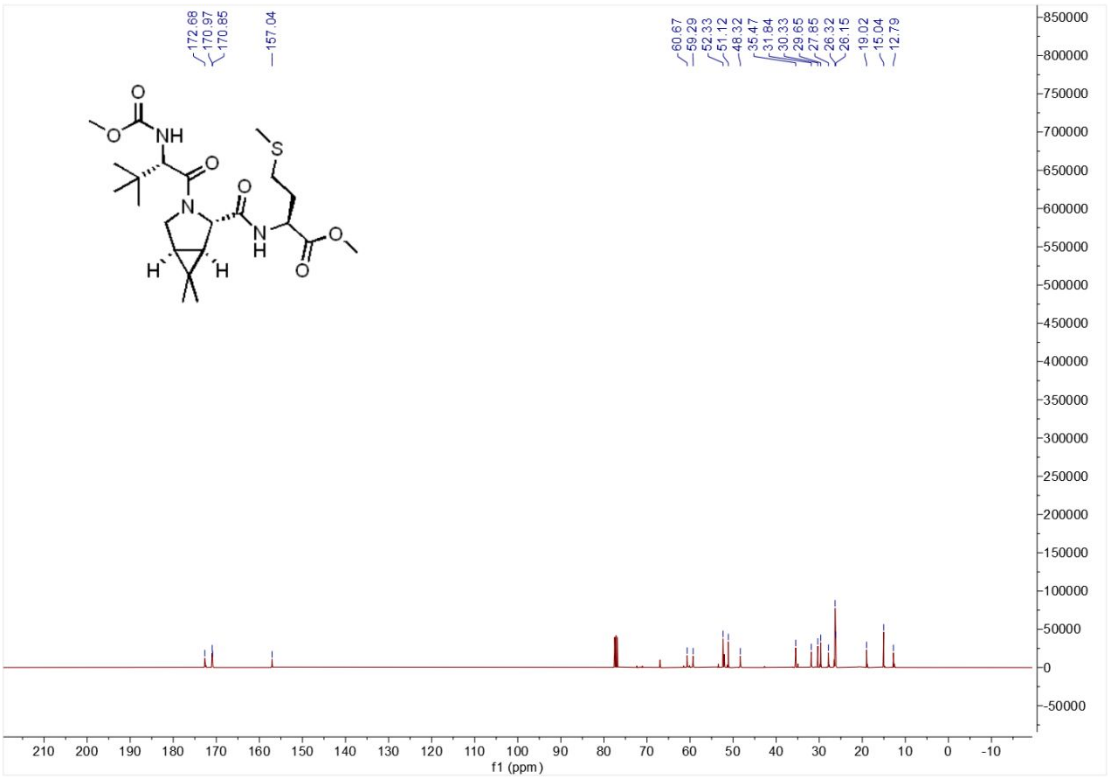

^{13}C NMR spectra of **12a**

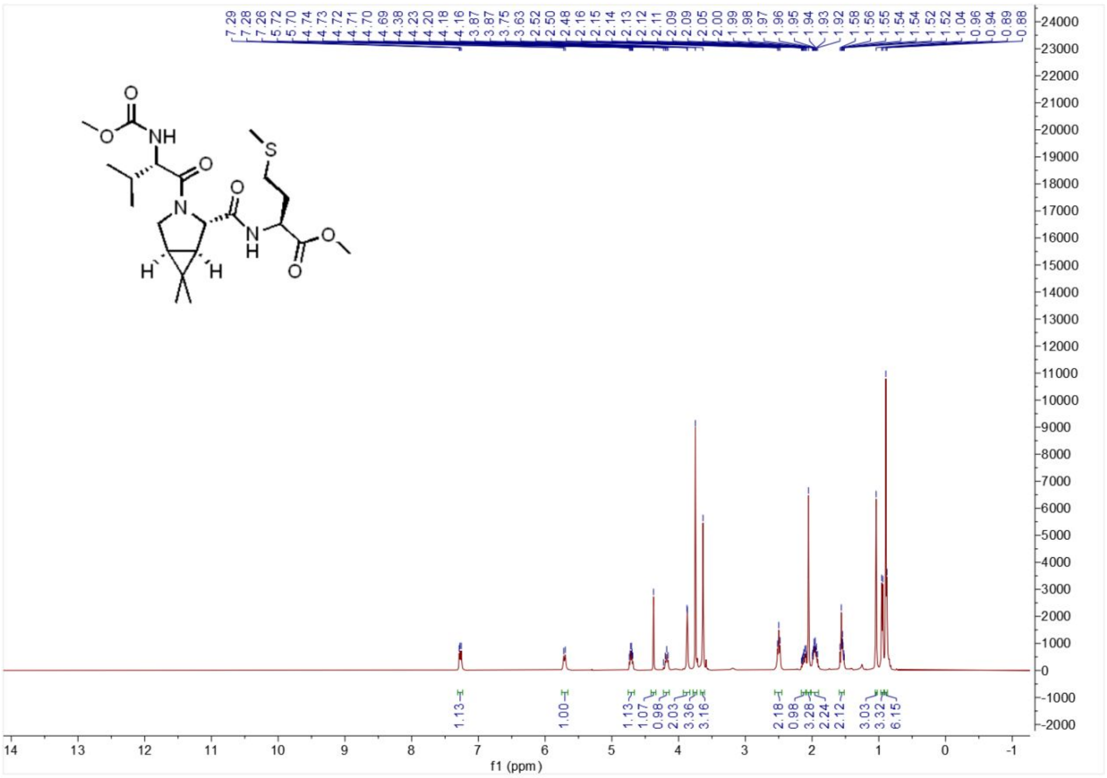

^{1}H NMR spectra of **12b**

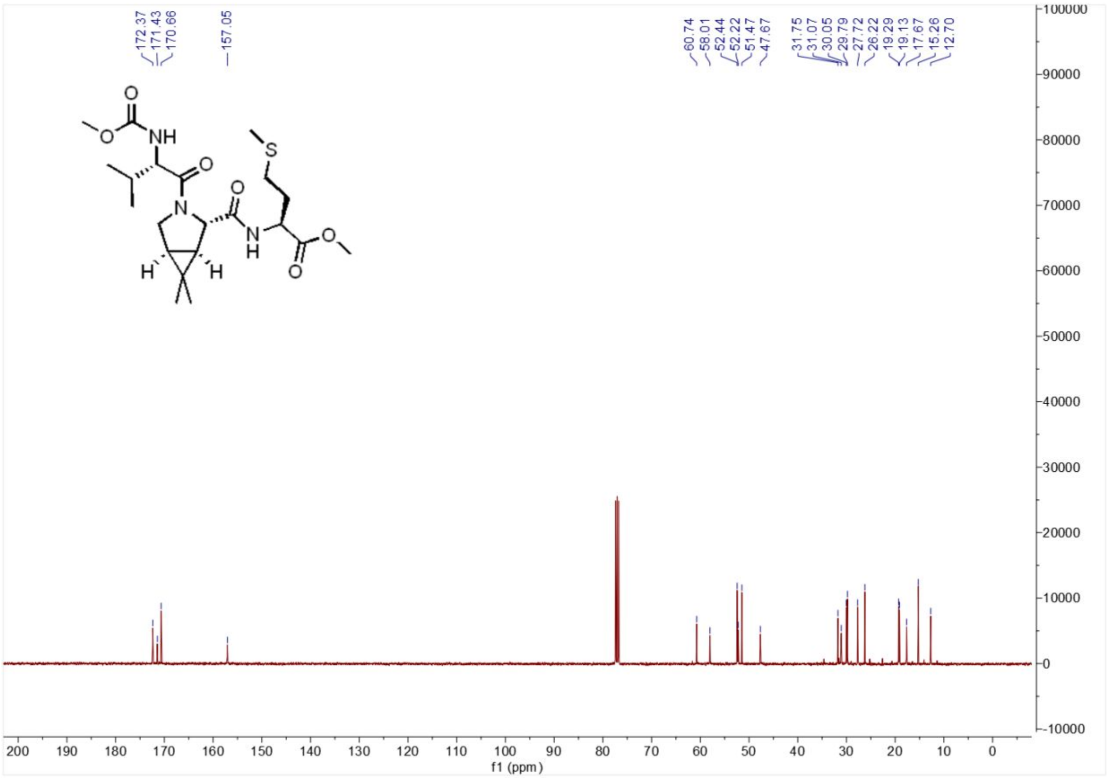

^{13}C NMR spectra of **12b**

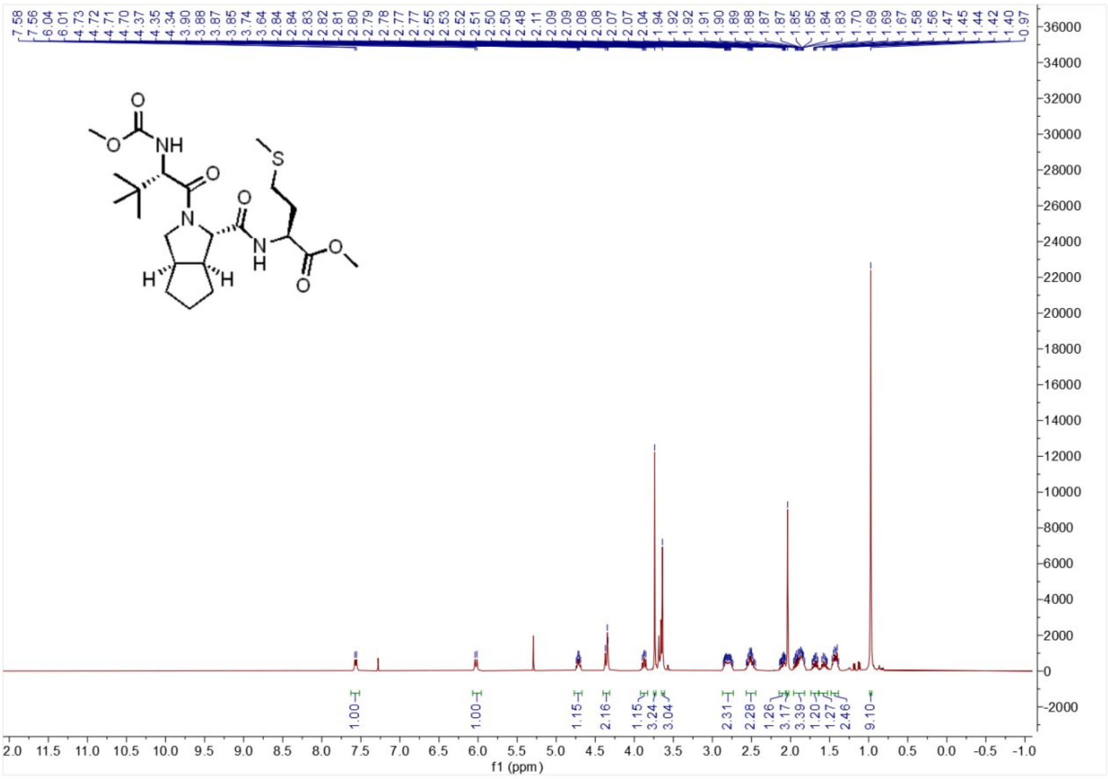

^{1}H NMR spectra of **12c**

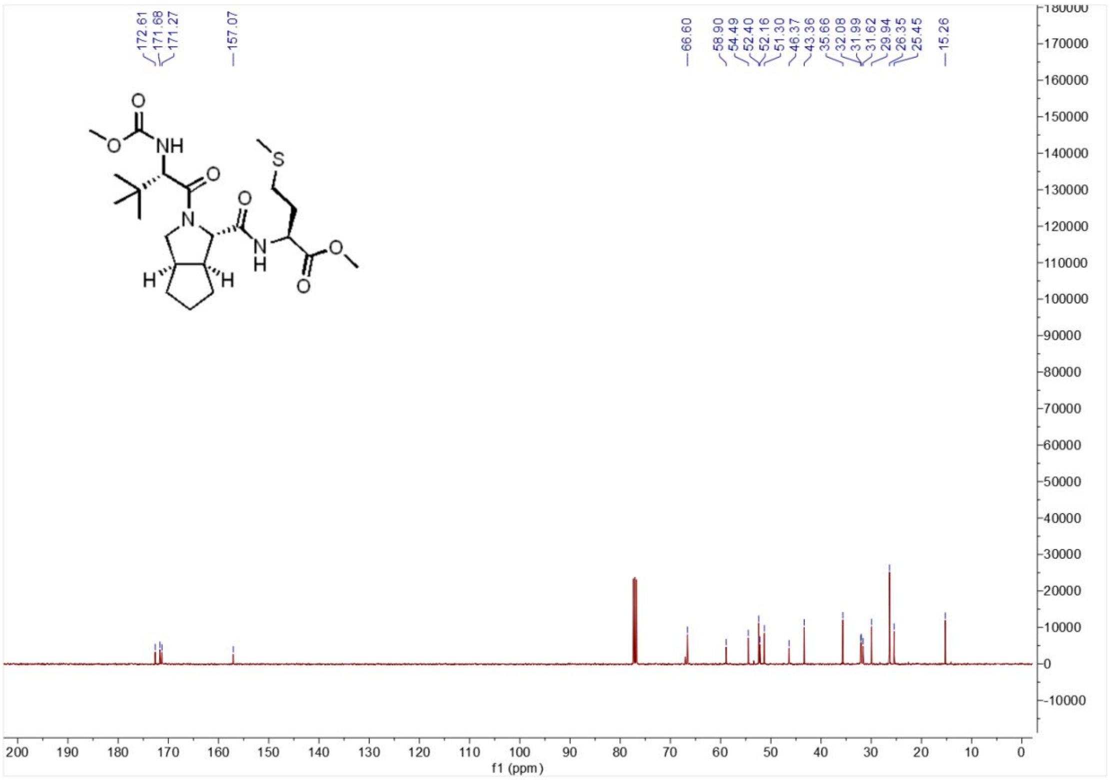

^{13}C NMR spectra of **12c**

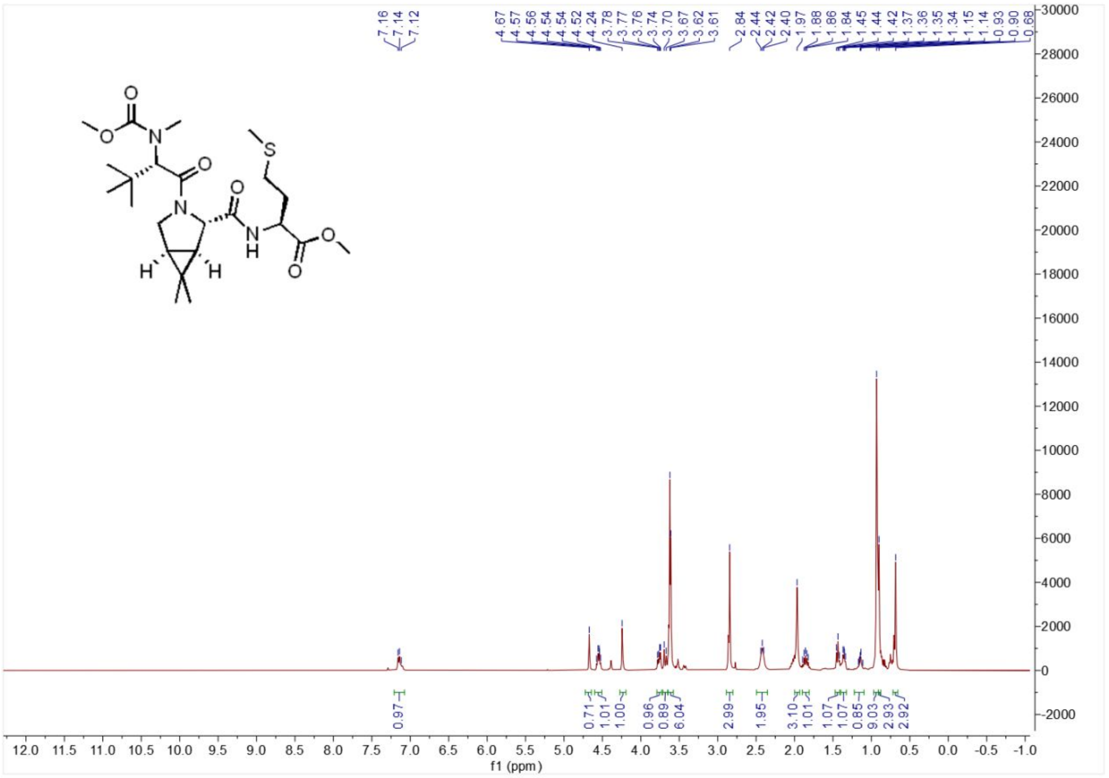

^{1}H NMR spectra of **12d**

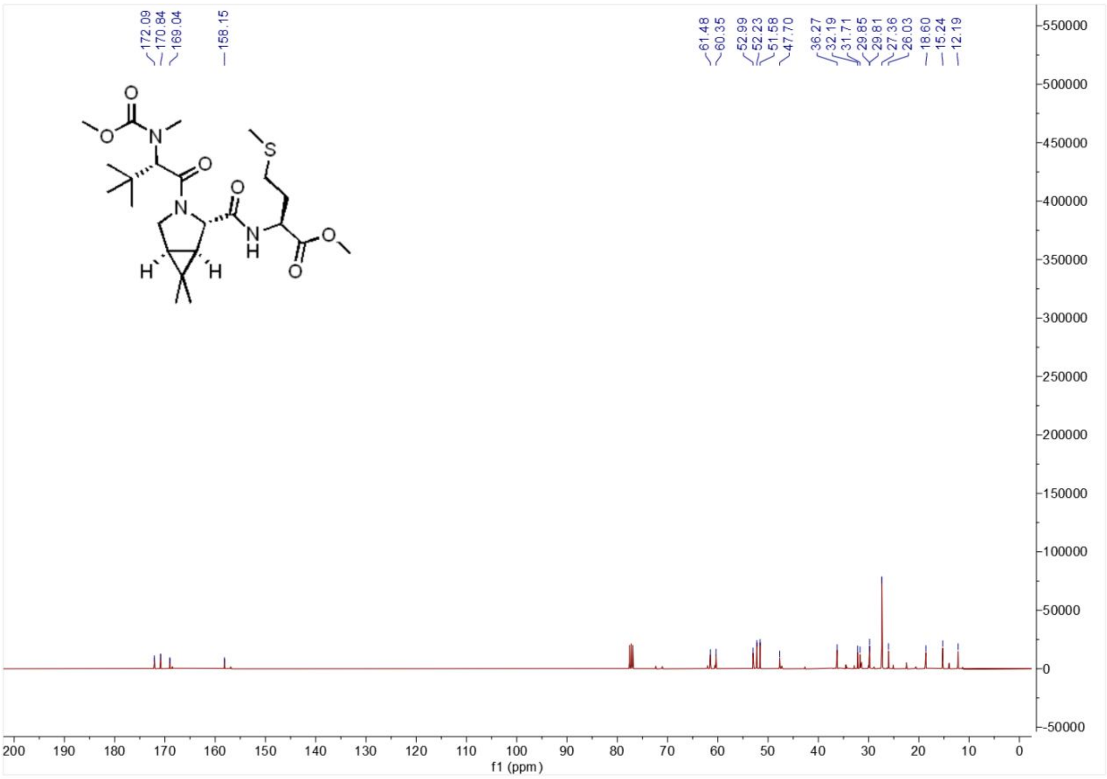

^{13}C NMR spectra of **12d**

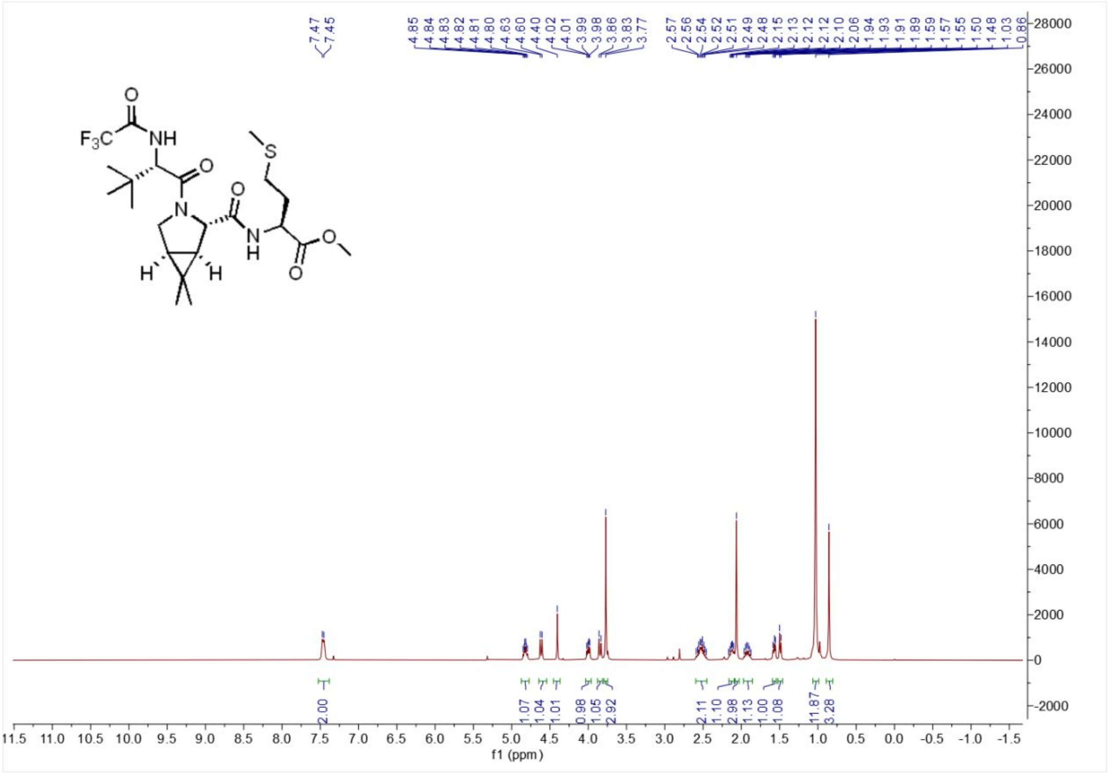

^{1}H NMR spectra of **16**

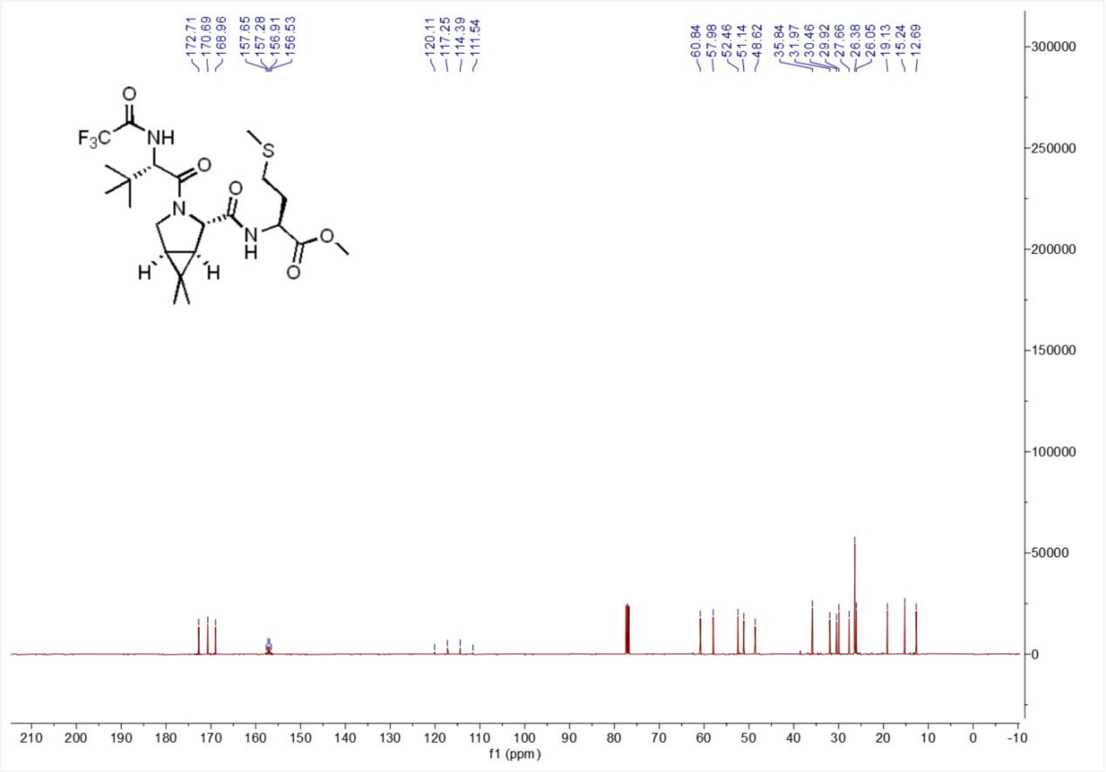

^{13}C NMR spectra of **16**

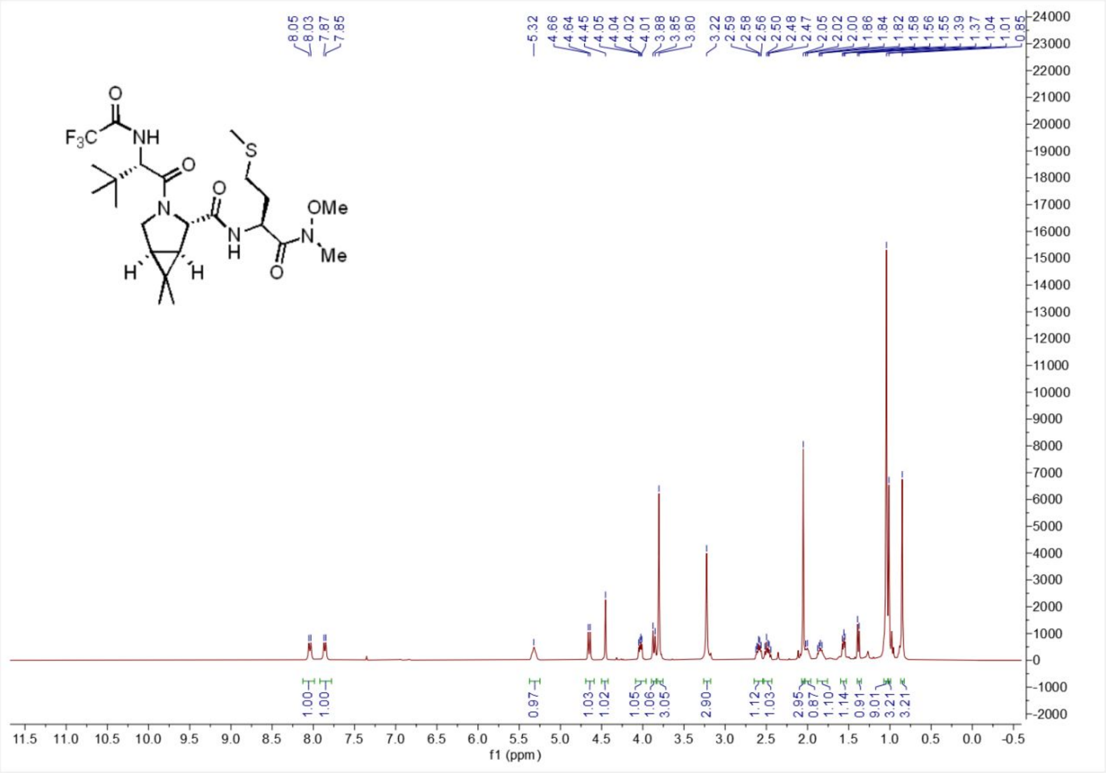

^{1}H NMR spectra of **18**

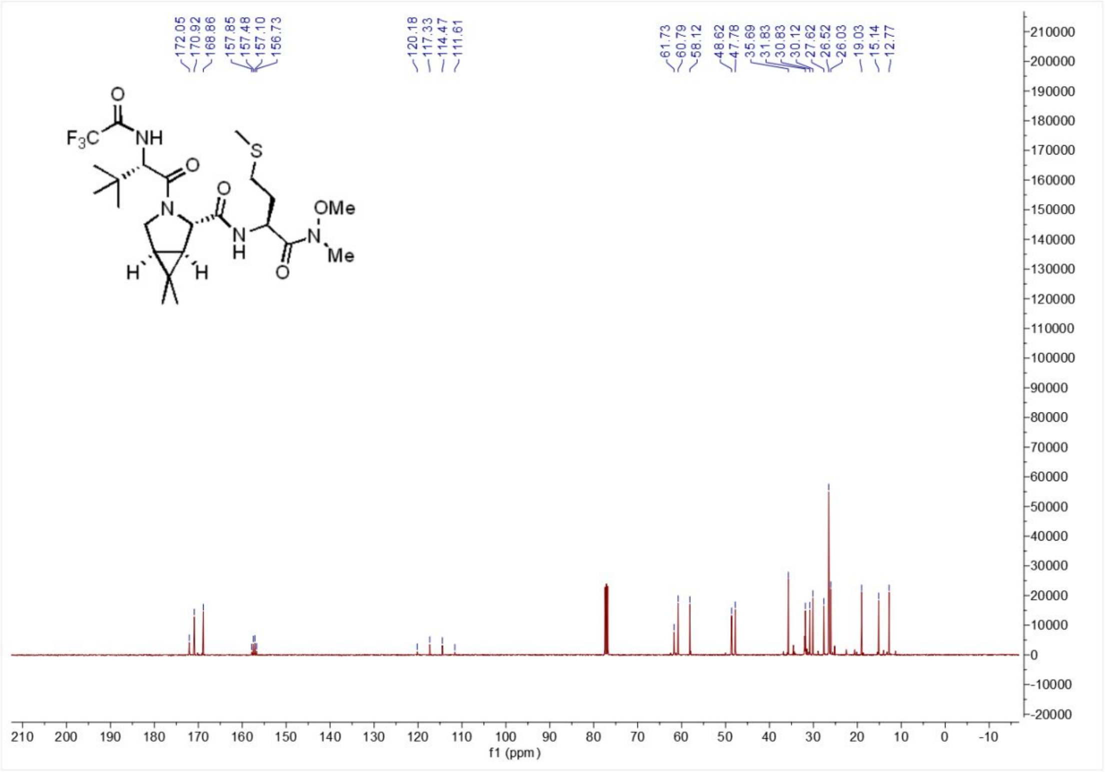

^{13}C NMR spectra of **18**

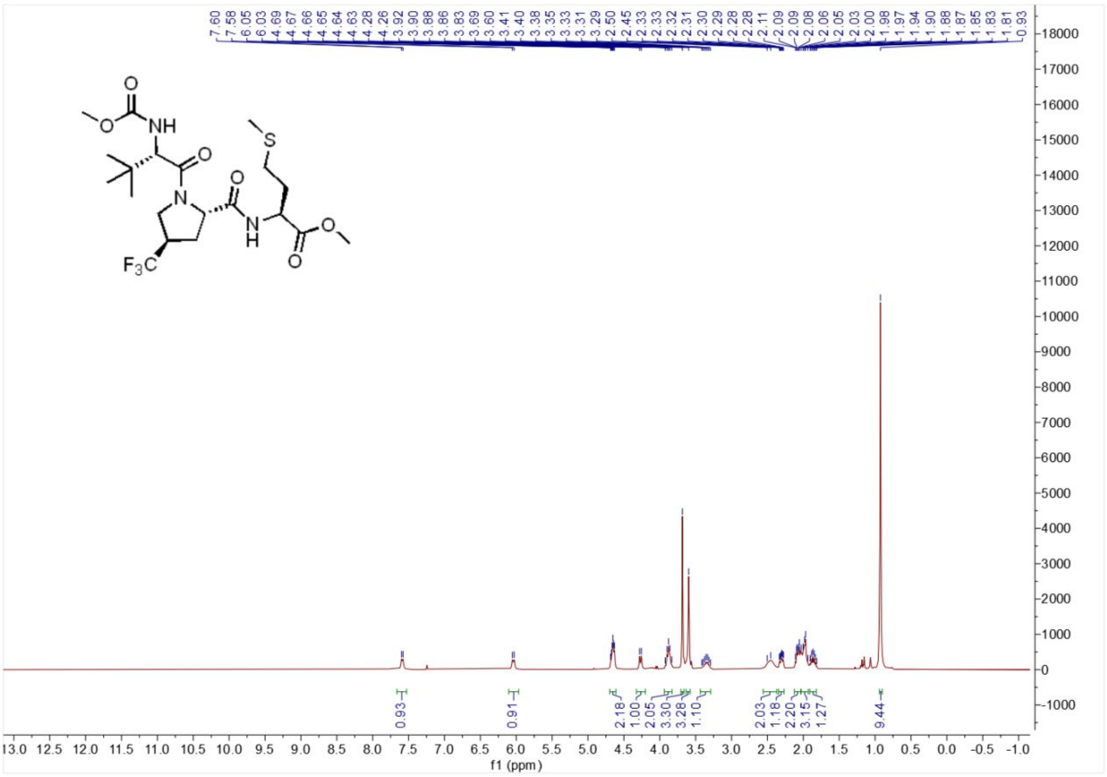

^{1}H NMR spectra of **22a**

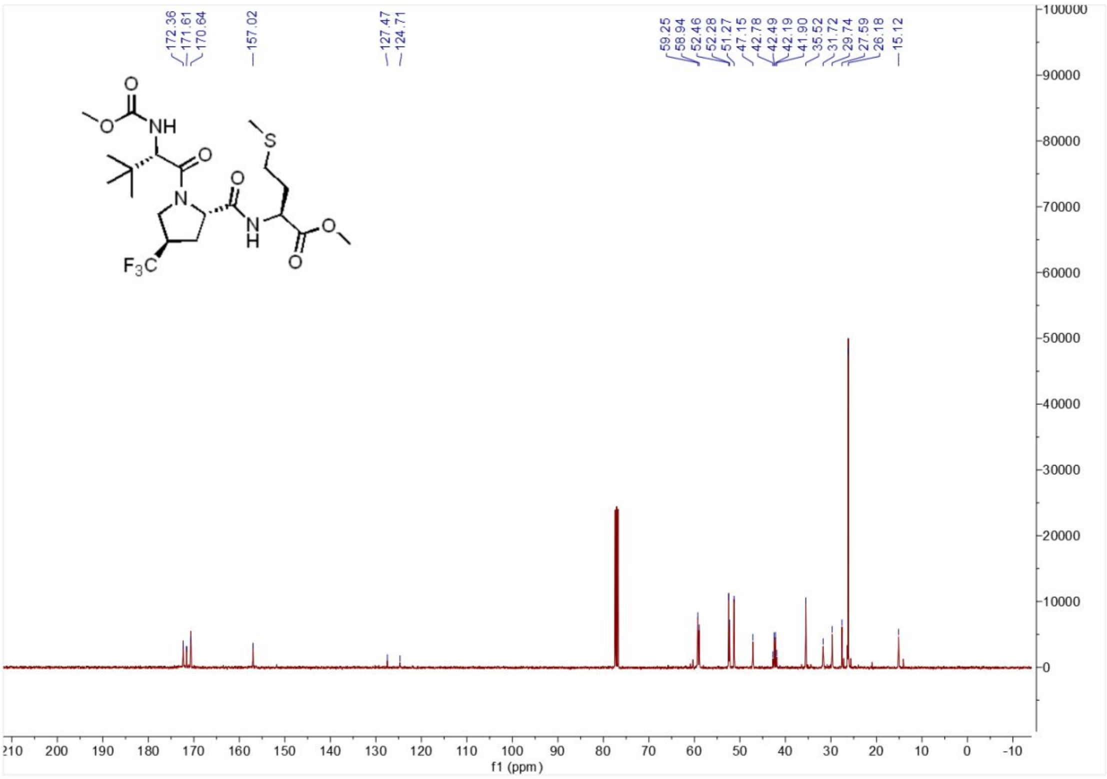

^{13}C NMR spectra of **22a**

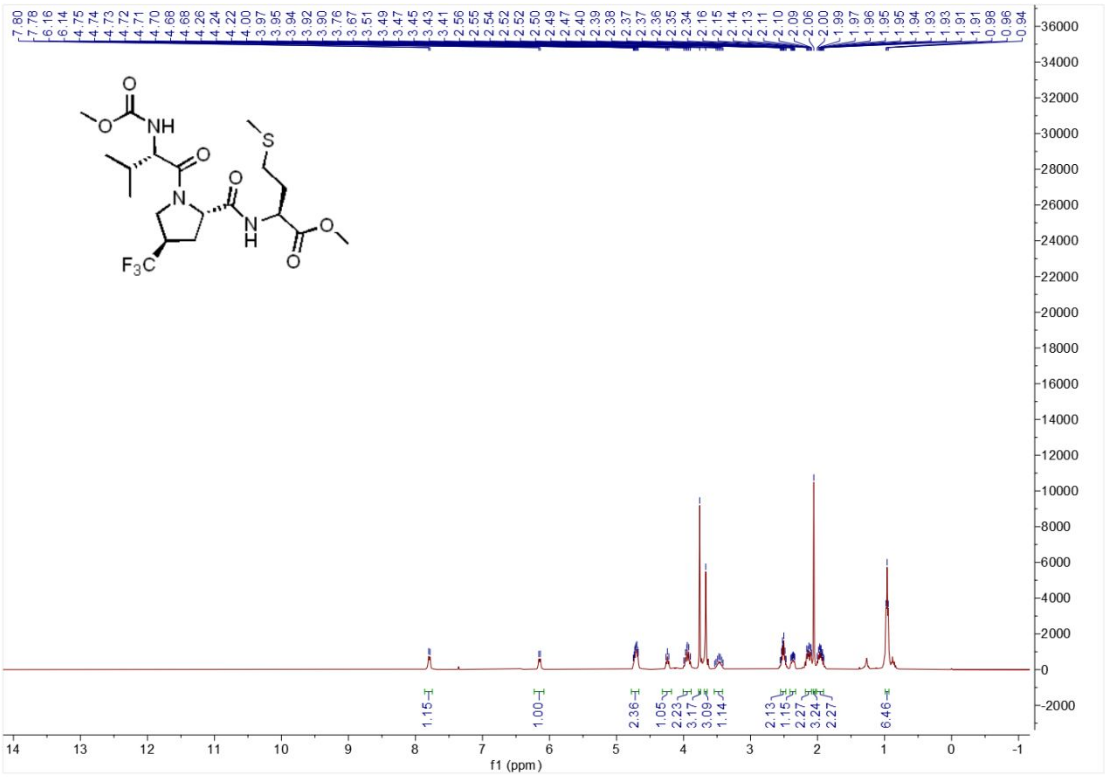

^{1}H NMR spectra of **22b**

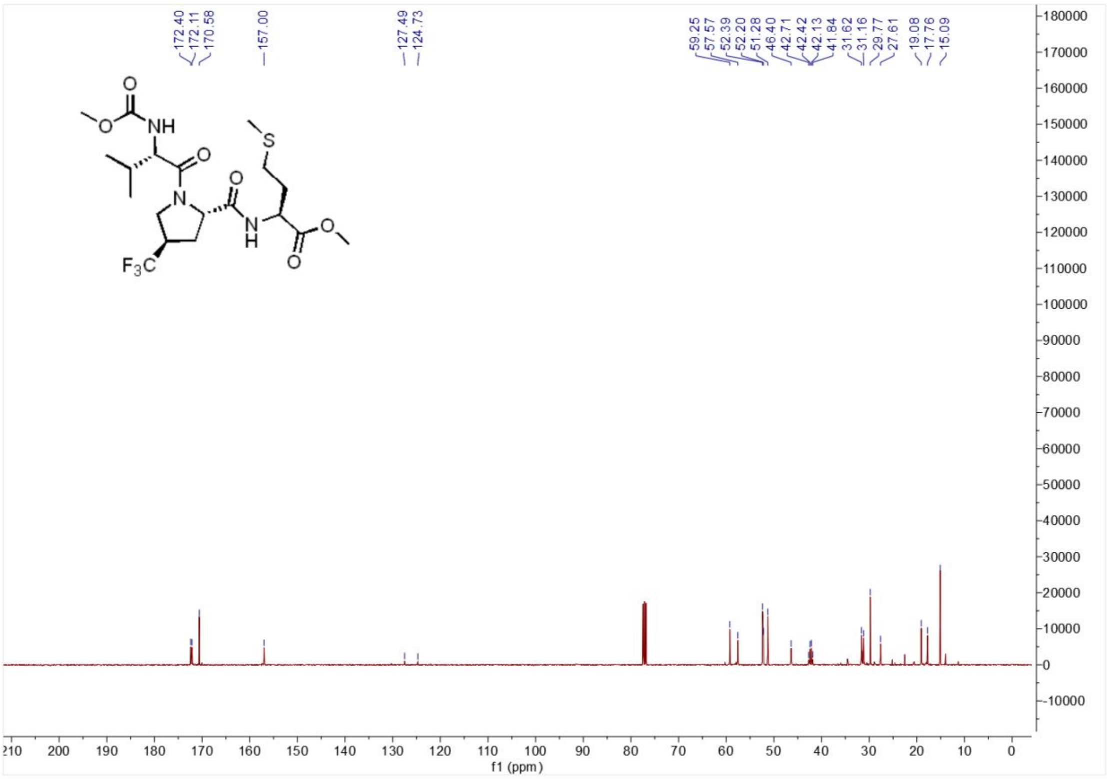

^{13}C NMR spectra of **22b**

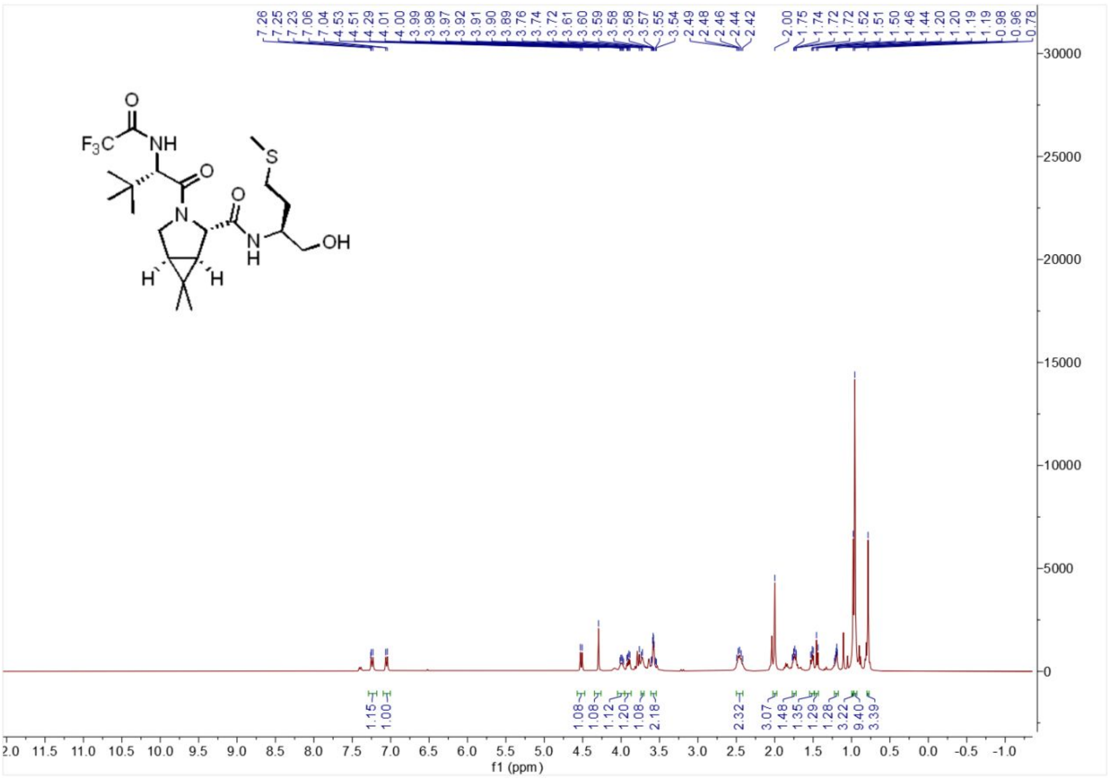

^{1}H NMR spectra of **10a**

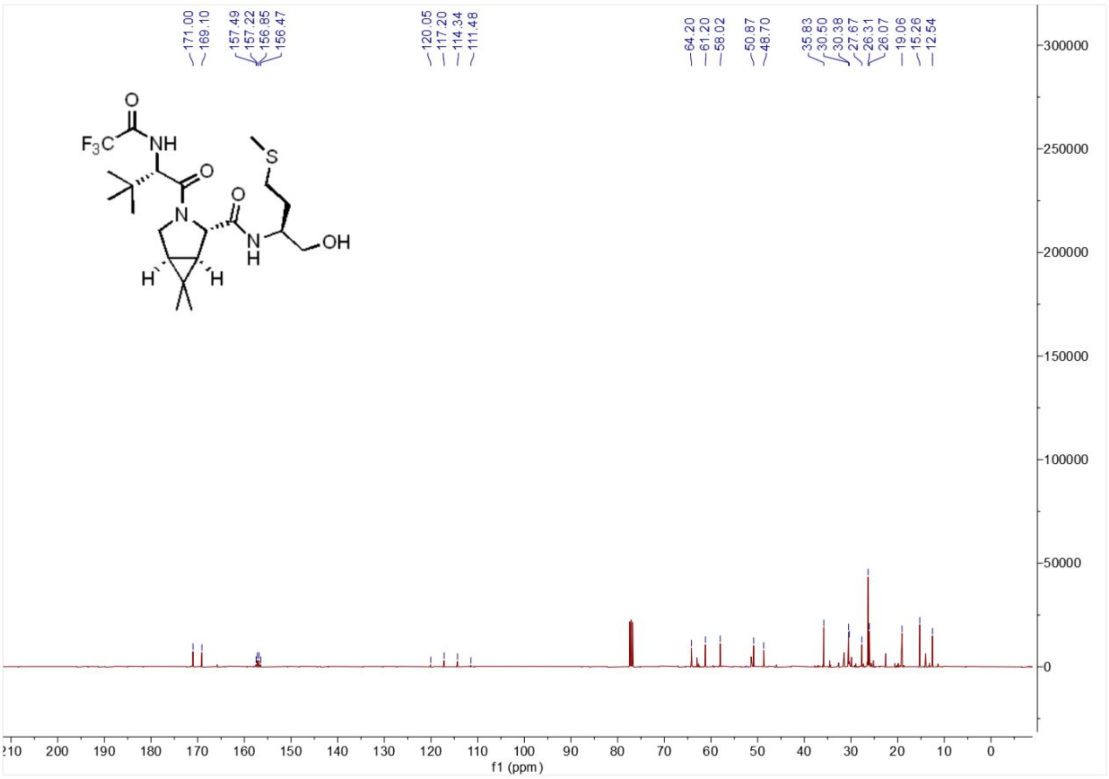

^{13}C NMR spectra of **10a**

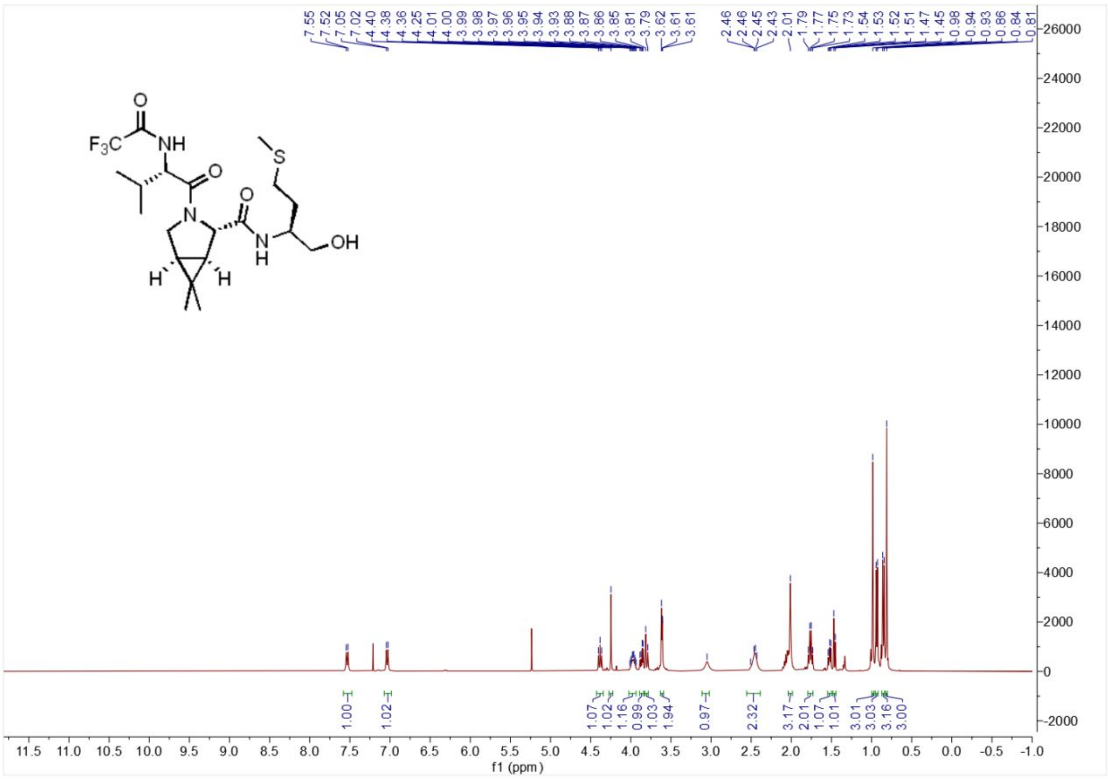

^{1}H NMR spectra of **10b**

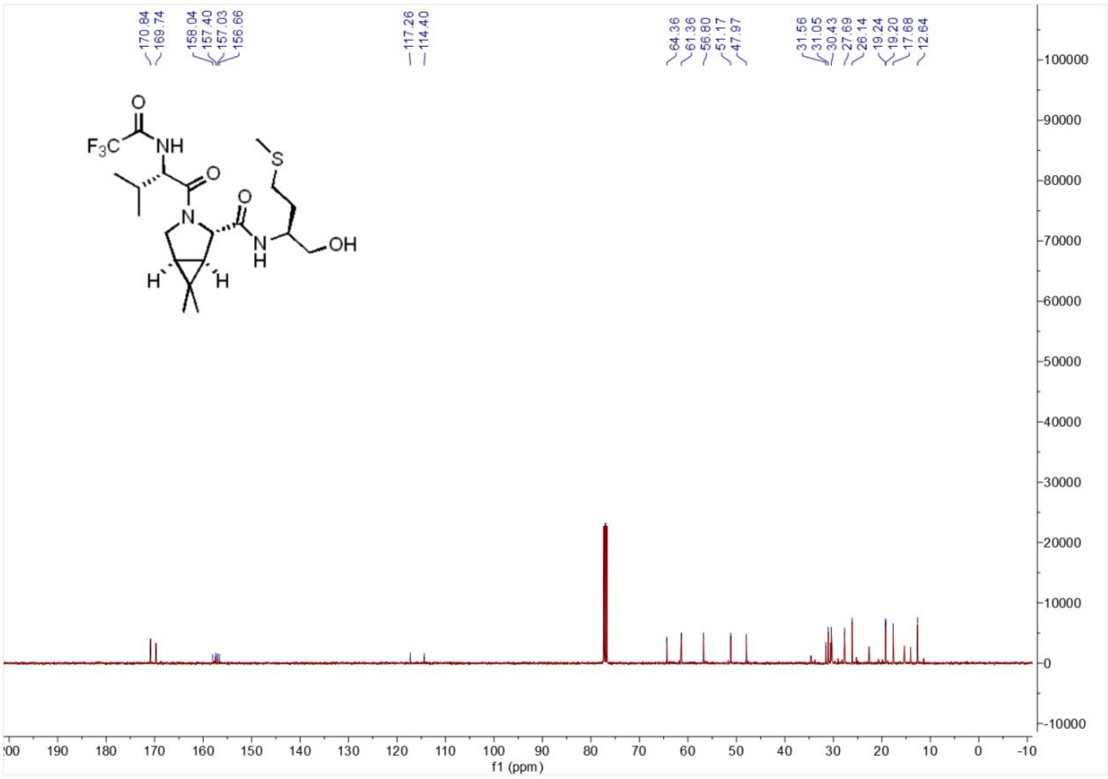

^{13}C NMR spectra of **10b**

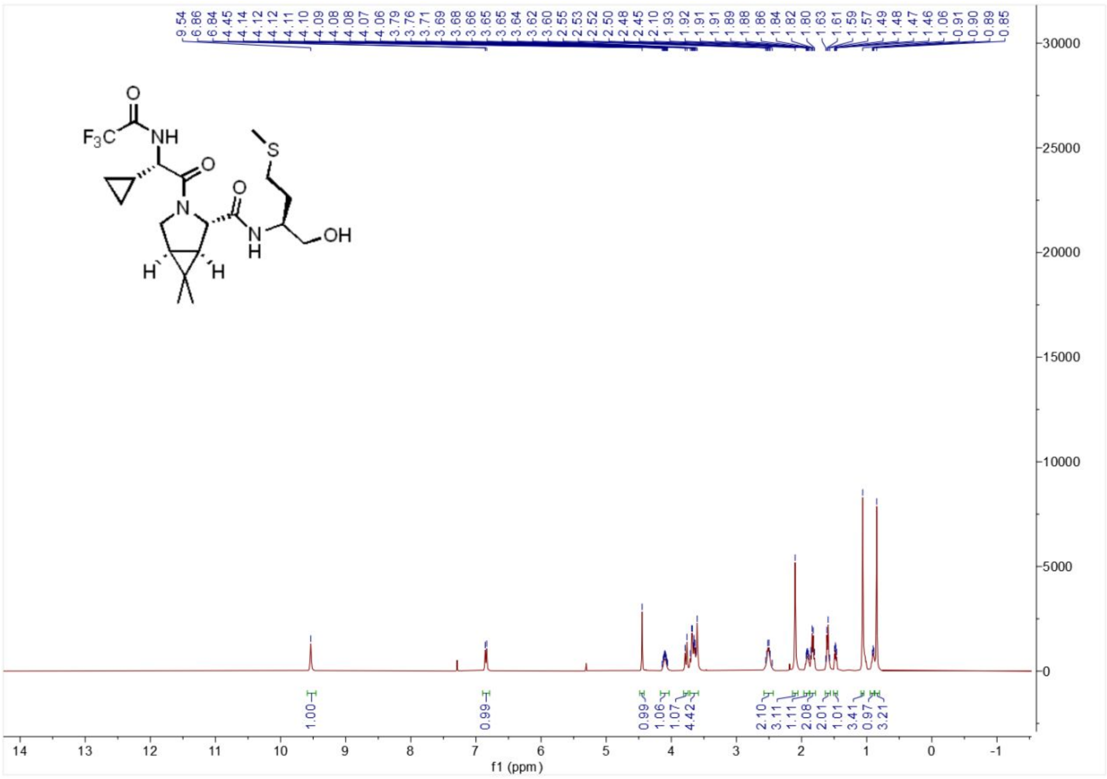

^{1}H NMR spectra of **10c**

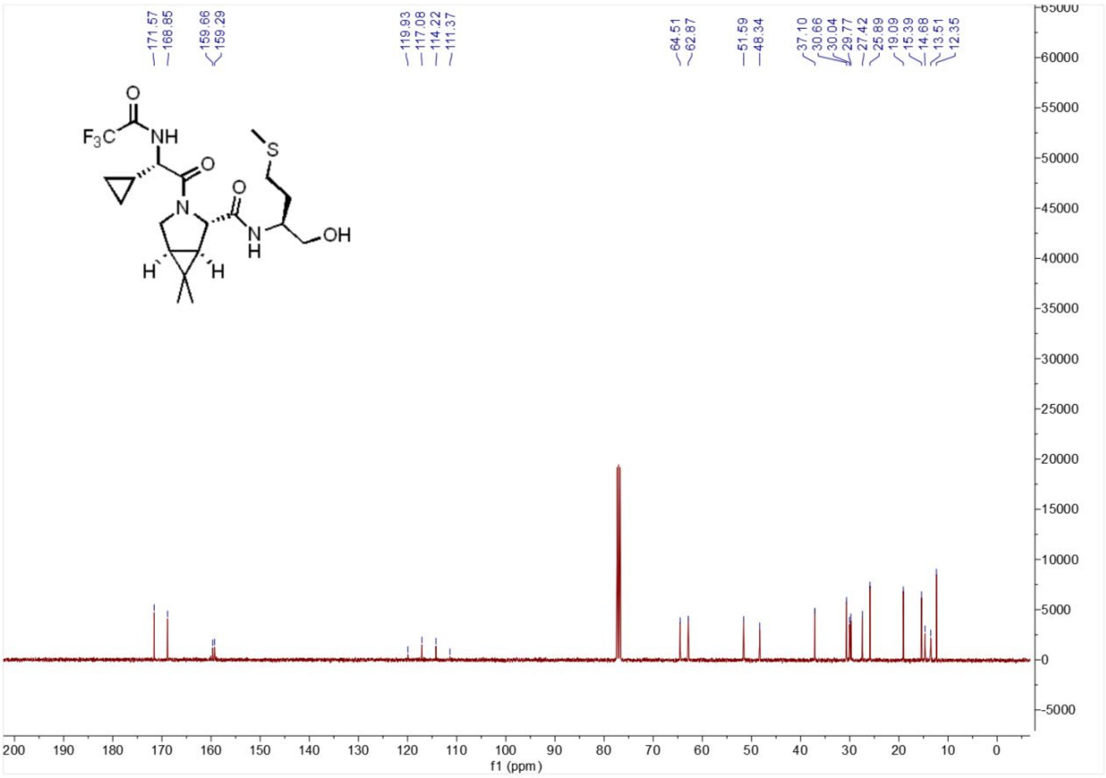

^{13}C NMR spectra of **10c**

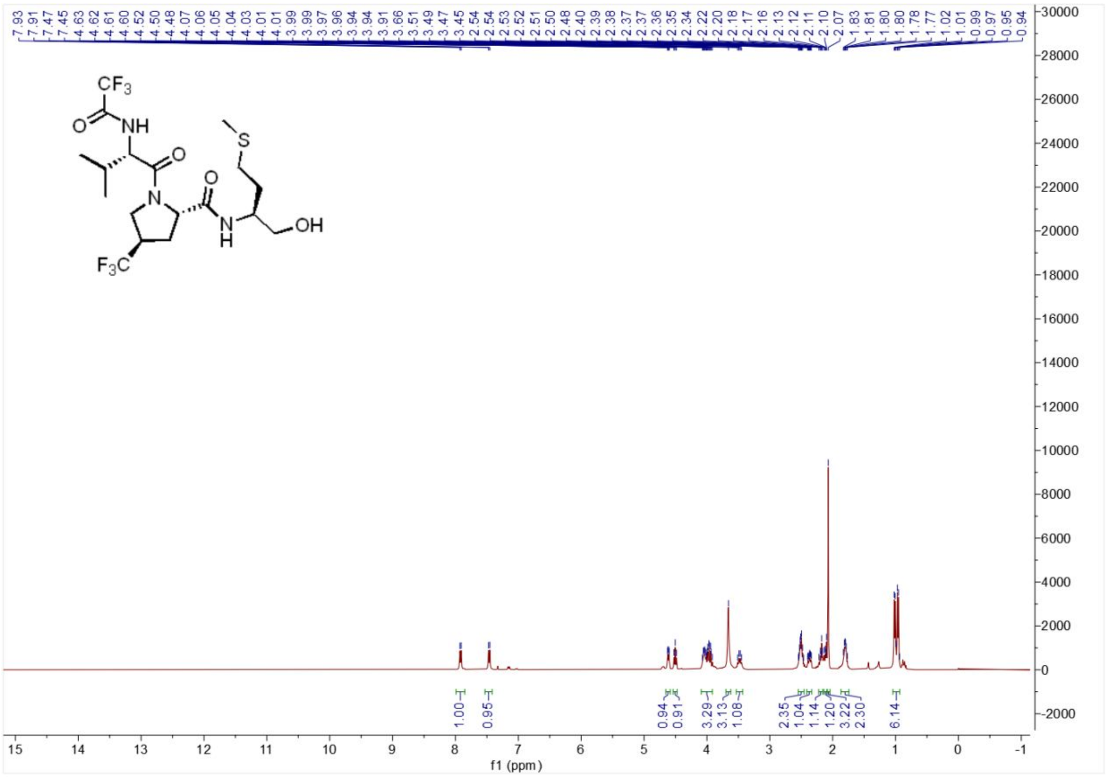

^{1}H NMR spectra of **10d**

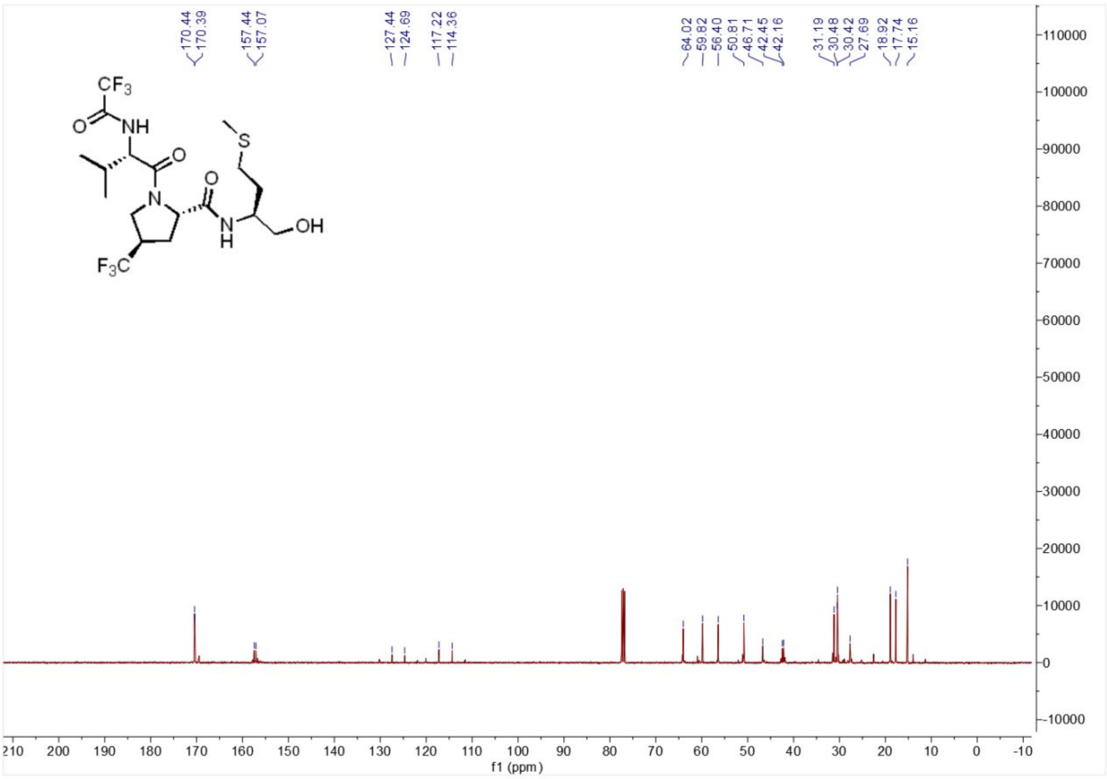

^{13}C NMR spectra of **10d**

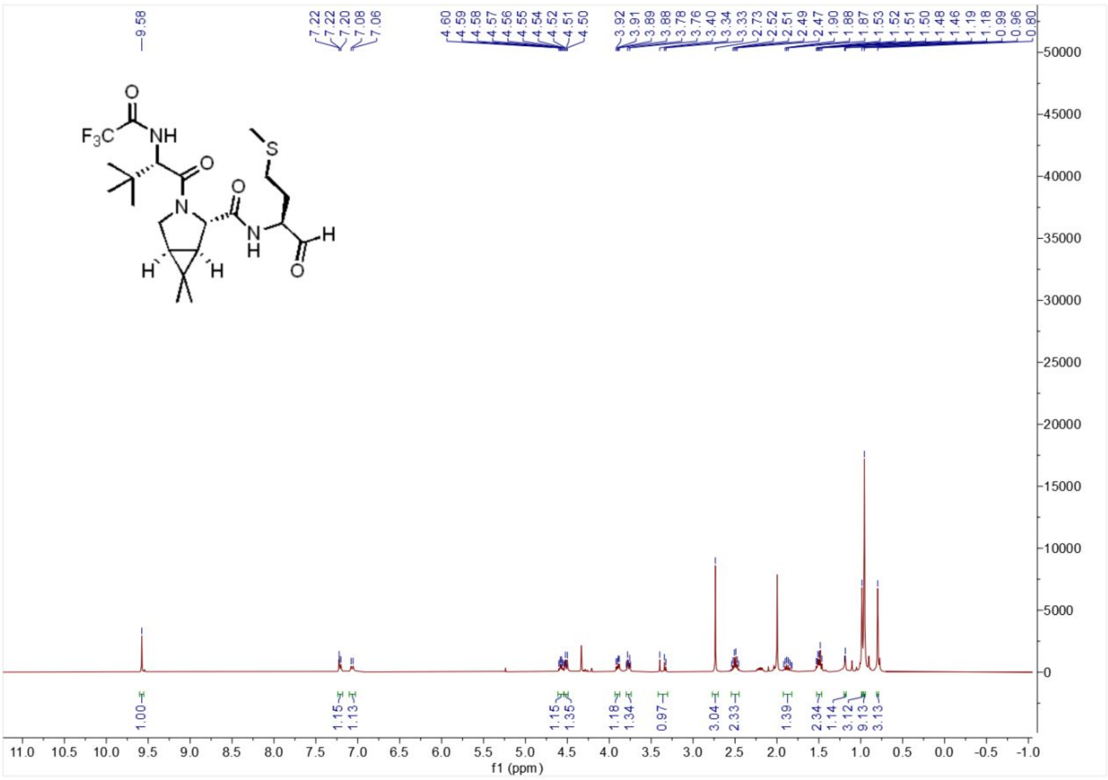

^{1}H NMR spectra of **Jun12504**

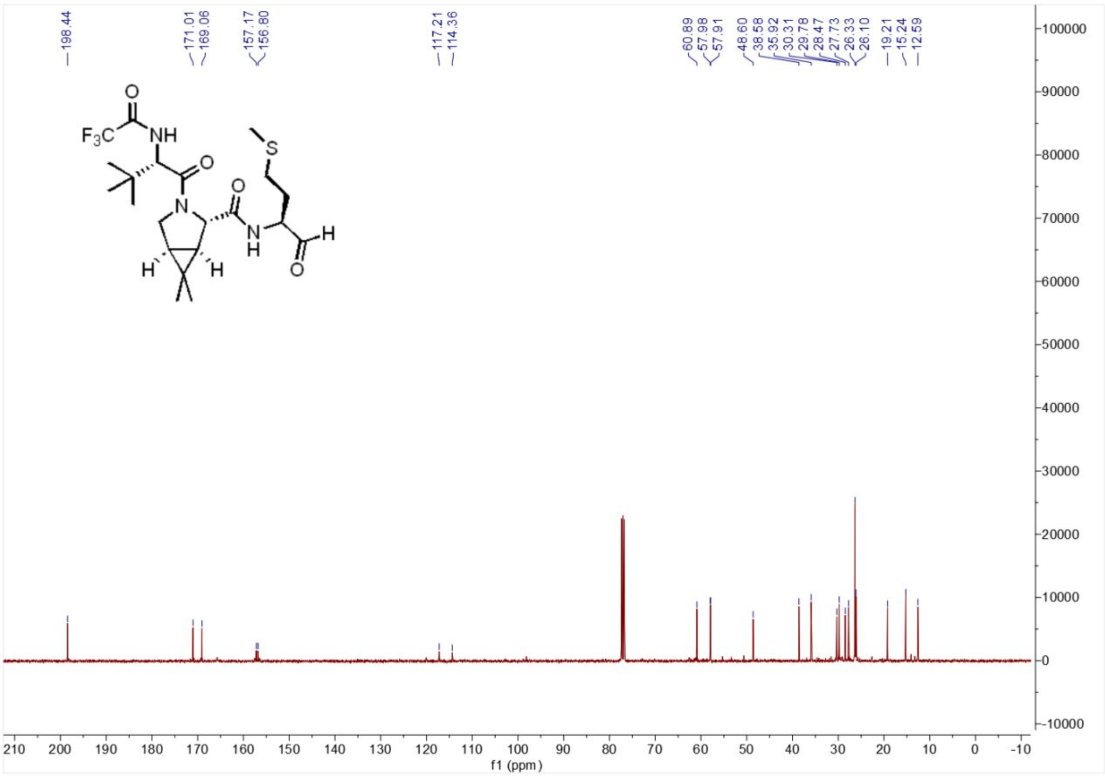

^{13}C NMR spectra of **Jun12504**

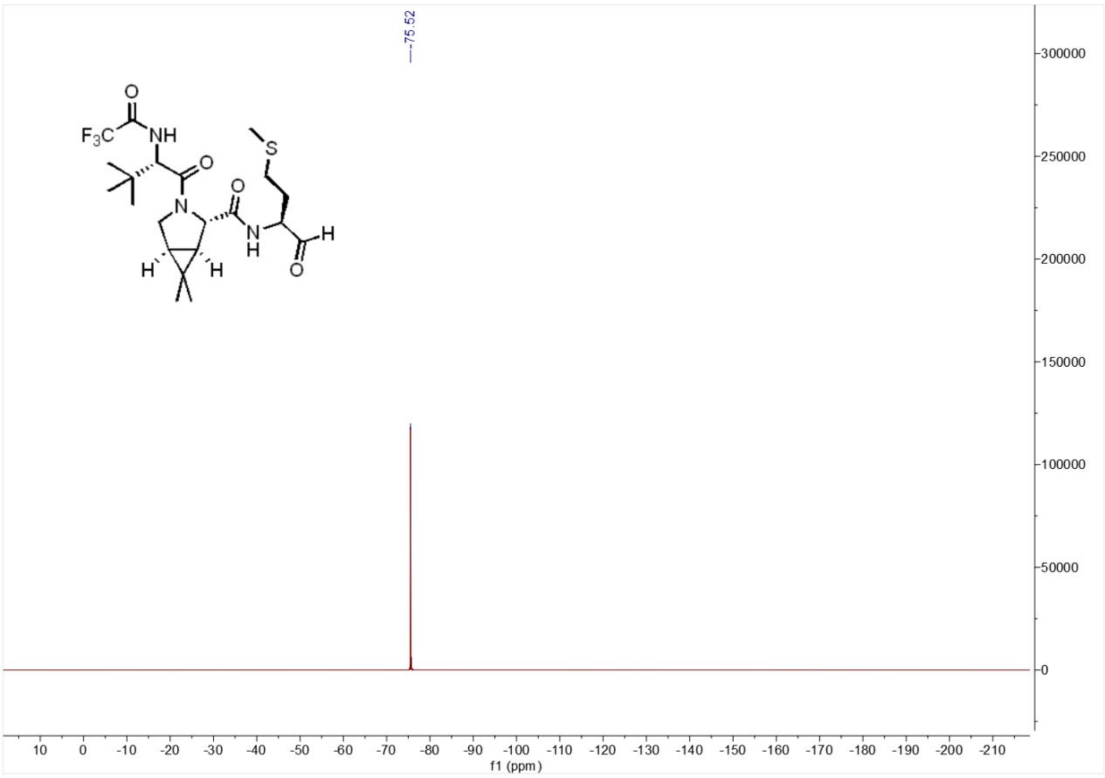

^{19}F NMR spectra of **Jun12504**

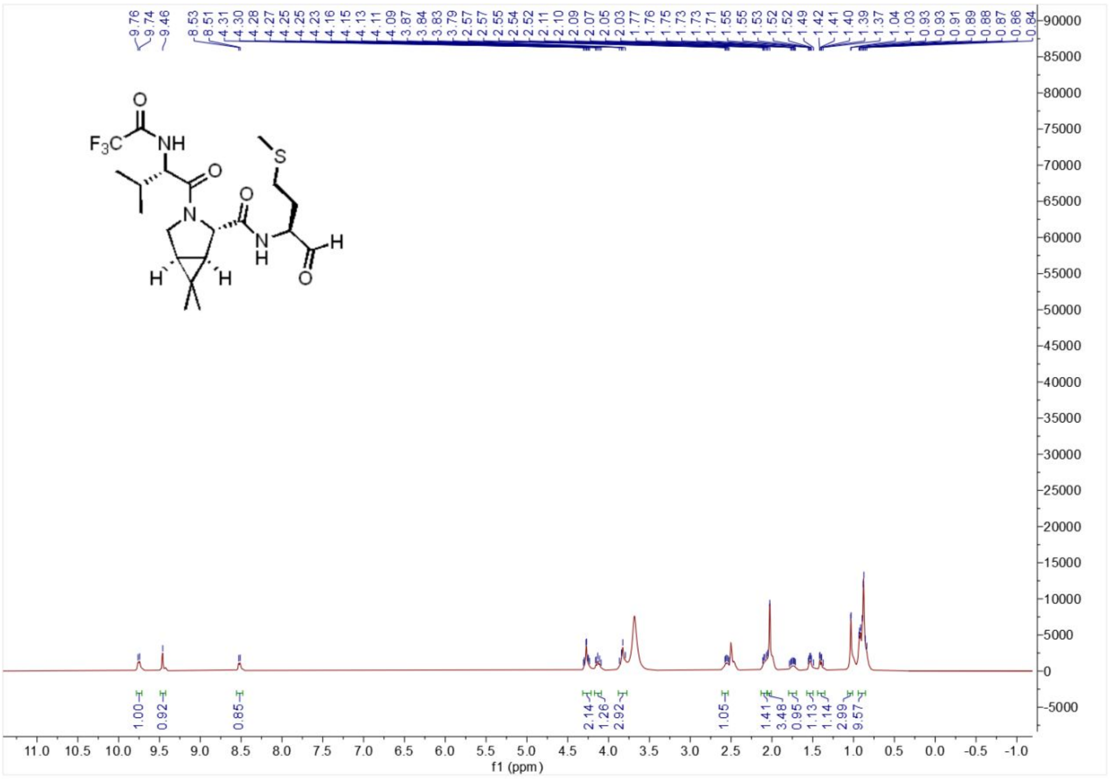

^{1}H NMR spectra of **Jun13698**

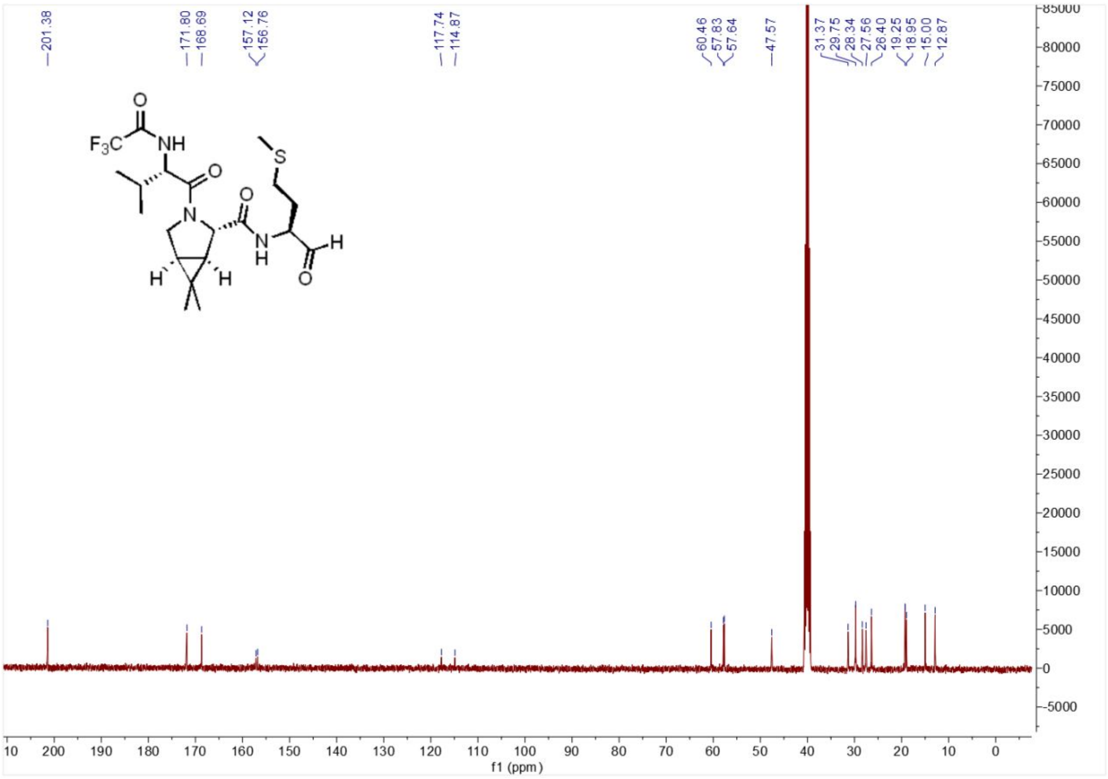

^{13}C NMR spectra of **Jun13698**

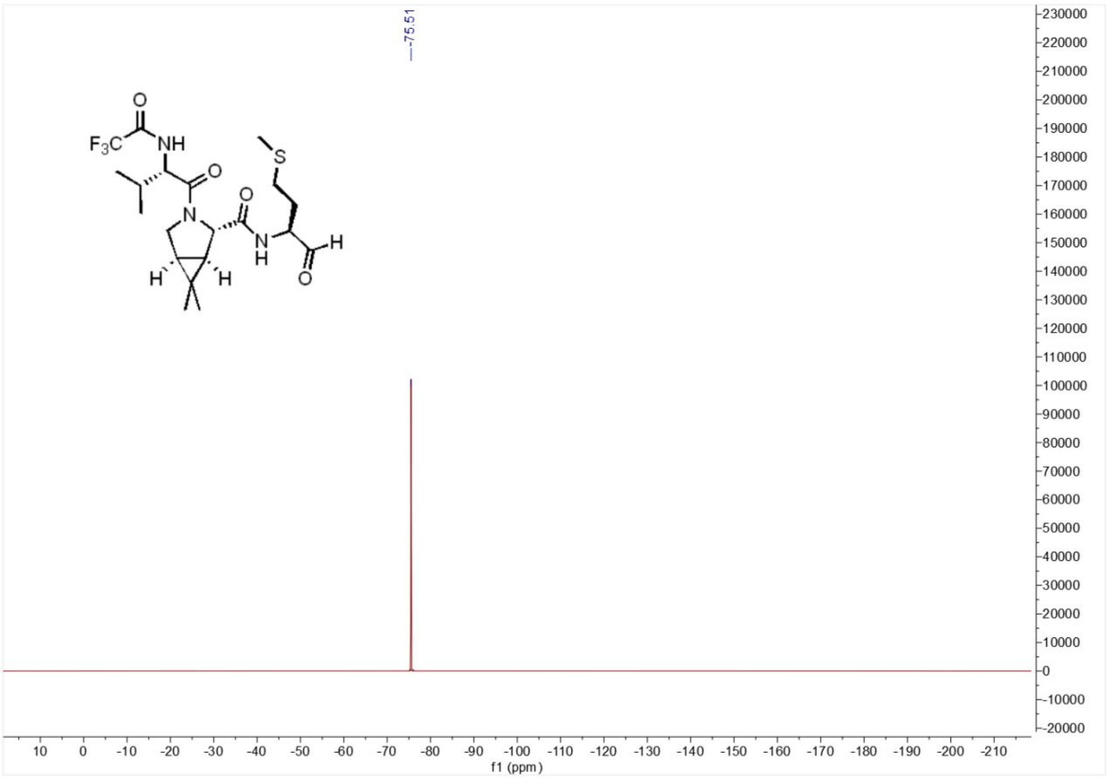

^{19}F NMR spectra of **Jun13698**

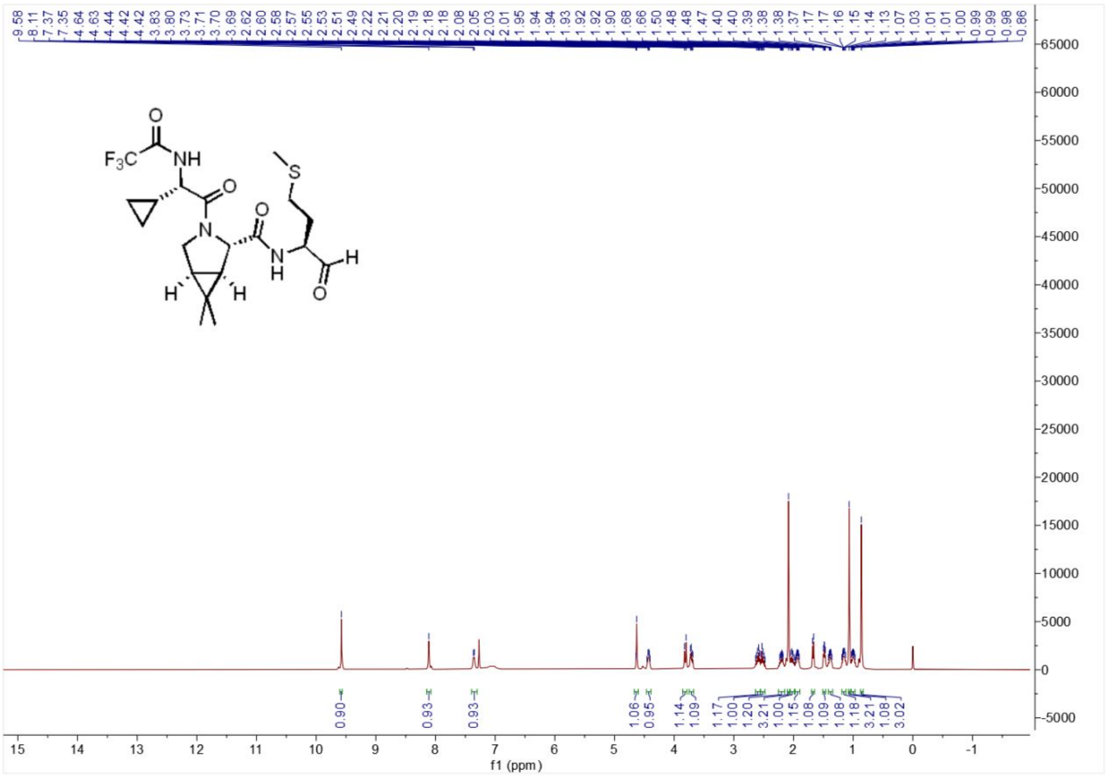

^{1}H NMR spectra of **Jun15574**

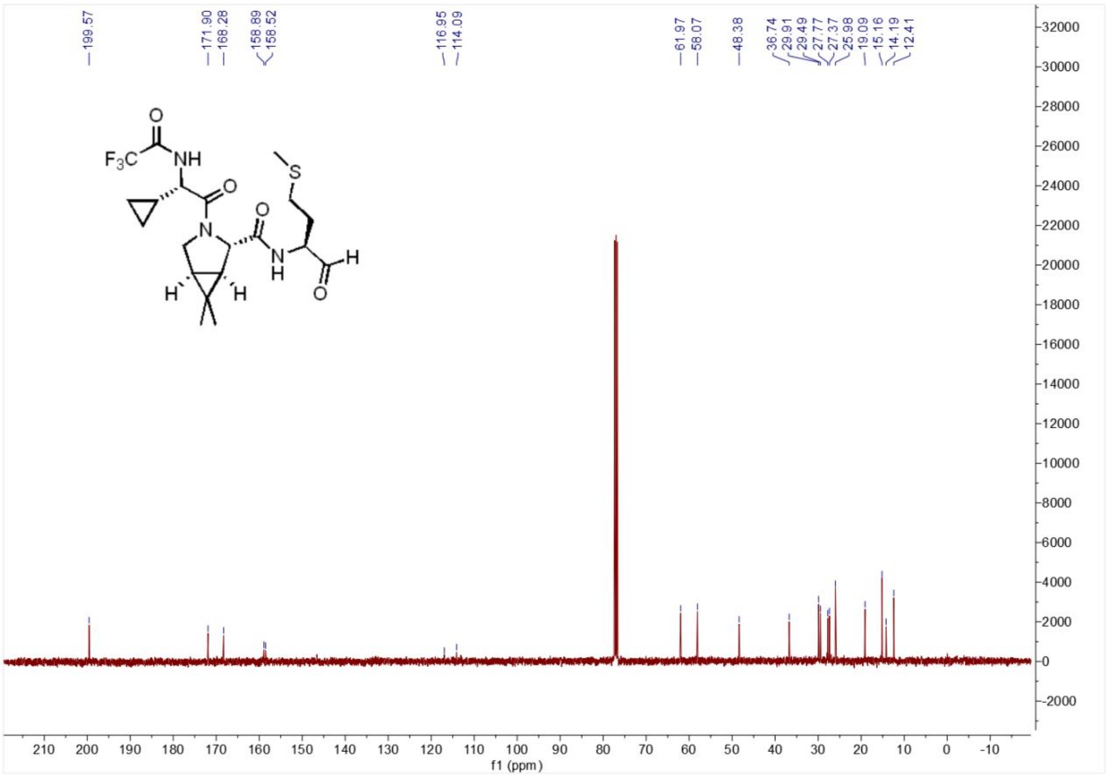

^{13}C NMR spectra of **Jun15574**

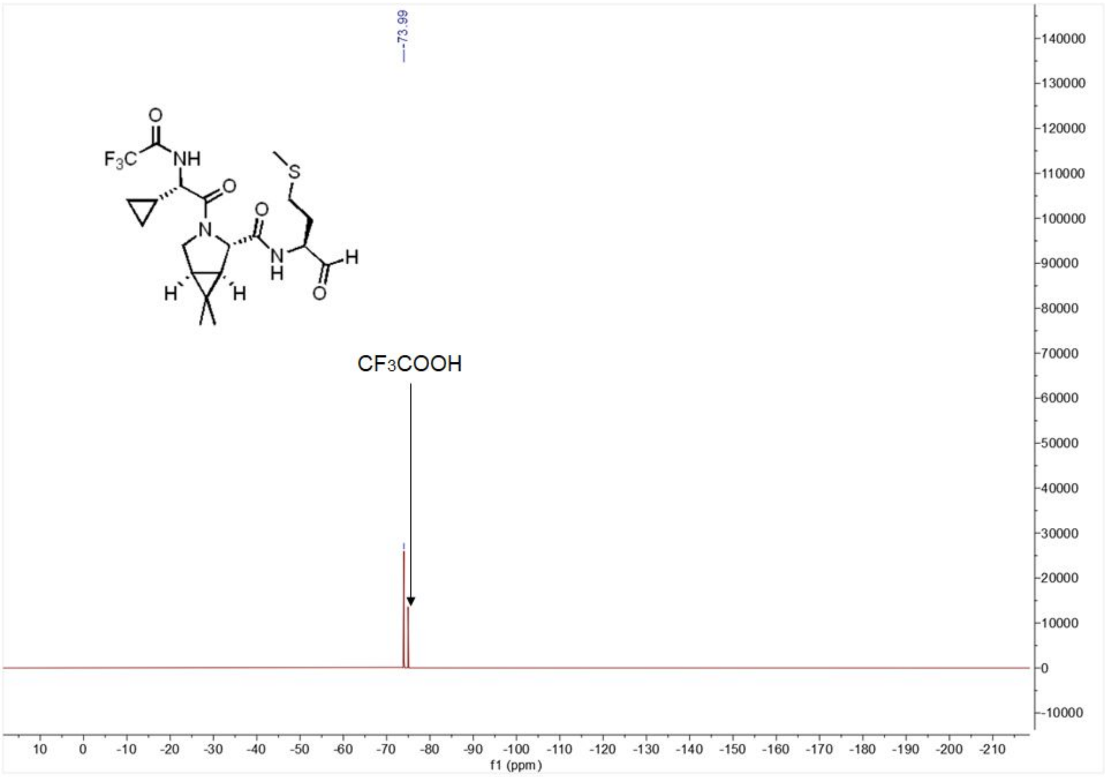

^{19}F NMR spectra of **Jun15574**

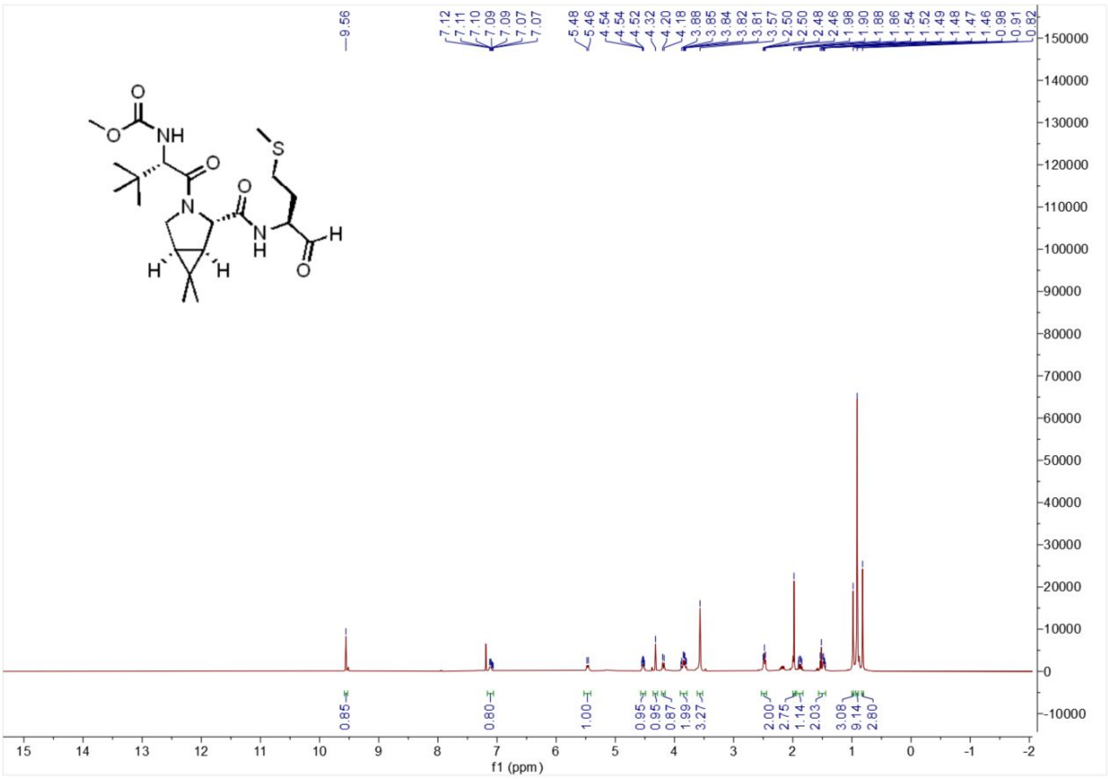

^1H NMR spectra of **Jun13699**

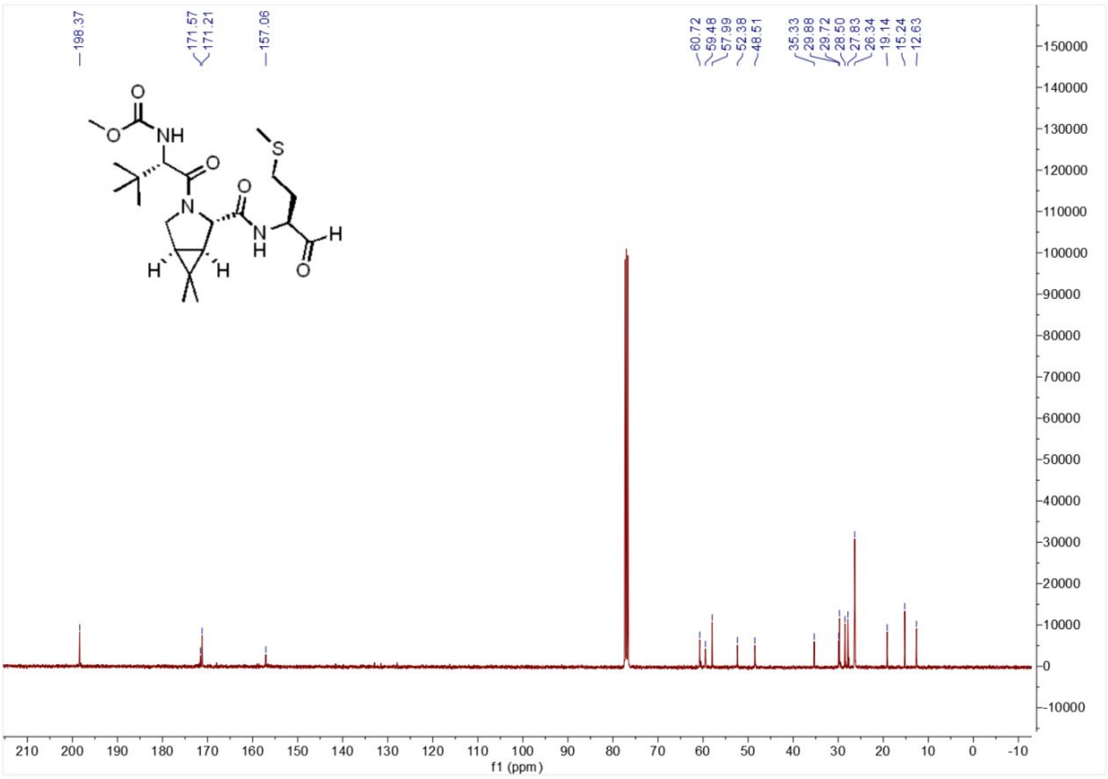

^{13}C NMR spectra of **Jun13699**

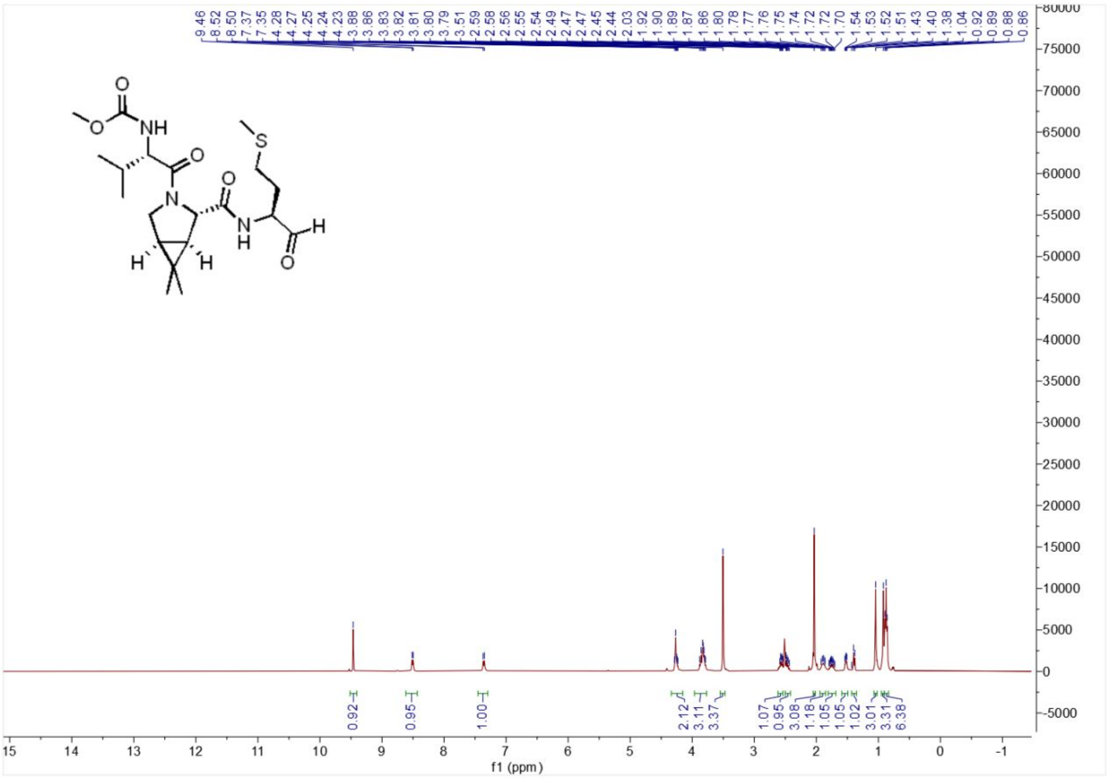

^{1}H NMR spectra of **Jun15516**

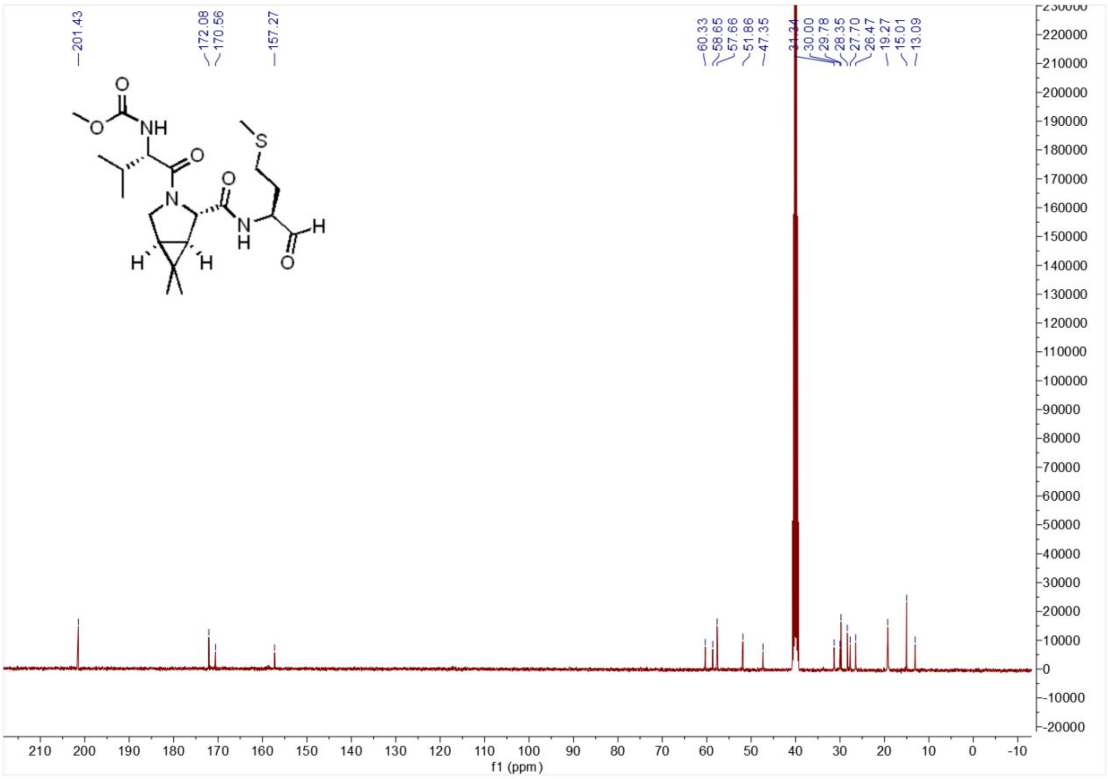

^{13}C NMR spectra of **Jun15516**

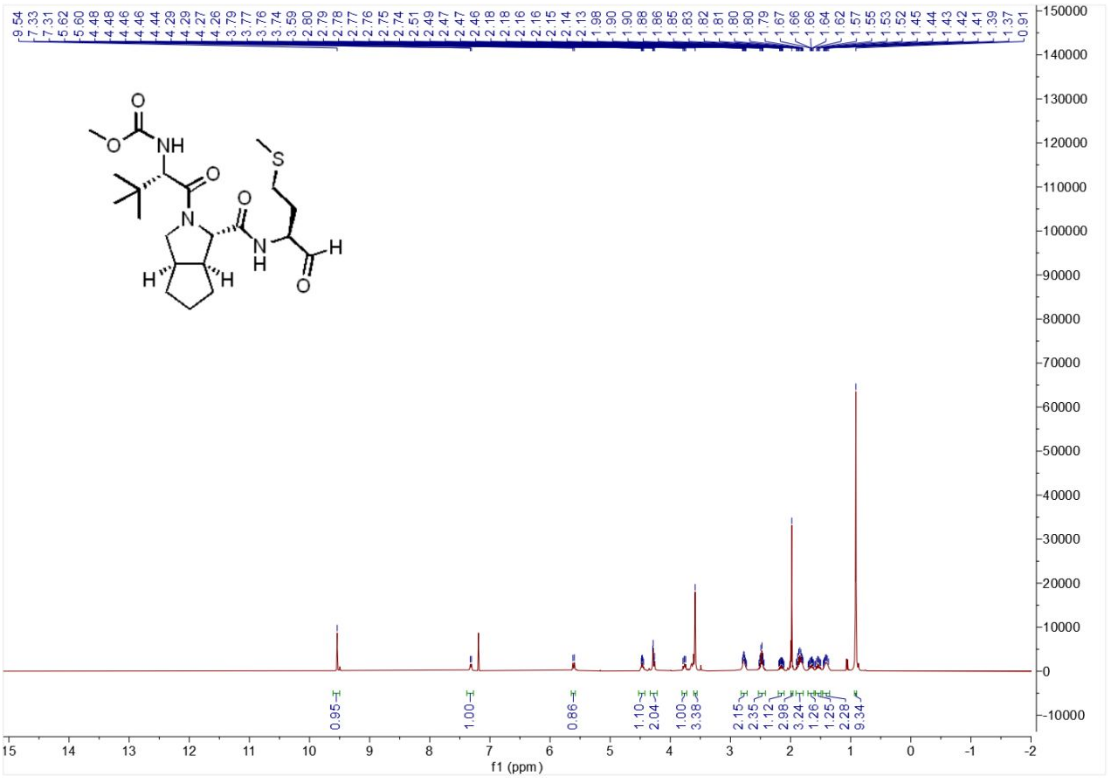

^{1}H NMR spectra of **Jun13856**

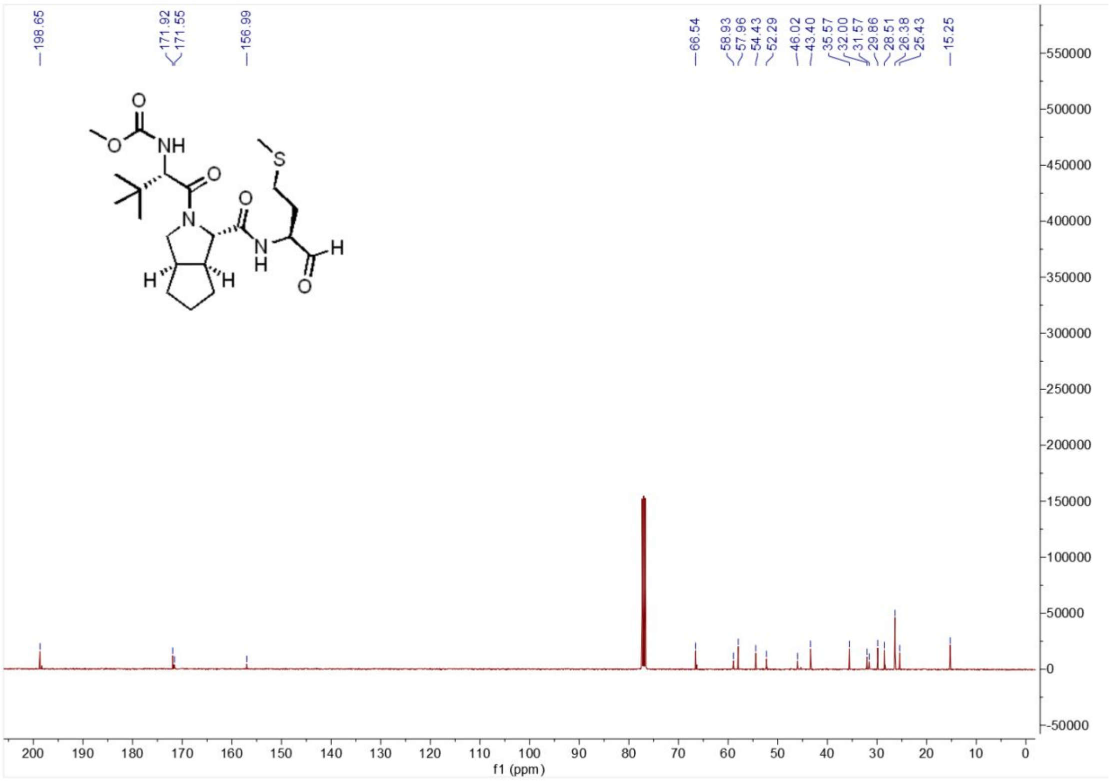

^{13}C NMR spectra of **Jun13856**

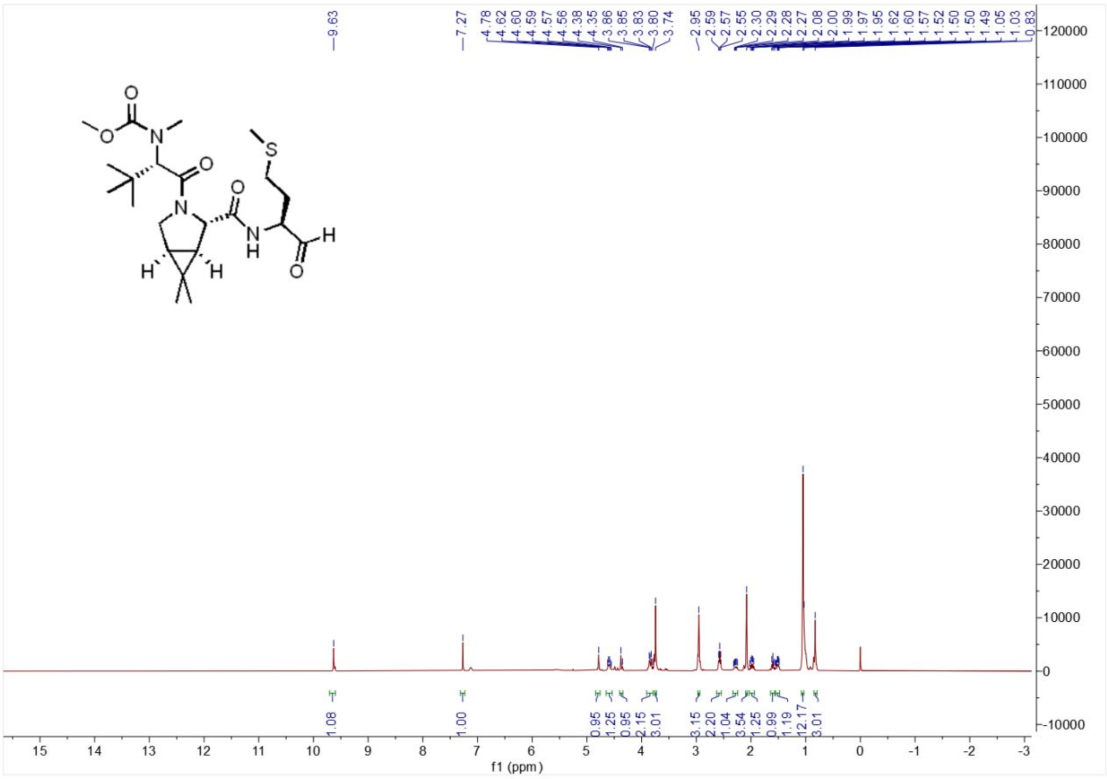

^{1}H NMR spectra of **Jun15514**

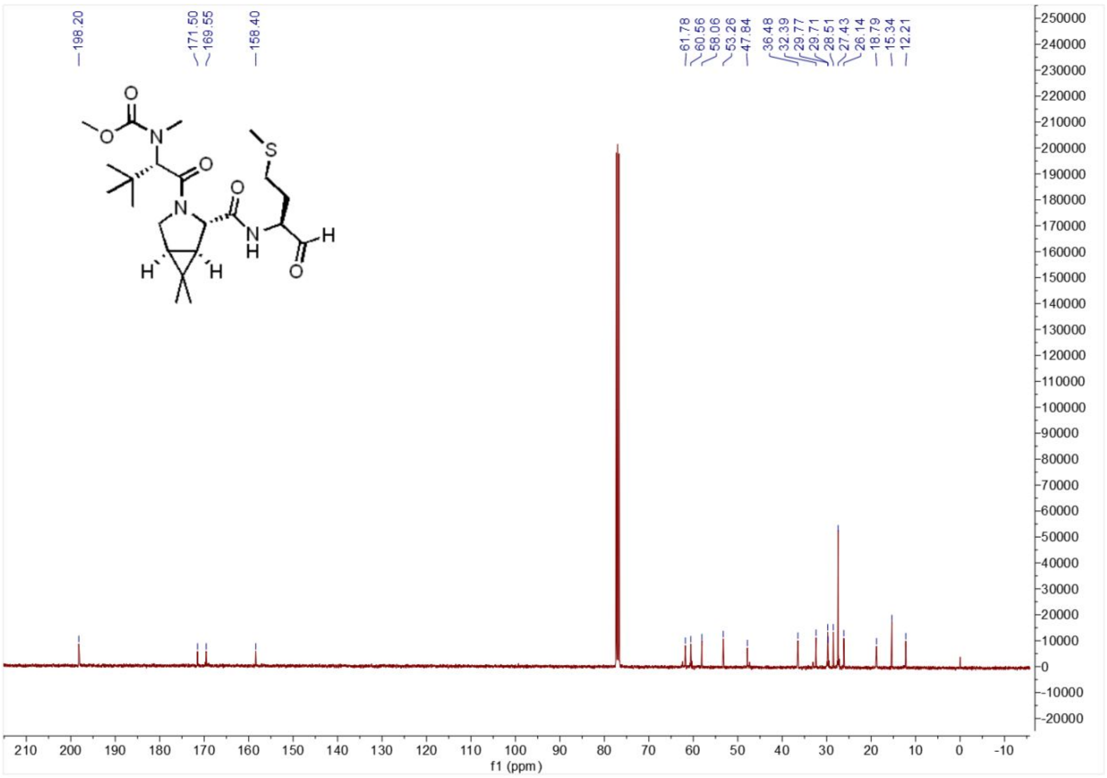

^{13}C NMR spectra of **Jun15514**

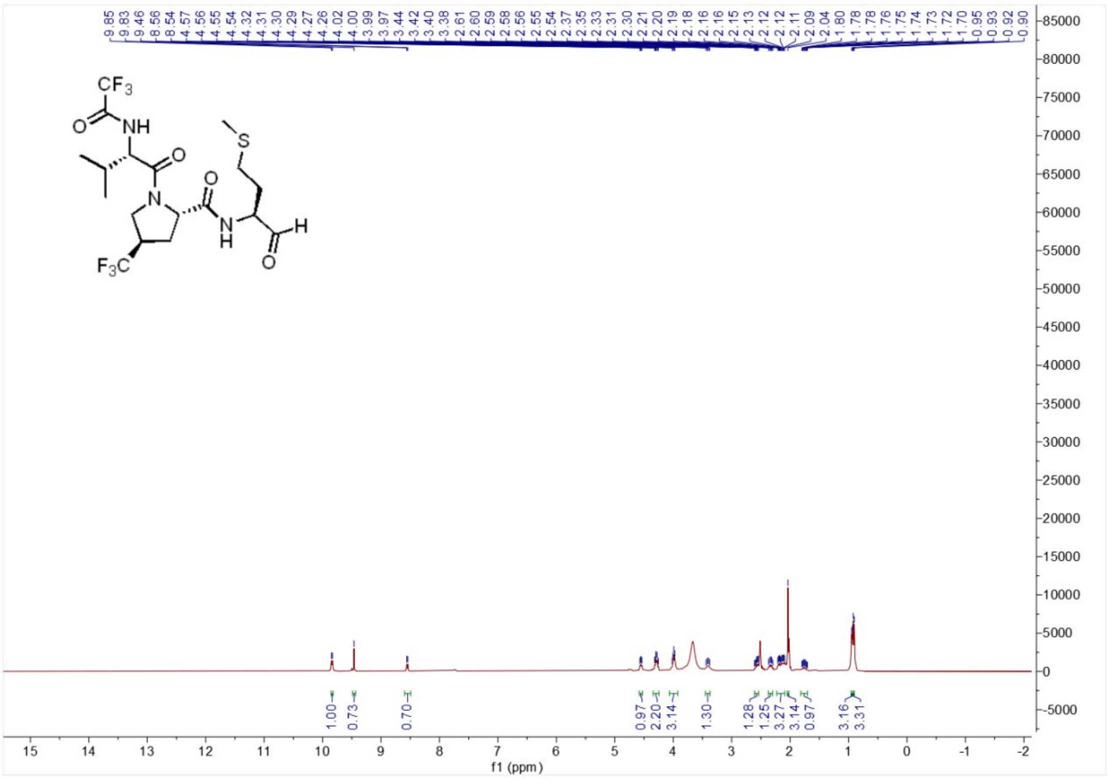

^{1}H NMR spectra of **Jun15666**

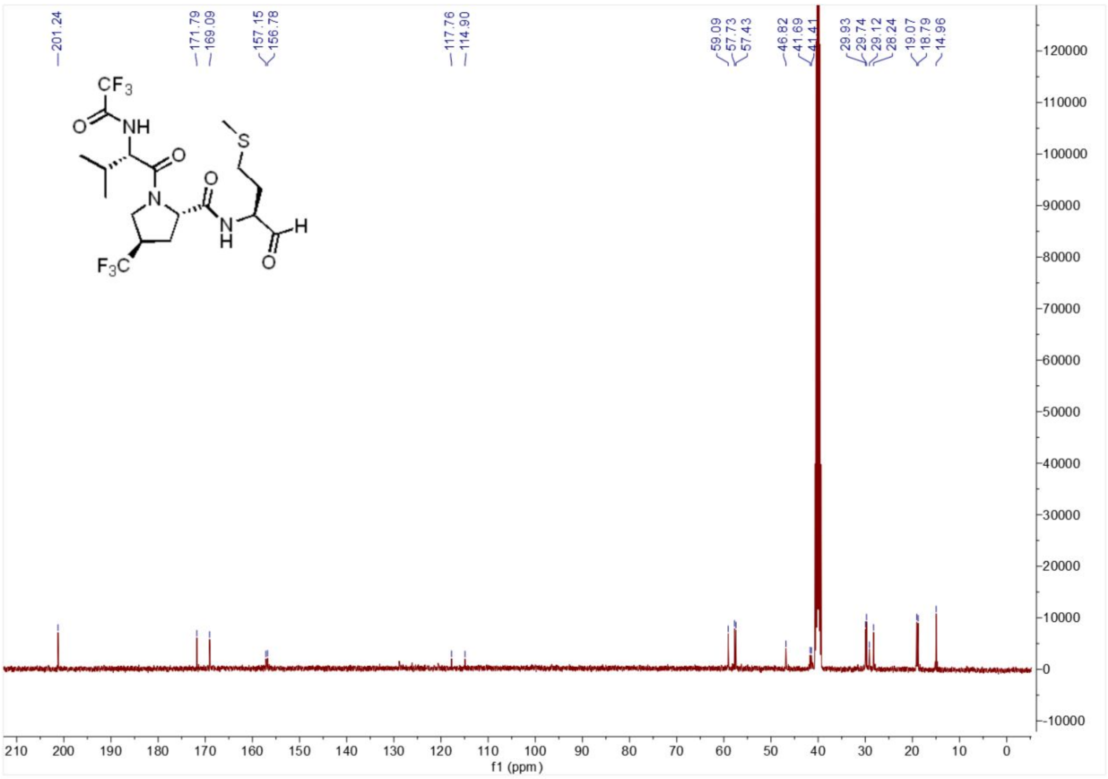

^{13}C NMR spectra of **Jun15666**

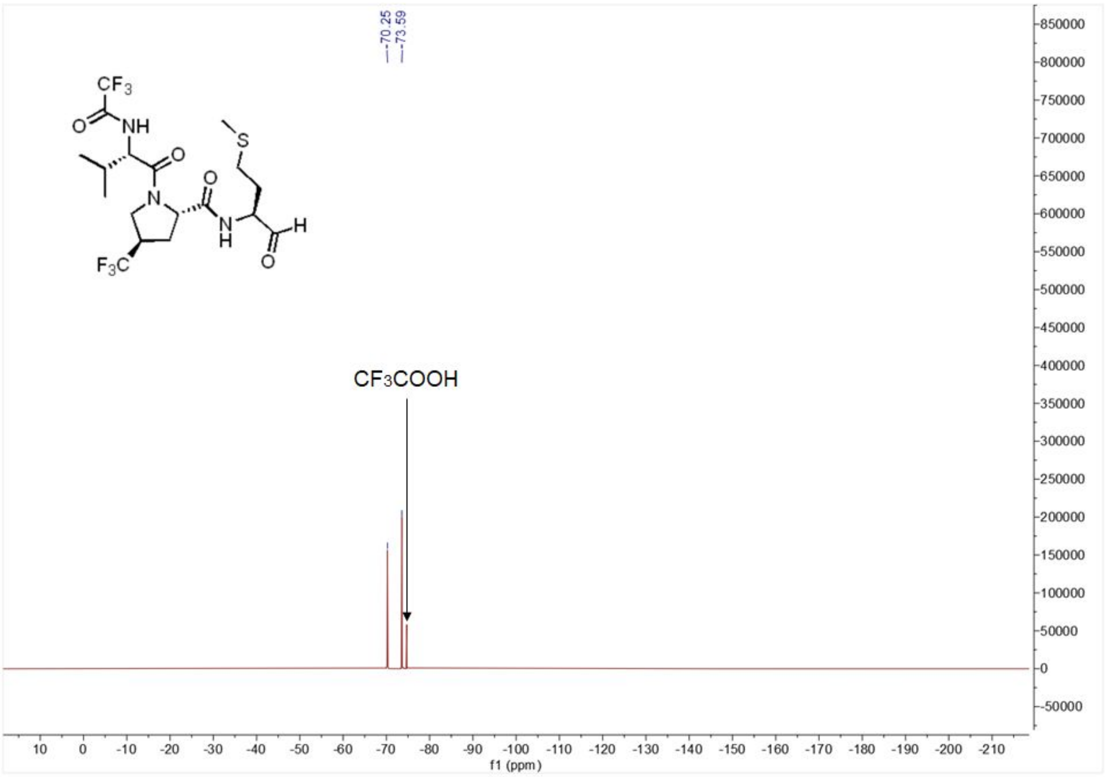

^{19}F NMR spectra of **Jun15666**

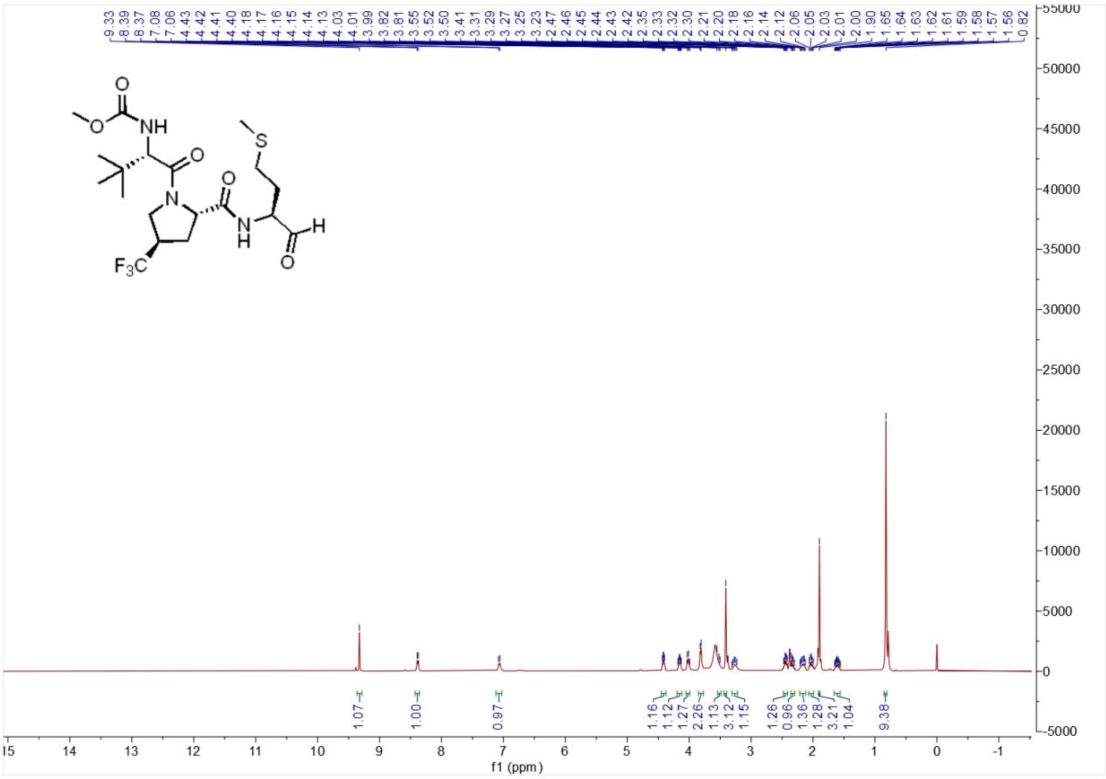

^{1}H NMR spectra of **Jun15515**

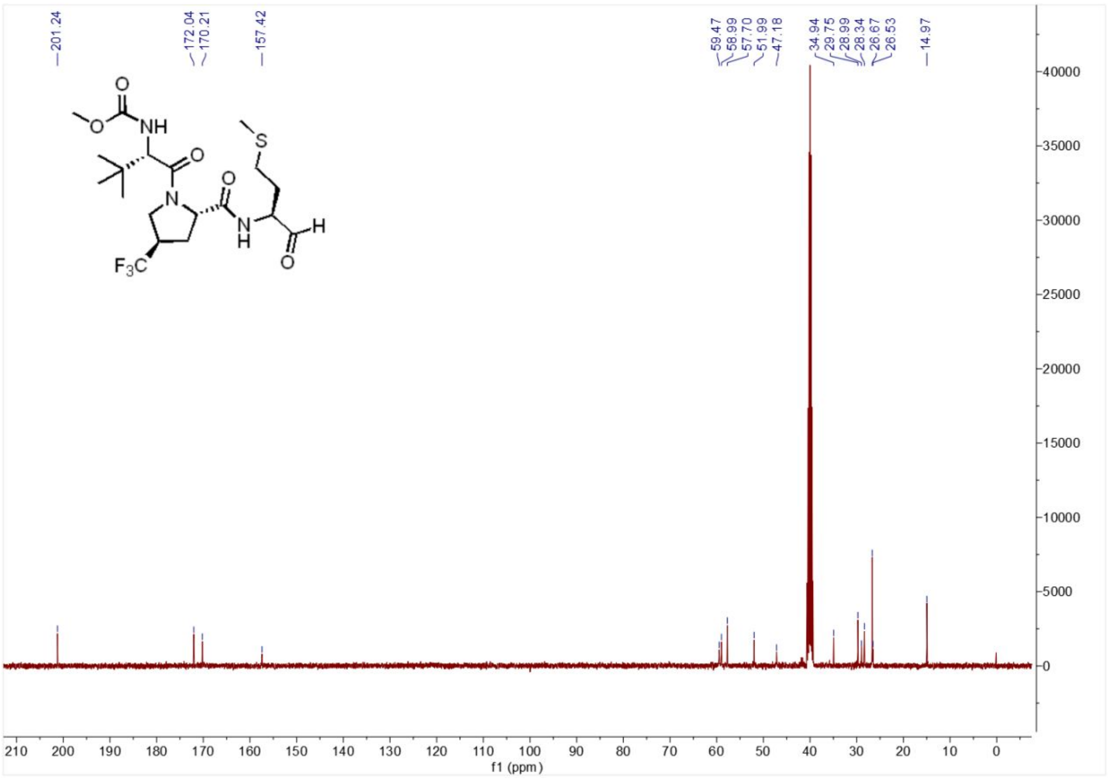

^{13}C NMR spectra of **Jun15515**

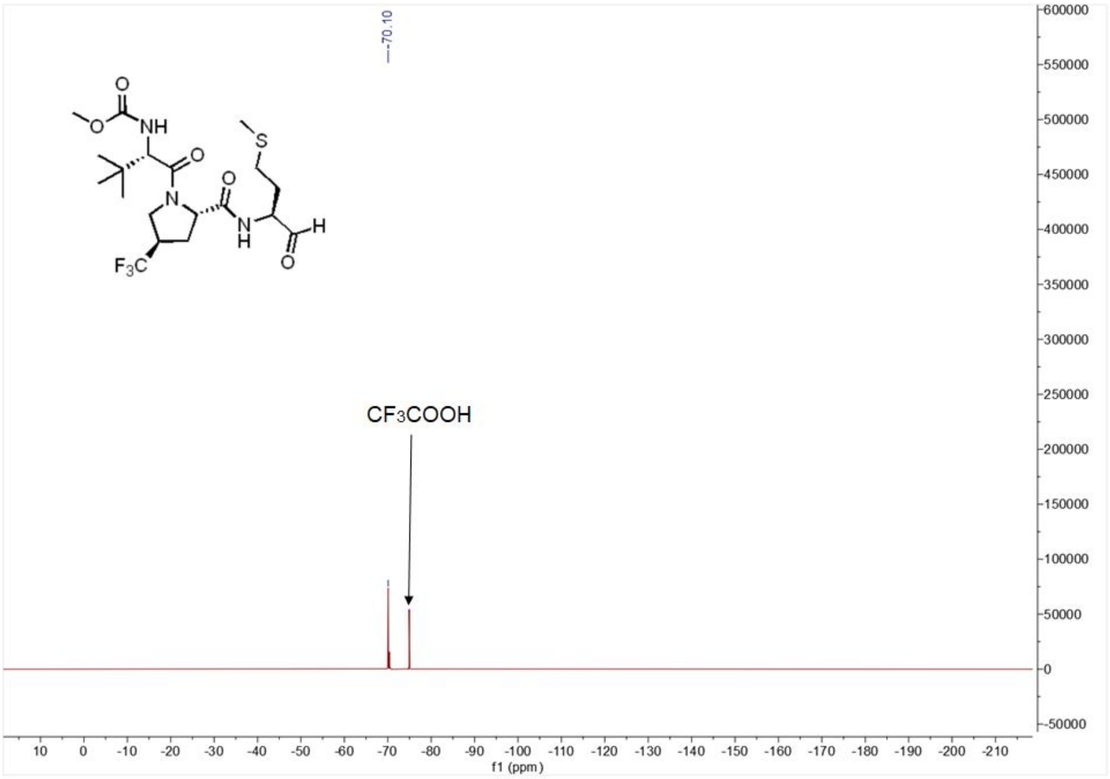

^{19}F NMR spectra of **Jun15515**

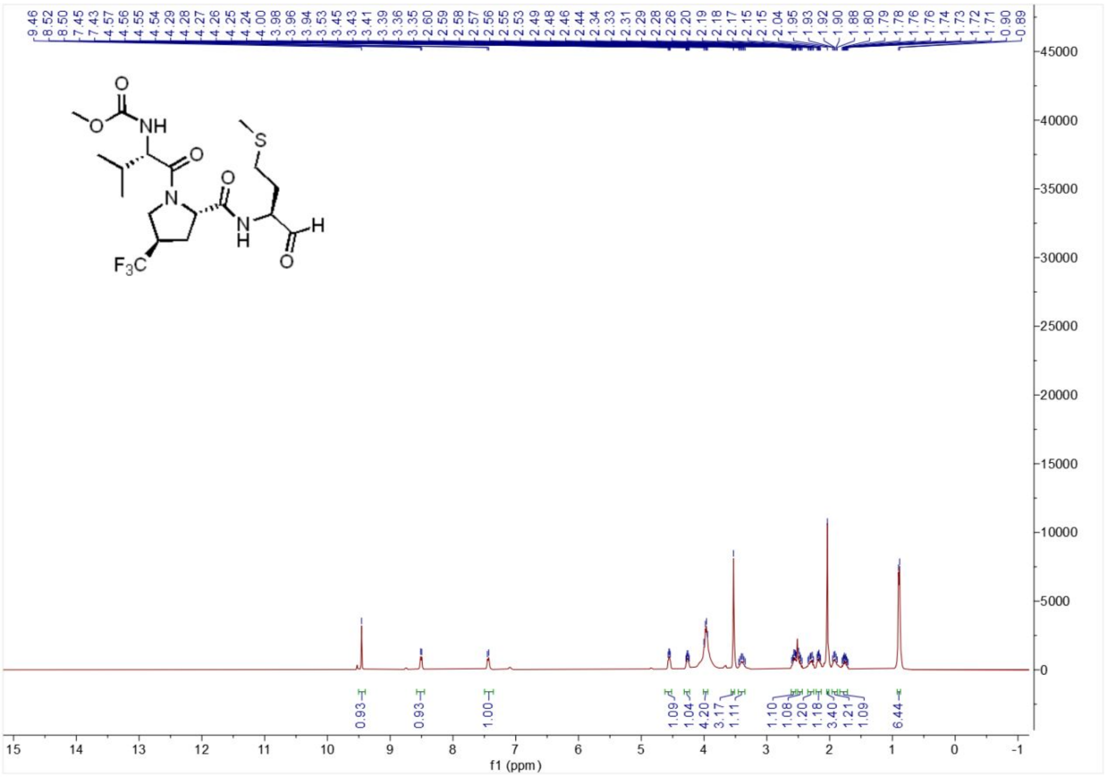

^{1}H NMR spectra of **Jun15634**

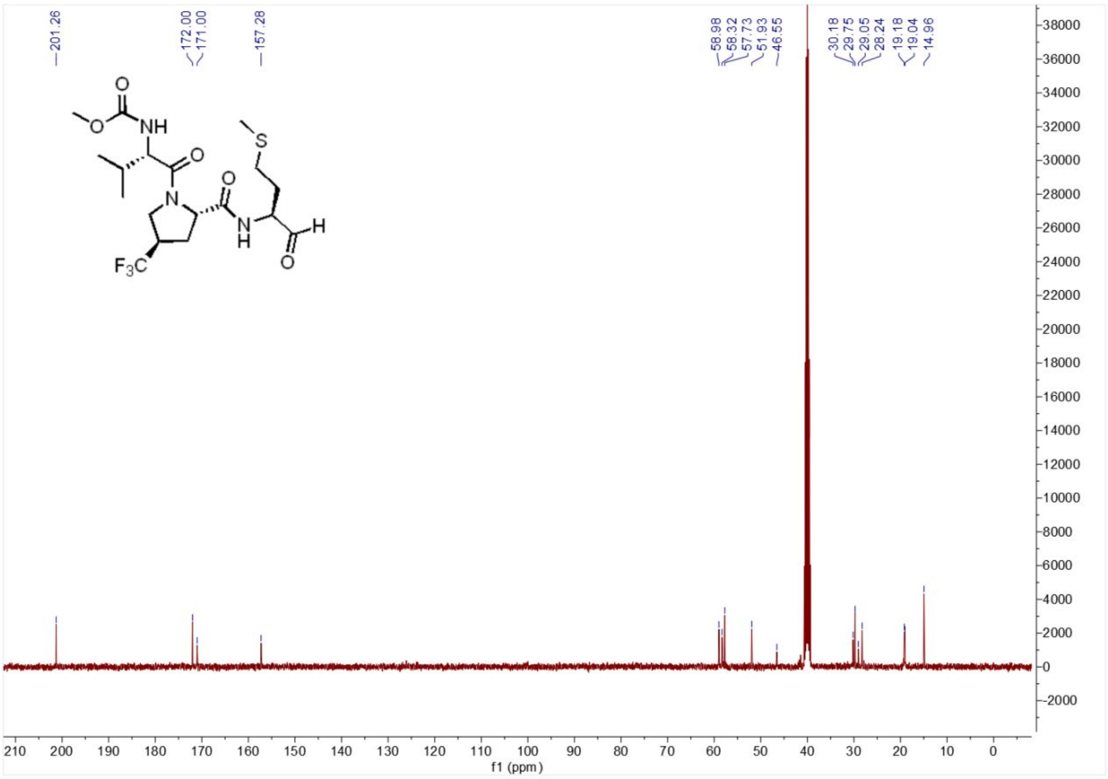

^{13}C NMR spectra of **Jun15634**

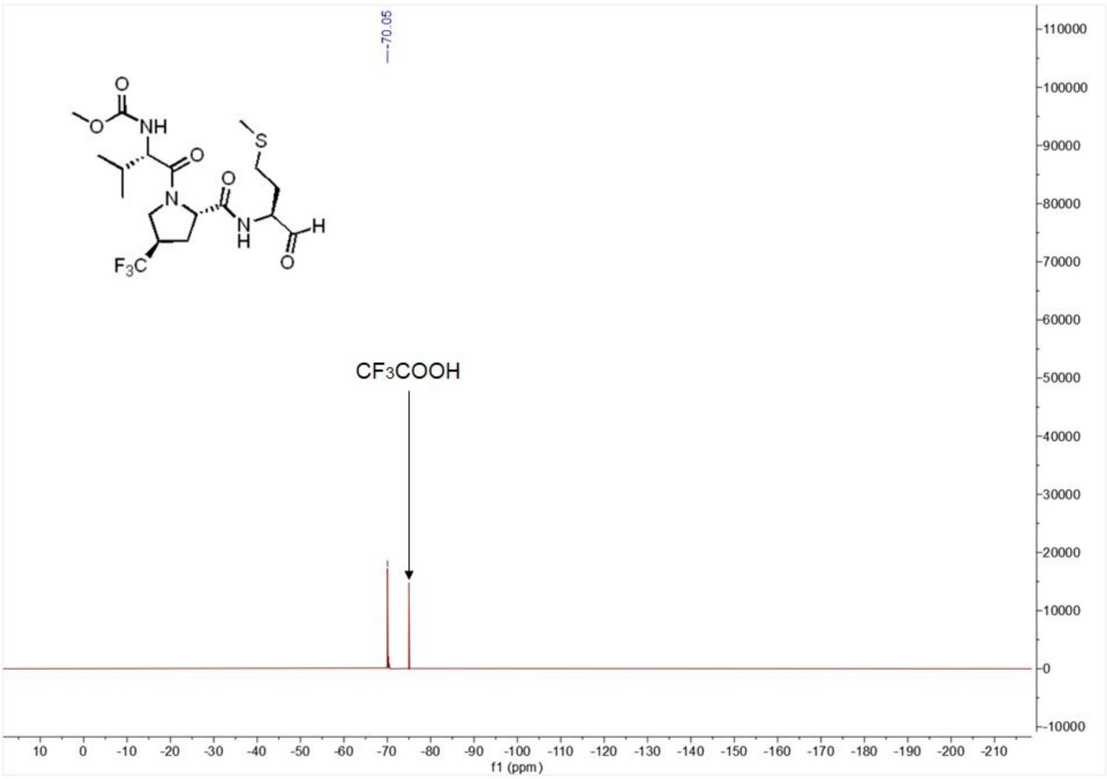

^{19}F NMR spectra of **Jun15634**

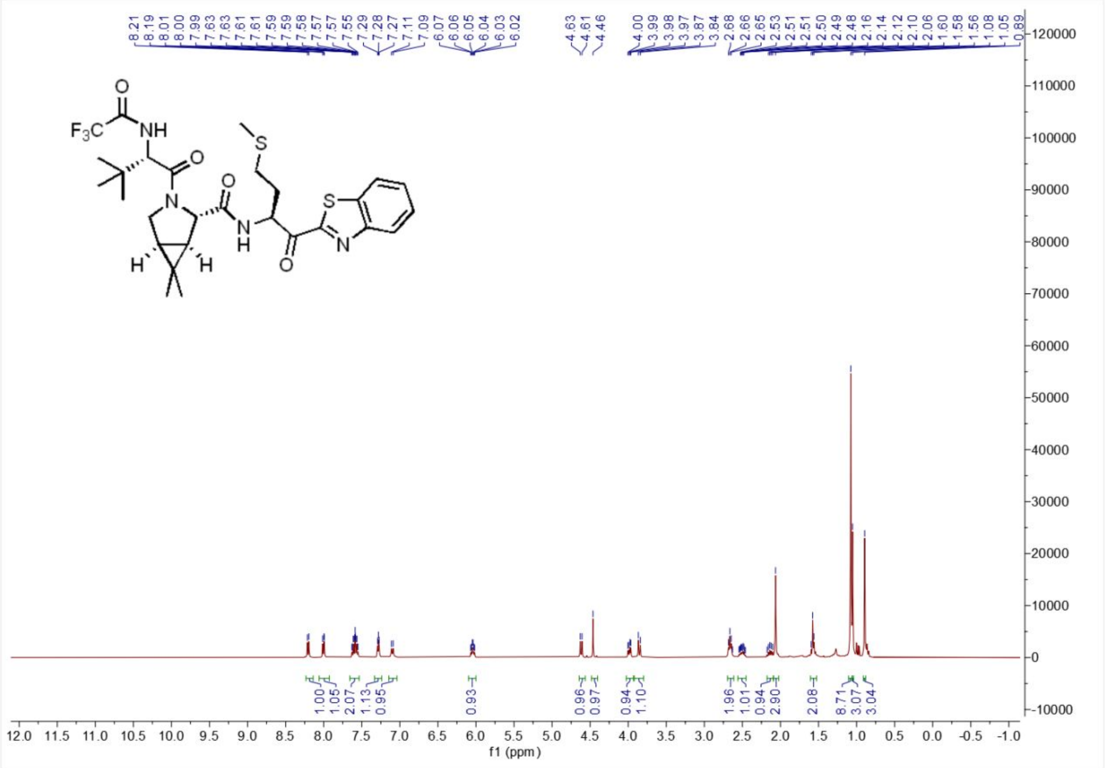

^{1}H NMR spectra of **Jun13603**

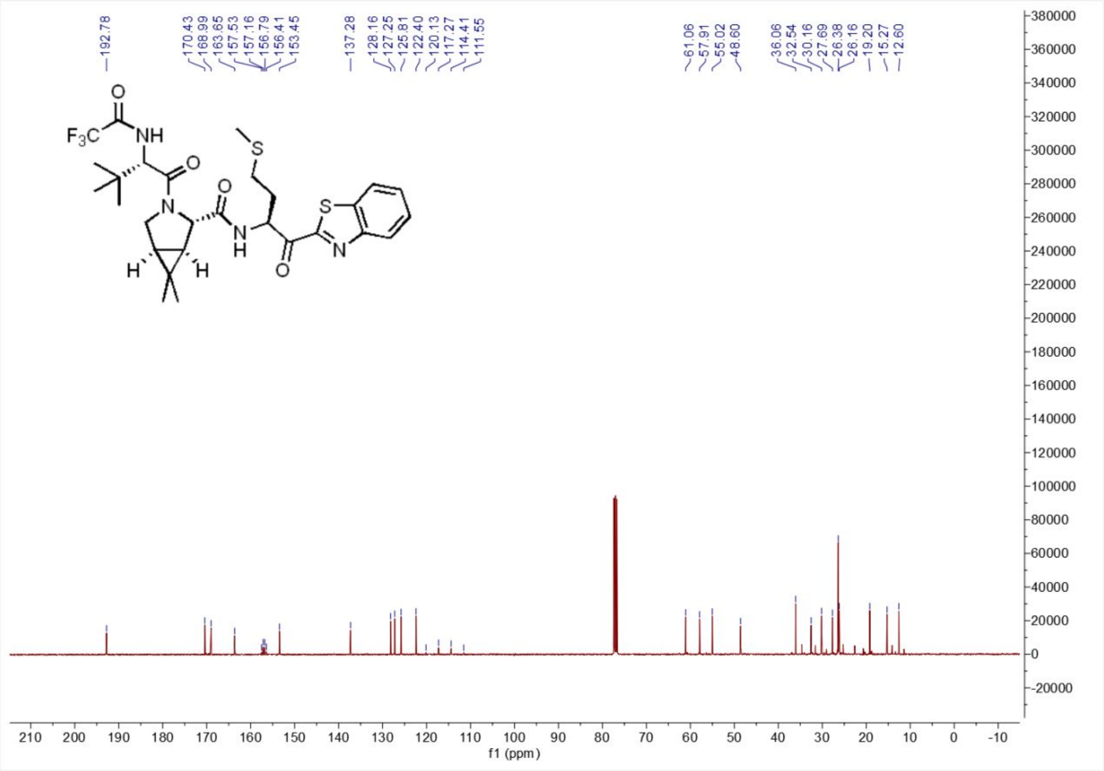

^{13}C NMR spectra of **Jun13603**

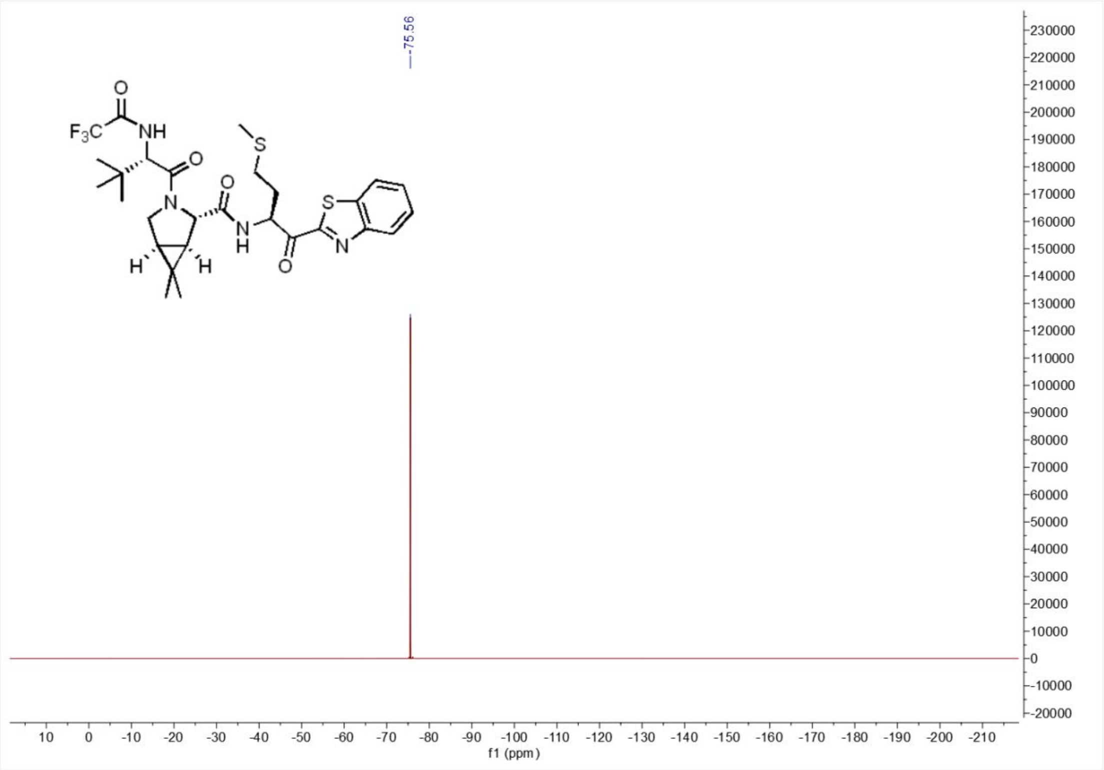

^{19}F NMR spectra of **Jun13603**

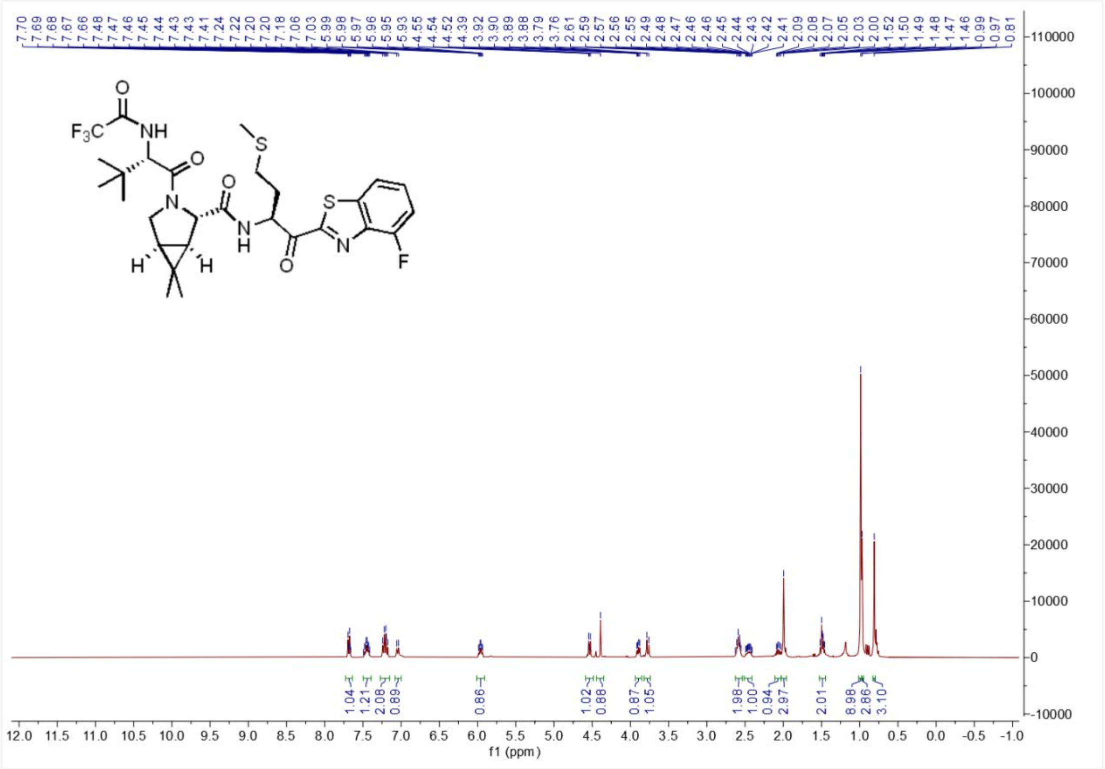

^{1}H NMR spectra of **Jun13604**

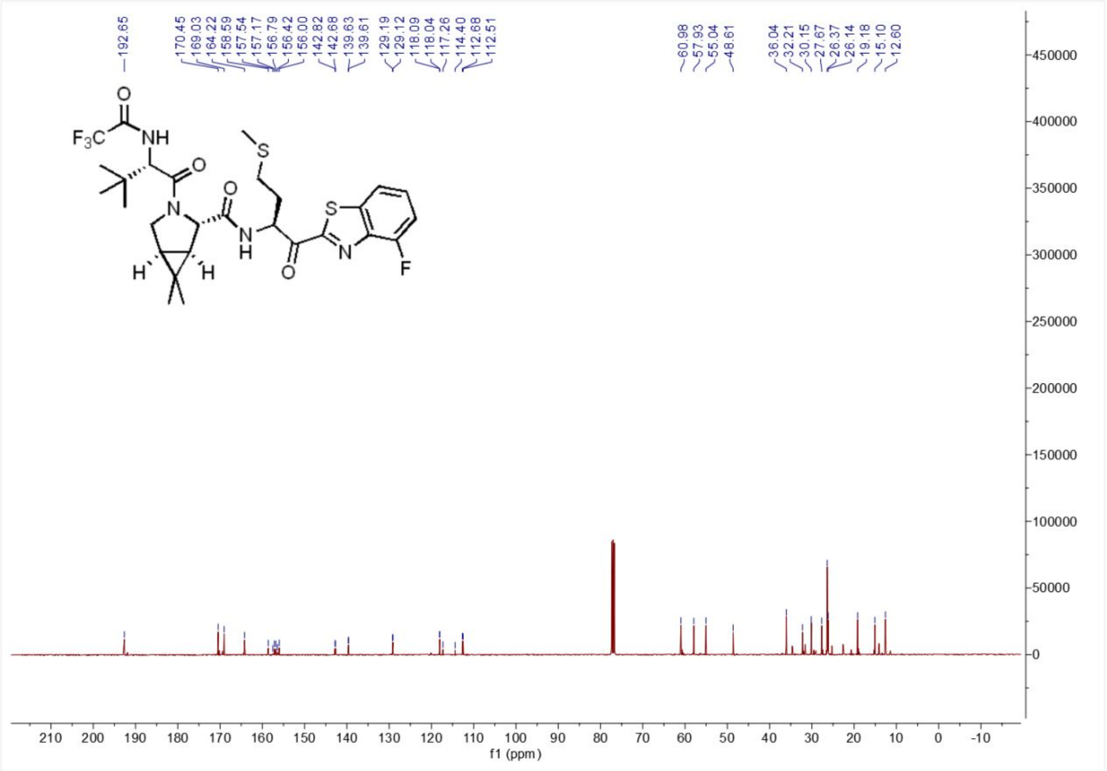

^{13}C NMR spectra of **Jun13604**

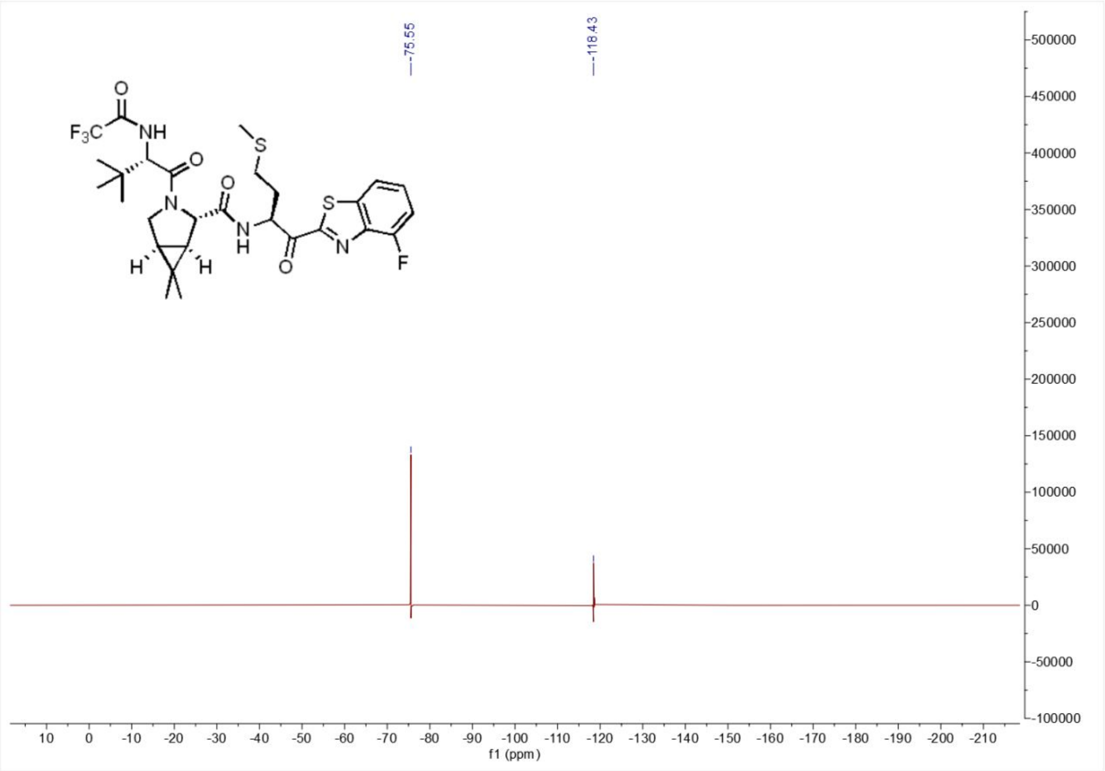

^{19}F NMR spectra of **Jun13604**

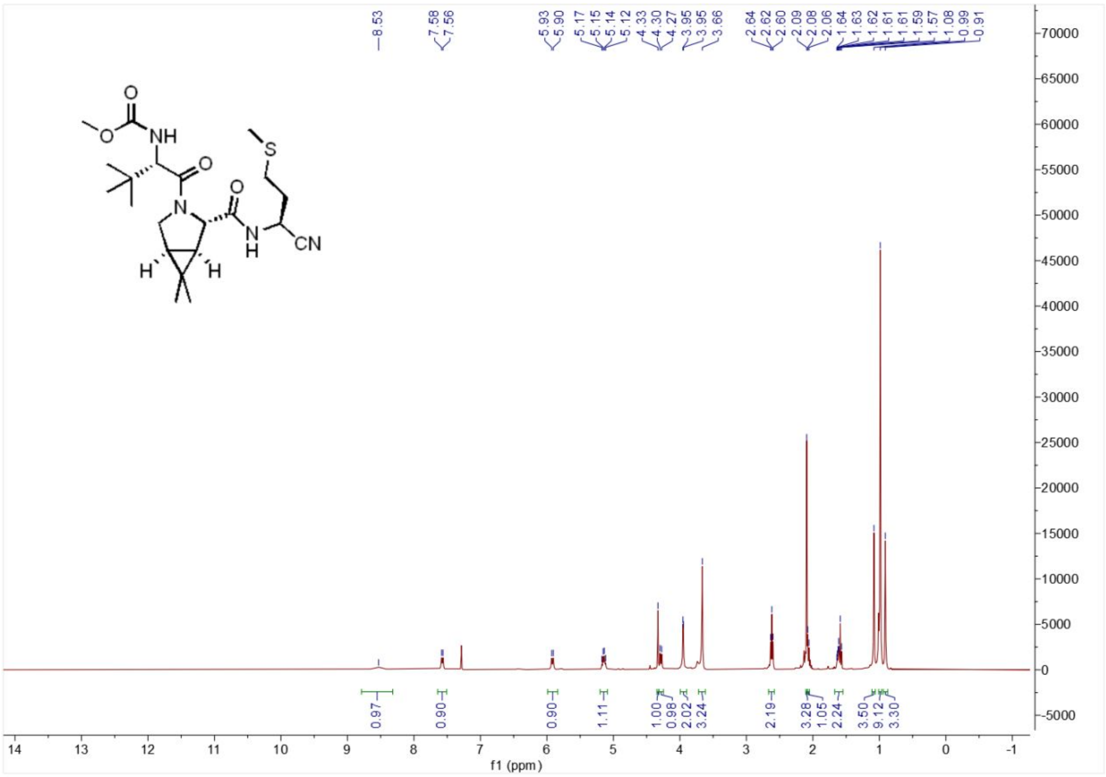

^{1}H NMR spectra of **Jun15467**

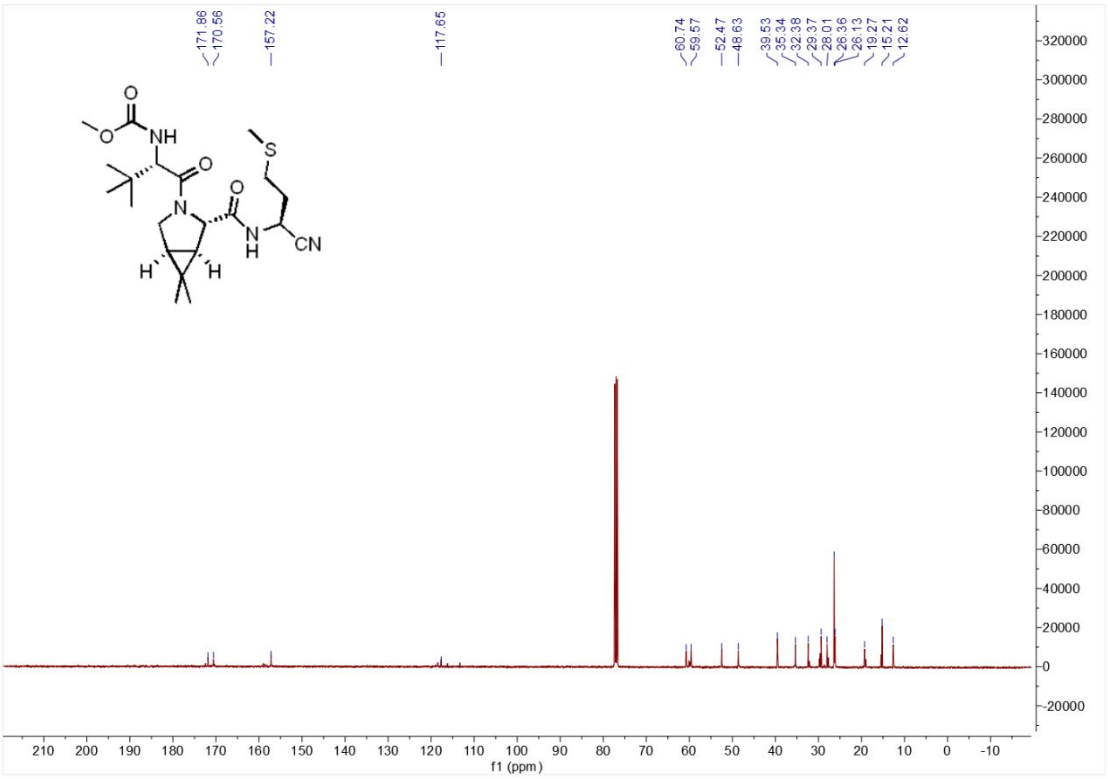

^{13}C NMR spectra of **Jun15467**

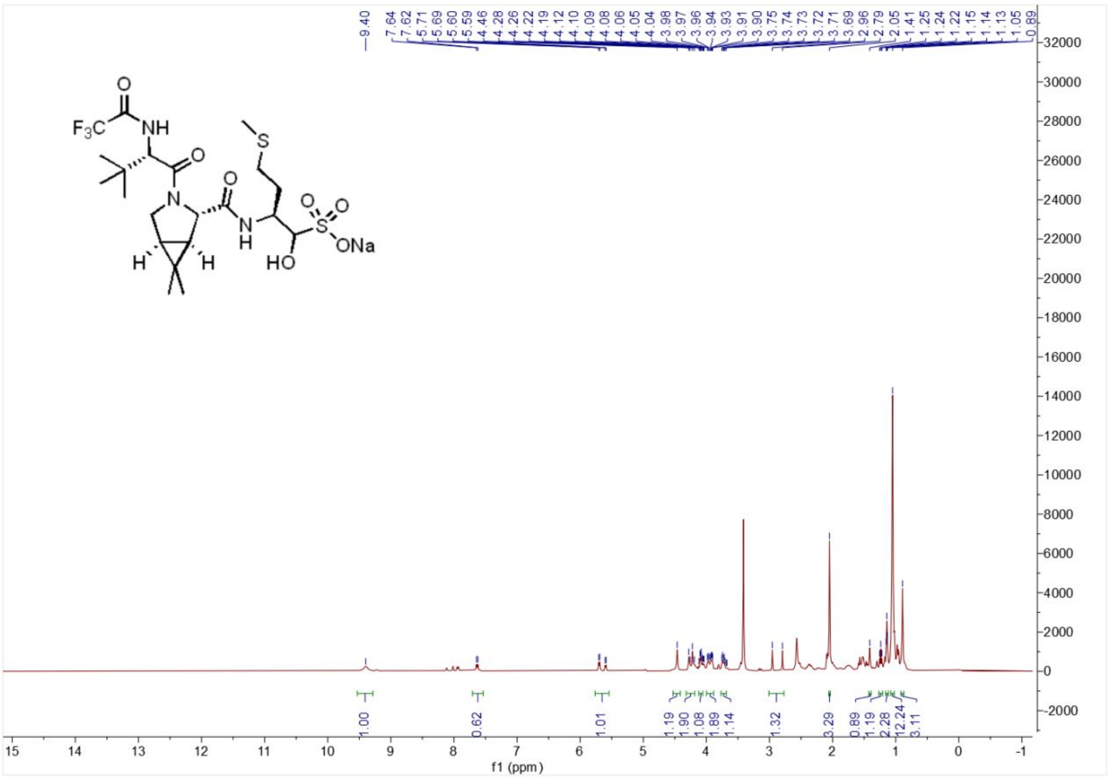

^{1}H NMR spectra of **Jun12607**

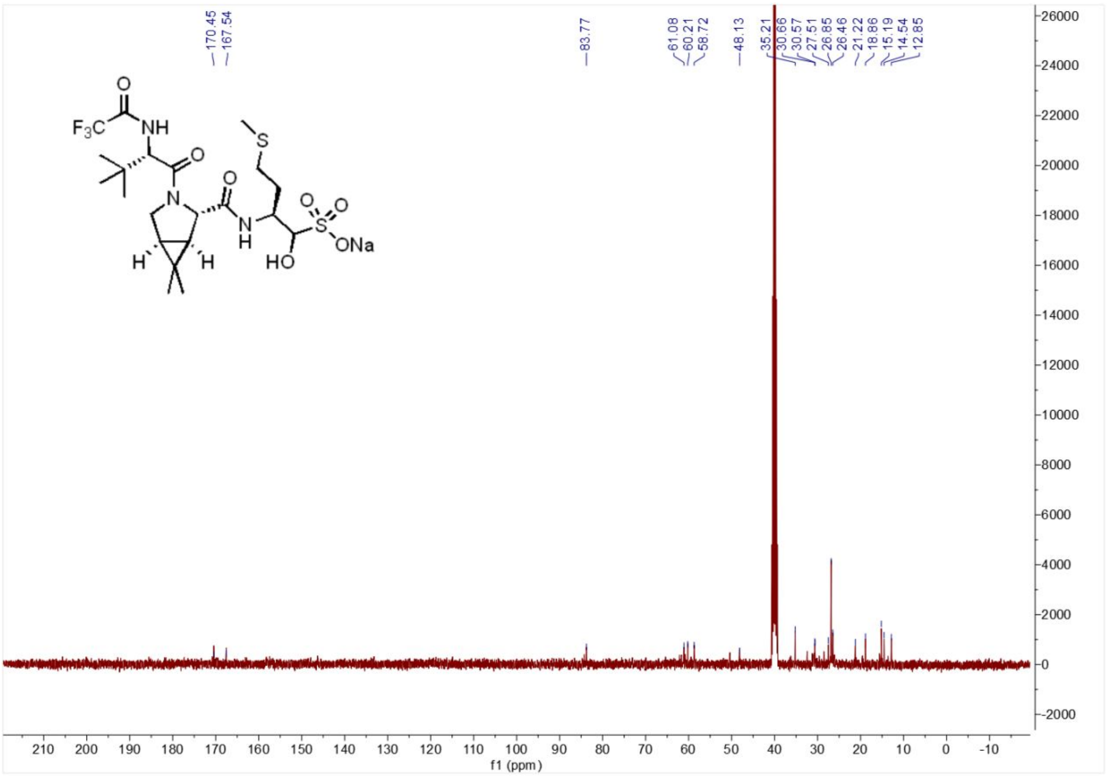

^{13}C NMR spectra of **Jun12607**

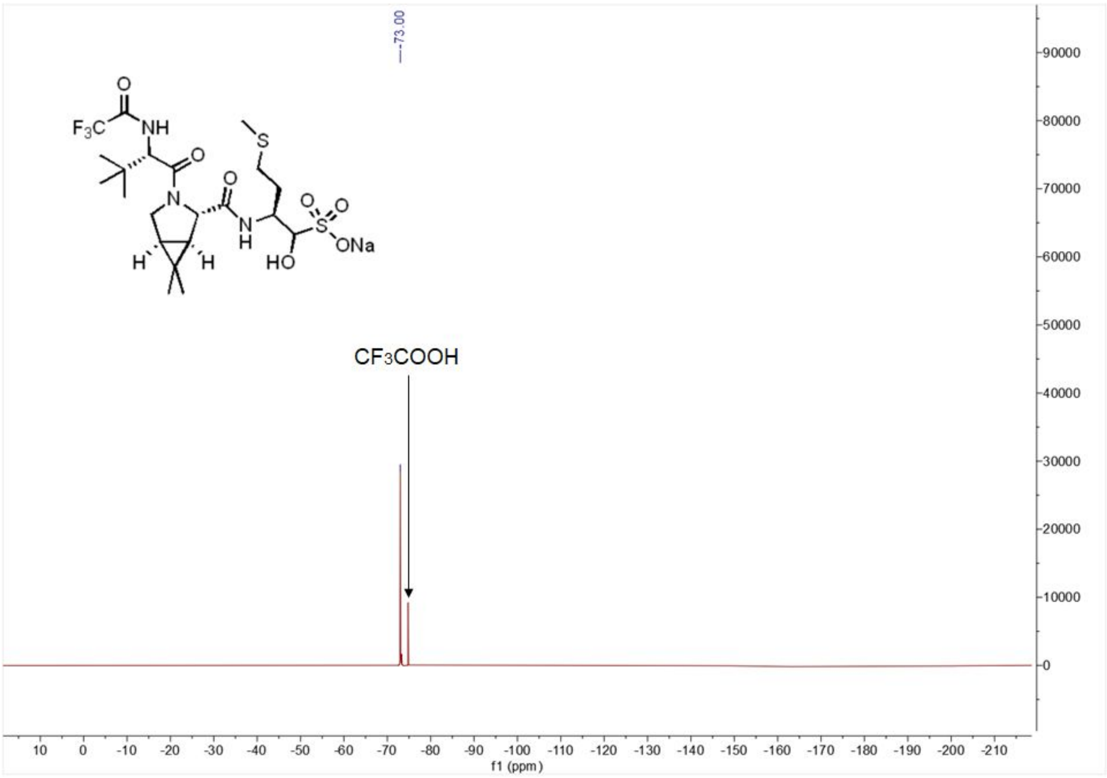

^{19}F NMR spectra of **Jun12607**

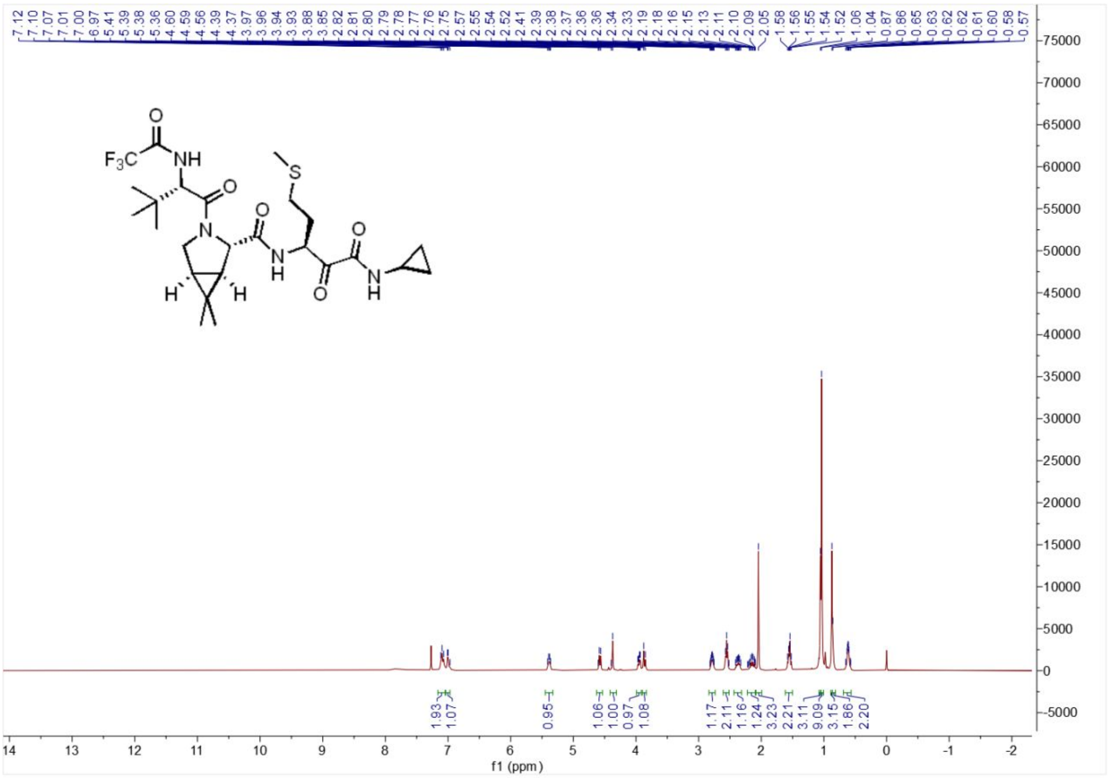

^{1}H NMR spectra of **Jun1422**

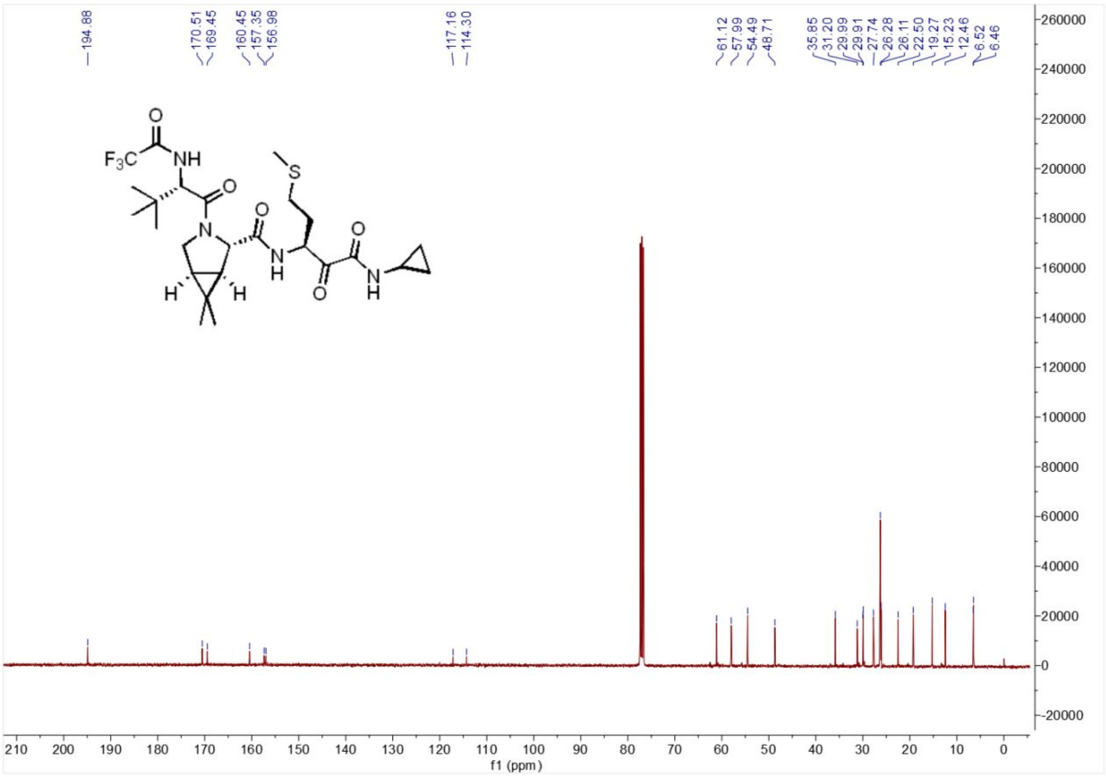

^{13}C NMR spectra of **Jun1422**

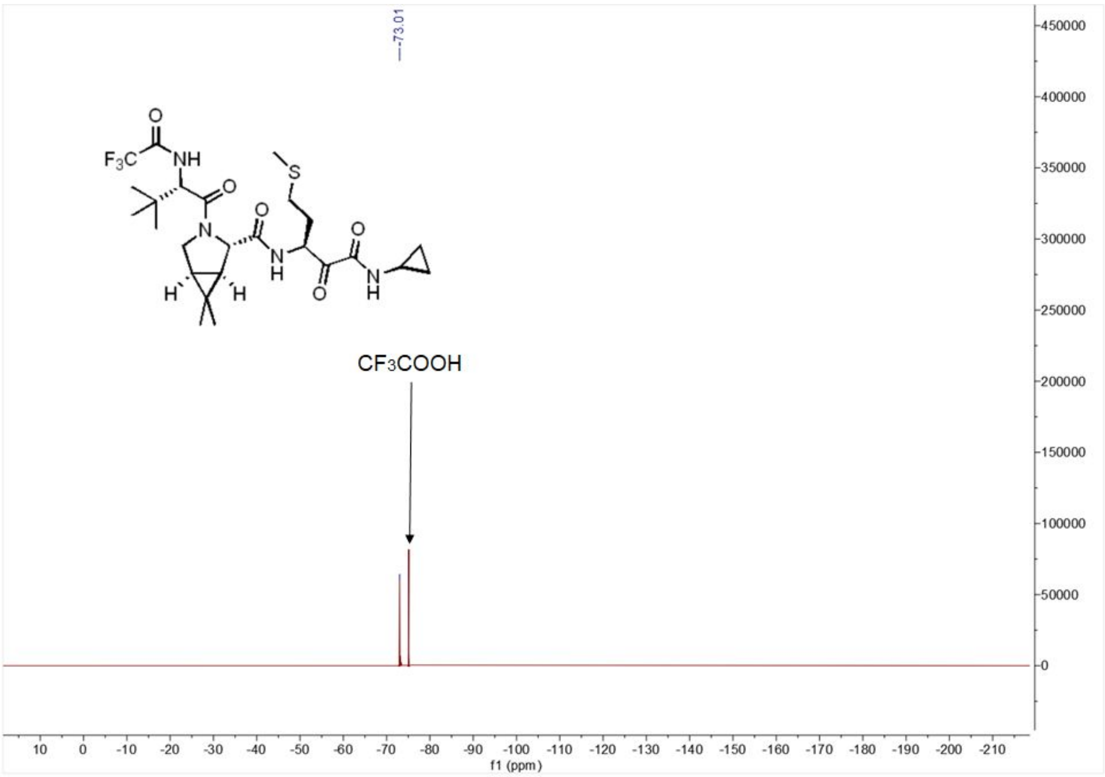

^{19}F NMR spectra of **Jun1422**

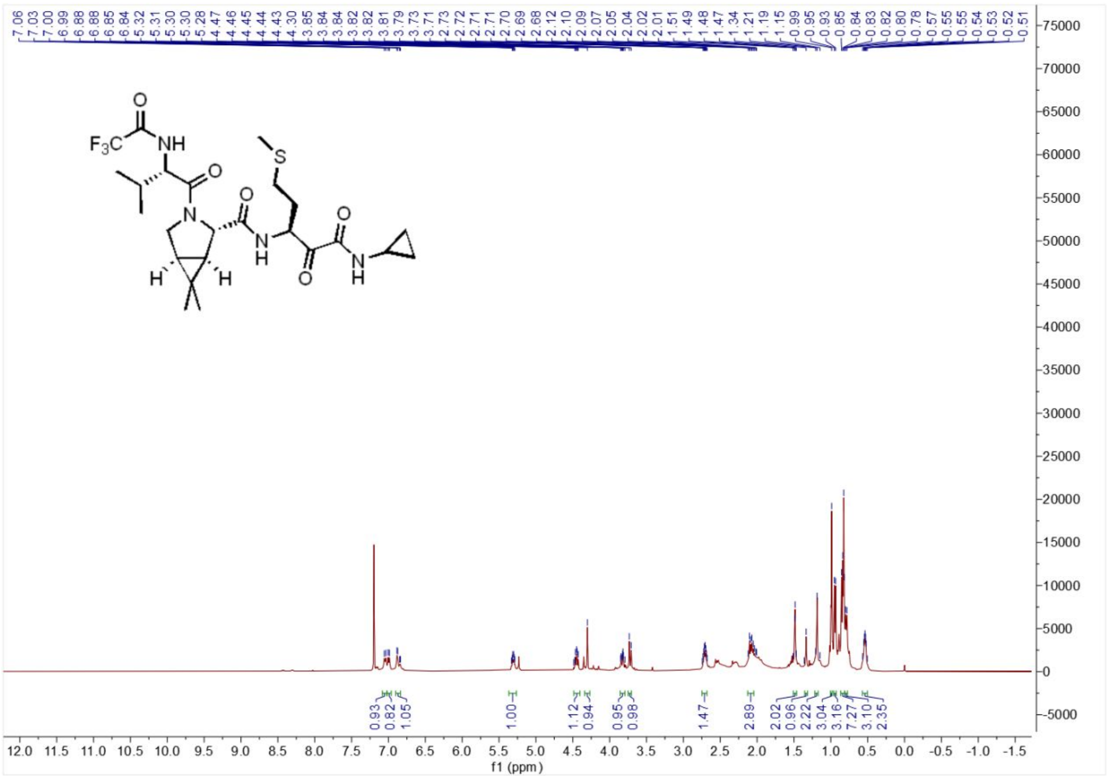

^{1}H NMR spectra of **Jun13734**

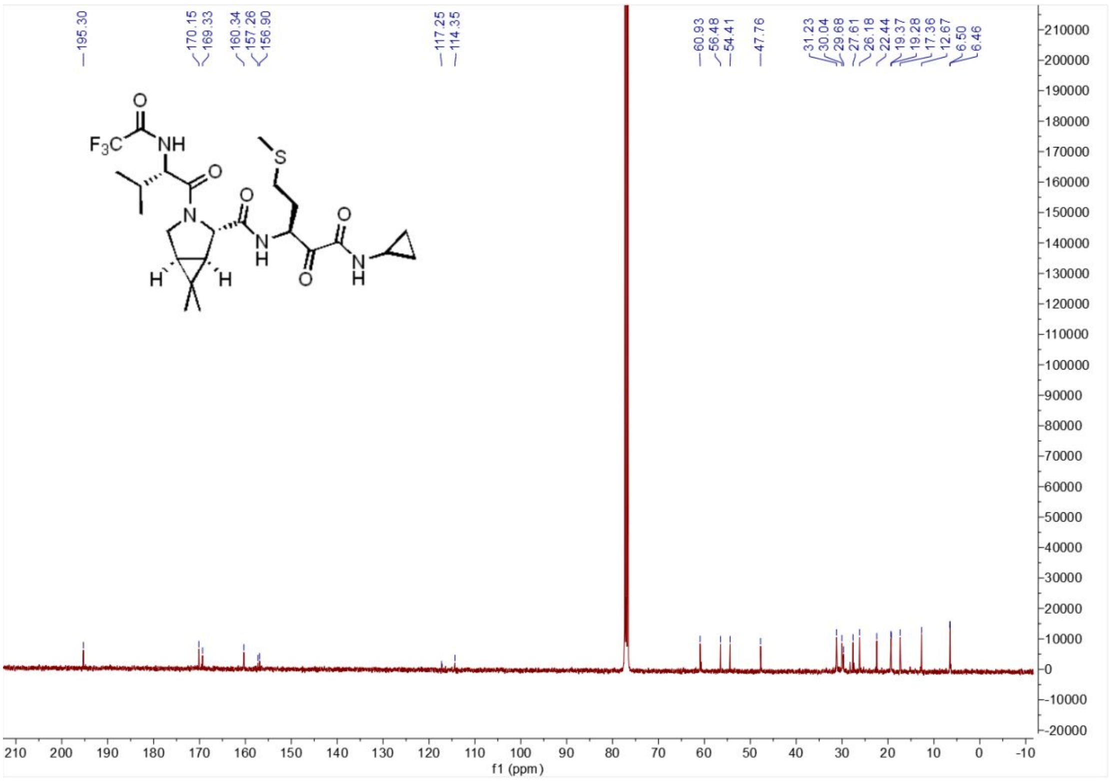

^{13}C NMR spectra of **Jun13734**

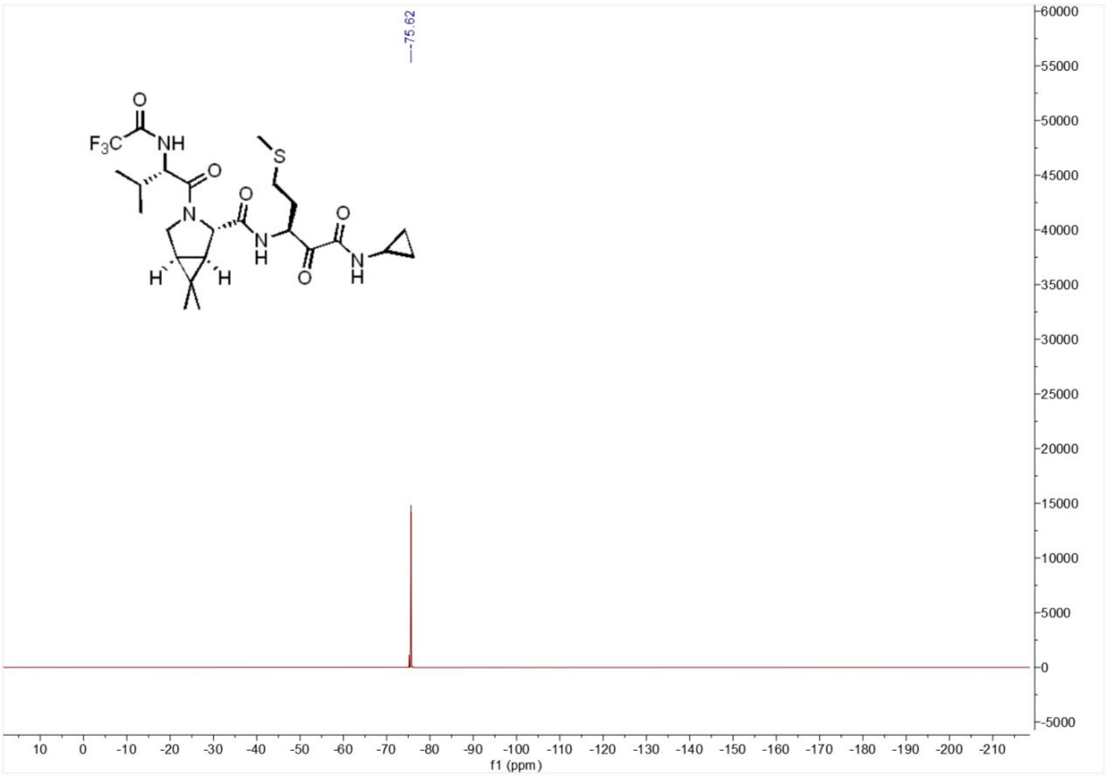

^{19}F NMR spectra of **Jun13734**

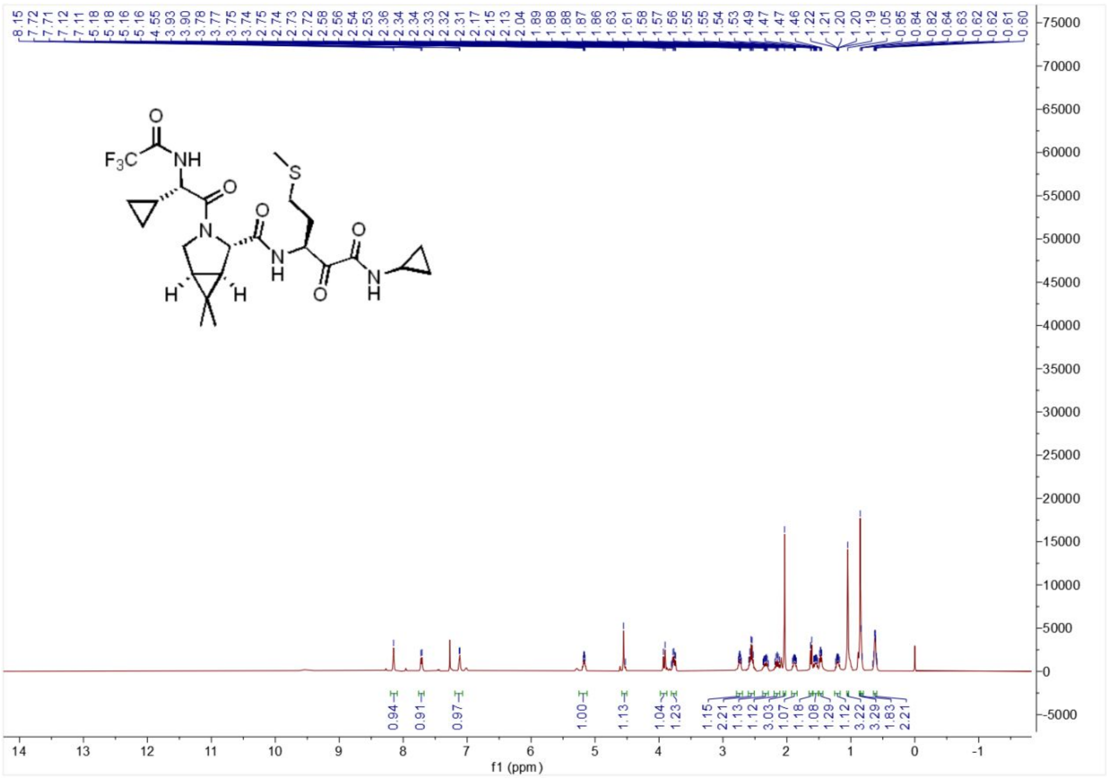

^{1}H NMR spectra of **Jun15575**

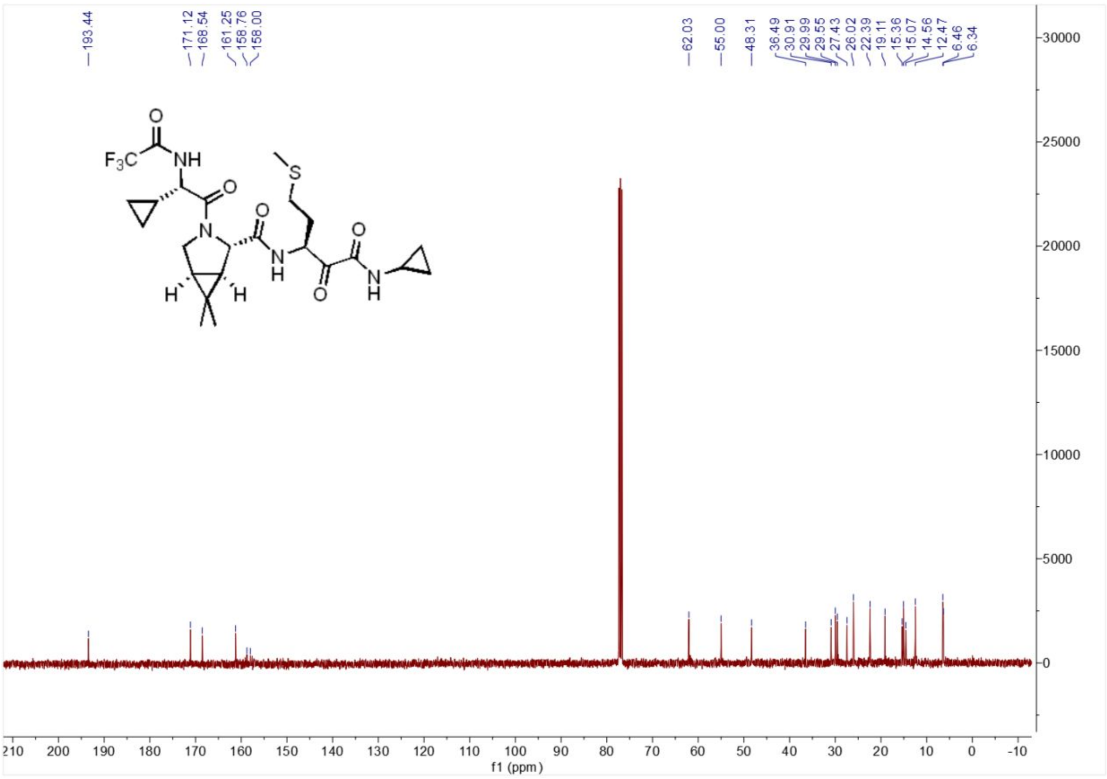

^{13}C NMR spectra of **Jun15575**

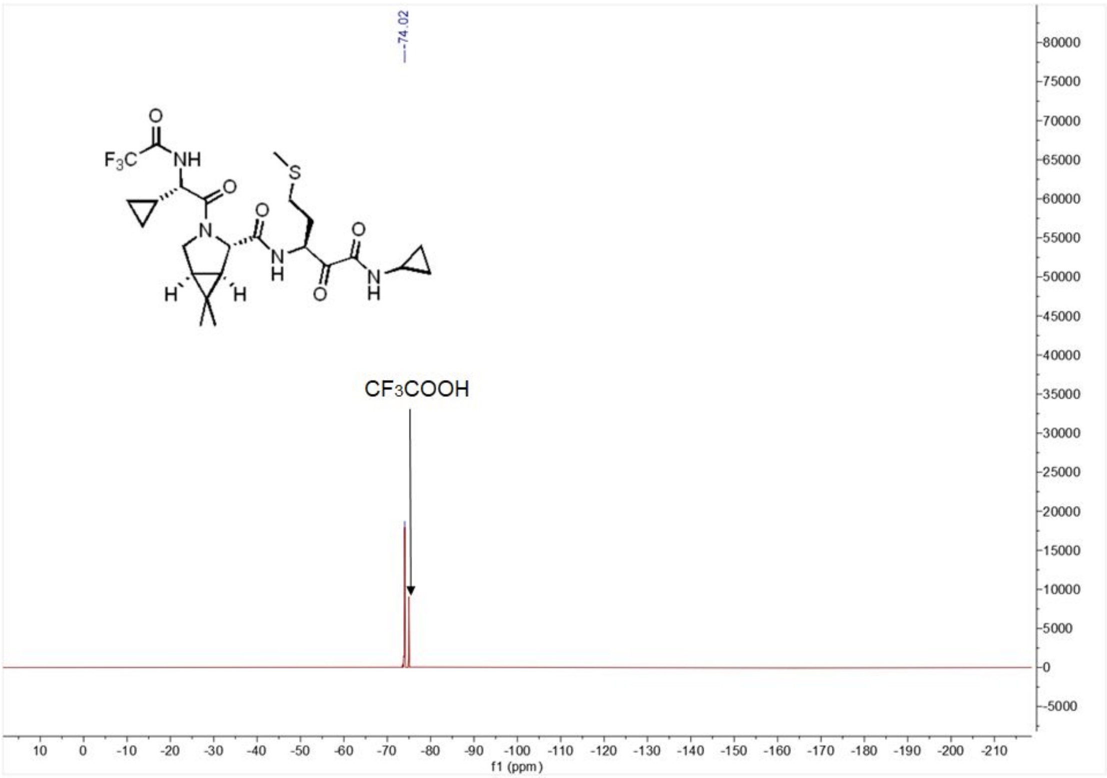

^{19}F NMR spectra of **Jun15575**

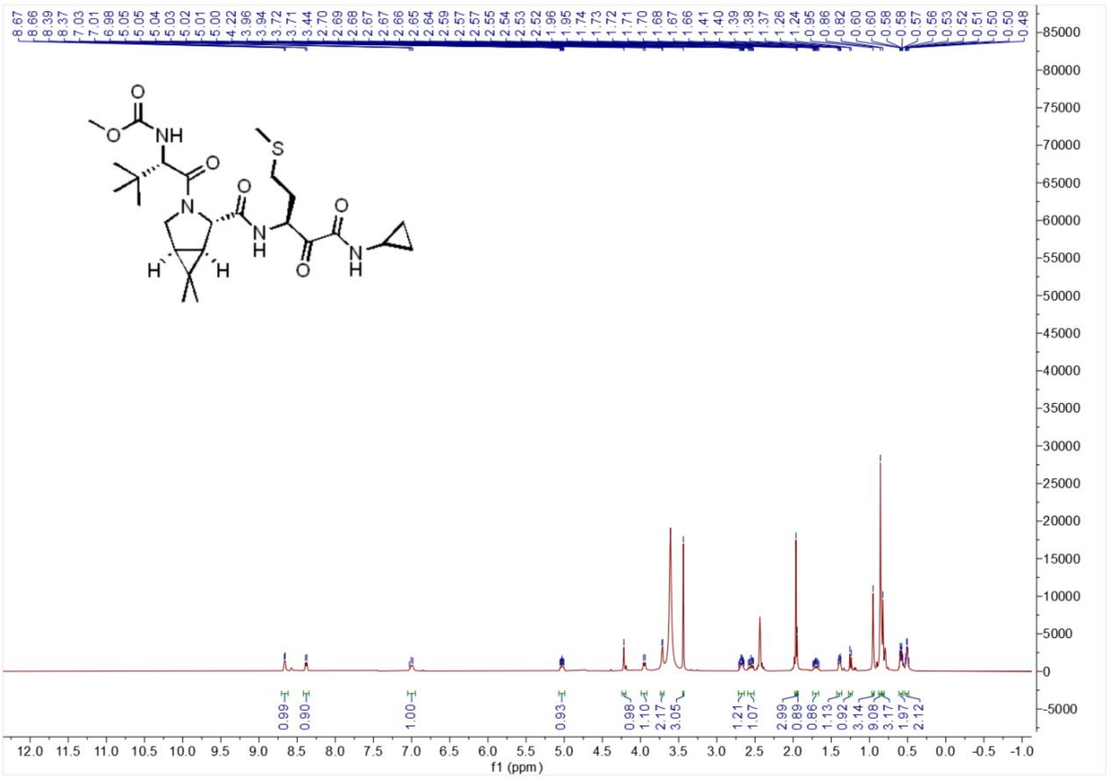

^{1}H NMR spectra of **Jun13735**

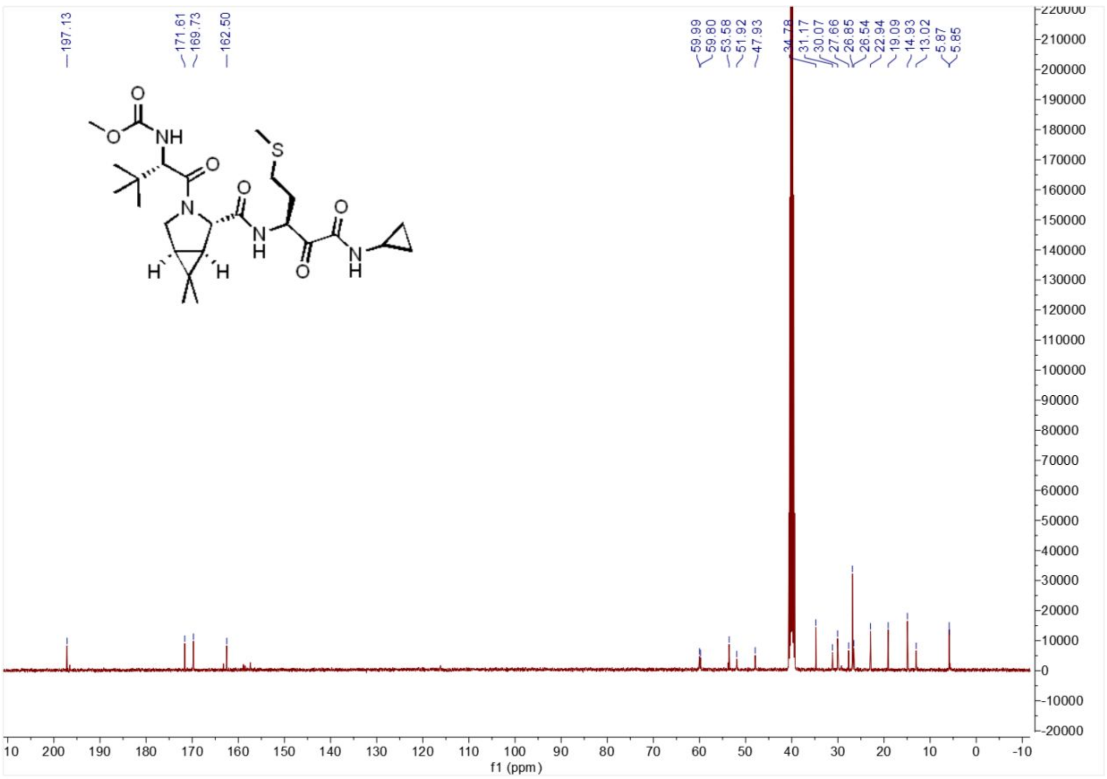

^{13}C NMR spectra of **Jun13735**

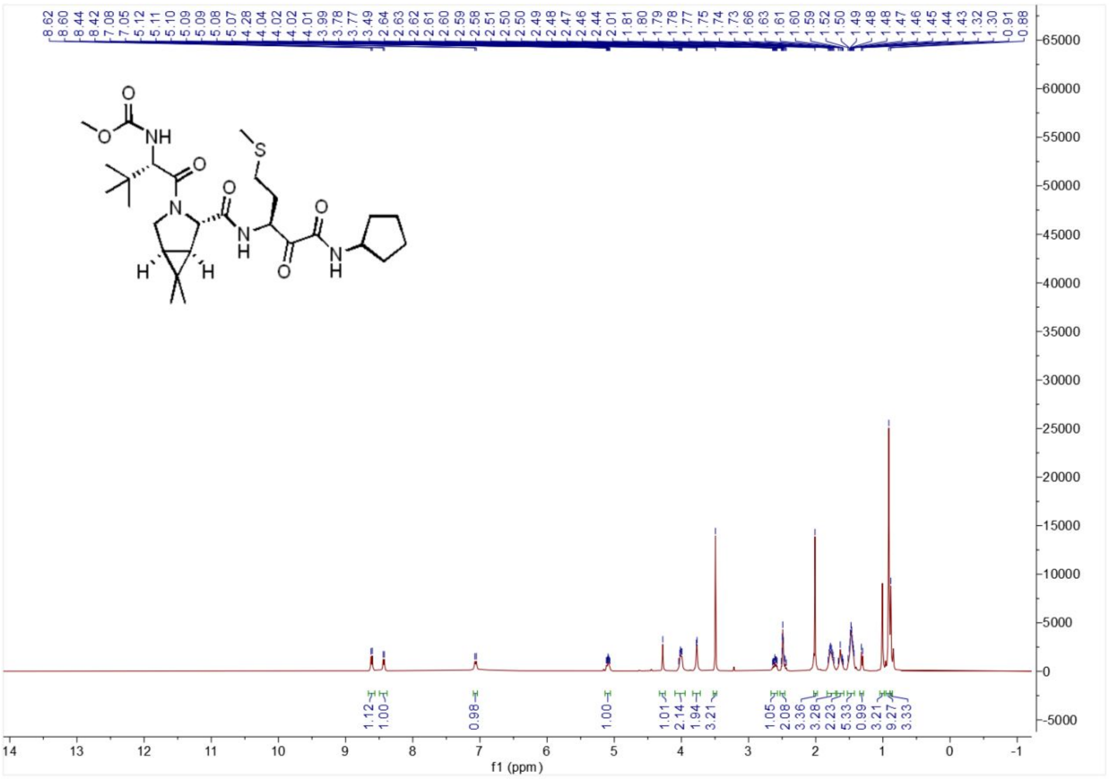

^{1}H NMR spectra of **Jun15494**

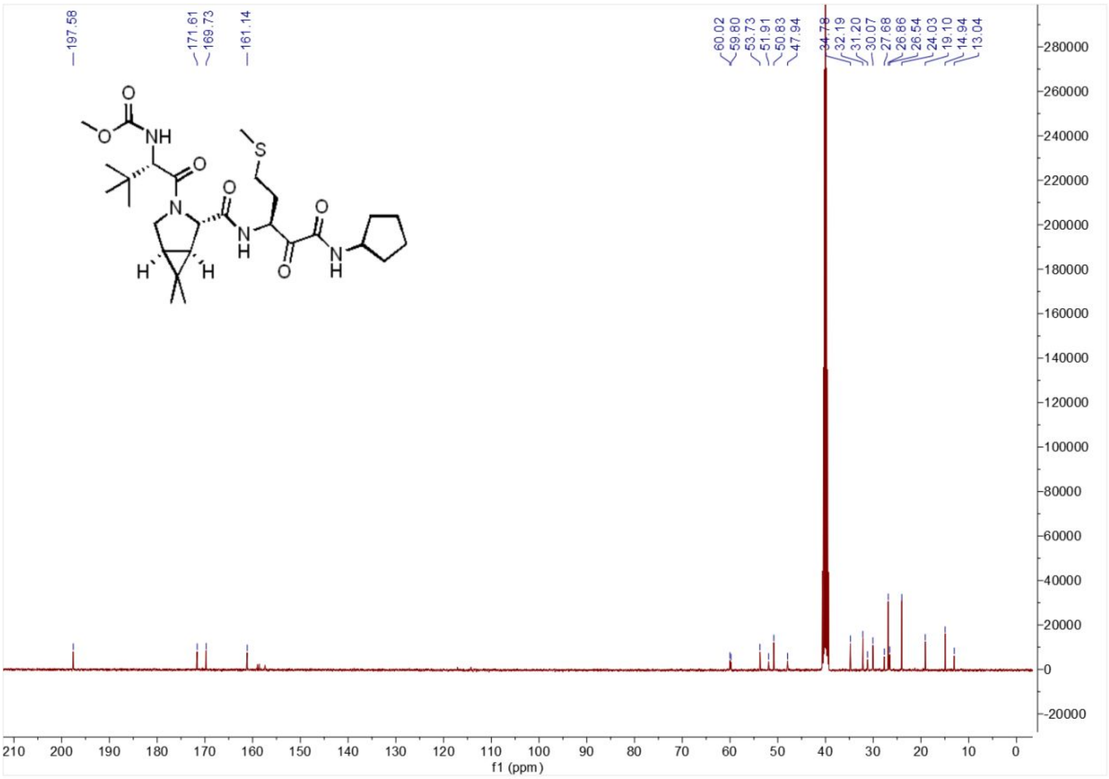

^{13}C NMR spectra of **Jun15494**

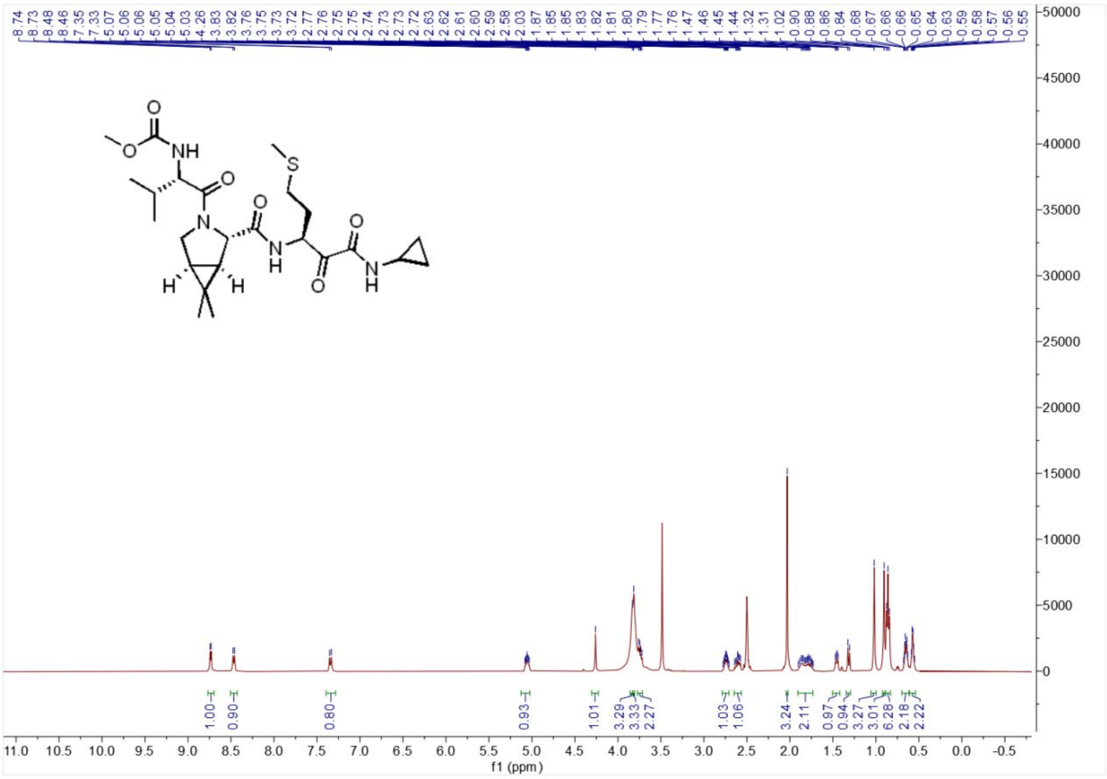

^{1}H NMR spectra of **Jun15573**

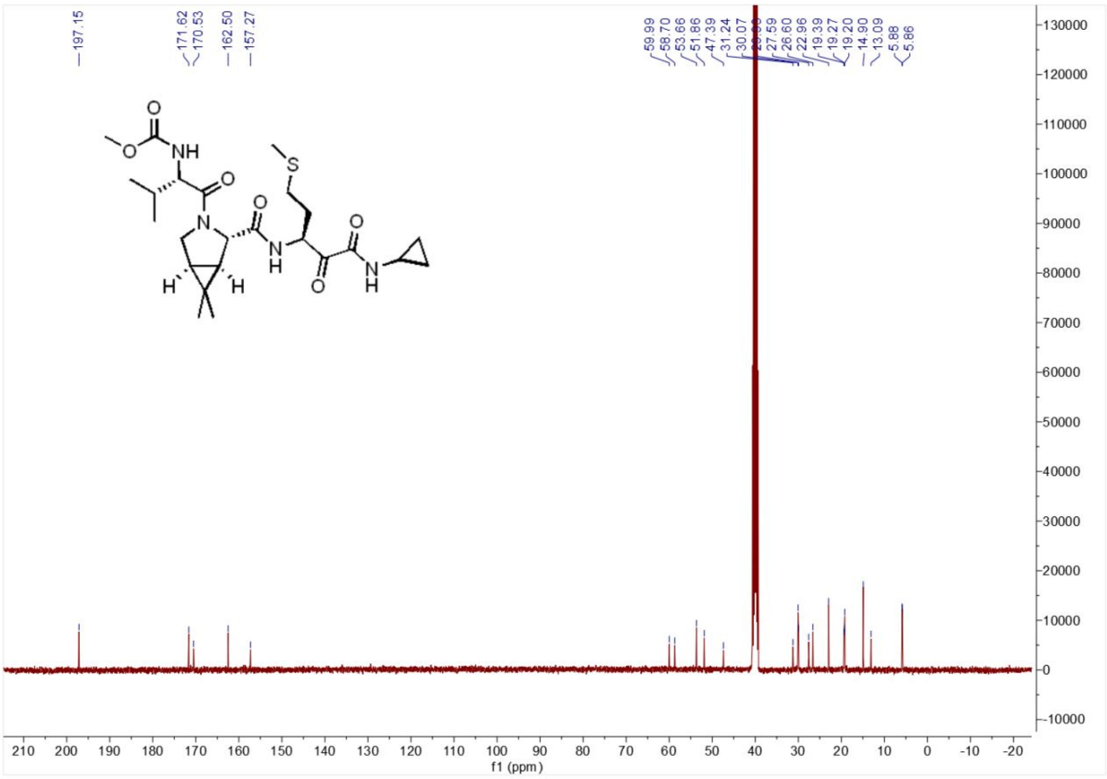

^{13}C NMR spectra of **Jun15573**

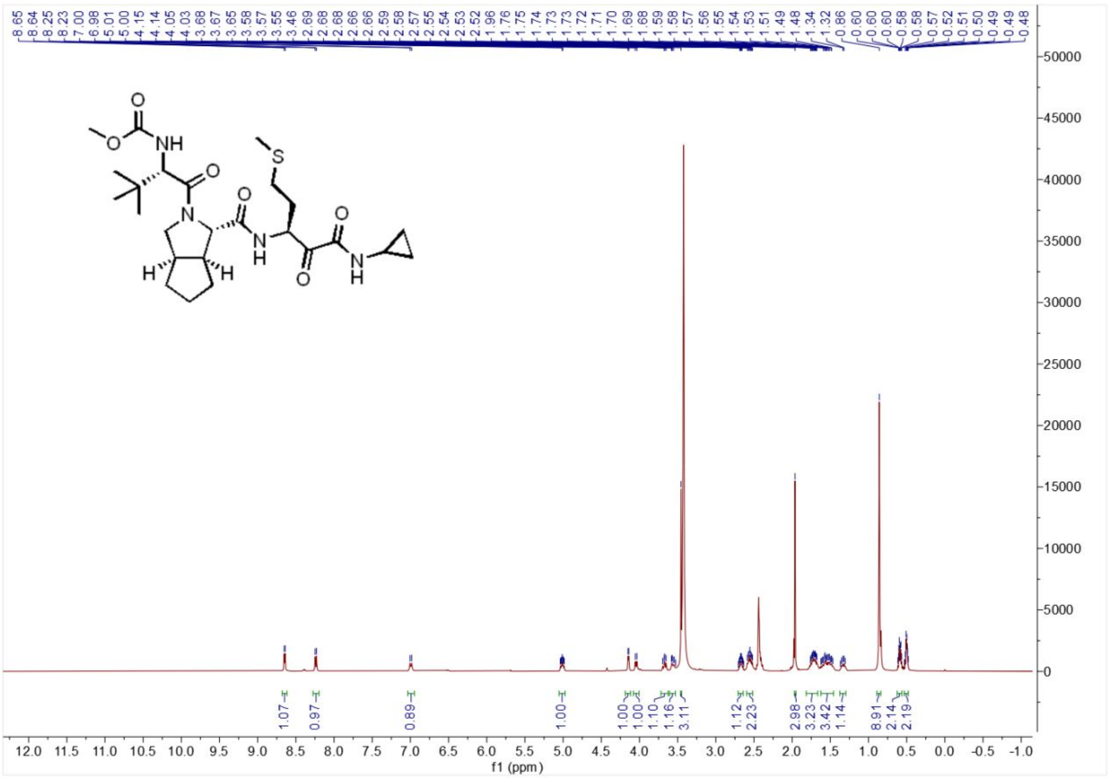

^{1}H NMR spectra of **Jun13857**

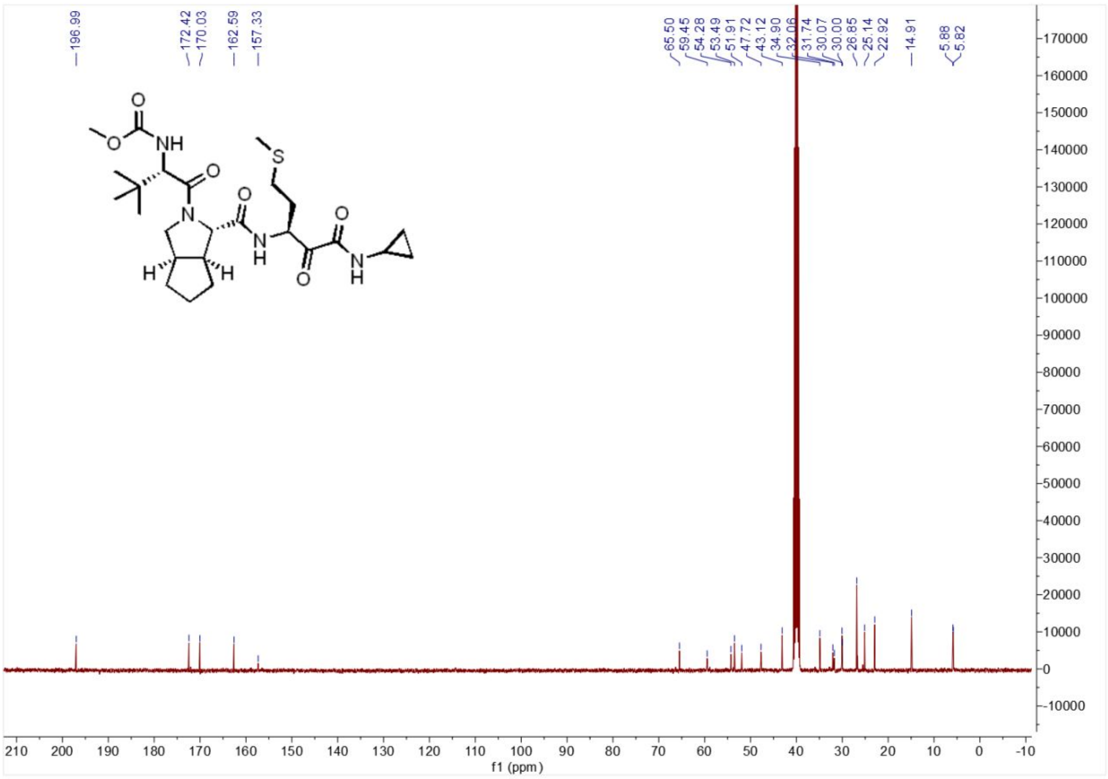

^{13}C NMR spectra of **Jun13857**

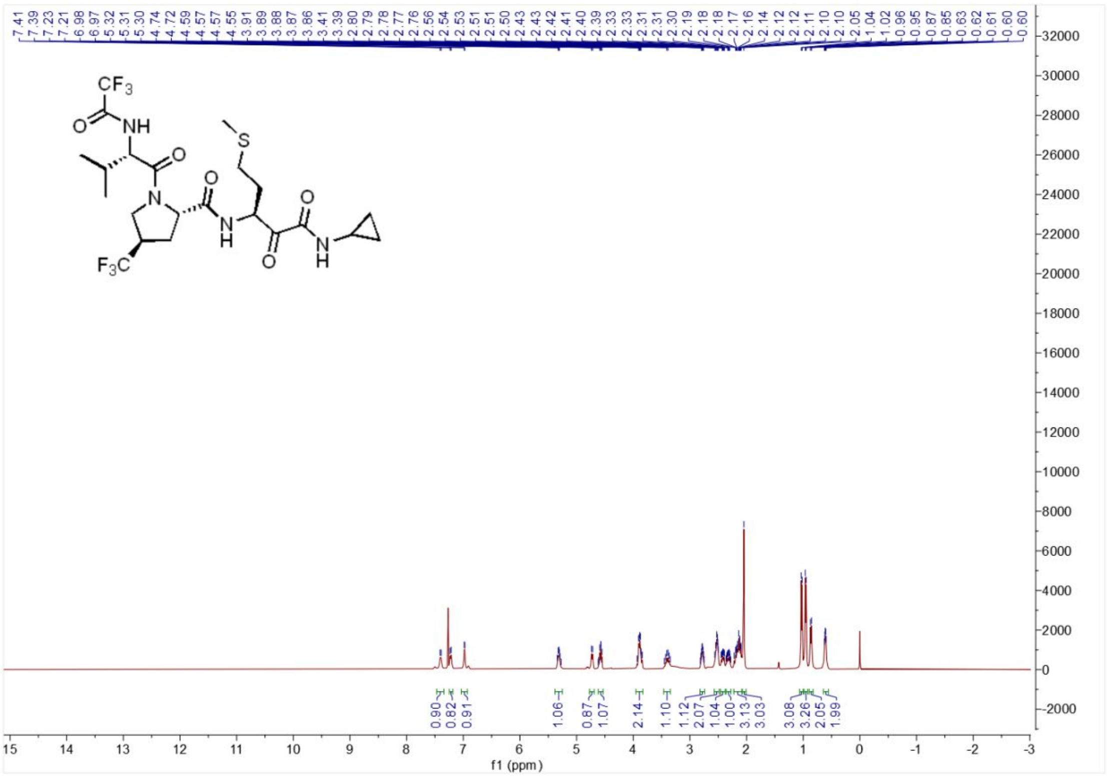

^{1}H NMR spectra of **Jun15667**

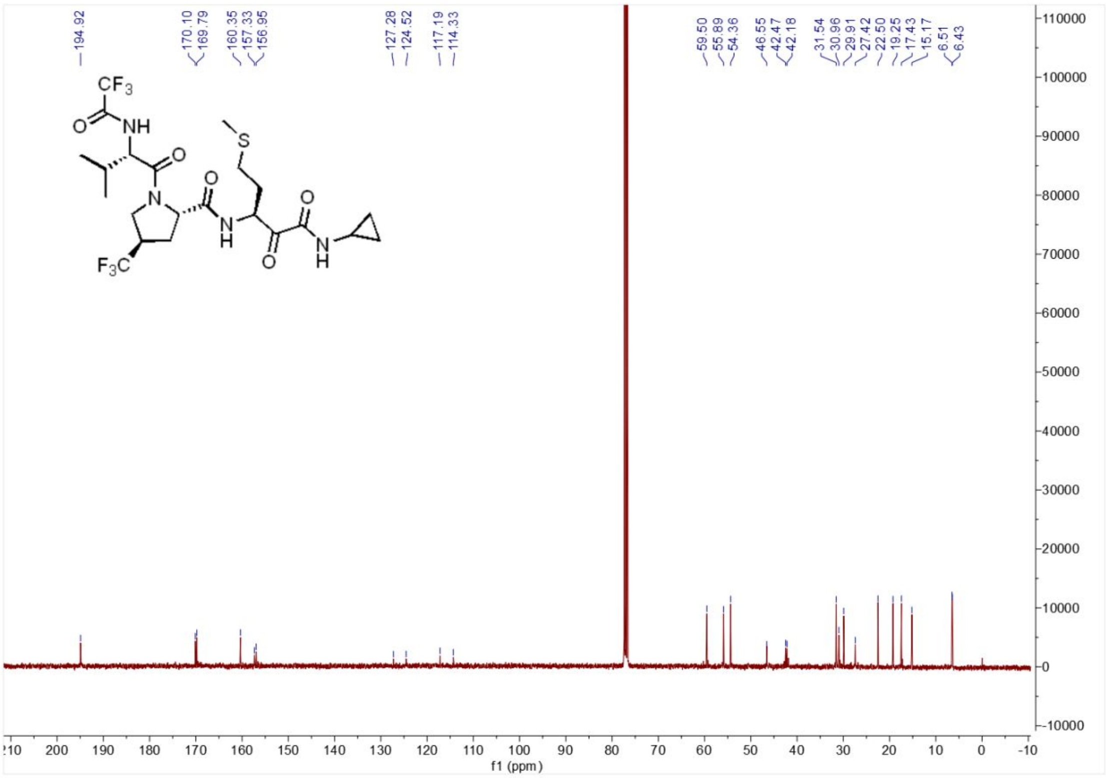

^{13}C NMR spectra of **Jun15667**

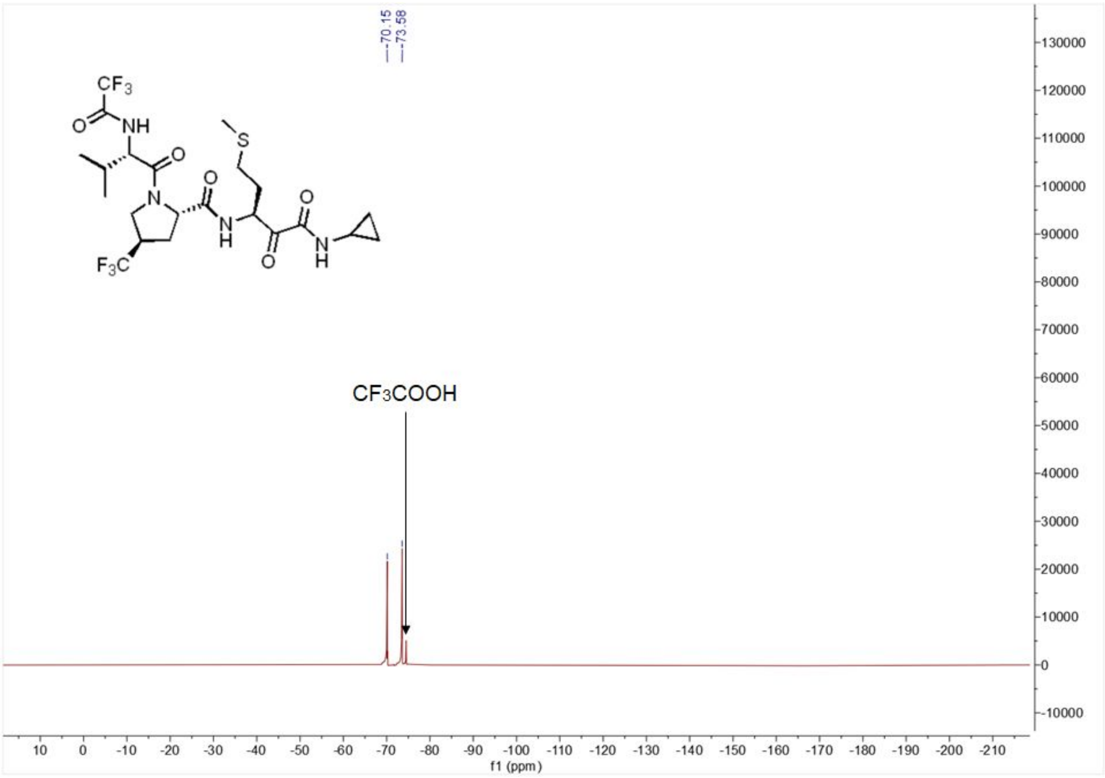

^{19}F NMR spectra of Jun15667

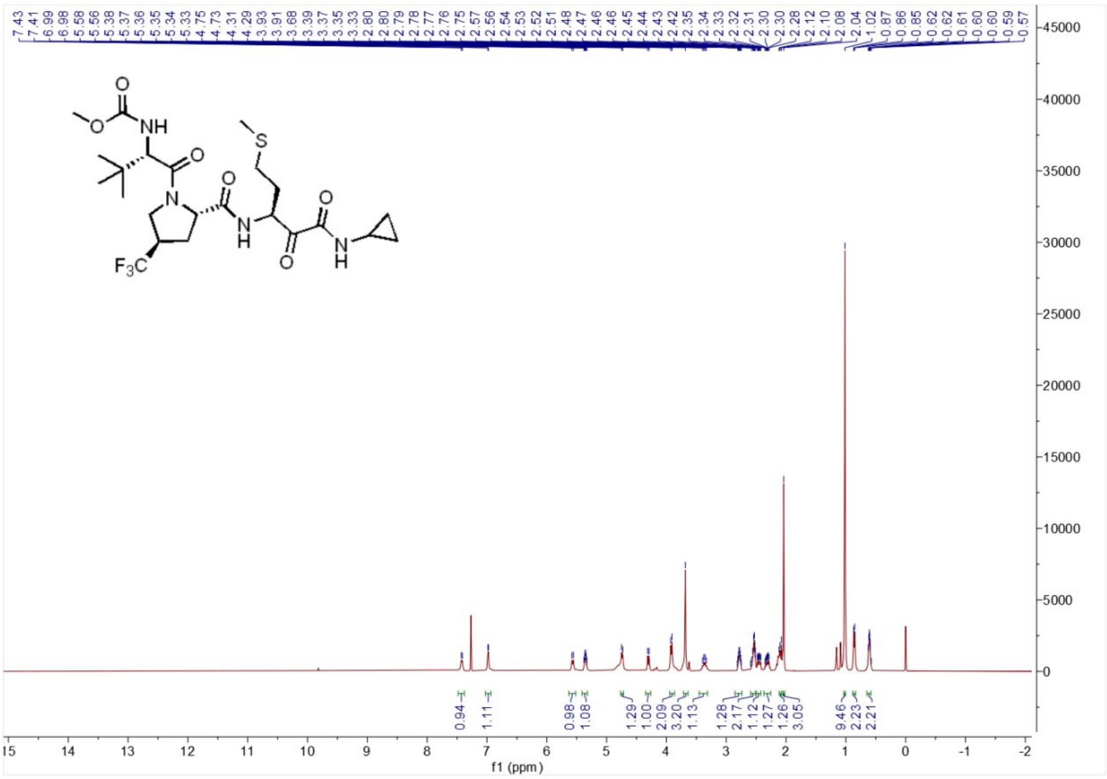

^{1}H NMR spectra of **Jun15635**

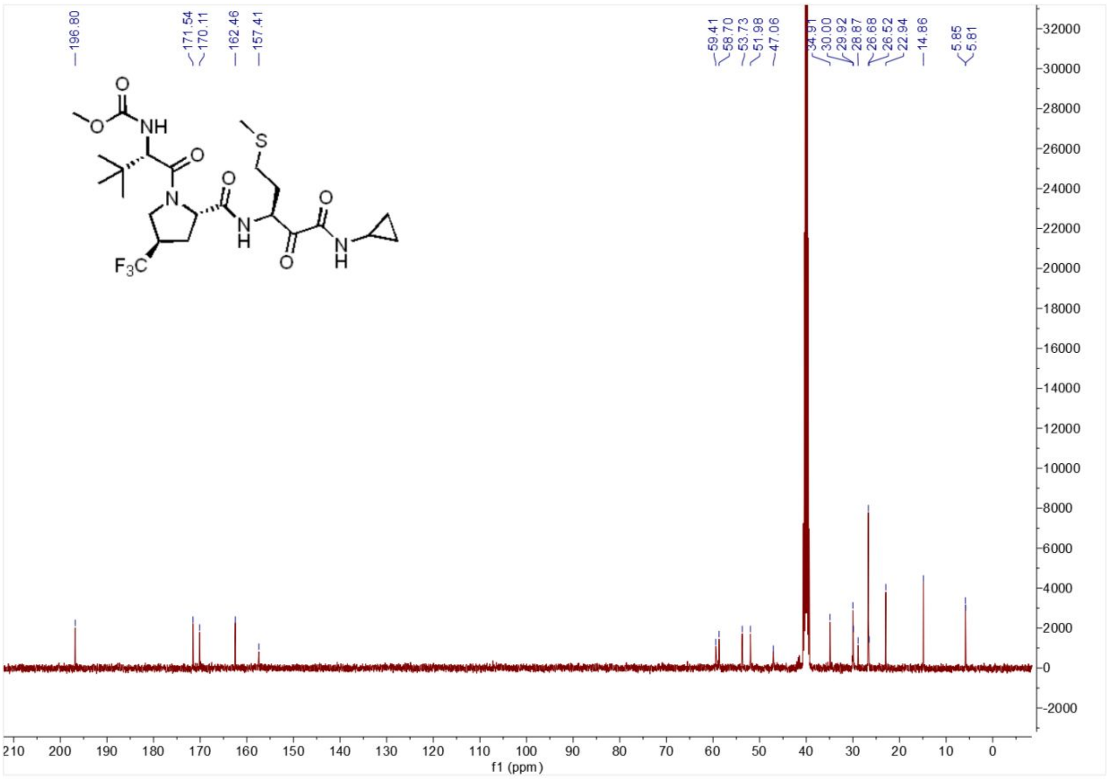

^{13}C NMR spectra of **Jun15635**

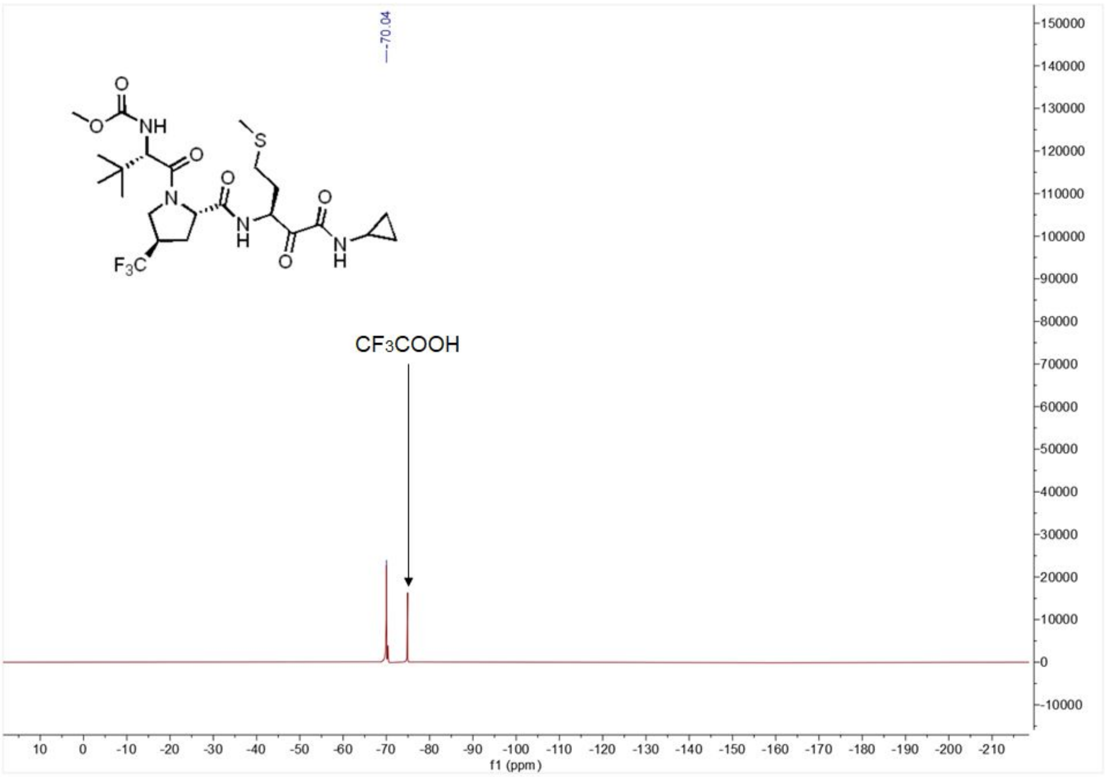

^{19}F NMR spectra of **Jun15635**

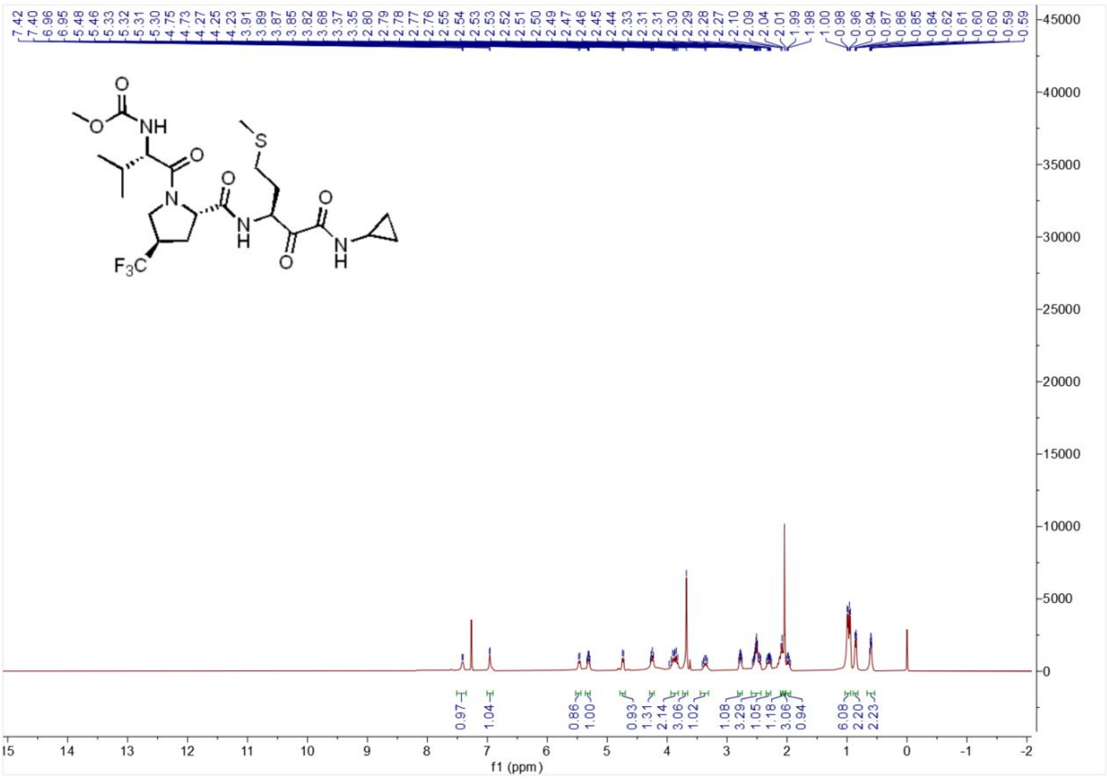

^{1}H NMR spectra of **Jun15636**

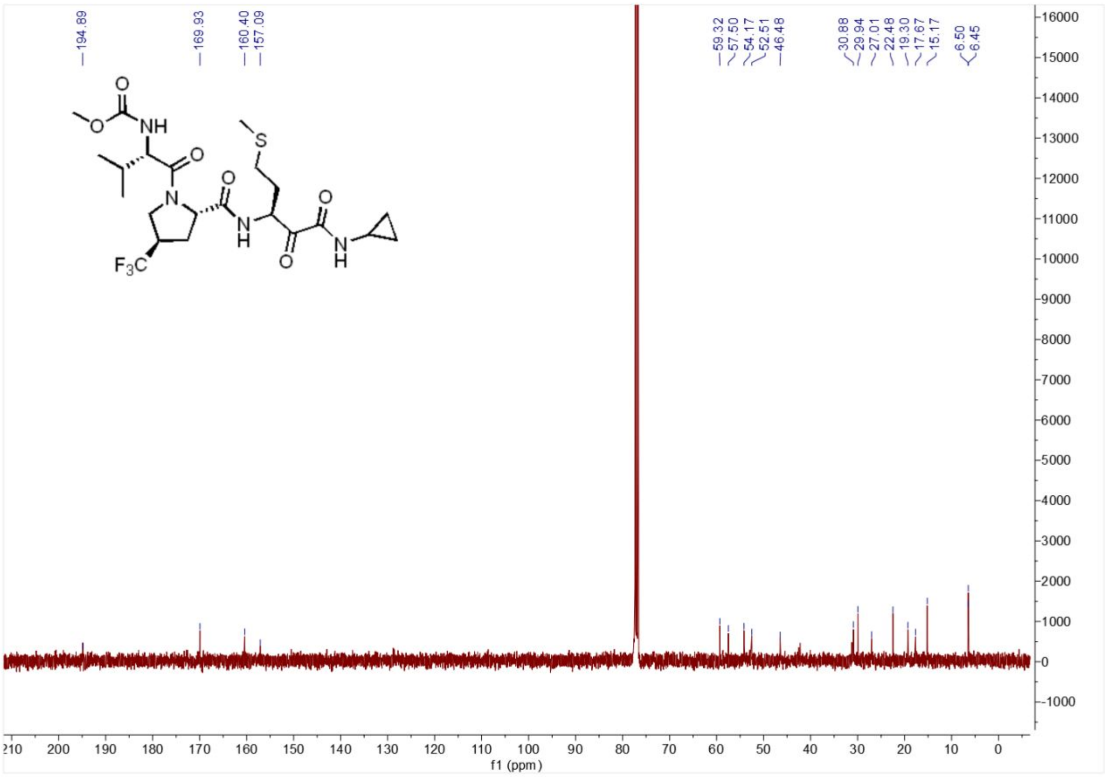

^{13}C NMR spectra of **Jun15636**

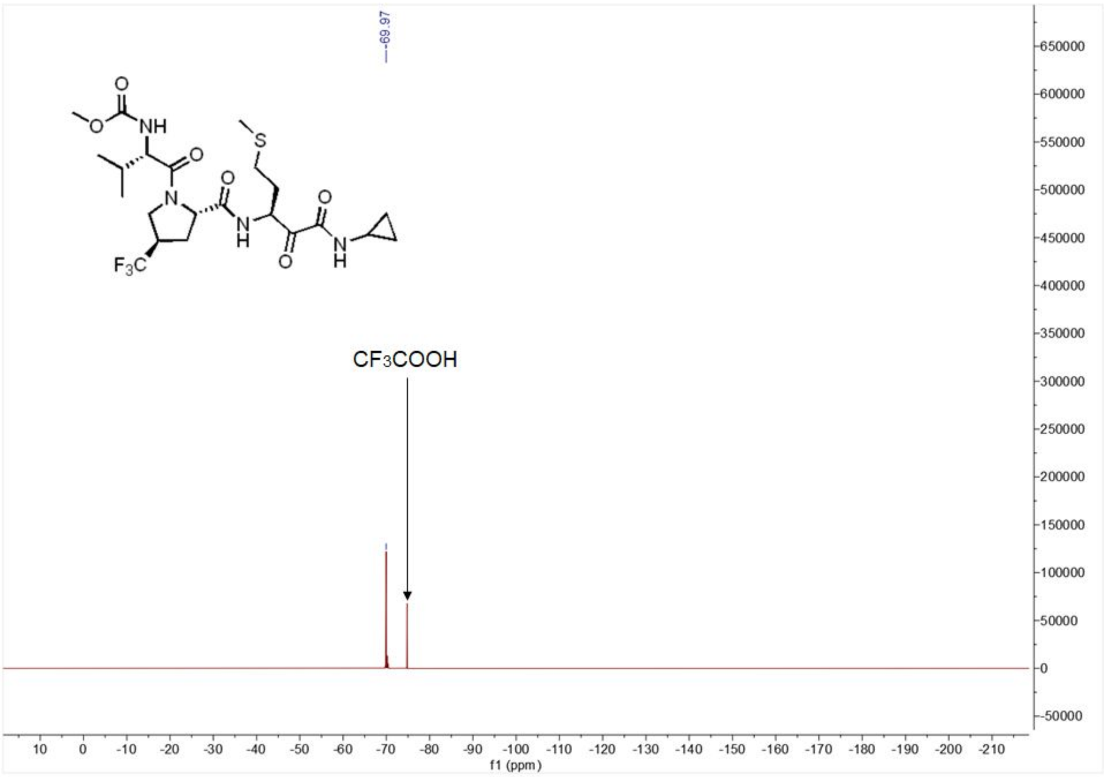

^{19}F NMR spectra of **Jun15636**

References

(1) Sacco, M. D.; Ma, C.; Lagarias, P.; Gao, A.; Townsend, J. A.; Meng, X.; Dube, P.; Zhang, X.; Hu, Y.; Kitamura, N.; et al. Structure and inhibition of the SARS-CoV-2 main protease reveal strategy for developing dual inhibitors against M(pro) and cathepsin L. *Sci Adv* **2020**, *6* (50), eabe0751.

(2) Kabsch, W. Xds. *Acta Crystallogr D Biol Crystallogr* **2010**, *66* (Pt 2), 125-132.

(3) Otwinowski, Z.; Minor, W. Processing of X-ray diffraction data collected in oscillation mode. *Methods Enzymol* **1997**, *276*, 307-326.

(4) Minor, W.; Cymborowski, M.; Otwinowski, Z.; Chruszcz, M. HKL-3000: the integration of data reduction and structure solution--from diffraction images to an initial model in minutes. *Acta Crystallogr D Biol Crystallogr* **2006**, *62* (Pt 8), 859-866.

(5) Emsley, P.; Cowtan, K. Coot: model-building tools for molecular graphics. *Acta Crystallogr D Biol Crystallogr* **2004**, *60* (Pt 12 Pt 1), 2126-2132.

(6) Liebschner, D.; Afonine, P. V.; Baker, M. L.; Bunkóczi, G.; Chen, V. B.; Croll, T. I.; Hintze, B.; Hung, L. W.; Jain, S.; McCoy, A. J.; et al. Macromolecular structure determination using X-rays, neutrons and electrons: recent developments in Phenix. *Acta Crystallogr D Struct Biol* **2019**, *75* (Pt 10), 861-877.

(7) Collaborative Computational Project, N. The CCP4 suite: programs for protein crystallography. *Acta Crystallogr D Biol Crystallogr* **1994**, *50* (Pt 5), 760-763.

(8) Joosten, R. P.; Long, F.; Murshudov, G. N.; Perrakis, A. The PDB_REDO server for macromolecular structure model optimization. *IUCrJ* **2014**, *1* (Pt 4), 213-220.

(9) Rostkowski, M.; Olsson, M. H.; Søndergaard, C. R.; Jensen, J. H. Graphical analysis of pH-dependent properties of proteins predicted using PROPKA. *BMC Struct Biol* **2011**, *11*, 6.

(10) Case, D. A.; Aktulga, H. M.; Belfon, K.; Cerutti, D. S.; Cisneros, G. A.; Cruzeiro, V. W. D.; Forouzesh, N.; Giese, T. J.; Gotz, A. W.; Gohlke, H.; et al. AmberTools. *J Chem Inf Model* **2023**, *63* (20), 6183-6191.

(11) Mobley, D. L.; Bannan, C. C.; Rizzi, A.; Bayly, C. I.; Chodera, J. D.; Lim, V. T.; Lim, N. M.; Beauchamp, K. A.; Slochower, D. R.; Shirts, M. R.; et al. Escaping Atom Types in Force Fields Using Direct Chemical Perception. *J Chem Theory Comput* **2018**, *14* (11), 6076-6092.

(12) Martínez, L.; Andrade, R.; Birgin, E. G.; Martínez, J. M. PACKMOL: a package for building initial configurations for molecular dynamics simulations. *J Comput Chem* **2009**, *30* (13), 2157-2164.

(13) Jorgensen, W. L.; Chandrasekhar, J.; Madura, J. D.; Impey, R. W.; Klein, M. L. Comparison of Simple Potential Functions for Simulating Liquid Water. *Journal of Chemical Physics* **1983**, *79* (2), 926-935, Article.

(14) Tian, C.; Kasavajhala, K.; Belfon, K. A. A.; Raguette, L.; Huang, H.; Migues, A. N.; Bickel, J.; Wang, Y.; Pincay, J.; Wu, Q.; et al. ff19SB: Amino-Acid-Specific

Protein Backbone Parameters Trained against Quantum Mechanics Energy Surfaces in Solution. *J Chem Theory Comput* **2020**, *16* (1), 528-552.
(15) Izaguirre, J. A.; Catarello, D. P.; Wozniak, J. M.; Skeel, R. D. Langevin stabilization of molecular dynamics. *The Journal of Chemical Physics* **2001**, *114* (5), 2090-2098.
(16) Feller, S. E.; Zhang, Y. H.; Pastor, R. W.; Brooks, B. R. Constant-Pressure Molecular-Dynamics Simulation - the Langevin Piston Method. *Journal of Chemical Physics* **1995**, *103* (11), 4613-4621.
(17) Åqvist, J.; Wennerström, P.; Nervall, M.; Bjelic, S.; Brandsdal, B. O. Molecular dynamics simulations of water and biomolecules with a Monte Carlo constant pressure algorithm. *Chemical Physics Letters* **2004**, *384* (4), 288-294.
(18) Ryckaert, J.-P.; Ciccotti, G.; Berendsen, H. J. C. Numerical integration of the cartesian equations of motion of a system with constraints: molecular dynamics of n-alkanes. *J. Comput. Phys.* **1977**, *23* (3), 327-341.
(19) Verlet, L. Computer "Experiments" on Classical Fluids. I. Thermodynamical Properties of Lennard-Jones Molecules. *Physical Review* **1967**, *159* (1), 98-103.
(20) Case, D. A.; Cheatham, T. E., 3rd; Darden, T.; Gohlke, H.; Luo, R.; Merz, K. M., Jr.; Onufriev, A.; Simmerling, C.; Wang, B.; Woods, R. J. The Amber biomolecular simulation programs. *J Comput Chem* **2005**, *26* (16), 1668-1688.
(21) Abraham, M. J.; Murtola, T.; Schulz, R.; Páll, S.; Smith, J. C.; Hess, B.; Lindahl, E. GROMACS: High performance molecular simulations through multi-level parallelism from laptops to supercomputers. *SoftwareX* **2015**, *1-2*, 19-25.
(22) Shirts, M. R.; Klein, C.; Swails, J. M.; Yin, J.; Gilson, M. K.; Mobley, D. L.; Case, D. A.; Zhong, E. D. Lessons learned from comparing molecular dynamics engines on the SAMPL5 dataset. *J Comput Aided Mol Des* **2017**, *31* (1), 147-161.
(23) Evans, D. J.; Holian, B. L. The Nose–Hoover thermostat. *The Journal of Chemical Physics* **1985**, *83* (8), 4069-4074.
(24) Parrinello, M.; Rahman, A. Polymorphic transitions in single crystals: A new molecular dynamics method. *Journal of Applied Physics* **1981**, *52* (12), 7182-7190.
(25) Essmann, U.; Perera, L.; Berkowitz, M. L.; Darden, T.; Lee, H.; Pedersen, L. G. A Smooth Particle Mesh Ewald Method. *Journal of Chemical Physics* **1995**, *103* (19), 8577-8593.
(26) Michaud-Agrawal, N.; Denning, E. J.; Woolf, T. B.; Beckstein, O. MDAnalysis: a toolkit for the analysis of molecular dynamics simulations. *J Comput Chem* **2011**, *32* (10), 2319-2327.
(27) Harris, C. R.; Millman, K. J.; van der Walt, S. J.; Gommers, R.; Virtanen, P.; Cournapeau, D.; Wieser, E.; Taylor, J.; Berg, S.; Smith, N. J.; et al. Array programming with NumPy. *Nature* **2020**, *585* (7825), 357-362.
(28) Humphrey, W.; Dalke, A.; Schulten, K. VMD: visual molecular dynamics. *J Mol Graph* **1996**, *14* (1), 33-38, 27-38.
(29) Vankadara, S.; Dawson, M. D.; Fong, J. Y.; Oh, Q. Y.; Ang, Q. A.; Liu, B.; Chang, H. Y.; Koh, J.; Koh, X.; Tan, Q. W. A warhead substitution study on the

coronavirus main protease inhibitor nirmatrelvir. *ACS medicinal chemistry letters* **2022**, *13* (8), 1345-1350.
(30) 3C-like protease inhibitors block coronavirus replication in vitro and improve survival in MERS-CoV–infected mice. *Science translational medicine* **2020**, *12* (557), eabc5332.
(31) Wang, R.; Shi, H. F.; Zhao, J. F.; He, Y. P.; Liu, J. P. Design, synthesis and aromatase inhibitory activities of novel indole-imidazole derivatives. *Bioorganic & Medicinal Chemistry Letters* **2013**, *23* (6), 1760-1762.
